# Heart failure-induced cognitive dysfunction is mediated by intracellular Ca^2+^ leak through ryanodine receptor type 2

**DOI:** 10.1038/s41593-023-01377-6

**Published:** 2023-07-10

**Authors:** Haikel Dridi, Yang Liu, Steven Reiken, Xiaoping Liu, Elentina K. Argyrousi, Qi Yuan, Marco C. Miotto, Leah Sittenfeld, Andrei Meddar, Rajesh Kumar Soni, Ottavio Arancio, Alain Lacampagne, Andrew R. Marks

**Affiliations:** 1 Department of Physiology and Cellular Biophysics, Clyde and Helen Wu Center for Molecular Cardiology, Columbia University Vagelos College of Physicians & Surgeons, New York, NY USA; 2 Taub Institute for Research on Alzheimer’s Disease and the Aging Brain, Columbia University, New York, NY USA; 3 Proteomics and Macromolecular Crystallography Shared Resource, Herbert Irving Comprehensive Cancer Center, New York, NY USA; 4 Department of Medicine, Columbia University, New York, NY USA; 5 Department of Pathology and Cell Biology, Columbia University, New York, NY USA; 6 PHYMEDEXP, University of Montpellier, CNRS, INSERM, CHU Montpellier, Montpellier, France; 7 LIA1185 CNRS, Montpellier, France

**Keywords:** Mechanisms of disease, Calcium channels, Cerebrovascular disorders

## Abstract

Cognitive dysfunction (CD) in heart failure (HF) adversely affects treatment compliance and quality of life. Although ryanodine receptor type 2 (RyR2) has been linked to cardiac muscle dysfunction, its role in CD in HF remains unclear. Here, we show in hippocampal neurons from individuals and mice with HF that the RyR2/intracellular Ca^2+^ release channels were subjected to post-translational modification (PTM) and were leaky. RyR2 PTM included protein kinase A phosphorylation, oxidation, nitrosylation and depletion of the stabilizing subunit calstabin2. RyR2 PTM was caused by hyper-adrenergic signaling and activation of the transforming growth factor-beta pathway. HF mice treated with a RyR2 stabilizer drug (S107), beta blocker (propranolol) or transforming growth factor-beta inhibitor (SD-208), or genetically engineered mice resistant to RyR2 Ca^2+^ leak (RyR2-p.Ser2808Ala), were protected against HF-induced CD. Taken together, we propose that HF is a systemic illness driven by intracellular Ca^2+^ leak that includes cardiogenic dementia.

## Abstract (web summary)

Dridi et al. identified a mechanism for cognitive dysfunction after heart failure in which hyper-adrenergic signaling and transforming growth factor-beta activation induced Ca^2+^ leak by RyR2 channels in hippocampal neurons.

## Main

HF is the most rapidly growing cardiovascular disorder affecting millions worldwide^1,2^, with associated high rates of mortality, poor quality of life, and high health care costs due to decreased cardiac function and dysfunction of other organ systems^3–5^. Recent studies suggest that CD in HF, known as ‘cardiogenic dementia’ may be caused by HF itself, with a prevalence of 20–80%^6,7^.

CD includes forgetfulness and poor learning ability, which may impair self-care^8–10^ and compliance^11,12^ in as many as 90% of those with HF. Noncompliance increases the risk of mortality and morbidity^13^. Indeed, CD impairs the ability of individuals with HF to make decisions in critical situations, such as early recognition and interpretation of worsening symptoms, and making appropriate decisions about their health. People with HF and preserved ejection fraction also exhibit CD^14^, including verbal memory and executive function deficits, known as cognitive inflexibility^15^. Structural changes in the brain including atrophy, increased white matter hyper-intensities, gray matter loss and silent cerebral infarction, are frequently observed in HF patients with CD^16,17^. Interestingly, these structural and functional changes coincide with a chronic inflammatory response and neurohormonal activation including the renin–angiotensin–aldosterone system and the adrenergic pathway^18^. Furthermore, clinical studies have linked cardiovascular diseases, dementia and Alzheimer’s disease through common triggers, including inflammation, oxidative stress, hypoxia^19^ and adrenergic signaling^20–22^.

Indeed, norepinephrine modulates the levels of consciousness^23,24^. The sympathetic nervous system is continuously activated in patients with HF^25^ and is known to be part of a major upstream signaling pathway that alters intracellular Ca^2+^ homeostasis and tightly controls neuronal cellular function and survival. Ca^2+^ dyshomeostasis is a hallmark of neurodegenerative diseases, including Alzheimer’s disease^26^, Huntington’s disease^27^ and Parkinson’s disease^28^. Intracellular Ca^2+^ signaling plays a role in regulating long-term potentiation (LTP), long-term depression and neurodegeneration^29–31^.

In neurons, activation of inositol-1,4,5-trisphosphate receptors (IP_3_Rs) and RyRs amplifies intracellular Ca^2+^ signals^32^. Increased intracellular Ca^2+^ concentration activates Ca^2+^-dependent processes involved in plasticity and synaptic transmission that are required for learning and memory^33^. RyR2, the Ca^2+^-activated intracellular Ca^2+^ release channel on the sarcoplasmic reticulum (SR) or endoplasmic reticulum (ER), is a homotetrameric macromolecular protein complex that includes four RyR2 monomers, 565-kDa polypeptide each^34^. The RyR2 channel is regulated by kinases and phosphatases^35^, phosphodiesterase^36^, calmodulin^37^, and the stabilizing subunit calstabin2 (FKBP12.6)^35^. Protein kinase A (PKA) and Ca^2+^/calmodulin-dependent protein kinase II (CAMKII) tether to RyR2 and phosphorylate the channel at Ser2808 and Ser2814, respectively^35,38^. PKA hyper-phosphorylation and/or oxidation/nitrosylation of RyR2 cause calstabin2 dissociation, leading to leaky channels that do not close properly^35,39^.

We^40^ and others have previously reported that RyR channels are dysfunctional not only in the cardiomyocytes of patients with HF^35,41^ but also in the skeletal muscle^42,43^, suggesting the existence of a common mechanism that primarily affects RyR2 in the cardiac muscle and propagates to affect RyRs in other organs expressing different isoforms of the channels, such as RyR2 in the pancreatic beta cells^44^ (may cause diabetes) and in the brain (may impair cognitive function), and RyR1 in the diaphragm/lung (may cause respiratory disorders) and locomotor muscle (may cause exercise intolerance and muscle fatigue).

In this study, we found that the hyper-adrenergic state and the enhanced inflammatory response in HF caused neuronal RyR2-mediated intracellular Ca^2+^ leak that subsequently affected cognition and memory. Neuronal Ca^2+^ dyshomeostasis increased mitochondrial Ca^2+^ content, contributing to oxidative overload, and altered the expression of key genes involved in cognitive function. Stabilizing leaky RyR2 channels using a small-molecule Rycal drug S107 prevented cognitive impairment induced by HF.

## Results

### Neuronal RyR2 channels are leaky in individuals with heart failure

**Fig. 1:**
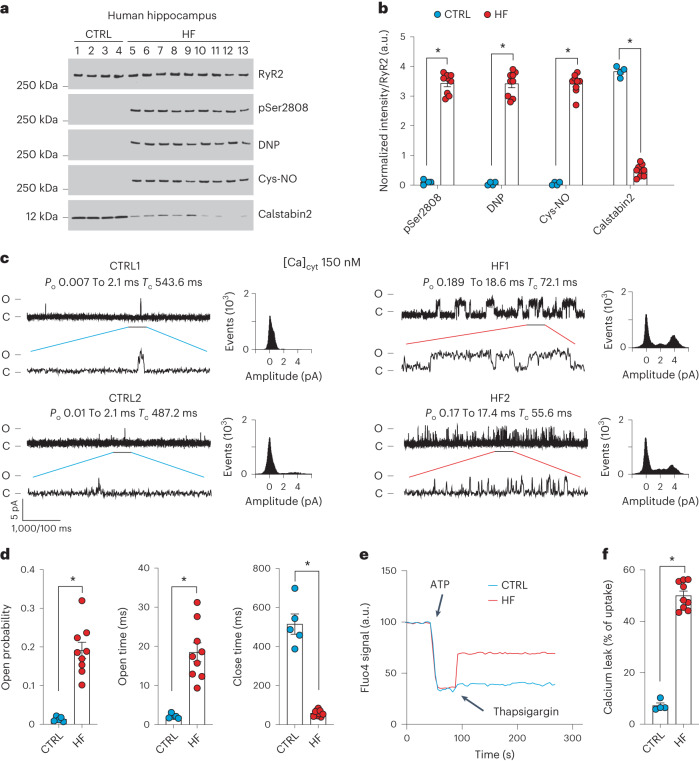
Hippocampal RyR2 channels are remodeled and leaky in patients with heart failure. **a**,**b**, Representative SDS–PAGE analysis and quantification of modified RyR2 and calstabin2 immunoprecipitated from hippocampi of controls and individuals with HF (IP RyR2; bands normalized to total RyR2). Control (CTRL), *n* = 4); HF, *n* = 9. **c**, Single-channel recordings of RyR2 incorporated in planar lipid bilayers with 150 nM Ca^2+^ in the *cis* chamber, corresponding to representative experiments performed using human hippocampal samples from controls and HF patients (two traces from two different controls and individuals with HF are shown) **d**, *P*_o_, *T*_o_ and *T*_c_ of RyR2 channels in controls (*n* = 5) and HF (*n* = 9) hippocampi. **e**, ER Ca^2+^ leak measured in microsomes from control (*n* = 4) and HF participant (*n* = 9) hippocampi. **f**, Bar graphs represent the quantification of microsomal Ca^2+^ leak as the percentage of uptake in controls (*n* = 5) and HF individuals (*n* = 9). Individual values are shown with the mean ± s.e.m. (*t*-test *P < 0.05, controls versus HF individuals). Data are derived from biologically independent samples. All statistical tests were two sided. a.u., arbitrary units. Source data

To evaluate RyR2 in the brains of individuals with HF, hippocampal biopsy samples from controls (non-HF) and de-identified individuals with HF (Supplementary Tables 1 and 2) were obtained from the Brain Bank at Columbia University and the National Institutes of Health (NIH) Neuro-Biobank. Immunoprecipitated RyR2 and isolated ER fractions were used to analyze the composition of the hippocampal RyR2 macromolecular complex and PTMs known to be associated with RyR channel Ca^2+^ leak^31,32,35^. Hippocampal RyR2 from individuals with HF (*n* = 9) exhibited PKA hyper-phosphorylation (on Ser2808), oxidation, cysteine nitrosylation, and were depleted of calstabin2, compared to controls (*n* = 4; Fig. 1a,b). This is the ‘biochemical signature’ of ‘leaky’ RyR2 channels^35,45^. Single-channel recordings of hippocampal RyR2, reconstituted into planar lipid bilayers, revealed increased open probability (*P*_o_), increased mean open time (*T*_o_) and decreased mean closed time (*T*_c_) in individuals with HF compared to controls (*P*_o _= 0.19% ± 0.02%, *T*_o_ = 18 ± 2 ms, *T*_c_ = 58 ± 05 ms in HF hippocampi, *n* = 9; versus *P*_o_ = 0.01% ± 0.002%, *T*_o_ = 2 ± 0.2 ms, *T*_c_ = 515 ± 52 ms in controls, *n* = 4; *P* < 0.05) in the presence of low, non-activating [Ca^2+^]_*cis*_ (150 nM), conditions under which normal RyR2 channels are tightly closed (Fig. 1c,d). This elevated *P*_o_ is consistent with pathological hippocampal ER Ca^2+^ leak^26,31^. Indeed, neuronal microsomes from individuals with HF exhibited increased RyR-mediated ER Ca^2+^ leak compared with controls (Fig. 1e,f).

### Impaired cognitive function in mouse model of heart failure

**Fig. 2:**
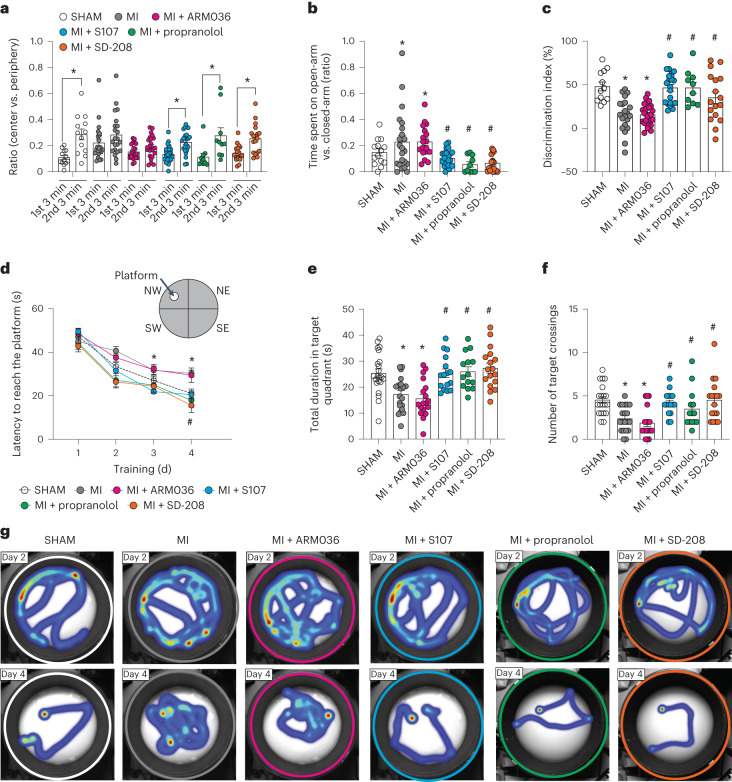
Mouse model of heart failure (myocardial infarction) is associated with cognitive dysfunction. **a**, Open field test of mice operated SHAM (*n* = 13), MI (*n* = 22), MI treated with ARM036 (MI + ARM036, *n* = 23), MI treated with S107 (MI + S107, *n* = 24), MI treated with propranolol (MI + propranolol, *n* = 10) and MI treated with TGF-β inhibitor (MI + SD-208, *n* = 18). Ratios of total time spent in the center area versus periphery area within first 3 min and second 3 min are shown. **b**, EPM test showing the ratios of time spent in the open-arm versus closed-arm in SHAM (*n* = 14), MI (*n* = 22), MI + ARM036 (*n* = 18), MI + S107 (*n* = 23), MI + propranolol (*n* = 10) and MI + SD-208 (*n* = 18) mice. **c**, Novel object recognition test showing the discrimination index in SHAM (*n* = 12), MI (*n* = 21), MI + ARM036 (*n* = 23), MI + S107 (*n* = 22), MI + propranolol (*n* = 10) and MI + SD-208 (*n* = 17) mice. **d**, MWM test (learning curves for 4 d) in SHAM (*n* = 22), MI (*n* = 20), MI + ARM036 (*n* = 19), MI + S107 (*n* = 19), MI + propranolol (*n* = 14) and MI + SD-208 (*n* = 19) mice. **e**, Probe trials after escape platform removed showing the total duration spent in the target quadrant at day 5 in SHAM (*n* = 22), MI (*n* = 20), MI + ARM036 (*n* = 18), MI + S107 (*n* = 17), MI + propranolol (*n* = 14) and MI + SD-208 (*n* = 17) mice. **f**, Number of target crossings at day 5 in SHAM (*n* = 20), MI (*n* = 20), MI + ARM036 (*n* = 18), MI + S107 (*n* = 16), MI + propranolol (*n* = 14) and MI + SD-208 (*n* = 17) mice. **g**, Heat maps showing the latency for each group at day 2 and day 4. Individual values are shown with mean ± s.e.m. Two-tailed *t*-test *P < 0.05 in **a** shows significance between the first 3 min and second 3 min of each group. One-way analysis of variance (ANOVA) was used to compare the difference between the six groups in **b**, **c**, **e** and **f**; Tukey’s test was used for multiple comparisons; two-way ANOVA was used in **d**. Tukey’s test post hoc correction for multiple comparisons was used. *P < 0.05, SHAM versus MI or MI + ARM036; ^#^*P* < 0.05, MI versus MI + S107, MI + propranolol or MI + SD-208. All statistical tests were two sided. Data are derived from biologically independent samples. Source data

Because of the complexity of the clinical manifestation of HF patients, we used the mouse model of HF (myocardial infarction, MI) with reduced ejection fraction^46^ (Supplementary Table 3) to evaluate the mechanisms of CD. We used the open field test and elevated plus maze (EPM) test to evaluate the behavioral phenotypes, and spontaneous exploratory activity in the mice^31,47^. In the open field test, mice were placed at the center of a chamber and allowed to explore for 6 min. Within the first and the second 3 min, the ratio of time spent in the center versus periphery area for MI mice was similar (0.22 ± 0.02 and 0.28 ± 0.03, *n* = 22, *P* = 0.18), whereas these ratios were significantly different in the SHAM group (0.1 ± 0.01 and 0.28 ± 0.04, *n* = 13, *P* < 0.05; Fig. 2a). In the EPM test, MI mice spent more time in the open arms of the EPM (Fig. 2b) compared to SHAM (0.22 ± 0.04 versus 0.14 ± 0.02; *P* < 0.05). The abnormal behaviors were prevented by the pharmacological treatments using the RyR2 stabilizer Rycal compound S107, the nonselective beta-adrenergic antagonist propranolol, and the anti-inflammatory transforming growth factor-beta (TGF-β) inhibitor SD-208 (Fig. 2a,b). As a control, we tested the effects of a closely related Rycal ARM036, which has the same mechanism of action as S107 but does not cross the blood–brain barrier (BBB)^27^. ARM036 had no effect on MI mouse cognitive performance in either task (Fig. 2a,b) indicating that the S107 effects were at the CNS level rather than the systemic level.

Next, a novel object recognition test was used to evaluate hippocampal-dependent short-term memory^31,48^. MI mice showed significantly lower discrimination index (15% ± 4%) compared to the SHAM group (48% ± 5%, *P* < 0.05; Fig. 2c). S107, propranolol and SD-208 treatments prevented short-term memory loss by increasing the discrimination index to 46% ± 4%, 46% ± 6% and 35% ± 6%, respectively (*P* < 0.05), whereas ARM036 did not increase the discrimination index (15% ± 3%; *P* = 0.25; Fig. 2c).

A Morris water maze (MWM) test was performed to assess hippocampal-dependent long-term spatial learning and memory^31,49^. MI mice exhibited significantly prolonged latency to find and reach the hidden platform on day 4 training trials (30 ± 1 s, *n* = 20) compared to the SHAM controls (21 ± 2 s, *P* *<* 0.05, *n* = 22). S107, propranolol and SD-208 treatments significantly reduced latency on day 4 (20 ± 2 s, 17 ± 3 s and 15 ± 3 s, respectively, *P* < 0.05, *n* = 19, 14 and 19 per group). Again, MI mice treated with ARM036 did not show significant reduction on the latency to reach the hidden platform (29 ± 3 s, *P* = 0.99, *n* = 19; Fig. 2d).

A probe trial was performed on day 5 of the MWM test. MI mice spent a significantly shorter duration in target quadrant (17 ± 1 s, *n* = 19) and exhibited a reduced number of target crossings (2.4 + 0.3, *n* = 19) within the 60-s probe trial compared to SHAM (25 ± 2 s and 4.6 ± 0.3, *P* < 0.05, *n* = 20; Fig. 2e,f). S107, propranolol and SD-208 treatments, but not ARM036, were able to correct the spatial memory deficit in MI by improving the time spent in the target quadrant (26 ± 2 s, 26 ± 2 s and 27 ± 2 s, respectively, *P* < 0.05) and target crossings (4.2 ± 0.3, 3.5 ± 0.6 and 4.5 ± 0.6, respectively, *P* < 0.05, *n* = 14–19 per group) in the probe trial (Fig. 2e,f). A heat map of the swimming behavior of these mice is shown at days 2 and 4 (Fig. 2g). Of note, the traveled distance and velocity of mice in the open field, EPM and MWM tests were comparable between all the groups (Supplementary Table 4).

### Neuronal RyR2 channels are leaky in the mouse model of heart failure

**Fig. 3:**
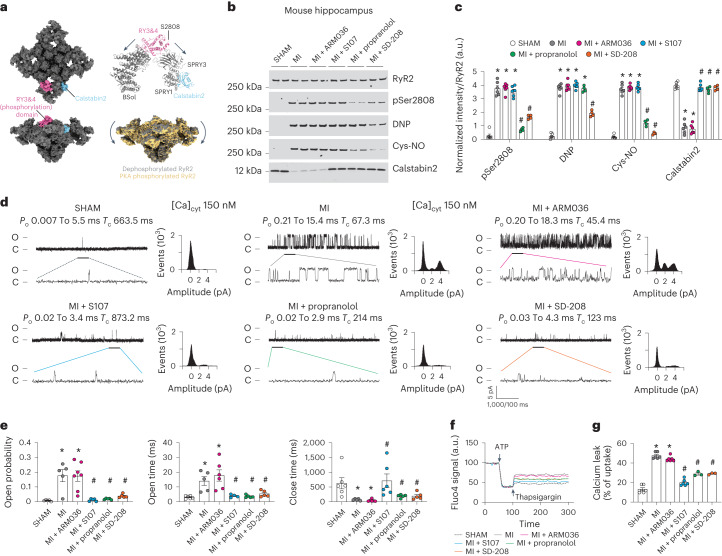
Mouse model of heart failure is associated with leaky hippocampal RyR2. **a**, Cryogenic electron microscopy structure of RyR2 (gray, top and side view) showing the location of the Ser2808 in the RY3&4 phosphorylation domain (magenta) and calstabin2 (cyan). RyR2 PKA phosphorylation shifted the channel toward a primed state (yellow)^50^. **b**,**c**, Representative SDS–PAGE analysis and quantification of modified RyR2 and calstabin2 immunoprecipitated from hippocampal RyR2 complex (IP RyR2; bands normalized to total RyR2) in SHAM (*n* = 6), MI (*n* = 6), MI + ARM036 (*n* = 6), MI + S107 (*n* = 6), MI+ propranolol (*n* = 4) and MI + SD-208 (*n* = 4) mice. **d**, Single-channel traces of RyR2 incorporated in planar lipid bilayers with 150 nM Ca^2+^ in the *cis* chamber, corresponding to representative experiments performed with hippocampal samples from SHAM (*n* = 6), MI (*n* = 5), MI + ARM036 (*n* = 6), MI + S107 (*n* = 5), MI + propranolol (*n* = 5) and MI + SD-208 (*n* = 5) mice. **e**, RyR2 *P*_o_, *T*_o_ and *T*_c_ in the same groups. **f**, Ca^2+^ leak measured in microsomes from mouse hippocampi of the same groups. **g**, Bar graphs represent the quantification of Ca^2+^ leak as the percentage of uptake in SHAM (*n* = 6), MI (*n* = 6), MI + ARM036 (*n* = 6), MI + S107 (*n* = 6), MI + propranolol (*n* = 3) and MI + SD-208 (*n* = 3) mice. Individual values are shown with the mean ± s.e.m. One-way ANOVA and Tukey’s test post hoc correction for multiple comparisons shows *P < 0.05, SHAM versus MI or MI + ARM036; ^#^*P* < 0.05, MI versus MI + S107, MI + propranolol or MI + SD-208. Data are derived from biologically independent samples. All statistical tests were two sided. Source data

We have recently solved the high-resolution three-dimensional (3D) structure of human RyR2 and showed that the PKA-phosphorylated channels on Ser2808 adopt a primed state (halfway between the closed and the open states), allowing the opening of channels at a lower cytosolic Ca^2+^ concentration resulting in leaky channels^50^ (Fig. 3a). Furthermore, we identified the binding site of the Rycal drugs (S107 and ARM210)^50,51^ on RyR1 and RyR2 and have shown that these drugs are able to reverse the primed state of leaky channels toward a fully closed state^50^. We then evaluated the PTMs and functional abnormalities of neuronal RyR2 in HF hippocampal samples from MI mice and compared them to SHAM. Hippocampal RyR2 was immunoprecipitated and immunoblotted to detect PTMs. Neuronal RyR2 from MI mice exhibited PKA hyper-phosphorylation (on Ser2808, the main PKA-phosphorylation site^50^), oxidation, cysteine nitrosylation and calstabin2 depletion compared to the SHAM hippocampal samples (*P* < 0.05; Fig. 3b,c). Single-channel recordings of neuronal RyR2 from MI mice revealed increased open probability (*P*_o _= 0.17% ± 0.04%) in the presence of low non-activating [Ca^2+^]_*cis*_, (SHAM, *P*_o_ = 0.07% ± 0.002%, *P* < 0.05). Hippocampal RyR2 from the MI mice also exhibited increased open time and decreased close time (Fig. 3d,e). This elevated *P*_o_ is consistent with pathological ER Ca^2+^ leak^26,31^. Indeed, neuronal microsomes from MI mice exhibited increased RyR-mediated ER Ca^2+^ leak compared with SHAM-operated mice (Fig. 3f,g). Interestingly, PTMs and functional RyR2 remodeling (leak) were comparable to the RyR2 abnormalities observed in human HF hippocampal samples (Fig. 1). S107 treatment (but not ARM036 treatment) restored calstabin2 binding to RyR2 and decreased the hippocampal RyR2 open probability (*P*_o _= 0.01% ± 0.003%, *P* < 0.05) and mean open time, and increased mean closed time, and reduced ER Ca^2+^ leak (Fig. 3e–g) indicating that the ability of S107 to cross the BBB was essential to fix the leaky hippocampal RyR2 channels. Propranolol treatment significantly reduced RyR2 phosphorylation, nitrosylation and restored calstabin2 binding to the channels. Meanwhile SD-208 treatment significantly decreased RyR2 phosphorylation, oxidation/nitrosylation and enabled rebinding of calstabin2 to the channels (Fig. 3b,c). Both propranolol and SD-208 treatment decreased RyR2 open probability (*P*_o _= 0.018% ± 0.020% and 0.030% ± 0.005% respectively) and mean open time, and increased mean closed time and reduced the ER Ca^2+^ leak compared to the untreated MI mice (Fig. 3e–g). As mentioned above, S107 is a specific RyR2-targeted drug that stabilizes the closed conformation of the channels without effects on PTMs, whereas propranolol and SD-208 prevent RyR2 Ca^2+^ leak by reducing the channel oxidation, nitrosylation and hyper-phosphorylation. Thus, a reduction of RyR2 PTMs and/or stabilization of specific interacting domains of the channels allow calstabin2 to rebind and rescue aberrant RyR2 opening and pathological ER Ca^2+^ leak.

### Impaired long-term potentiation and hippocampal glucose metabolism in myocardial infarction mice

**Fig. 4:**
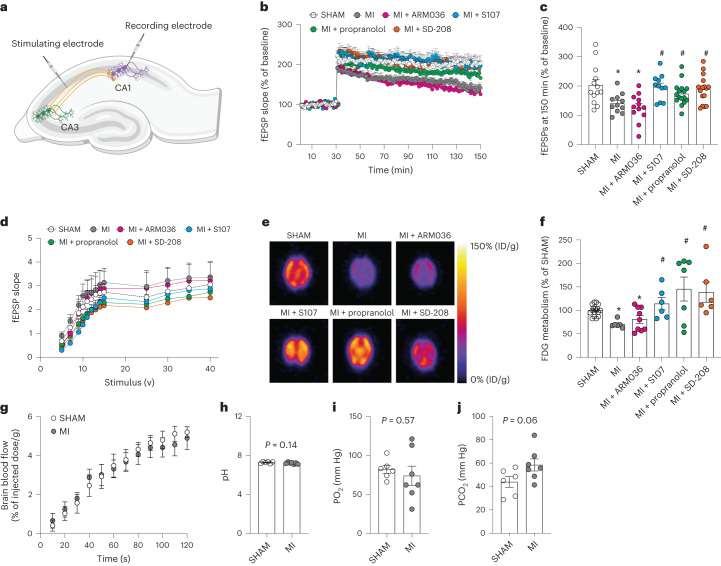
Mouse model of heart failure exhibits impaired long-term potentiation and diminished hippocampal glucose uptake. **a**, Schematic representation of a hippocampal brain slice for LTP experiments and the positioning of the stimulating and recording electrodes. **b**, fEPSPs in hippocampal slices from each experimental group (SHAM (*n* = 13), MI (*n* = 12), MI + ARM036 (*n* = 12), MI + S107 (*n* = 11), MI + propranolol (*n* = 17) and MI + SD-208 (*n* = 16)). **c**, fEPSPs at 150 min in all the experimental groups. **d**, Basal neurotransmission (fEPSP slope), which remained unaltered between the different groups. **e**, Representative microPET images of FDG uptake (percentage of injected dose per gram (%ID/g)) in the mouse brains of different groups. **f**, Quantification of FDG uptake in the brains of mice from different experimental groups shown as a percentage of the FDG uptake in the SHAM mice (SHAM (*n* = 17), MI (*n* = 6), MI + ARM036 (*n* = 9), MI + S107 (*n* = 6), MI+ propranolol (*n* = 7) and MI + SD-208 (*n* = 6)). **g**, Quantification of 2-min dynamic microPET scans of MI (*n* = 4) and SHAM (*n* = 4) mice demonstrating similar brain blood flow FDG uptake in the brains of both groups of mice during the first 2 min after intravenous injection (%ID/g). **h**–**j**, pH, PO_2_ and PCO_2_ blood levels in SHAM (*n* = 6) and MI (*n* = 7) mice. Individual values are shown with the mean ± s.e.m. One-way ANOVA and Tukey’s test post hoc correction for multiple comparisons, *P < 0.05, SHAM versus MI or MI + ARM036; ^#^*P* < 0.05, MI versus MI + S107, MI + propranolol or MI + SD-208. A *t*-test was used in **h**–**j**. Data are derived from biologically independent samples. All statistical tests were two sided. Source data

Field excitatory postsynaptic potentials (fEPSPs) were evaluated at the Schaffer collateral using a bipolar electrode placed at the CA3 and recording at the CA1 (Fig. 4a). Hippocampal slices obtained from MI mice showed decreased LTP as compared to slices obtained from SHAM animals (*P* = 0.01). S107 treatment reversed this effect (*P* = 0.001), with slices eliciting the same LTP response as SHAM, while treatment with ARM036 had no effect on hippocampal synaptic plasticity, resulting in impaired LTP in comparison to SHAM animals (*P* = 0.01). In addition, treatments with propranolol (*P* = 0.01) and SD-208 (*P* = 0.005) prevented MI-induced LTP deficits (Fig. 4b, c). Importantly, MI and treatment with the various compounds after MI had no effect on basal synaptic transmission determined by the input–output curves (I–O; *P* = 0.5; Fig. 4d).

Thereafter, we used [^18^F]fluorodeoxyglucose (FDG) with positron emission tomography (PET) to measure energy consumption in neurons, which reflects neuronal communication signals and the integrative local neuronal activity^52^. Deteriorating brain glucose metabolism measured by FDG-PET has been used as a clinical marker for Alzheimer’s disease diagnosis^52^. Thus, we evaluated hippocampal FDG metabolism in our experimental groups (Fig. 4e,f). Interestingly, hippocampal FDG metabolism was significantly reduced by ~30% in the MI-treated (70 ± 3, *P* < 0.05) and MI + ARM036-treated (80 ± 8, *P* < 0.05) groups compared to SHAM (100 ± 3). The BBB-permeant drug S107, propranolol and SD-208 significantly improved the hippocampal FDG metabolism (114 ± 13, 145 ± 26 and 138 ± 22, respectively, *P* < 0.05; Fig. 4e,f). Importantly, the reduced FDG metabolism in the hippocampi of MI mice was not the result of changes in brain blood flow, which were not reduced in HF mice as measured by a dynamic microPET scan of MI and SHAM mice, nor were there any significant changes in the blood gases including pH, O_2_ or CO_2_ levels, at 2 months after MI (Fig. 4g–j). Both decreased LTP and FDG metabolism are likely due to impaired neurotransmission.

### RyR2-p.Ser2808Ala mice are protected against cognitive dysfunction

To further evaluate the specific contribution of RyR2 to CD in HF, we used a mouse model with RyR2 phospho-mimetic PKA phosphorylation on Ser2808 causing RyR2 Ca^2+^ leak (RyR2-p.Ser2808Asp)^26,31,53^ and compared it to a non-leaky RyR2 mouse model that is protected against RyR2 Ca^2+^ leak due to the genetic ablation of the RyR2 PKA-phosphorylation site Ser2808 (RyR2-p.Ser2808Ala)^26,31,54^ (see cardiac function in Supplementary Table 5).

**Extended Data Fig. 1:**
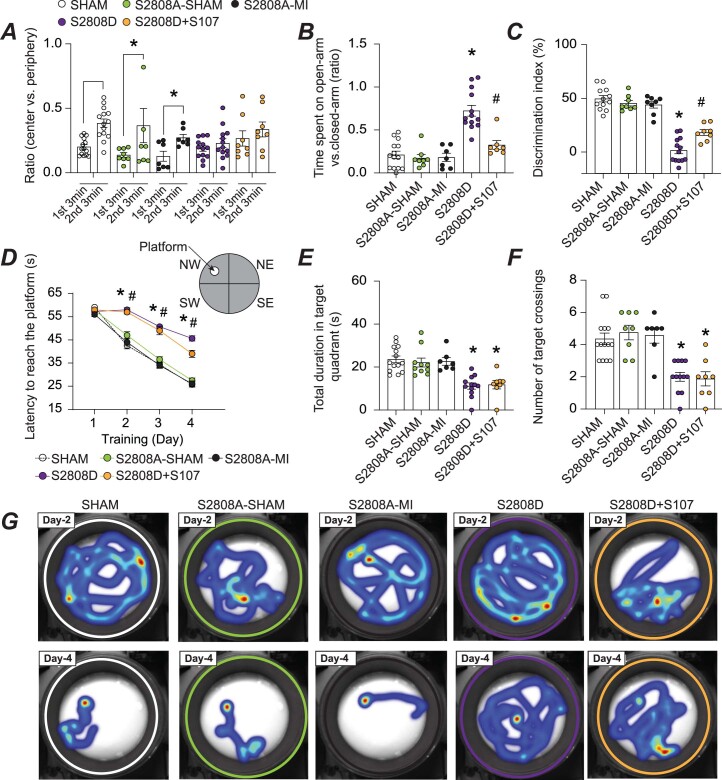
Mouse model of leaky RyR2 (constitutive RyR2 PKA-phosphorylation) is associated with cognitive dysfunction. Mouse model of leaky RyR2 (phospho-mimetic mutation) is associated with cognitive dysfunction. **a**) Open field test of SHAM (n = 14), S2808A-SHAM (n = 8), S2808A-MI (n = 8), S2808D (n = 13), and S2808D + S107 (n = 8) mice. Ratios of total time spent in the center area versus periphery area within first (1st) 3 min and second (2nd) 3 min are shown. **b)** Elevated plus maze test in SHAM (n = 14), S2808A-SHAM (n = 8), S2808A-MI (n = 8), S2808D (n = 13), and S2808D + S107 (n = 8) mice. Ratios of time spent on the open-arm versus closed-arm are shown. **c)** Novel object recognition test in SHAM (n = 14), S2808A-SHAM (n = 8), S2808A-MI (n = 8), S2808D (n = 13), and S2808D + S107 (n = 8) mice. Discrimination index is shown. **d)** Morris water maze test (learning curves) in SHAM (n = 14), S2808A-SHAM (n = 8), S2808A-MI (n = 8), S2808D (n = 13), and S2808D + S107 (n = 8) mice. **e)** Probe trials after escape platform removed in the same groups showing the total duration spent in the target quadrant. **f)** Number of target crossings SHAM (n = 14), S2808A-SHAM (n = 8), S2808A-MI (n = 8), S2808D (n = 13), and S2808D + S107 (n = 8) mice. **g**) Heat maps showing the latency from each group at Day 2 and Day 4. Individual values are shown with mean ± SEM (t-test * p < 0.05 in panel **A** shows significance between the first 3 min and second 3 min of the same groups. One-way ANOVA was used to compare the difference between the 5 groups in panel B, C, E and F; Two-way ANNOVA was used in panel D. Tukey’s test post-hoc correction for multiple comparisons was used; * p < 0.05, S2808A-SHAM vs. S2808D or S2808D + S107; ^#^ p < 0.05, S2808D vs. S2808D + S107. No differences were detected between S2808A-SHAM and S2808A-MI. All statistical tests were two-sided. Data are derived from biologically independent samples. Source data

**Extended Data Fig. 2:**
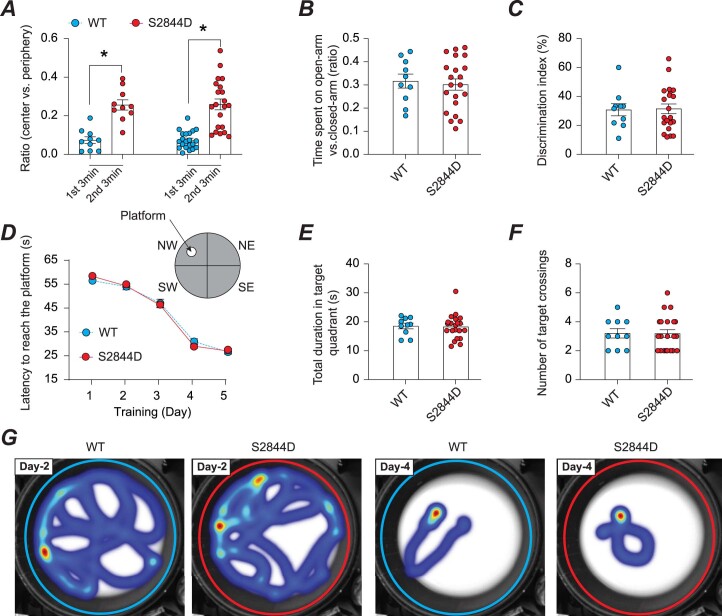
Cognitive function in RyR1-S2844D mice. **a**) Open field test using WT mice (n = 10) and a mouse model with leaky RyR1 channels (S2844D) (n = 21). Ratios of total time spent in the center area versus periphery area within first 3 min and second 3 min are shown. **b)** Elevated plus maze test in WT mice (n = 10) and S2808D (n = 21). Ratios of time spent in the open-arm versus closed-arm are shown. **c)** Novel object recognition test in WT mice (n = 10) and S2808D (n = 21). Discrimination index is shown. **d)** Morris water maze test (learning curves) in WT mice (n = 10) and S2808D (n = 21). **e)** Probe trials after escape platform removed in the same groups showing the total duration spent in the target quadrant in WT mice (n = 10) and S2808D (n = 21). **f)** Number of target crossings in WT mice (n = 10) and S2808D (n = 21). **g**) Heat maps showing the latency from each group at Day 2 and Day 5. Individual values are shown with mean ± SEM. T-test was used in panel A-C, E-F, * p < 0.05 in panel A shows significance between the first 3 min and second 3 min of each group). Two-way ANOVA was used in panel D. Tukey’s test post-hoc correction for multiple comparisons was used. All statistical tests were two-sided. Data are derived from biologically independent samples. Source data

**Extended Data Fig. 3:**
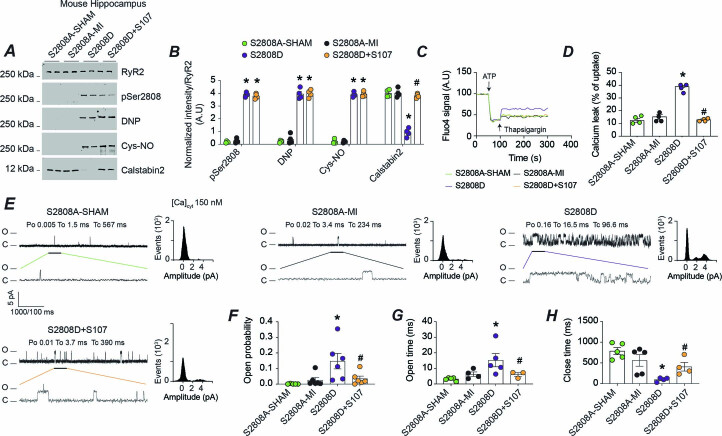
Constitutive RyR2 phosphorylation on Ser2808 (S2808D mice) induces ER Ca^2+^ leak in the hippocampus. Phospho-mimetic mutation (RyR2-S2808D mice) induces ER Ca^2+^ leak in the hippocampus. **a**, **b**) Representative SDS-PAGE analysis and quantification of modified RyR2 and calstabin2 immunoprecipitated from hippocampus of S2808A-SHAM (n = 4), S2808A-MI (n = 4), S2808D (n = 4), S2808D + S107 mice (n = 4) (IP RyR2: Bands normalized to total RyR2); n = 4 in each group. **c**) ER Ca^2+^ leak measured in microsomes from hippocampi of S2808A-SHAM (n = 4), S2808A-MI (n = 4), S2808D, S2808D + S107 mice (n = 4). **d)** Bar graphs represent the quantification of Ca^2+^ leak as the percentage of uptake in all the experimental groups (n = 4 per group). **e)** Single-channel traces of RyR2 incorporated in planar lipid bilayers with 150 nM Ca^2+^ in the *cis* chamber, corresponding to representative experiments performed with hippocampal samples from S2808A-SHAM, S2808A-MI, S2808D, S2808D + S107 mice. **f–h)** RyR2 open probability (Po), mean open time (To), and mean close time (Tc) in S2808A-SHAM, S2808A-MI, S2808D, and S2808D + S107 mice (n = n = 5, 5, 4 and 4 respectively). Individual values are shown with mean ± SEM. One way-ANOVA and Tukey’s test post-hoc correction for multiple comparisons shows * p < 0.05, S2808A-SHAM vs. S2808D or S2808D + S107; # p < 0.05, S2808D vs. S2808D + S107. No differences were detected between S2808A-SHAM and S2808A-MI. All statistical tests were two-sided. Data are derived from biologically independent samples. Source data

RyR2-p.Ser2808Ala-MI mice were protected against impaired locomotive activity and behavioral abnormalities and exhibited better learning and memory compared to RyR2-p.Ser2808Asp mice. S107 treatment improved the short-term memory and reduced the disinhibited behavior of the RyR2-p.Ser2808Asp mice (Extended Data Fig. 1). To ascertain the specific contribution of the RyR2 isoform in this process, we evaluated the CD of genetically altered mice harboring a leaky RyR1 isoform (RyR1-p.Ser2844Asp). We did not detect any cognitive impairment in these mice compared to controls (Extended Data Fig. 2). Moreover, RyR2-p.Ser2808Ala-MI were protected against RyR2 PTM and Ca^2+^ leak (Extended Data Fig. 3).

### RyR2-mediated endoplasmic reticulum Ca^2+^ leak upon adrenergic activation in heart failure

**Fig. 5:**
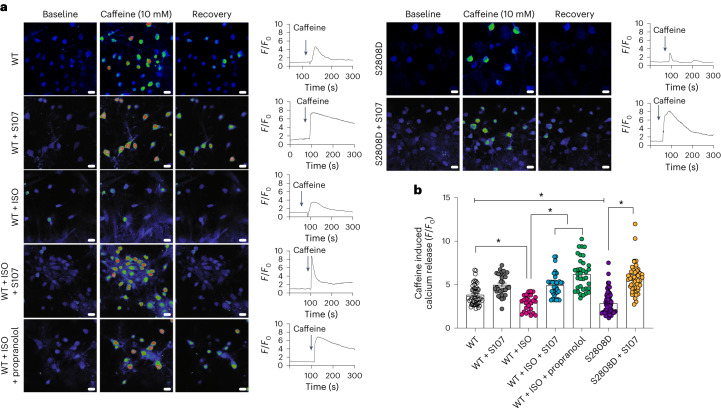
Adrenergic agonist and RyR2 Ser2808 phospho-mimetic mutation deplete endoplasmic reticulum Ca^2+^ stores in primary hippocampal neurons. **a**, Representative images of 14-d cultured hippocampal neurons stimulated with 10 mM caffeine. For each condition, the Ca^2+^ levels are shown at baseline, during stimulation and at recovery. **b**, Quantification of caffeine-induced Ca^2+^release (*F/F*_0_) in response to 10 mM caffeine in neurons from wild-type (WT; *n* = 50), WT + S107 (*n* = 30), WT + isoproterenol (ISO; *n* = 26), WT + ISO + propranolol (*n* = 35), neurons expressing RyR2-p.Ser2808Asp untreated (S2808D; *n* = 70) and treated with S107 (p.Ser2808Asp + S107; *n* = 54). Individual values are shown with the mean ± s.e.m. (*t*-test *P < 0.05). Scale bar, 10 μm. A reduction in caffeine-induced Ca^2+^ release indicates a Ca^2+^-depleted ER due to persistent RyR2-mediated Ca^2+^ leak. Data are derived from biologically independent samples. All statistical tests were two sided. Source data

To determine whether the activation of the adrenergic pathway indeed causes neuronal RyR2-mediated ER Ca^2+^ leak, we used caffeine (10 mM), a well characterized RyR agonist to assess RyR2 function specifically in hippocampal neurons without interference of other cell types such as glial cells or astrocytes. Indeed, caffeine application induced a small Ca^2+^ release in the hippocampal neurons treated with isoproterenol (an adrenergic agonist), which was prevented by either propranolol or S107. Moreover, hippocampal neurons expressing RyR2-p.Ser2808Asp exhibited a reduced RyR2 caffeine-induced Ca^2+^ release compared to controls. Interestingly, S107 treatment restored caffeine-induced RyR2 Ca^2+^ release in neurons expressing RyR2-p.Ser2808Asp mutant (Fig. 5). These findings are in accordance with the beneficial effects of S107 and propranolol on the long-term postsynaptic potentiation, brain glucose metabolism and the cognitive function.

### Gene expression changes and neurodegenerative pathways in heart failure

**Fig. 6:**
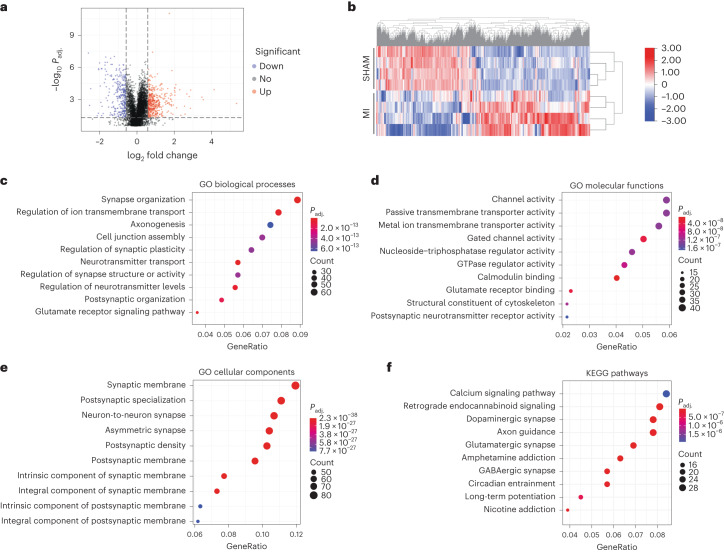
Quantitative proteomics analysis. **a**, Quantitative proteomics was performed on hippocampus samples from SHAM (*n* = 4) and MI (*n* = 4) mice. The Volcano plot shows differentially expressed proteins (*P* adjusted < 0.05, fold change ≥ 1.5) in SHAM and MI mice. Red indicates upregulation, while blue represents downregulation of protein expression. Black indicates unchanged expression levels. **b**, The heat map of significantly dysregulated proteins (312 downregulated; 425 upregulated) The color scale bar shows the row normalized log_2_ protein abundance. **c**–**f**, Dot plots show top ten GO biological processes (**c**), molecular functions (**d**), cellular components (**e**) and KEGG pathways (**f**) that were enriched from differentially expressed proteins. Significantly changed protein abundance was determined by unpaired *t*-test with a threshold for significance of *P* < 0.05 (permutation-based FDR correction), fold change ≥ 1.5, unique peptides ≥ 2. Data are derived from biologically independent samples. All statistical tests were two sided. See Supplementary Table 7 for protein list. Source data

We analyzed the proteome of whole-cell lysates isolated from hippocampi of MI (*n* = 4) and SHAM (*n* = 4) mice. We obtained 6,049 proteins with at least one unique peptide and a 1% false discovery rate (FDR). A volcano plot of all proteins is shown in Fig. 6a. Based on the criteria of adjusted *P* value < 0.05, fold change ≥ 1.5, and unique peptides ≥ 2, we found 737 differentially expressed unique proteins (MI versus SHAM). Among these, 425 proteins were upregulated and 312 were downregulated. The heat map of 737 differentially expressed proteins is shown in Fig. 6b. The MI and SHAM groups were separately clustered, and the four replicates of each group showed good reproducibility. We then performed Gene Ontology (GO) enrichment analyses of these changed proteins. The top ten significant GO terms for biological processes, molecular functions and cellular components are shown in Fig. 6c–e. The biological process GO analysis shows that the differentially expressed proteins were enriched for the following terms: synaptic organization, ion and neurotransmitter transport, synapse activity and plasticity (Fig. 6c). The molecular functions of these dysregulated proteins are mainly related to ion channel activity, transporter activity, GTPase regulator activity, and calmodulin binding (Fig. 6d). Significantly dysregulated proteins were located mainly at the synaptic membrane (Fig. 6e). We also analyzed KEGG (Kyoto Encyclopedia of Genes and Genomes) pathways and found significant enrichment of Ca^2+^ signaling pathways (Fig. 6f). These findings are in accordance with the defective RyR2 Ca^2+^ handling and CD that we observed in MI mice.

**Fig. 7:**
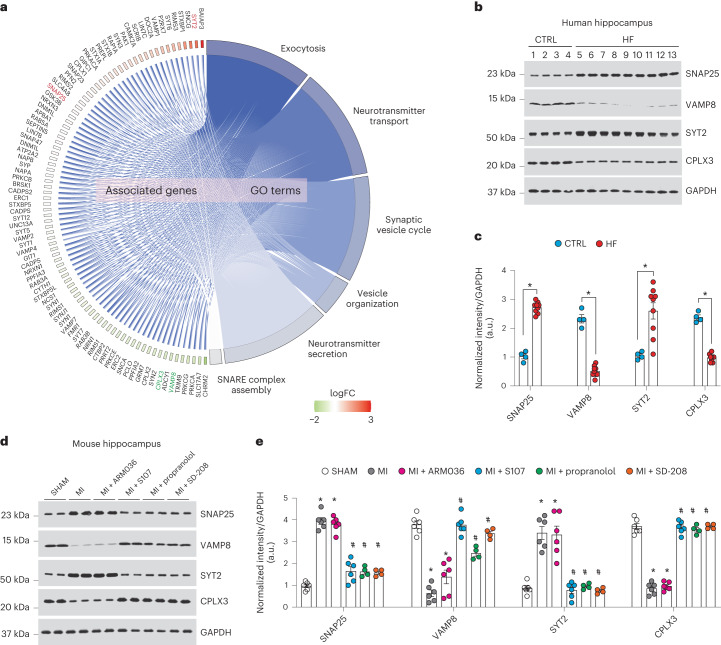
Altered synaptic protein expression in heart failure. **a**, Cohort plot representation of differentially expressed synaptic proteins (SHAM versus MI) from six significantly enriched synaptic transmission GO terms and generated by GOplot. The color map represents fold change of proteins (log_2_ scale). Selected proteins in the SNARE pathway are highlighted in red (upregulated) or green (downregulated). **b**,**c**, Immunoblots showing total expression of SNAP25, VAMP8, SYT2 and CPLX3, normalized to GAPDH in the hippocampi of controls (*n* = 4) and individuals with HF (*n* = 9). Individual values are shown with the mean ± s.e.m. (*t*-test *P < 0.05, control versus individuals with HF). **d**,**e**, Immunoblots showing total expression of SNAP25, VAMP8, SYT2 and CPLX3, normalized to GAPDH in the hippocampi of SHAM (*n* = 6), MI (*n* = 6), MI + ARM036 (*n* = 6), MI + S107 (*n* = 6), MI + propranolol (*n* = 4) and MI + SD-208 (*n* = 4) mice. Individual values are shown with the mean ± s.e.m. One-way ANOVA and Tukey’s test post hoc correction for multiple comparisons, *P < 0.05, SHAM versus MI or MI + ARM036; ^#^*P* < 0.05 MI versus MI + S107, MI + propranolol or MI + SD-208. Data are derived from biologically independent samples. All statistical tests were two sided. Source data

Based on the proteome data in the setting of defective Ca^2+^ regulation, we selected four proteins for further study. These four proteins were from the most enriched synaptic transmission pathways that are regulated by Ca^2+^, located near the synaptic membranes, involved in learning and memory process, and involved in neurotransmission as potential downstream signals of leaky RyR2. These selections included synaptosomal-associated protein 25 (SNAP25), vesicle-associated membrane protein 8 (VAMP8), synaptogamin-2 (SYT2) and complexin3 (CPLX3). We analyzed their protein expression levels by immunoblot in individuals with HF, and in SHAM and MI mice with or without each of the aforementioned treatments. We found decreased VAMP8 and CPLX3 expression in hippocampi of individuals with HF and MI mice compared to SHAM mice, while SNAP25 and SYT2 expression were increased, confirming our proteomic analyses. These data indicate a potential role for the SNARE signaling pathway in HF^55^. S107, propranolol and SD-208 treatment, but not ARM036, restored the expression of these proteins to the control levels (*P* < 0.05, *n* = 4–9 per group; Fig. 7a–e).

Next, we performed GSEA with the canonical pathway and GO gene sets. The top 20 (activated and suppressed) significant GO terms for biological processes, molecular function and cellular components are shown in Extended Data Fig. 5. Interestingly, there was enrichment of GO terms related to mitochondria and neurotransmission as well as oxidative phosphorylation pathways (Extended Data Figs. 5 and 6a), which is potentially due to mitochondrial Ca^2+^ overload (see below), which would stimulate the activity of the tricarboxylic acid cycle and enhance the respiratory chain complex activity by providing more substrates. We also observed significant enrichment of neurodegenerative disease pathways, including those for Alzheimer’s disease, Huntington’s disease and Parkinson’s disease (Extended Data Fig. 6b–d). These GO terms for oxidative phosphorylation defects, Parkinson’s, Alzheimer’s and Huntington’s diseases detected by proteomics were also found at the RNA level (by RNA-sequencing analyses; Extended Data Figs. 7–9).

### Upstream signaling of leaky hippocampal RyR2

We also measured the hippocampal levels of norepinephrine and PKA activity. Hippocampal norepinephrine levels and PKA activity were increased in MI and RyR2-p.Ser2808Asp mice (*P* < 0.05, *n* = 3 in each group; Supplementary Table 5), in line with their respective reduced cardiac function (Supplementary Table 6). Interestingly, RyR2-p.Ser2808Ala-MI mice showed normal norepinephrine and PKA activity comparable to the RyR2-p.Ser2808Ala-SHAM mice. S107 and ARM036 treatments had no effect on brain norepinephrine nor PKA activity compared to untreated MI mice (*P* = 0.9, *n* = 3), because they act directly on RyR channels not on components of the adrenergic pathway.

**Extended Data Fig. 4:**
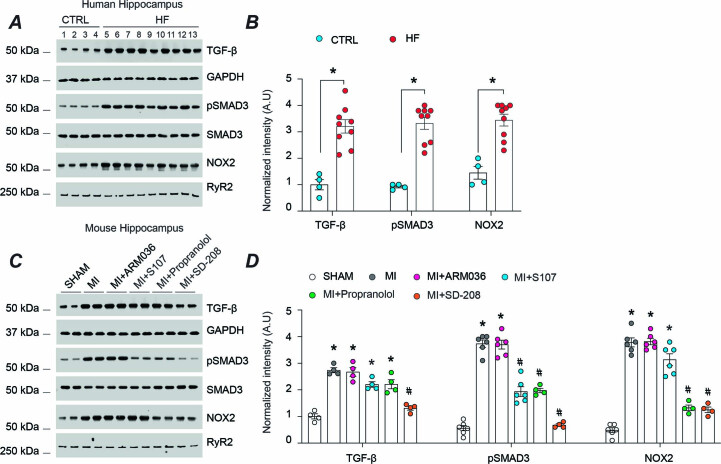
TGF-β activation in HF. **a)** Immunoblots showing expressing levels of TGF-β, phosphorylated SMAD3, total SMAD3, and NOX2 binding to RyR2 in the hippocampi of controls (n = 4) and HF patients (n = 9). **b)** Bar graphs depicting the ratio of TGF-β expression normalized to GAPDH, phosphorylated SMAD3 to total SMAD3 and NOX2 binding to RyR2 (IP RyR2). The same quantity of proteins were loaded on two separate gels and blotted separately for SMAD3 and pSMAD3. Individual values are shown with mean ± SEM (t-test * p < 0.05, Controls vs. HF patients). **c)** Immunoblots showing expressing levels of TGF-β, phosphorylated SMAD3, total SMAD3, and NOX2 binding to RyR2 in the hippocampi of SHAM, MI, MI + ARM036, MI + S107, MI+ propranolol and MI + SD-208 mice (n = 6, 6, 6, 6, 4 and 4 respectively). **d)** Bar graphs depicting the ratio of TGF-β expression normalized to GAPDH, phosphorylated SMAD3 to total SMAD3 and NOX2 binding to RyR2 (IP RyR2). The same quantity of proteins were loaded on two separate gels and blotted separately for SMAD3 and pSMAD3. Individual values are shown with mean ± SEM. One-way ANOVA and Tukey’s test post-hoc correction for multiple comparisons shows * p < 0.05, SHAM vs. MI, MI + ARM036 or MI + S107; ^#^p < 0.05, MI vs. MI + S107, MI+ propranolol or MI + SD-208. All statistical tests were two-sided. Data are derived from biologically independent samples. Source data

TGF-β has been implicated in CNS disorders including Alzheimer’s disease^56,57^. In accordance with our proteomic data, TGF-β levels and phosphorylated SMAD3 (a downstream signal of TGF-β) were increased in hippocampal tissues of individuals with HF and MI mice (*P* < 0.05). Furthermore, NADPH oxidase 2 (NOX2) binding to hippocampal RyR2 was increased in both HF individuals and MI mice, which may account for the oxidation of RyR2 channels and ER Ca^2+^ leak in accordance with previous findings^58–60^ (Extended Data Fig. 4). TGF-β levels, phosphorylation of SMAD3 and NOX2 binding to hippocampal RyR2 were significantly reduced by propranolol and SD-208 treatment in line with the reduced RyR2 oxidation and phosphorylation levels shown in Fig. 3b,c. S107 only diminished the SMAD3 phosphorylation, whereas ARM036 had no effect on any of these changes (Extended Data Fig. 4).

### Mitochondrial Ca^2+^ and oxidative overload in heart failure

We measured the mitochondrial Ca^2+^ content and reactive oxygen species (ROS) production and evaluated the expression and PTMs (phosphorylation) of Ca^2+^-activated enzymes that have been shown to be involved in neurodegenerative diseases such as Alzheimer’s disease.

**Extended Data Fig. 5:**
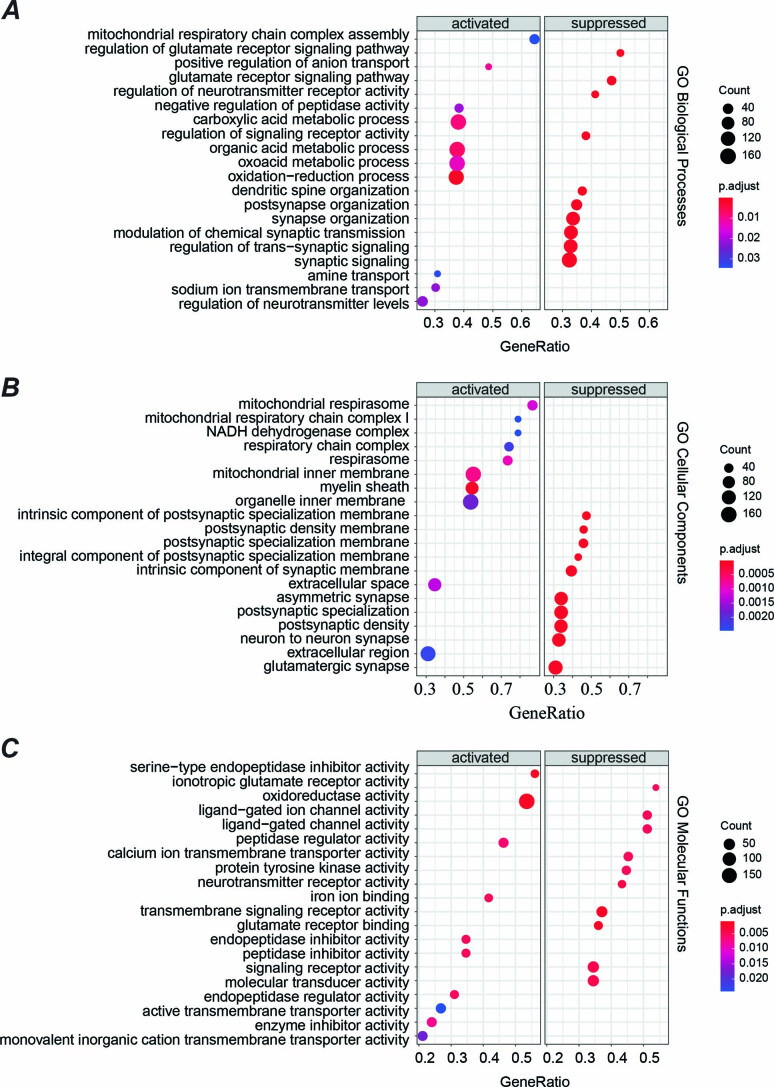
Pre-ranked gene set enrichment analysis (GSEA) of the hippocampal proteomics. Dot plots show: **a)** Top 20 up- and top 20 down-regulated GO biological process, **b)** top 10 up- and top 20 down-regulated GO cellular component, **c)** top 10 up- and top 20 down-regulated GO molecular function terms. Significantly changed protein abundance was determined by unpaired t-test with a threshold for significance of p < 0.05 (permutation-based FDR correction), fold-change ≥1.5, unique peptides ≥2. Data are derived from biologically independent samples. All statistical tests were two-sided*.Source file PRIDE* #PXD042295.

**Extended Data Fig. 6:**
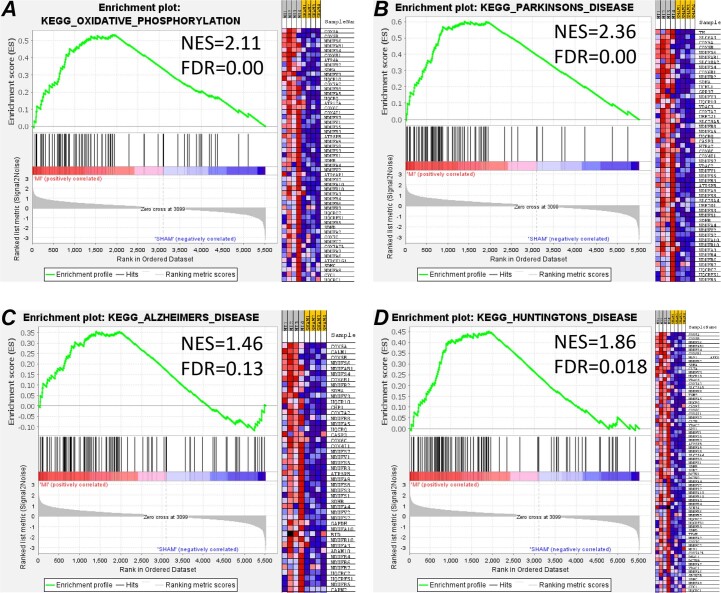
Gene set enrichment analysis (GSEA) of the hippocampal proteomics. The enrichment plots of representative KEGG pathway gene sets demonstrate that oxidative phosphorylation **(a)**, Parkinson’s disease **(b)**, Alzheimer’s disease **(c)**, and Huntington’s disease **(d)** are significantly enriched in MI compared to SHAM. The heatmap on the right side of each panel visualizes the genes contributing to the enriched pathways. For the detailed list *see* Supplementary Table 8. Signal-to-noise ratio was used to rank the genes per their correlation with either MI phenotype (red) or SHAM phenotype (blue). The y-axis represents enrichment score (ES) and on the x-axis are genes (vertical black lines) represented in gene sets. The GSEA analysis calculates an enrichment score (the maximum deviation from zero) reflecting the degree of over-representation of a gene set at the top or the bottom of the ranked gene list. A positive ES indicates gene set enrichment at the top of the ranked list; a negative ES indicates gene set enrichment at the bottom of the ranked list. NES, normalized enrichment score; FDR, FDR adjusted p-value.

**Extended Data Fig. 7:**
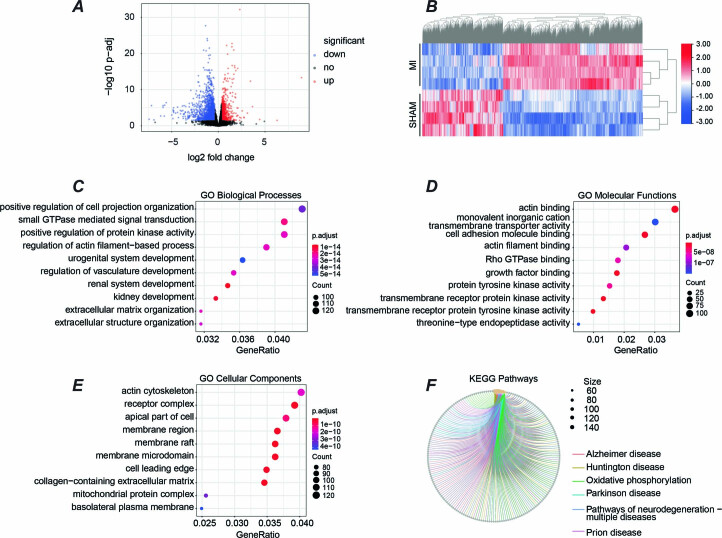
RNA sequencing analysis. RNA-sequencing was performed on the hippocampi of SHAM and MI mice (n = 4 for each group). **a)** The Volcano plot shows differentially expressed genes (p-adj<0.05, fold-change ≥1.3) in SHAM and MI mice. Red indicates up-regulated, while blue represents down-regulated genes. Black indicates unchanged expression levels. **b)** The heat map shows significantly dysregulated genes (down-regulated: 2003, up-regulated: 1149 genes), the color scale bar shows the row normalized log2 protein abundance. **c)** Dot plots show top 10 GO biological processes, **d)** molecular functions, **e)** cellular components, and **f)** KEGG pathways that were enriched from differentially expressed genes. Significantly changed gene abundance was determined by unpaired t-test with a threshold for significance of p < 0.05 (permutation-based FDR correction), fold-change ≥1.5. Data are derived from biologically independent samples. All statistical tests were two-sided. *See* Supplementary Table 9*for gene list. Data are accessible on SRA- Accession:* PRJNA956662.

**Extended Data Fig. 8:**
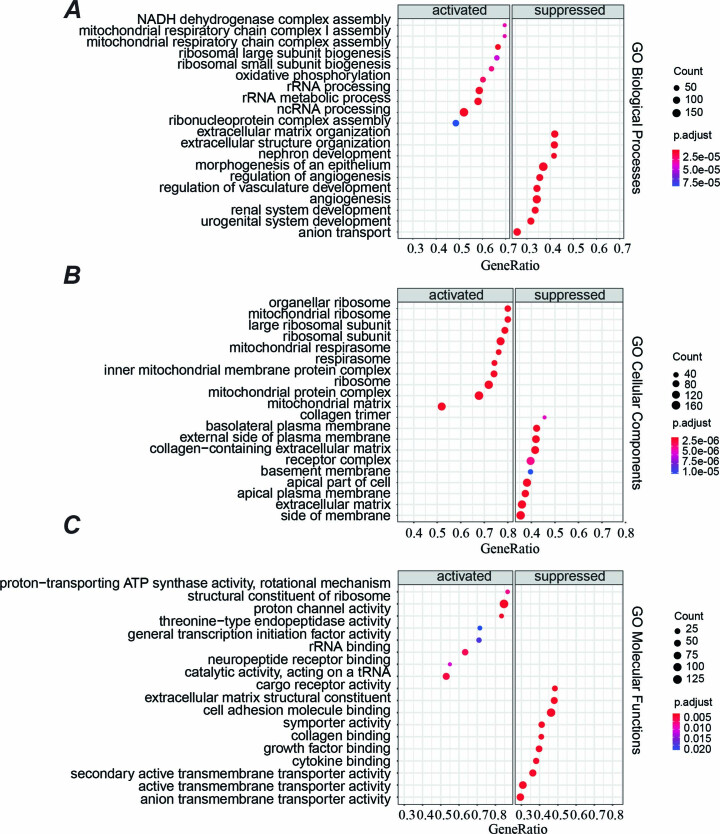
Pre-ranked gene set enrichment analysis (GSEA) of RNA sequencing. Dot plots show: **a)** Top 20 up- and top 20 down-regulated GO biological process, **b)** top 20 up- and top 20 down-regulated GO cellular component, **c)** top 20 up- and top 20 down-regulated GO molecular function terms. Significantly changed gene abundance was determined by unpaired t-test with a threshold for significance of p < 0.05 (permutation-based FDR correction), fold-change ≥1.5. Data are derived from biologically independent samples. All statistical tests were two-sided.

**Extended Data Fig. 9:**
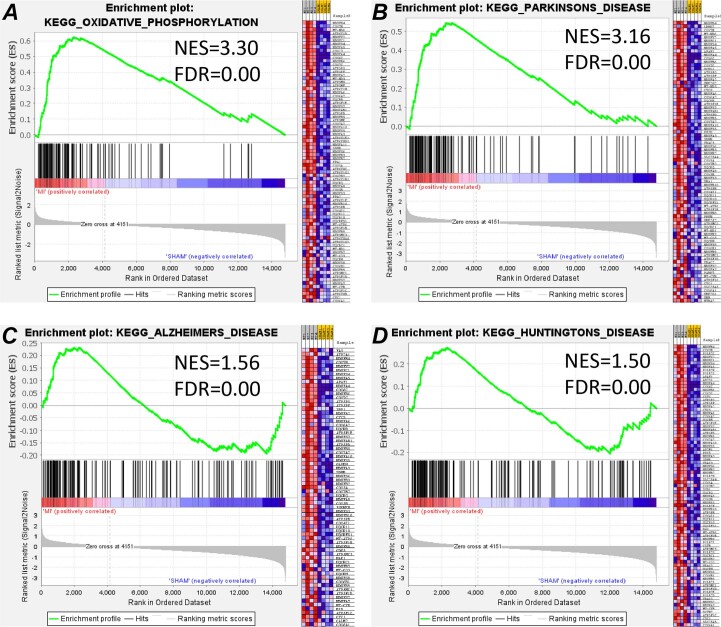
Gene set enrichment analysis (GSEA) of the hippocampal RNA sequencing. The enrichment plots of representative KEGG pathway gene sets demonstrate that oxidative phosphorylation **(a)**, Parkinson’s disease **(b)**, Alzheimer’s disease **(c)**, and Huntington’s disease **(d)** are significantly enriched in MI compared to SHAM. The heatmap on the right side of each panel visualizes the genes contributing to the enriched pathways. For the detailed list, *see* Supplementary Table 10. Signal-to-Noise ratio was used to rank the genes per their correlation with either MI phenotype (red) or SHAM phenotype (blue). The y-axis represents enrichment score (ES) and on the x-axis are genes (vertical black lines) represented in gene sets. The GSEA analysis calculates an enrichment score (the maximum deviation from zero) reflecting the degree of over-representation of a gene set at the top or the bottom of the ranked gene list. A positive ES indicates gene set enrichment at the top of the ranked list; a negative ES indicates gene set enrichment at the bottom of the ranked list. NES, normalized enrichment score; FDR, FDR adjusted p-value.

**Extended Data Fig. 10:**
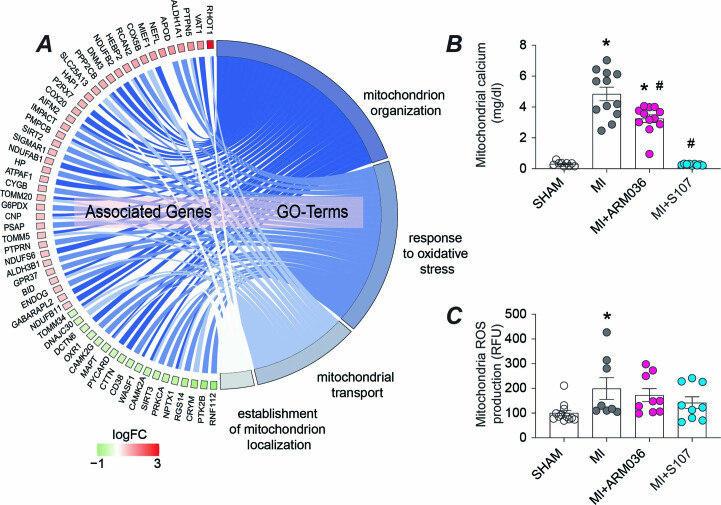
Mitochondrial Ca^2+^ overload and oxidative stress in HF. **a)** Cohort plot representation of differentially expressed mitochondrial proteins (SHAM vs MI) from 4 significantly enriched mitochondrial GO-terms and generated by GOplot. The color map represents fold change of proteins (log_2_ scale). **b**) Ca^2+^ accumulation in isolated mitochondria from SHAM (n = 6), MI (n = 5), MI + ARM036 (n = 5), and MI + S107 (n = 5) mice. **C)** Reactive oxygen species (ROS) production in isolated mitochondria from SHAM (n = 6), MI (n = 6), MI + ARM036 (n = 6), and MI + S107 (n = 5) mice. Individual values are shown with mean ± SEM (one-way ANOVA and Tukey’s test post-hoc correction for multiple comparisons show * p < 0.05, SHAM vs. MI or MI + ARM036; ^#^p < 0.05, MI vs. MI + S107). All statistical tests were two-sided. Source data

Our cohort plot of differentially expressed mitochondrial proteins shows an increase of the mitochondrial Rho GTPase 1 (RHOT1) in the MI mice (Extended Data Fig. 10a), which is a mitochondrial GTPase involved in mitochondrial fission during high Ca^2+^ conditions^61^. In line with the increased RyR2 Ca^2+^ leak, there was increased mitochondrial Ca^2+^ accumulation in MI mice (*P* < 0.05, *n* = 12). Interestingly, mitochondrial Ca^2+^ levels were significantly reduced by S107 treatment (*P* < 0.05, *n* = 9), indicating that leaky RyR2 channels are an upstream event of the mitochondrial Ca^2+^ overload (Extended Data Fig. 10b). Mitochondrial ROS production was significantly increased in the MI mice (*n* = 9) compared to the SHAM-operated group (*n* = 12, *P* < 0.05; Extended Data Fig. 10c). Although the mitochondrial ROS production was attenuated by S107, it did not reach statistical significance (*P* = 0.2), despite the reduction of the mitochondrial Ca^2+^ content. This is potentially due to irreversible damage that targets the electron transport chain that subsequently has led to increased electron transport chain protein expression levels observed in our proteomic analysis as a compensatory mechanism (Extended Data Figs. 5 and 6).

### Alzheimer’s disease-like signaling in heart failure

Abnormal Ca^2+^ regulation can contribute to the activation of Ca^2+^-dependent enzymes such as AMP-activated protein kinase (AMPK), cyclin-dependent kinase 5 (CDK5) and enhanced calpain activity^26,62^. Activation of these enzymes in response to elevated cytosolic Ca^2+^ levels are upstream signals of both pTau and amyloid deposits in Alzheimer’s disease brains^26,63^ and could be a major factor in the CD observed in HF. Hippocampal samples from both HF individuals and MI mice showed increased AMPK and GSK3β phosphorylation (on Thr216) and CaMKII activity (Supplementary Table 6), compared to controls (*P* < 0.05). Interestingly, phosphorylation levels of Tau on Ser199/202/262 and Thr205 were significantly increased in both HF individuals and MI mice (*P* < 0.05). We also observed an increase in p25 expression, the neurotoxic activator of CDK5, which plays an important role in amyloid precursor protein processing in AD. Subsequently, the amyloid beta pathway may be activated, as BACE1 and βCTF levels were significantly increased in individuals with HF and MI mice (Supplementary Figs. 1 and 2). Finally, all of these changes were prevented/attenuated by treatment with S107, propranolol or SD-208 (*P* < 0.05, *n* = 4), but not by ARM036 (Supplementary Fig. 2).

## Discussion

The major findings of this study are: (1) hippocampal neurons in HF have leaky RyR2, which correlates with behavioral abnormalities; (2) RyR2 channels are leaky due to stress-induced phosphorylation, oxidation, nitrosylation and depletion of the stabilizing subunit calstabin2; (3) hyper-adrenergic signaling and activation of TGF-β signaling are upstream signals of leaky RyR2; (4) excessive RyR2 Ca^2+^ leak is associated with mitochondrial dysfunction, impaired synaptic transmission and increased Tau pathway activation similar to that observed in Alzheimer’s disease; (5) the Rycal drug (S107) prevents loss of the RyR2 stabilizing subunit calstabin2 and rescues the aforementioned subcellular events. Of note, we recently reported the binding site for the Rycal S107 (ref. ^51^) in RyR1 and ARM210 (ref. ^50^) in RyR2 using cryogenic electron microscopy, and have gained insights into its mechanism of action on leaky channels. Rycal drugs bind to a cleft in the RY1&2 domain of the channel where they stabilize interactions of key residues and reduce the flexibility between domains of the cytoplasmic shell, bringing the overall channel conformation from a primed state that is easily activated closer to the closed state thus preventing aberrant ER Ca^2+^ leak^50^.

In human and experimental models of HF, increased activity of the sympathetic nervous system^64^ results in increased systemic catecholamine levels^65^, that is, norepinephrine, an activator ligand of the adrenergic pathway. The improvement of the cognitive function and neuronal activity in the propranolol-treated MI mice, clearly shows that adrenergic signaling is an important component of cardiogenic dementia. Of note, this neurohormonal dysregulation that affects Ca^2+^ homeostasis is not limited to the heart and the brain of HF patients, but also impacts other organs, including skeletal muscle, and accounts for chronic fatigue, reduced exercise capacity and respiratory muscle weakness. Indeed, we have previously reported RyR1 remodeling in skeletal muscle of HF individuals, which impairs their exercise tolerance^42^.

We observed elevated brain norepinephrine levels and increased PKA activity associated with RyR2 Ca^2+^ leak in mice with failing hearts. This biochemical signature and dysfunction of RyR2 were correlated with behavioral abnormalities.

TGF-β, an upstream mediator of RyR2 oxidation via NOX2 enzymes^58–60^, is also increased, thereby accounting for further oxidation of RyR channels and increased SR/ER Ca^2+^ leak in HF^66,67^. In line with previously published studies^68^, TGF-β inhibitor SD-208 significantly reduced RyR2 oxidation, rebound calstabin2 to the channel and decreased ER Ca^2+^ leak in the MI mice. Moreover, SD-208 improved LTP, brain glucose metabolism and the overall cognitive function of MI mice. Although the TGF-β family inhibitors have shown relative efficacy as an anticancer treatment, the SD-208 compound has rarely been tested for its effects on cognition. For instance, SD-208 was able to inhibit germinal matrix hemorrhage in a rat model of the disease with a partial recovery of the motor and cognitive function^69^. SD-208 treatment improved the spatial learning in a rat model of HIV-1-associated neurocognitive disorders^70^. Of note, this TGF-β inhibitor was used in our study to dissect the cellular mechanisms of cardiogenic dementia, but its potential as a drug candidate remains uncertain because of its cardiac toxicity and the side effects of blocking the physiological TGF-β signaling in healthy cells^71^. Our results indicate a beneficial effect of propranolol on cognition and memory in the context of cardiogenic dementia, in line with previously published studies showing improvement of cognitive function with beta-blocker therapy in different contexts such as hypertensive older individuals and patients Alzheimer’s disease^72,73^. Interestingly, propranolol displayed beneficial effects on cognition, especially on sustaining cognitive performance over time in healthy individuals^74^, and reduced amyloid and Tau pathology in Alzheimer’s transgenic mice^75^. Other studies have reported deleterious effects of beta blockers on cognitive function in patients after MI and in the general population^76,77^. Because propranolol is extensively used to treat HF patients, a randomized clinical trial is necessary to resolve this controversy.

Mitochondrial Ca^2+^ accumulation, a downstream effect of TGF-β and leaky RyR2 channels was enhanced in MI mice, with significant upregulation of mitochondrial ROS production. These changes trigger a vicious cycle in which RyR2 leak leads to mitochondrial Ca^2+^ overload, which then produces ROS and further oxidizes RyR2 channels^46^. Such a vicious cycle of Ca^2+^/ROS alteration could lead to gene and/or protein expression abnormalities that would worsen the prognosis of HF patients over time. The deep changes in the transcriptome and the proteome in the MI mice along with the epigenetic alterations we previously reported in the RyR2-p.Ser2808Asp mice serve as preliminary evidence of this hypothesis^31^.

Logically, oxidative stress and Ca^2+^ dysregulation would initiate a cellular response that would manifest at the level of neuronal gene expression. Support for this view stems from our observation of upregulation of 425 and downregulation of 312 proteins and genes in hippocampi from MI mice. Interestingly, the expression of key proteins regulated by Ca^2+^ and involved in synaptic transmission were modified including CPLX3, as well as SNAP25, SYT2 and VAMP8, which may explain, in part, the CD observed in our MI and RyR2-p.Ser2808Asp mice. For example, Ca^2+^ triggers rapid exocytosis of neurotransmitters from neurons. Such a process is mediated by synaptogamins, an abundant component of synaptic vesicles that binds Ca^2+^ ions through two C2 domains^78^. Our data revealed a significant increase of the SYT2 isoform and SNAP25 at the protein expression level, which is an indicator of defective Ca^2+^-dependent exocytosis and altered hippocampal synaptic transmission in HF in line with the decreased LTP and brain glucose metabolism in the MI mice.

Furthermore, the changes in protein expression parallel some of the changes observed in models of neurodegenerative diseases. Postmortem studies of Alzheimer’s disease brains have shown altered levels of several synaptic proteins, including SNAP25 and synaptogamins, components of the SNARE complex^79,80^. Furthermore, cerebrospinal fluid (CSF) levels of SNAP25 (ref. ^81^) and synaptogamins^82^ have been assessed and found to be elevated in patients with Alzheimer’s disease or middle cognitive impairment, compared to controls. To our knowledge, this is the first study reporting alterations in the hippocampal synaptic proteins and cognitive impairment in HF and providing a unique transcriptome and proteome library for the future mechanistic investigations and supported by in vivo and in vitro cognitive testing. This is particularly important for the initiation of large-scale clinical studies to assess some of these markers in the CSF or the plasma of HF patients as a predictive molecular fingerprint to monitor the onset and the progression of cognitive impairment in these patients. Finally, we report impaired hippocampal glucose metabolism in HF mice using [^18^F]FDG-PET scan imaging. This is a valuable neuroimaging tool for early detection of cardiogenic dementia and could be added as a clinical biomarker to support assessment and management of HF patients. Further studies to confirm its efficacy in early detection of CD are needed.

Taken together, our data are in line with previous reports^26,27,31,83^ suggesting that defective Ca^2+^ plays an instrumental role in neurodegeneration and cognitive impairment. Further analyses of protein expression levels and PTMs showed significant upregulation of Ca^2+^-dependent enzymes involved in Tau processing and Alzheimer’s disease. These markers were found to be increased in a small cohort of individuals with coronavirus disease 2019 suspected of developing a forme fruste of Alzheimer’s disease^83^ due to defective Ca^2+^ regulation and inflammation. HF patients and MI mice exhibited increased TGF-β and SMAD3 phosphorylation levels that potentially play a role in cardiogenic dementia.

**Fig. 8:**
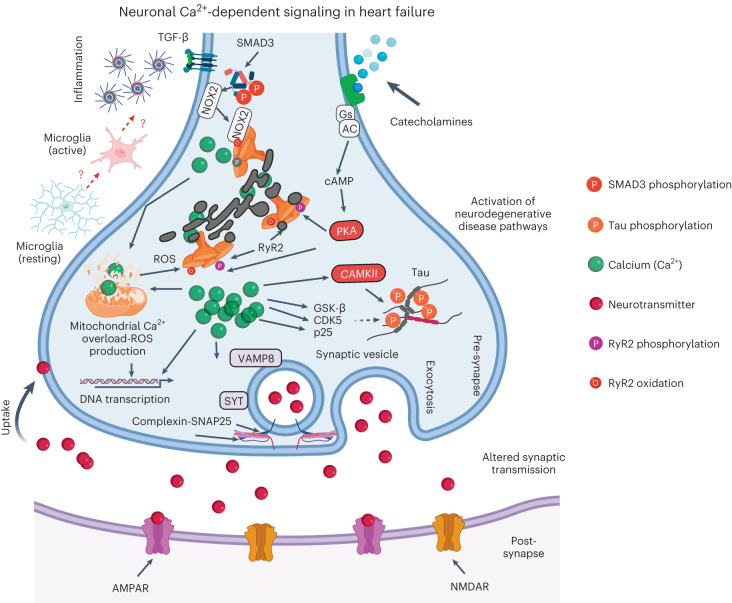
Neuronal Ca^2+^ signaling in heart failure. Increased catecholamine levels during HF activate PKA, which phosphorylates RyR2 on Ser2808 (Fig. 3). Increased inflammation in HF includes activation of the TGF-β pathway resulting in SMAD3 phosphorylation and upregulation of NOX2 and binding to RyR2 (Extended Data Fig. 4). NOX2 promotes oxidation of RyR2 channels^58–60^. The combination of oxidation and phosphorylation of RyR2 results in ER Ca^2+^ leak (Fig. 3). Ca^2+^ leak through RyR2 leads to increased mitochondrial Ca^2+^ accumulation, which enhances mitochondrial ROS production (Extended Data Fig. 10). Therefore, a vicious cycle is created between the mitochondria and RyR2, where increased ER Ca^2+^ leak causes mitochondrial ROS production and increased mitochondrial ROS production further oxidizes RyR2 and renders it leakier. Chronic RyR2 Ca^2+^ leak depletes ER Ca^2+^ content and reduces the Ca^2+^ transient (Fig. 5) required for synaptic vesicle release during synaptic transmission (Figs. 4 and 7). Furthermore, oxidative stress and Ca^2+^ dyshomeostasis alter gene transcription (Extended Data Fig. 7), with a particular effect on proteins that are regulated by Ca^2+^ and involved in neurotransmission. Dysregulation of key proteins involved in synaptic transmission is reflected in the impaired LTP observed in the MI mice (Fig. 4b,c). Accumulation of Ca^2+^ in the cytosol activates Ca^2+^-dependent enzymes including CAMKII, GSK-β, CDK5 and p25, which subsequently leads to Tau phosphorylation, a hallmark of neurodegenerative disease (Supplementary Figs. 9 and 10). All these activated signaling cascades can be prevented, at least in part, by S107, a Rycal drug that reduces the ER Ca^2+^ leak. Gs, G protein; AC, adenylyl cyclase; cAMP, cyclic AMP; GSK-β, glycogen synthase kinase 3 beta. Created with BioRender.com.

Increased adrenergic activity and inflammatory pathway activation in HF primarily impairs intracellular Ca^2+^ regulation. Excessive ER Ca^2+^ leak enhances oxidative stress, dysregulates neuronal gene/protein expression and primes neurodegenerative pathways. These subcellular changes impair the learning and memory processes in HF, which is detrimental for patients’ compliance to medication and early recognition of worsening symptoms. These pathways are summarized in Fig. 8.

Limitations of our study include a small sample size of only nine individuals with HF who had incomplete clinical information. We used multiple mouse models to overcome these limitations. Moreover, all individuals with HF were younger than controls, minimizing age-related confounding factors. Other factors such as individuals’ backgrounds, socioeconomic status, hospitalization history and drug use could, however, contribute to CD.

## Methods

### Human samples

De-identified human hippocampus and cortex samples were obtained from the Brain Bank at Columbia University and the NIH Neuro-Biobank. The control hippocampal autopsy samples exhibited absence of neurological disorders and plaques, and previous experiments using these specific control samples had shown a lack of remodeling and leak in RyR2 (ref. ^31^). Information on the individuals with HF and controls is listed in Supplementary Tables 1 and 2. We obtained an Institutional Review Board exemption for the use of these specimens (AAAU3119).

### Animal models

Six-month-old male C57BL/6 mice (Jackson laboratory), RyR2-p.Ser2808Ala and RyR2-p.Ser2808Asp mice (C57BL/6 background, available in the Marks Laboratory) were maintained and studied according to protocols approved by the Institutional Animal Care and Use Committee of Columbia University (reference no. AC-AAAC5453). Animals were randomly assigned to one of the designed groups. All in vivo animal experiments were performed by investigators blinded to genotype and treatment groups. To induce HF in C57BL/6 and p.Ser2808Ala mice, the proximal left anterior descendent coronary artery was ligated under general anesthesia using isoflurane (1.5%) in orally intubated mice as previously described^84^. The Rycal S107 (BBB permeant) was administered in drinking water at 75 mg per kg body weight per day for 1 month as previously described^31^. To differentiate between CNS effects versus peripheral cardiac effects, the Rycal ARM036 (non-BBB permeant)^27^ was administered in drinking water at 20 mg per kg body weight per day for 1 month. Propranolol (10 mg per kg body weight per day) and SD-208 (10 mg per kg body weight per day) treatments were given by intraperitoneal injection. Standard food was provided ad libitum throughout the experiments.

### Echocardiography

Cardiac function was assessed in anesthetized mice (1–3% isoflurane, 100% oxygen) by transthoracic echocardiography using a high-resolution ultrasound system (Vevo 2100; VisualSonics) equipped with a 40-MHz linear array transducer (MS550, Vevo2100, VisualSonics). A left ventricular parasternal long-axis two-dimensional view in M-mode was performed at the level of papillary muscle to assess left ventricular wall thicknesses and internal diameters, allowing the calculation of the fractional shortening and ejection fraction by the Teicholz method as previously described^53^. HF animal models’ characteristics are shown in Supplementary Tables 3 and 6.

### Behavioral studies

#### Open field

The behavior assays were performed 1 month after the initiation of the cardiac insult and last over several weeks. Briefly, the open field test was processed in a chamber with an area of 50 cm × 50 cm and walls of 38 cm in height^31^. Each mouse was placed at the center of the chamber and allowed to move freely for 6 min. The total time spent in the center area and peripheral area was recorded.

### Elevated plus maze

The levels of disinhibited behavior were evaluated using the EPM test^31,47^. The maze was 40 cm high and contained two 61 cm × 5 cm open arms and two 61 cm × 5 cm × 15 cm closed arms with a center area of 5 cm × 5 cm. Briefly, each mouse was placed in the center area of the apparatus and allowed to move freely for 5 min. The total time spent in the open arm and close arm was recorded.

### Novel object recognition

The short-term recognition memory was evaluated using the novel object recognition test as previously described^31,48,85^. The same open field arena was used for this test. One day after the open field test, each mouse was returned to the open field arena that contained two identical objects and allowed to freely explore for 10 min. After a 1-h interval, each mouse was placed back into the same arena, where one of the objects was replaced with a novel object, for another 5 min. The discrimination index of each mouse to explore the novel object was recorded and calculated.

### Morris water maze

The MWM was used to study spatial learning and memory in MI mice and SHAM mice as controls^31,49^. The experimental apparatus included a water tank that had a diameter of 122 cm and a height of 82 cm, filled with water at 23 ± 2 °C and mixed with nontoxic white paint to make it opaque. The surface of the water pool was vertically divided into four quadrants. A plastic platform with a surface area of 10 cm × 10 cm and height of 55 cm was located in the middle of the northwest quadrant and 1 cm underneath the surface during the experiment. The experiment lasted for 5 d, including 4 d of training trials and 1 d of probe trial on day 5. During daily training trials, each mouse was released from different quadrants alternately in each trial. There were three trials per day and each trial lasted for 60 s. The duration for each mouse to find and sit on the platform for at least 2 s was defined as latency. On day 5 of the experiment, the hidden platform was removed, and each mouse was allowed to free swim for 60 s. The total duration spent in the quadrant with the previously hidden target (target quadrant) was recoded and the number of crossings through that quadrant (target crossing) was also recorded.

All the experiments were recorded by a camera installed over the experimental apparatus and analyzed by Etho Vision XT video tracking software (Noldue Information Technology). Two-way ANOVA analysis was performed for the training trials of MWM, and *t*-test and one-way ANOVA analysis were performed with Tukey’s test post hoc correction for the rest of the tests. Minimum statistically significant differences were established at *P* < 0.05.

### Brain PET/CT imaging

For the brain glucose metabolism assessment, a 10-min static PET scan of brains was performed. After completing the behavioral assays, mice from different experimental groups were injected intravenously with 5.4–8.1 MBq (146–220 μCi) of [^18^F]FDG. Around 2 h after injection, brains were dissected and static 10-min PET images were obtained using an Inveon MicroPET scanner (Siemens).

For the brain blood flow assessment, a 2-min dynamic PET scan of mice was performed. MI and SHAM mice were injected intravenously with 4.0–4.3 MBq (109–117 mCi) of FDG, and 2-min dynamic PET scans were obtained using an Inveon microPET scanner (Siemens)^86–88^. The body temperature of mice was maintained at 37 °C. MicroCT (MILabs) was used for anatomical references. Regions of interest were manually drawn over the hippocampus. All PET images were reconstructed using an 3D-OSEM algorithm with three iterations in a 256 × 256 matrix (Inveon, Siemens) and analyzed using VivoQuant version 4 (Invicro). Decay correction was applied to all PET data.

### Field excitatory postsynaptic potentials evaluation

LTP experiments were performed as previously described^89^. Briefly, transverse hippocampal slices (400 μm) were cut by tissue chopper and transferred to a recording chamber in which the temperature was maintained at 29 °C. During the recovery and recording periods, the slices were constantly perfused with artificial CSF (124.0 mM NaCl, 4.4 mM KCl, 1.0 mM Na_2_HPO_4_, 25.0 mM NaHCO_3_, 2.0 mM CaCl_2_, 2.0 mM MgCl_2_ and 10.0 mM glucose) that was bubbled with 95% O_2_ and 5% CO_2_. fEPSPs were evaluated at the Schaffer collateral by a bipolar electrode placed at the CA3 and recording at the CA1 with an artificial CSF-filled glass pipette. Basal synaptic transmission was evaluated via measurement of the I–O curve via plotting the relationship between increased voltages (5 V to 40 V) and evoked fEPSP responses. A 30-min baseline was recorded every minute at a voltage eliciting 35% of the maximum evoked response as determined by the I–O curve. LTP was elicited by a theta-burst stimulation (four pulses at 100 Hz, with the bursts repeated at 5 Hz and three tetani of ten-burst trains administered at 15-s intervals), and responses were recorded for 2 h. Results were analyzed in pClamp (Molecular Devices) and fEPSP slopes for I–O and LTP traces were compared by two-way ANOVA for repeated measures.

### Hippocampal neurons culture and Ca^2+^ imaging

Hippocampi from WT and RyR2-p.Ser2808Asp pups were dissociated and cultured using Pierce primary neuron isolation kit (88280) according to manufacturer instructions. Briefly, hippocampi were dissected from one postnatal day mice and digested with 0.25% papain at 37 °C for 30 min. Digested cells were resuspended in pre-warmed serum-supplemented neuronal culture medium and plated in collagen/poly-d-lysine-coated 35-mm dishes with coverslip at 37 °C in a 5% CO_2_ incubator. After 24 h, the medium was replaced with an equivalent volume of serum-free neuronal culture medium. Next, 1× neuronal growth supplement was added at day 3 to reduce the non-neuronal cell contamination and maintain neurons at high purity during the culture period. Treated cells were incubated overnight with the following drugs: S107 (10 μM), isoproterenol (1 μM) and propranolol (1 μM). Cells were loaded with 10 μM fluo-4, AM, in culture medium for 30 min at 37 °C and then washed and incubated with Krebs solution (140 mM NaCl, 5 mM KCl, 2 mM CaCl_2_, 1 mM MgCl_2_, 11 mM glucose and 5 mM HEPES, pH 7.4). Imaging experiments were performed at room temperature (RT; 26 °C). Caffeine was prepared in Krebs solution and added to the cells at 10 mM. Time-series confocal imaging was performed by excitation with a 488-nm light from the argon laser of a Zeiss LSM 800 inverted confocal microscope (×40 oil immersion lens). Data were analyzed using ImageJ software.

### Lysate preparation and western blots

Tissues (50 mg) were lysed using a Dounce homogenizer in 0.25 ml of 10 mM Tris maleate (pH 7.0) buffer with protease inhibitors (complete inhibitors from Roche). Samples were centrifuged at 800*g* for 20 min and the protein concentrations of the supernatants were determined by Bradford assay. To analyze protein expression in tissues, tissue lysates (20 μg) were separated by 4–20% SDS–PAGE and immunoblots were developed using antibodies against the indicated proteins (see list of antibodies used in Supplementary Information). Each protein was detected on a separate immunoblot.

### Analyses of ryanodine receptor complex

RyR2 was immunoprecipitated from 0.1 mg of hippocampus lysate using an anti-RyR2-specific antibody (2 μg) in 0.5 ml of a modified radioimmune precipitation assay buffer (50 mm Tris-HCl, pH 7.2, 0.9% NaCl, 5.0 mm NaF, 1.0 mm Na_3_VO_4_, 1% Triton X-100 and protease inhibitors; RIPA) overnight at 4 °C. The immune complexes were incubated with protein A-Sepharose beads (Sigma) at 4 °C for 1 h, and the beads were washed three times with RIPA as previously described^58^. Then, 10 μl of immunoprecipitated volume was loaded on 6% gels to probe total phosphorylated, nitrosylated and total RyR2 or on 4–20% gradient gels to probe calstabin2 and NOX2. Proteins were transferred onto nitrocellulose membranes for 1 h at 200 mA. Immunoblots were developed using the following primary antibodies (see list of antibodies used in Supplementary Information): anti-RyR2, anti-phospho-RyR-Ser(P)-2808, anti-Cys-NO, anti-calstabin and anti-NOX2. The channel’s oxidation was determined by probing the carbonyl groups in the protein side chains by reaction with 2,4-dinitrophenylhydrazine. The DNP signal associated with RyR2 was determined using a specific anti-DNP antibody according to the manufacturer using an Odyssey system (LI-COR Biosciences) with infrared-labeled anti-mouse and anti-rabbit immunoglobulin G (IgG; 1:5,000 dilution) secondary antibodies. RyR2 PTM levels, calstabin2 and NOX2 binding were quantified using image studio (LI-COR Biosciences) and normalized to total immunoprecipitated RyR2 protein.

### Protein kinase A activity assay

PKA activity in hippocampal lysates was determined using a PKA activity kit (Thermo Fisher, EIAPKA). Briefly, samples were added to a microtiter plate containing an immobilized PKA substrate that is phosphorylated by PKA in the presence of ATP. After incubating the samples with ATP at RT for 2 h, the plate was incubated with the phospho-PKA substrate antibody for 60 min. After washing the plate with wash buffer, goat anti-rabbit IgG horseradish peroxidase conjugate was added to each well. The plate was aspirated, washed, and TMB substrate was added to each well, which was then incubated for 30 min at RT. A plate reader was used to determine the absorbance at 450 nm. The sample signals were compared to a standard curve.

### Calmodulin-dependent protein kinase II activity assay

CaMKII activity in hippocampal lysates was determined using the CycLex CaM kinase II Assay Kit (MBL International). Briefly, samples were added to a microtiter plate containing an immobilized CaMKII substrate that is phosphorylated by CaMKII in the presence of Mg^2+^ and ATP. After incubating the samples in kinase buffer containing Mg^2+^ and ATP at RT for 1 h, the plate was washed and incubated with the horseradish peroxidase-conjugated anti-phospho-CaMKII substrate antibody for 60 min. The plate was aspirated, washed, and TMB substrate was added to each well, which was then incubated for 30 min at RT. A plate reader was used to determine the absorbance at 450 nm. The sample signals were compared to a standard curve.

### Endoplasmic reticulum vesicle preparation

Hippocampi were homogenized on ice in 300 mM sucrose, 20 mM PIPES (pH 7.0) in the presence of protease inhibitors (Roche), and centrifuged at 5,900*g* for 20 min at 4 °C. The supernatant was ultracentrifuged at 100,000*g* for 1 h at 4 °C. The final pellet containing microsomal fractions enriched in ER vesicles was resuspended and aliquoted in 300 mM sucrose, 5 mM PIPES (pH 7.0) containing protease inhibitors. Samples were frozen in liquid nitrogen and stored at −80 °C.

### Single-channel data using planar lipid bilayers

Planar lipid bilayers were formed using a 3:1 mixture of phosphatidylethanolamine and phosphatidylcholine (Avanti Polar Lipids) suspended (30 mg ml^−1^) in decane by painting the lipid/decane solution across a 200-µm aperture in a polysulfonate cup (Warner Instruments) separating two chambers. The *trans* chamber (1 ml), representing the intra-ER/SR (luminal) compartment, was connected to the headstage input of a bilayer voltage clamp amplifier (BC-525D, Warner Instruments) and the *cis* chamber (1 mL), representing the cytoplasmic compartment, was held at virtual ground. Solutions in both chambers were as follows: 1 mM EGTA, 250/125 mM HEPES/Tris, 50 mM KCl, 0.64 mM CaCl_2_, pH 7.35 as *cis* solution and 53 mM Ca(OH)_2_, 50 mM KCL, 250 mM HEPES, pH 7.35 as *trans* solution. The concentration of free Ca^2+^ in the *cis* chamber was calculated using the WinMaxC program (version 2.50; https://somapp.ucdmc.ucdavis.edu/pharmacology/bers/maxchelator/webmaxc/webmaxcE.htm). ER vesicles were added to the *cis* side, and fusion with the lipid bilayer was induced by making the *cis* side hyperosmotic by the addition of 400–500 mM KCl. After the appearance of potassium and chloride channels, the *cis* compartment was perfused with the *cis* solution. Single-channel currents were recorded at 0 mV by using a Bilayer Clamp BC-535 amplifier (Warner Instruments), filtered at 1 kHz, and digitized at 4 kHz. All experiments were performed at RT. Data acquisitions were performed using Digidata 1440A and Axoscope 10.2 software, and recordings were analyzed using Clampfit 10.2 (Molecular Devices). Open probability was identified by 50% threshold analyses using a minimum of 2 min of continuous recording. At the conclusion of each experiment, ryanodine (5 µM) was added to the *cis* chamber to confirm channels as RyR as previously described^51^.

### Endoplasmic reticulum Ca^2+^ leak assay

ER microsomes (5 μg ml^−1^) were diluted into a buffer (pH 7.2) containing 8 mM k-phosphocreatine, and 2 units per ml of creatine kinase, mixed with 3 μM Fluo-4 and added to multiple wells of a 96-well plate. Ca^2+^ loading of the microsomes was initiated by adding 1 mM ATP. After Ca^2+^ uptake (50 s), 3 μM thapsigargin was added to inhibit the Ca^2+^ reuptake by sarco-endoplasmic reticulum Ca^2+^-ATPase. ER Ca^2+^ leak was measured by the increase in intensity of the Fluo-4 signal (measured in a Tecan fluorescence plate reader). The Ca^2+^ leak was quantified as the percentage of uptake.

### Norepinephrine, cardiac troponin I, brain natriuretic peptide and blood gas levels

Hippocampal norepinephrine, cardiac troponin I and brain natriuretic peptide plasma levels were determined by using commercially available ELISA kits (Biomatik and Elabscience, respectively) according to the manufacturer’s instructions. Blood gas analyses was performed using CG8+ cartridges and the i-STAT1 analyzer from ABBOTT, according to the manufacturer’s instructions.

### Isolation of mitochondria

Mice hippocampi were removed, washed with PBS, and the mitochondria were isolated as previously described^90^. Briefly, tissues were placed in a homogenization medium at 5 ml g^–1^ of tissue (320 mM sucrose, 225 mM mannitol, 5 mM Tris-Hcl, pH 7.4). Tissues were homogenized in a glass Potter and centrifuged for 3 min at 1.330*g* at 4 °C. The pellet was then discarded, and the supernatant was centrifuged for 10 min at 21,000*g*. The mitochondrial pellet was resuspended in 15 ml of the mitochondrial isolation buffer (75 mM sucrose, 225 mM mannitol, 5 mM Tris-Hcl, pH 7.4) and centrifuged again for 10 min at 21,000*g* at 4 °C. Pellet was resuspended in the mitochondrial isolation assay and assessed for protein concentration using a Bradford assay.

### Mitochondrial Ca^2+^ content

In total, 20 μg of isolated mitochondria was sonicated and used to measure the mitochondrial Ca^2+^ content using the *o*-cresolphthalein complexone assay (Cayman Chemical) according to the manufacturer’s instructions and as previously described^46^.

### Mitochondrial reactive oxygen species production

Isolated mitochondria (20 μg) were incubated in VO_2_ buffer (250 mM sucrose, 50 mM KCl, 25 Mm Tris·HCl, and 10 mM K_2_HPO_4_, pH 7.4) in a 96-well black plate. ROS production was assessed at 37 °C for 60 min during states 3 and 4 respiration by adding respiration substrates before the addition of 50 μM dichlorodihydrofluorescein diacetate (H_2_DCFDA, Invitrogen). ROS production is directly proportional to fluorescence emission monitored at an excitation of 485 nm and emission of 528 nm with a microplate fluorimeter. Microplate data were compiled and analyzed using i-control Microplate Reader Software and results were expressed as arbitrary fluorescence units^91^.

### Global quantitative proteomics analysis

For global quantitative proteomics of fresh frozen hippocampal samples from MI and SHAM mice, diaPASEF^92^ (data-independent acquisition)-based proteomics were used. The cutoff values for differentially expressed proteins included *P* value < 0.05 (permutation-based FDR correction), fold change ≥ 1.5 and unique peptides ≥ 2 (volcano plot). The significantly changed proteins between MI and SHAM hippocampus were used for heat map, GO and KEGG analysis. Gene-set enrichment analyses also was performed to identify the statistically significant gene sets in a ranked gene list. More details are described in the Supplementary Information.

### Statistics

Data are presented as individual values with the mean ± s.e.m. Normal distribution was tested by Shapiro–Wilk normality and log normality tests. Statistical analyses were performed using an unpaired two-tailed Student’s *t*-test, and one- or two-way ANOVA with Tukey’s test post hoc correction for multiple comparisons. Minimum statistically significant differences were established at *P* < 0.05. No statistical methods were used to predetermine sample sizes, but our sample sizes are similar to those reported in previous publications^26^_._ Animals who died during the behavioral tests were excluded. No data were excluded for the remaining experiments.

### Reporting summary

Further information on research design is available in the Nature Portfolio Reporting Summary linked to this article.

## Online content

Any methods, additional references, Nature Portfolio reporting summaries, source data, extended data, supplementary information, acknowledgements, peer review information; details of author contributions and competing interests; and statements of data and code availability are available at 10.1038/s41593-023-01377-6.

## Supplementary information

Supplementary Information Extended methods. List of used drugs. List of used antibodies. Supplementary Figs. 1 and 2 and Extended Figs. 1–10. Full uncut gels shown in the supplementary figures. Used RStudio codes. References.

Reporting Summary

Supplementary Tables 1–10 Supplementary information on the human specimens, the animal models and the sequencing data.

## Data Availability

The data supporting the findings of this study are documented within the paper and Supplementary Information. Proteomics data are deposited at PRIDE under accession number PXD042295. No custom software codes were used*.* RNA-sequencing data are deposited on the Sequence Read Archive under accession number PRJNA956662. Data are accessible at the Center for Computational Mass Spectrometry MassIVE resource under accession MSV000091695. Source data are provided with this paper.

## Source data

Source Data Fig. 1 Unprocessed western blots and statistical source data.
Source Data Fig. 2 Statistical source data.
Source Data Fig. 3 Unprocessed western blots and statistical source data.
Source Data Fig. 4 Statistical source data.
Source Data Fig. 5 Statistical source data.
Source Data Fig. 6 Statistical source data.
Source Data Fig. 7 Unprocessed western blots and statistical source data.
Source Data Extended Data Fig. 1 Statistical source data.
Source Data Extended Data Fig. 2 Statistical source data.
Source Data Extended Data Fig. 3 Statistical source data.
Source Data Extended Data Fig. 4 Statistical source data.
Source Data Extended Data Fig. 10 Statistical source data.
