# Supplementary material for: Heart failure-induced cognitive dysfunction is mediated by intracellular Ca2+ leak through ryanodine receptor type 2
Source: Nat Neurosci. 2023 Jul 10;26(8):1365–78. doi: 10.1038/s41593-023-01377-6 (PMC10400432; doi:10.1038/s41593-023-01377-6)
Supplement: Supplementary file 1 — Extended methods. List of used drugs. List of used antibodies. Supplementary Figs. 1 and 2 and Extended Figs. 1–10. Full uncut gels shown in the supplementary figures. Used RStudio codes. References. [file 41593_2023_1377_MOESM1_ESM.pdf]

nature neuroscience

Article

https://doi.org/10.1038/s41593-023-01377-6

Heart failure-induced cognitive dysfunction is mediated by intracellular Ca^{2+} leak through ryanodine receptor type 2

In the format provided by the authors and unedited

extended methods and supplementary information

Extended methods.

Global quantitative proteomics analysis

For global quantitative proteomics of fresh frozen hippocampal samples from MI and SHAM mice, diaPASEF[1] (Data independent acquisition) based proteomics was used. In brief, frozen mouse hippocampal tissues were lysed by bead-beating in lysis buffer[2] (2% SDS, 1% SDC, 100 mM Tris-HCl pH 8.5, and protease inhibitors) and boiled for 10 min at 95°C, 1500 rpm. Protein reduction and alkylation of cysteines was performed with 10mM TCEP and 40mM CAA at 45°C for 10 min, followed by sonication in a water bath and then cooled down to room temperature. Cleared lysate was precipitated with the acetone-salt method, as previously described[3], and precipitated pellets were resuspended in SDC buffer (1% SDC, and 100 mM TrisHCl pH 8.5). Protein digestion was processed overnight by adding LysC and trypsin in a 1:50 ratio (µg of enzyme to µg of protein) at 37° C and 1400 rpm. Peptides were acidified by adding 1% TFA, vortexed, and subjected to StageTip clean-up via SDB-RPS. Peptides were loaded on one 14-gauge StageTip plugs. Peptides were washed two times with 200 µl 1% TFA 99% ethyl acetate followed 200 µl 0.2% TFA/5%ACN in centrifuge at 3000 rpm, followed by elution with 60 µl of 1% Ammonia, 50% ACN into Eppendorf tubes and dried at 45°C in a SpeedVac centrifuge. Samples were resuspended in 10 µl of LC buffer (3% ACN/0.1% FA). Peptide concentrations were determined using NanoDrop and 200 ng of each sample were used for PASEF and diaPASEF analysis on timsTOFPro.

For spectral library generation, 10 µg of each digested tissue sample was pooled and dried in speedVac. Pooled dried peptides were resuspended in 100 µl of 1% TFA, pH 2 and subjected to fractionation with mixed mode SDB-SCX StageTip[3]. Peptides were fractionated into 9 fractions; each fractionated peptide was in dissolved in 10 µl of (3% acetonitrile/ 0.1% formic acid) and injected using PASEF method.

Peptides were separated within 120 min at a flow rate of 400 nl/min on a reversed-phase C18 column with an integrated CaptiveSpray Emitter (25 cm x 75µm, 1.6 µm, IonOpticks). Mobile phases A and B were with 0.1% formic acid in water and 0.1% formic acid in ACN.

The fraction of B was linearly increased from 2 to 23% within 90 min, followed by an increase to 35% within 10 min, and a further increase to 80% before re-equilibration. The timsTOF Pro was operated in PASEF mode[1] with the following settings: Mass Range 100 to 1700m/z, 1/K0 Start 0.6 V·s/cm2, End 1.6 V·s/cm2, Ramp time 100ms, Lock Duty Cycle to 100%, Capillary Voltage 1600V, Dry Gas 3 l/min, Dry Temp 200°C, PASEF settings: 10 MSMS Frames (1.16 seconds duty cycle), charge range 0-5, active exclusion for 0.4 min, Target intensity 20000, Intensity threshold 2500, CID collision energy 59eV. A polygon filter was applied to the *m/z* and ion mobility plane to select features most likely representing peptide precursors rather than singly charged background ions. diaPASEF[1] experiment was acquired at defined 32 × 25 Th isolation windows from *m/z* 400 to 1,200. To adapt the MS1 cycle time in diaPASEF, we set the repetitions to 2 in the 16-scan diaPASEF scheme and to 4 in the 4-scan diaPASEF scheme in these experiments. The collision energy was ramped linearly as a function of the mobility from 59 eV at 1/K0=1.6 Vs cm-2 to 20 eV at 1/K0=0.6 Vs cm-2.

To generate the sample specific spectral libraries, the acquired PASEF raw files and diaPASEF raw files were searched with UniProt mouse database in Pulsar search engine using the Hybrid spectral library generation functionality of Spectromine with default settings[4]. The raw intensities for the proteins were calculated by summation of the peptide intensities. diaPASEF data were analyzed with Spectronaut Pulsar X[4], a mass spectrometer vendor software independent from Biognosys. The default settings were used for targeted analysis of diaPASEF data in Spectronaut, except the decoy generation was set to mutated. The false discovery rate (FDR) will be estimated with the mProphet approach and set to 1% at peptide precursor level and at 1% at protein level. Results obtained from Spectronaut were further analyzed using the Spectronaut statistical package.

Significantly changed protein abundance was determined by unpaired t-test with a threshold for significance of $p < 0.05$ (permutation-based FDR correction), fold-change ≥1.5, unique peptides ≥2. The significantly changed proteins between MI and SHAM hippocampus were processed for Volcano plot using R ggplot2 package[5] and hierarchical clustering using TBtools software[6]. Gene ontology (GO) and Kyoto Encyclopedia of Genes and Genomes (KEGG) for the DEGs were processed by R clusterProfiler package[7]. The potential biological processes (BP), cellular components (CC), molecular functions (MF), and pathways among the DEGs were shown in functional enrichment analysis results. The cut-off value for significant GO and KEGG results was adjusted p value <0.05.

Gene set enrichment analysis (GSEA) was performed to identify the statistically significant gene sets in the comparison between MI and SHAM. The gene list was pre-ranked based on fold change before enrichment analysis. GO enrichment analysis was performed through gseGO function in clusterProfiler package. The adjusted p-value<0.05 was set as the cut-off criteria. KEGG enrichment analysis was conducted by the GSEA software obtained from the Broad Institute (http://www.broad.mit.edu/GSEA)[8]. The significantly enriched pathways were defined by nominal |NES|>1, NOM p-value <0.05, and FDR q-value <0.25.

RNA Sequencing

Eukaryotic total RNA was extracted from 4 MI and 4 SHAM hippocampus samples. Quality control of RNA was performed by RNA Integrity Number (RIN) assessments[9]. Poly-A pull-down was used to enrich mRNAs from total RNA samples, then proceeded with library construction using Illumina TruSeq chemistry. Libraries were then sequenced using Illumina NovaSeq 6000 at Columbia Genome Center. Samples were multiplexed in each lane, which yielded targeted number of paired-end 100bp reads for each sample. RTA (Illumina) for base calling and bcl2fastq2 (version 2.19) for converting BCL to fastq format was used, coupled with adaptor trimming. We performed a pseudoalignment to a kallisto index created from transcriptomes (Mouse: GRCm38) using kallisto (0.44.0)[10]. Differentially expressed genes (DEGs) under various conditions using DESeq2 and designed R packages were used to test differential expression between two experimental groups from RNA-seq counts data. The cut-off values for DEGs included adjusted-p-value <0.05 and fold-change≥1.3. The following clustering and functional enrichment analysis (GO and KEGG pathway) DEGs between MI and SHAM hippocampus were performed as proteomics analysis. The cut-off value for significant GO and KEGG results was adjusted p-value <0.05.

List of used drugs

**Drug**	**Source**	**Concentration**
**Rycal S107**	Marks lab	75mg/kg/day in mice/ 10μM in cells
**Rycal ARM036**	Marks lab	20mg/kg/day in mice
**Propranolol**	Sigma Aldrich cat# 318-98-9	10mg/kg/day in mice/ 1μM in cells
**SD-208**	BLDpharm	10mg/kg/day in mice/1μM in cells
**Isoproterenol**	Sigma Aldrich cat# 5984-95-2	1μM in cells

List of used antibodies

Protein	Antibodies sources	Dilution	Secondary (dilution: 1/5000)
RyR2	Custom made: Acta Neuropathologica volume 134, pages749–767 (2017)	1/2500	IRDye® 800CW Goat anti-Rabbit IgG
pSer2808	Custom made. Circ Res. 2004;94(6): e61–e70.	1/1000	IRDye® 800CW Goat anti-Rabbit IgG
DNP	Millipore Oxyblot (S7150).<br>Lot. 3249659<br>Validated by Western blot of derivatized samples	1/1000	IRDye® 800CW Goat anti-Rabbit IgG
Cys-NO	ABM Y061263<br>Lot. AP10387<br>Validated by Western blot of at 1:0,000 using nitrosylated Cysteine–BSA as control.	1/1000	IRDye® 800CW Goat anti-Rabbit IgG
Calstabin2	Custom. JBC. 267 (14):9474-9477 (1992).	1/2500	IRDye® 800CW Goat anti-Rabbit IgG
Snap25	Thermofisher, MA5 17609<br>Lot. WD 3256763<br>Validated by western blot of PC-12 cell lines	1/1000	IRDye® 800CW Goat anti-Mouse IgG
Vamp8	Abnova, H00008673-B01P<br>WD3257113<br>Validate by Western blot of VAMP transfected Cell Lines	1/1000	IRDye® 800CW Goat anti-Rabbit IgG
Syt2	Abcam. Ab181123<br>Lot. GR164541<br>Validated by Western blot of rat and mouse brain tissue lysate	1/1000	IRDye® 800CW Goat anti-Rabbit IgG

**Cplx3**	Thermofisher, PA5-24148<br>Lot. WD3256486<br>Validated by Western blot analysis in mouse liver tissue lysate	1/1000	IRDye® 800CW Goat anti-Rabbit IgG
**GAPDH**	Thermofisher, PA1987<br>Lot. XJ358966<br>Validated by Western Blot in tissue extract of Ms Brain	1/5000	IRDye® 800CW Goat anti-mouse IgG
**p-AMPK**	Thermofisher, PA5-104982<br>Lot. VJ3103601<br>Validated by Western Blot of H202 treated EC304 Cells.	1/1000	IRDye® 800CW Goat anti-Rabbit IgG
**AMPK**	Abcam, ab207442<br>Lot. GR300197<br>Validated by Western Blot of Human skeletal muscle lysate	1/1000	IRDye® 800CW Goat anti-Rabbit IgG
**p-GSK3β (T216)**	Abcam, ab75745<br>Lot. 1010539<br>Validated by Western blot of 293 cell extracts treated with insulin or with a PKC activator. (phorbol 12-myristate 13-acetate. PMA).	1/1000	IRDye® 800CW Goat anti-Rabbit IgG
**GSK3β**	Abcam, ab32391<br>Lot. 1024397<br>Validated by Western blot of A431 cell lysate as well as wild type HAP1 whole cell lysate and GSK3β knockout HAP1 whole cell lysate.	1/1000	IRDye® 800CW Goat anti-Rabbit IgG
**p-Tau (S199)**	Thermofisher, 44-734G<br>Lot. 2285802<br>Validated by Western blot of untreated human recombinant Tau or treated with GSK-3β. The antibody has been used in several manuscripts for Western blots.	1/1000	IRDye® 800CW Goat anti-Rabbit IgG
**p-Tau (S202/T205)**	Abcam, ab210703<br>Lot. GR3256698<br>Validated by Western blot of human brain tissue lysate.	1/1000	IRDye® 800CW Goat anti-Rabbit IgG

**p-Tau (S262)**	Thermofisher, 44-750G<br>Lot. 2548898<br>Validated by Western blot of mouse brain, rat brain, and mouse kidney lysate	1/1000	IRDye® 800CW Goat anti-Rabbit IgG
**Tau**	Thermofisher, PA5-27287<br>Lot. WA3171630<br>Validated by Western blot of mouse and rat brain lysates	1/1000	IRDye® 800CW Goat anti-Rabbit IgG
**CDK5**	Thermofisher, AHZ0492<br>Lot. VJ3096132<br>Validated by Western blot of cell lines including CF7, Jurkat, PC-3, MDA-MB-231, A549, HeLa and HT-29. And with HEK (+/- CD5 ko).	1/1000	IRDye® 800CW Goat anti-mouse IgG
**P25**	Thermofisher, PA5-57726<br>Lot. XF3609058A<br>Validated by immunofluorescent staining of human cell line A549	1/1000	IRDye® 800CW Goat anti-Rabbit IgG
**APP**	Thermofisher, 14-9749-82<br>Lot. 2458748<br>Validated by Western Blot of mice and rat brain lysate	1/1000	IRDye® 800CW Goat anti-Rabbit IgG
**BACE1**	Abcam, ab183612<br>Lot. GR3240345<br>Validated by Western Blotting of Mouse hippocampus lysate	1/1000	IRDye® 800CW Goat anti-Rabbit IgG
**B-CTF**	Millipore, MABN381<br>Validated by Western Blotting in DAPT treated HEK293 cell lysate.	1/1000	IRDye® 800CW Goat anti-mouse IgG
**TGF-b1**	Abcam, ab215715<br>Lot. GR3412442<br>Validated by Western Blot of Wild-type A549, K562 and SH-SY5Y whole cell lysates	1/1000	IRDye® 800CW Goat anti-Rabbit IgG

**p-Smad3**	Abcam, ab52903<br>Lot. GR328135<br>Validated by Western Blot of HL-60 treated with TGF-ß cell lysates	1/1000	IRDye® 800CW Goat anti-Rabbit IgG
**Smad3**	Abcam, ab40854<br>Lot. GR3255567<br>Validated by Western Blot of Jurkat whole cell lysates	1/1000	IRDye® 800CW Goat anti-Rabbit IgG
**Nox2**	Thermofisher, PA5-79118<br>Lot. YA3804004<br>Validated by Western Blot of mice and rat thymus tissue and brain lysate	1/1000	IRDye® 800CW Goat anti-Rabbit IgG

Supplementary Figures

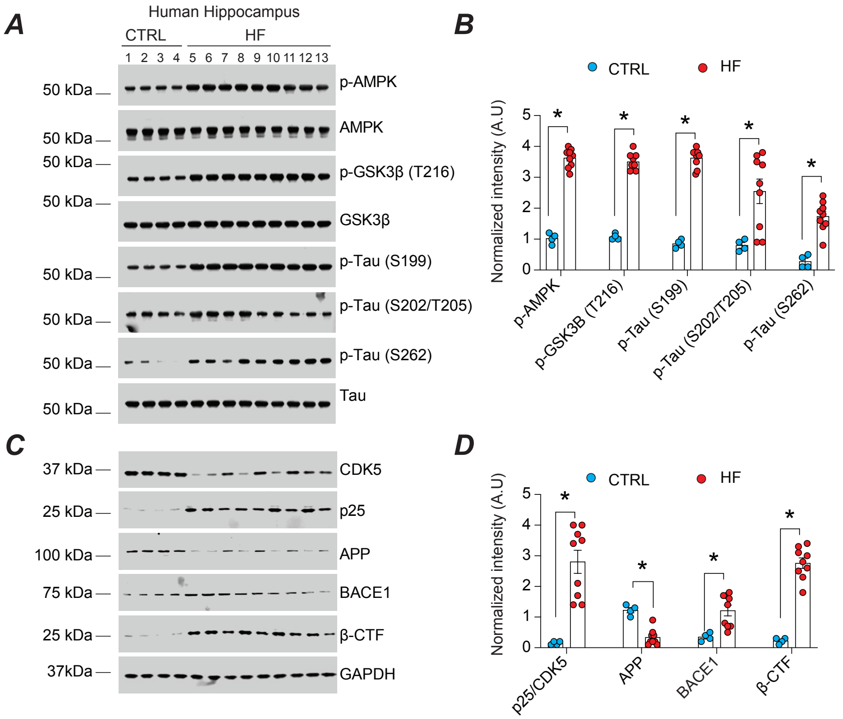

Fig.S1. Alzheimer's-like signaling pathways in human HF. A) Immunoblots showing phosphorylated and total AMPK, phosphorylated (on Thr216) and total GSK-β, phosphorylated Tau (on Ser199, Ser202/Thr205, and Ser262), and total Tau expression in the hippocampi of controls and HF patients. **B)** Bar graphs depicting the ratio of each protein phosphorylation to its total expression. **C)** Immunoblots showing the expression levels of CDK5, p25, APP, BACE1, β-CTF, and GAPDH in the hippocampi of control and HF patients. **D)** Bar graphs depicting the ratio of each protein expression to GAPDH and p25 to CDK5 expression levels. Controls (n=4), HF patients (n=9). Individual values are shown with mean±SEM (t-test * $p < 0.05$, Controls vs. HF patients). All statistical tests were two-sided. Data are derived from biologically independent samples. *Source file extended Fig.11.*

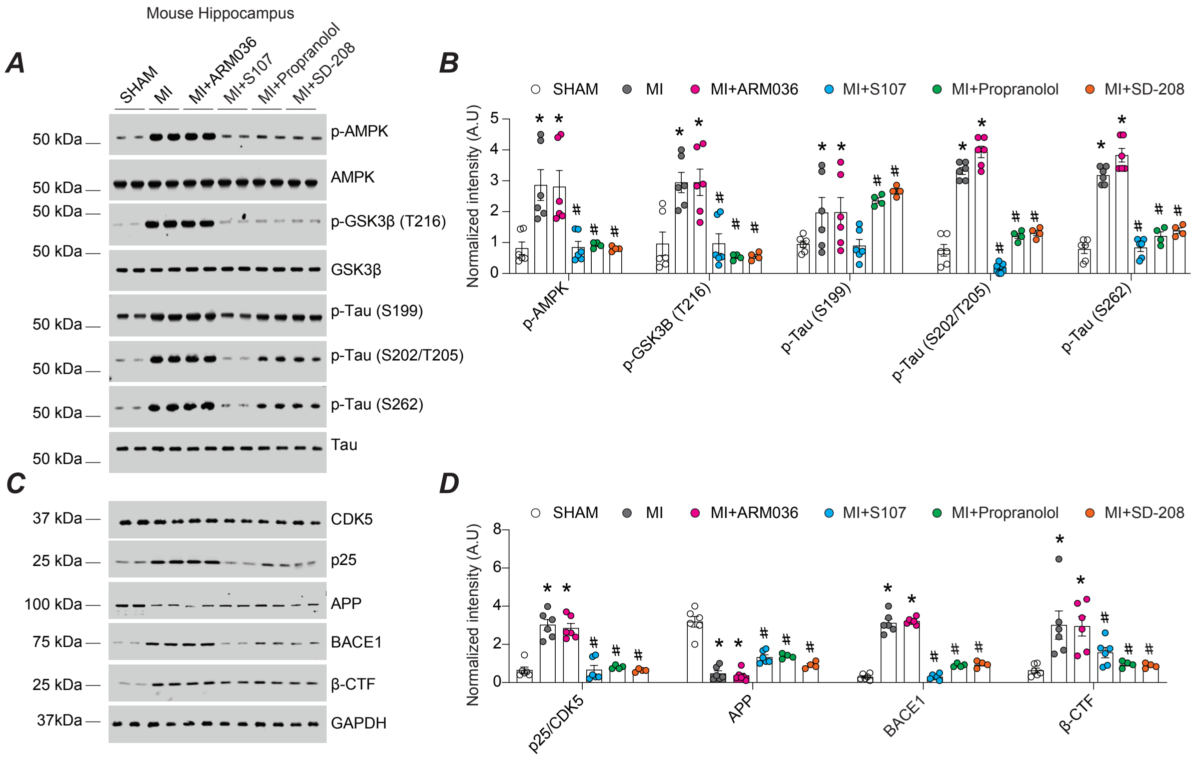

Fig.S2. Alzheimer's-like signaling pathways in murine model of HF. A) Immunoblots showing phosphorylated and total AMPK, phosphorylated (on Thr216) and total GSK-β, phosphorylated Tau (on Ser199, Ser202, Thr205, and Ser262), and total Tau expression in the hippocampi of SHAM, MI, MI+ARM036, MI+S107, MI+ propranolol and MI+SD-208 mice. **B)** Bar graphs depicting the ratio of each protein phosphorylation to its total expression. Sample size n=6 in SHAM, n=6 in MI, n=6 in MI+ARM036, n=6 in MI+S107, n=4 in MI+propranolol and n=4 in MI+SD-208. **C)** Immunoblots showing the expression levels of CDK5, p25, APP, BACE1, β-CTF, and GAPDH in the hippocampi of SHAM, MI, MI+ARM036, MI+S107, MI+ propranolol and MI+SD-208 mice. **D)** Bar graphs depicting the ratio of each protein level to GAPDH and p25 to CDK5 expression levels. Sample size n=6 in SHAM, n=6 in MI, n=6 in MI+ARM036, n=6 in MI+S107, n=4 in MI+propranolol and n=4 in MI+SD-208. Individual values are shown with mean±SEM (One way-ANOVA and Tukey's test post-hoc correction for multiple comparisons show * $p < 0.05$, SHAM vs. MI or MI+ARM036; #$p < 0.05$, MI vs. MI+S107, MI+ propranolol or MI+SD-208). All statistical tests were two-sided. Data are derived from biologically independent samples. <u>*Source file Extended Fig.12.*</u>

Extended Figure Legends:

Extended Data Fig.1: Murine model of leaky RyR2 (phospho-memetic mutation) is associated with cognitive dysfunction. A) Open field test of SHAM (n=14), S2808A-SHAM (n=8), S2808A-MI (n=8), S2808D (n=13), and S2808D+S107 (n=8) mice. Ratios of total time spent in the center area versus periphery area within first (1st) 3min and second (2nd) 3min are shown. **B)** Elevated plus maze test in SHAM (n=14), S2808A-SHAM (n=8), S2808A-MI (n=8), S2808D (n=13), and S2808D+S107 (n=8) mice. Ratios of time spent on the open-arm versus closed-arm are shown. **C)** Novel object recognition test in SHAM (n=14), S2808A-SHAM (n=8), S2808A-MI (n=8), S2808D (n=13), and S2808D+S107 (n=8) mice. Discrimination index is shown. **D)** Morris water maze test (learning curves) in SHAM (n=14), S2808A-SHAM (n=8), S2808A-MI (n=8), S2808D (n=13), and S2808D+S107 (n=8) mice. **E)** Probe trials after escape platform removed in the same groups showing the total duration spent in the target quadrant. **F)** Number of target crossings SHAM (n=14), S2808A-SHAM (n=8), S2808A-MI (n=8), S2808D (n=13), and S2808D+S107 (n=8) mice. **G**) Heat maps showing the latency from each group at Day 2 and Day 4. Individual values are shown with mean ± SEM (t-test * p <0.05 in panel **A** shows significance between the first 3min and second 3min of the same groups. One-way ANOVA was used to compare the difference between the 5 groups in panel B, C, E and F; Two-way ANNOVA was used in panel D. Tukey's test post-hoc correction for multiple comparisons was used; * p <0.05, S2808A-SHAM vs. S2808D or S2808D+S107; $^{\#}$ p<0.05, S2808D vs. S2808D+S107. No differences were detected between S2808A-SHAM and S2808A-MI. All statistical tests were two-sided. Data are derived from biologically independent samples.

Extended Data Fig.2: Cognitive function in RyR1-S2844D mice. Open field test using WT mice (n=10) and a mouse model with leaky RyR1 channels (S2844D) (n=21). Ratios of total time spent in the center area versus periphery area within first 3 min and second 3 min are shown. **B)** Elevated plus maze test in WT mice (n=10) and S2808D (n=21). Ratios of time spent in the open-arm versus closed-arm are shown. **C)** Novel object recognition test in WT mice (n=10) and S2808D (n=21). Discrimination index is shown. **D)** Morris water maze test (learning curves) in WT mice (n=10) and S2808D (n=21). **E)** Probe trials after escape platform removed in the same groups showing the total duration spent in the target quadrant in WT mice (n=10) and S2808D (n=21). **F)** Number of target crossings in WT mice (n=10) and S2808D (n=21). **G**) Heat maps showing the latency from each group at Day 2 and Day 5. Individual values are shown with mean±SEM. T-test was used in panel A-C, E-F, * p <0.05 in panel A shows significance between the first 3min and second 3min of each group). Two-way ANOVA was used in panel D. Tukey's test post-hoc correction for multiple comparisons was used. All statistical tests were two-sided. Data are derived from biologically independent samples.

Extended Data Fig.3: Phospho-memetic mutation (RyR2-S2808D mice) induces ER Ca^{2+} leak in the hippocampus. A-B) Representative SDS-PAGE analysis and quantification of modified RyR2 and calstabin2 immunoprecipitated from hippocampus of S2808A-SHAM (n=4), S2808A-MI (n=4), S2808D (n=4), S2808D+S107 mice (n=4) (IP RyR2: Bands normalized to total RyR2); n=4 in each group. **C**) ER Ca^{2+} leak measured in microsomes from hippocampi of S2808A-SHAM (n=4), S2808A-MI (n=4), S2808D, S2808D+S107 mice (n=4). **D)** Bar graphs represent the quantification of Ca^{2+} leak as the percentage of uptake in all the experimental groups (n=4 per group). **E)** Single-channel traces of RyR2 incorporated in planar lipid bilayers with 150 nM Ca^{2+} in the *cis* chamber, corresponding to representative experiments performed with hippocampal samples from S2808A-SHAM, S2808A-MI, S2808D, S2808D+S107 mice. **F-G-H)** RyR2 open probability (Po), mean open time (To), and mean close time (Tc) in S2808A-SHAM, S2808A-MI, S2808D, and S2808D+S107 mice (n=n=5, 5, 4 and 4 respectively). Individual values are shown with mean ± SEM. One way-ANOVA and Tukey's test post-hoc correction for multiple comparisons shows * p <0.05, S2808A-SHAM vs. S2808D or

S2808D+S107; # p<0.05, S2808D vs. S2808D+S107. No differences were detected between S2808A-SHAM and S2808A-MI. All statistical tests were two-sided. Data are derived from biologically independent samples.

Extended Data Fig.4: TGF-β activation in HF. A) Immunoblots showing expressing levels of TGF-β, phosphorylated SMAD3, total SMAD3, and NOX2 binding to RyR2 in the hippocampi of controls (n=4) and HF patients (n=9). **B)** Bar graphs depicting the ratio of TGF-β expression normalized to GAPDH, phosphorylated SMAD3 to total SMAD3 and NOX2 binding to RyR2 (IP RyR2). Same quantity of proteins were loaded on two separate gels and blotted separately for SMAD3 and pSMAD3. Individual values are shown with mean ± SEM (t-test * p <0.05, Controls vs. HF patients). **C)** Immunoblots showing expressing levels of TGF-β, phosphorylated SMAD3, total SMAD3, and NOX2 binding to RyR2 in the hippocampi of SHAM, MI, MI+ARM036, MI+S107, MI+ propranolol and MI+SD-208 mice (n=6, 6, 6, 6, 4 and 4 respectively). **D)** Bar graphs depicting the ratio of TGF-β expression normalized to GAPDH, phosphorylated SMAD3 to total SMAD3 and NOX2 binding to RyR2 (IP RyR2). Same quantity of proteins were loaded on two separate gels and blotted separately for SMAD3 and pSMAD3. Individual values are shown with mean±SEM. One-way ANOVA and Tukey's test post-hoc correction for multiple comparisons shows * p <0.05, SHAM vs. MI, MI+ARM036 or MI+S107; $^{\#}$p<0.05, MI vs. MI+S107, MI+ propranolol or MI+SD-208. All statistical tests were two-sided. Data are derived from biologically independent samples.

Extended Data Fig.5: Pre-ranked gene set enrichment analysis (GSEA) of the hippocampal proteomics. Dot plots show: **A)** Top 20 up- and top 20 down-regulated GO biological process, **B)** top 10 up- and top 20 down-regulated GO cellular component, **C)** top 10 up- and top 20 down-regulated GO molecular function terms. Significantly changed protein abundance was determined by unpaired t-test with a threshold for significance of p <0.05 (permutation-based FDR correction), fold-change ≥1.5, unique peptides ≥2. Data are derived from biologically independent samples. All statistical tests were two-sided. *Source file MassIVE MSV000091695*.

Extended Data Fig.6: Gene set enrichment analysis (GSEA) of the hippocampal proteomics. The enrichment plots of representative KEGG pathway gene sets demonstrate that oxidative phosphorylation **(A),** Parkinson's disease **(B)**, Alzheimer's disease **(C)**, and Huntington's disease **(D)** are significantly enriched in MI compared to SHAM. The heatmap on the right side of each panel visualizes the genes contributing to the enriched pathways. For the detailed list *see Supplementary Table 8*. Signal-to-noise ratio was used to rank the genes per their correlation with either MI phenotype (red) or SHAM phenotype (blue). The y-axis represents enrichment score (ES) and on the x-axis are genes (vertical black lines) represented in gene sets. The GSEA analysis calculates an enrichment score (the maximum deviation from zero) reflecting the degree of over-representation of a gene set at the top or the bottom of the ranked gene list. A positive ES indicates gene set enrichment at the top of the ranked list; a negative ES indicates gene set enrichment at the bottom of the ranked list. NES, normalized enrichment score; FDR, FDR adjusted p-value.

Extended Data Fig.7: RNA sequencing analysis. RNA-sequencing was performed on the hippocampi of SHAM and MI mice (n=4 for each group). **A)** The Volcano plot shows differentially expressed genes (p-adj<0.05, fold-change ≥1.3) in SHAM and MI mice. Red indicates up-regulated, while blue represents down-regulated genes. Black indicates unchanged expression levels. **B)** The heat map shows significantly dysregulated genes (down-regulated: 2003, up-regulated: 1149 genes), the color scale bar shows the row normalized log2 protein abundance. **C)** Dot plots show top 10 GO biological processes, **D)** molecular functions, **E)** cellular components, and **F)** KEGG pathways that were enriched from differentially expressed genes. Significantly changed gene abundance was determined by unpaired t-test with a threshold for significance of p <0.05 (permutation-based FDR correction), fold-change ≥1.5. Data are derived from biologically independent samples. All statistical tests were two-sided. *See Supplementary Table 9 for gene list. Data are accessible on SRA- Accession: PRJNA956662.*

Extended Data Fig.8: Pre-ranked gene set enrichment analysis (GSEA) of RNA sequencing. Dot plots show: **A)** Top 20 up- and top 20 down-regulated GO biological process, **B)** top 20 up- and top 20 down-regulated GO cellular component, **C)** top 20 up- and top 20 down-regulated GO molecular function terms. Significantly changed gene abundance was determined by unpaired t-test with a threshold for significance of p <0.05 (permutation-based FDR correction), fold-change ≥1.5. Data are derived from biologically independent samples. All statistical tests were two-sided.

Extended Data Fig.9: Gene set enrichment analysis (GSEA) of the hippocampal RNA sequencing. The enrichment plots of representative KEGG pathway gene sets demonstrate that oxidative phosphorylation **(A),** Parkinson's disease **(B),** Alzheimer's disease **(C),** and Huntington's disease **(D)** are significantly enriched in MI compared to SHAM. The heatmap on the right side of each panel visualizes the genes contributing to the enriched pathways. For the detailed list, *see Supplementary Table 10*. Signal-to-Noise ratio was used to rank the genes per their correlation with either MI phenotype (red) or SHAM phenotype (blue). The y-axis represents enrichment score (ES) and on the x-axis are genes (vertical black lines) represented in gene sets. The GSEA analysis calculates an enrichment score (the maximum deviation from zero) reflecting the degree of over-representation of a gene set at the top or the bottom of the ranked gene list. A positive ES indicates gene set enrichment at the top of the ranked list; a negative ES indicates gene set enrichment at the bottom of the ranked list. NES, normalized enrichment score; FDR, FDR adjusted p-value.

Extended Data Fig.10: Mitochondrial Ca^{2+} overload and oxidative stress in HF. A) Cohort plot representation of differentially expressed mitochondrial proteins (SHAM vs MI) from 4 significantly enriched mitochondrial GO-terms and generated by GOplot**.** The color map represents fold change of proteins (log_2 scale). **B**) Ca^{2+} accumulation in isolated mitochondria from SHAM (n=6), MI (n=5), MI+ARM036 (n=5), and MI+S107 (n=5) mice. **C)** Reactive oxygen species (ROS) production in isolated mitochondria from SHAM (n=6), MI (n=6), MI+ARM036 (n=6), and MI+S107 (n=5) mice. Individual values are shown with mean±SEM (one-way ANOVA and Tukey's test post-hoc correction for multiple comparisons show * p <0.05, SHAM vs. MI or MI+ARM036; $^{\#}$p<0.05, MI vs. MI+S107). All statistical tests were two-sided.

Full uncut gels shown in the supplementary figures.

Fig.S1. Alzheimer's-like signaling pathways in human HF

Human tissues

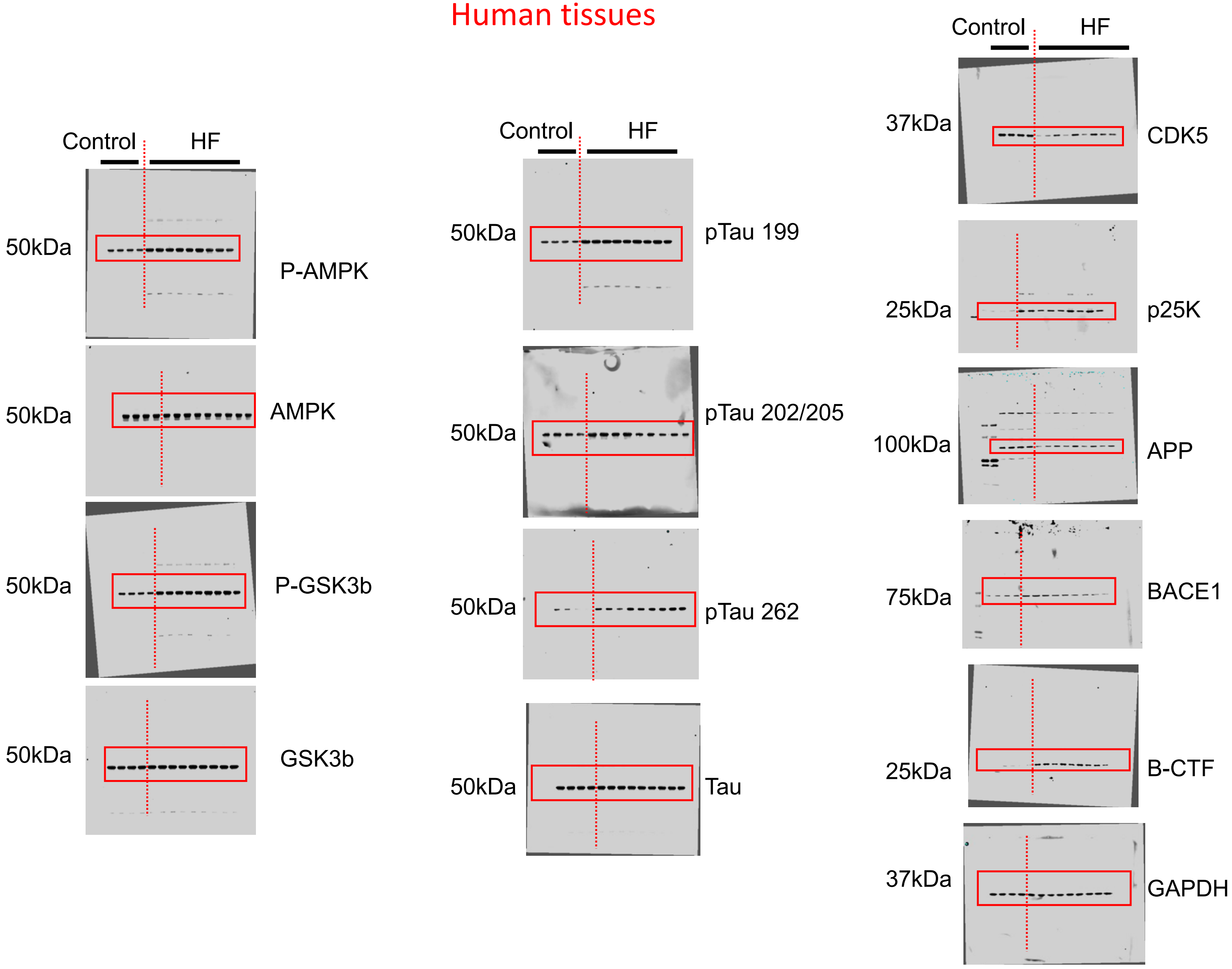

212 **Fig.S2. Alzheimer's-like signaling pathways in murine model of HF**

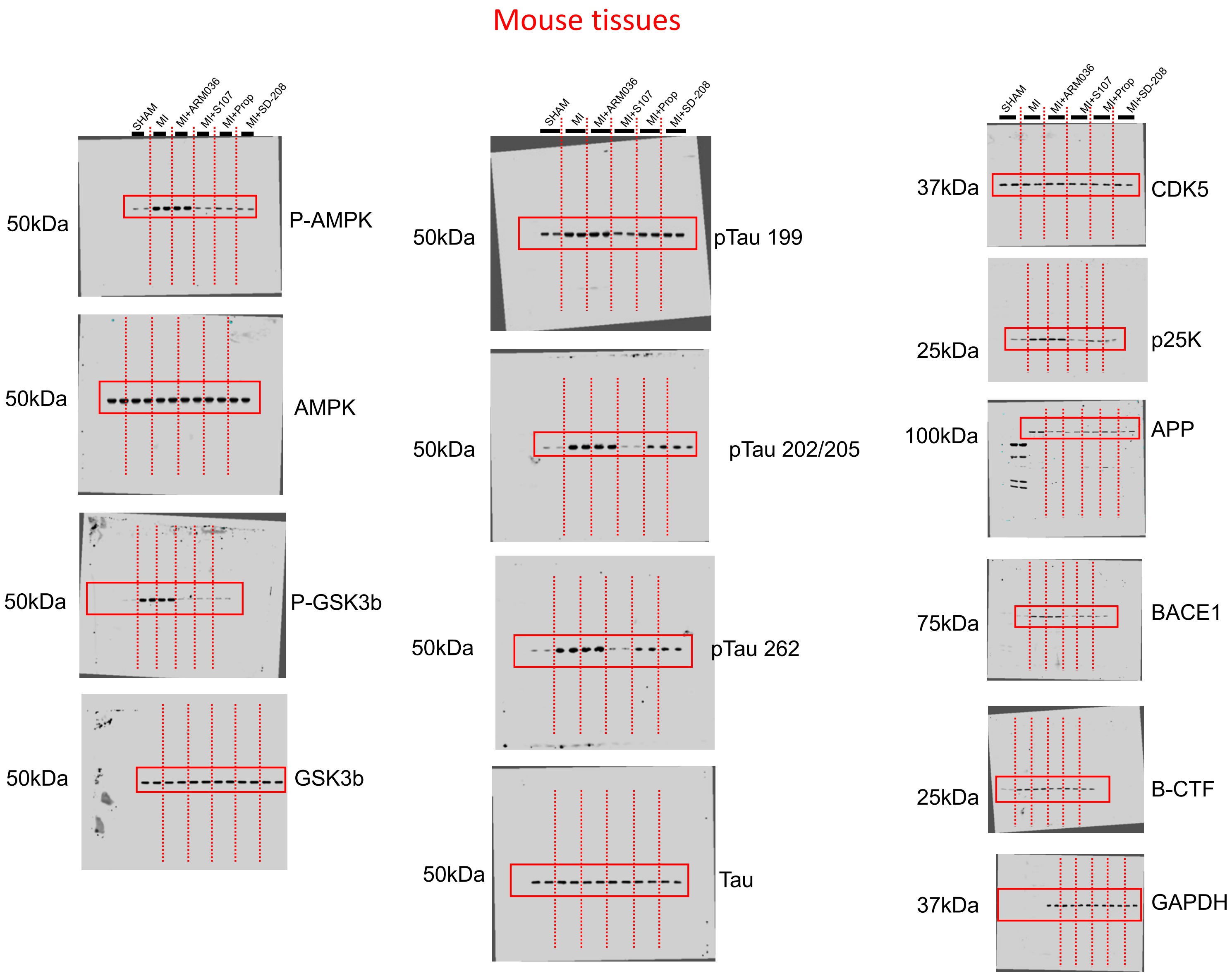

213

Rstudio used codes.

Figure 6A, ED_Fig7A

```
library(ggplot2)
library(clusterProfiler)
library(org.Mm.eg.db)
library(stringr)
library(GOplot)

DEG<-read.csv("FULL.csv",header=T)

DEG$significant = as.factor(ifelse(DEG$padj < 0.05 & abs(DEG$log2FoldChange) > 0.58,
                                   ifelse(DEG$log2FoldChange > 0.58,'up','down'),'no'))
table(DEG$significant)

g <- ggplot(data=DEG,
            aes(x=log2FoldChange,
                y=-log10(padj),color=significant)) +
  geom_point(alpha=0.4, size=1.75) + theme_bw() +
  theme(panel.grid.major=element_line(colour=NA),
```

```
        panel.background = element_rect(fill = "transparent",colour = NA)) +
  xlab("log2 fold change") +
  ylab("-log10 p-adj") +
  theme(plot.title = element_text(size=15,hjust = 0.5)) +
  geom_hline(yintercept=1.30102999566 ,linetype=4) +
  geom_vline(xintercept=c(-0.58,0.58) ,linetype=4 ) +
  scale_colour_manual(values = c('blue','black','red'))

print(g)



DEG<-read.csv("FC1.5.csv",header=T)

all_gene_id<-DEG$Gene
length(all_gene_id)
keytypes(org.Mm.eg.db)
all_gene_id_to_ENTREZID = bitr(all_gene_id, fromType="SYMBOL", toType="ENTREZID", OrgDb="org.Mm.eg.db")
```

Figure 6C, ED_Fig7C

```
ego_BP <- enrichGO(gene    = all_gene_id_to_ENTREZID$ENTREZID,
                   OrgDb         = org.Mm.eg.db,
                   ont           = "BP",
                   pAdjustMethod = "BH",
                   pvalueCutoff  = 0.05,
                   qvalueCutoff  = 0.2,
                   readable      = TRUE)
p1 <- dotplot(ego_BP, showCategory=10,title="GO Biological Processes")
p2 <- p1 + scale_y_discrete(labels = function(y) str_wrap(y, width = 40))
p2
```

Figure 6D, ED_Fig7D

```
ego_MF <- enrichGO(gene    = all_gene_id_to_ENTREZID$ENTREZID,
                   OrgDb         = org.Mm.eg.db,
                   ont           = "MF",
                   pAdjustMethod = "BH",
                   pvalueCutoff  = 0.05,
                   qvalueCutoff  = 0.2,
                   readable      = TRUE)
p1 <- dotplot(ego_MF, showCategory=10,title="GO Molecular Functions")
p2 <- p1 + scale_y_discrete(labels = function(y) str_wrap(y, width = 40))
p2
```

Figure 6E, ED_Fig7E

```
ego_CC <- enrichGO(gene    = all_gene_id_to_ENTREZID$ENTREZID,
                OrgDb        = org.Mm.eg.db,
                ont          = "CC",
                pAdjustMethod = "BH",
                pvalueCutoff  = 0.05,
                qvalueCutoff  = 0.2,
                readable      = TRUE)
p1 <- dotplot(ego_CC, showCategory=10,title="GO Cellular Components")
p2 <- p1 + scale_y_discrete(labels = function(y) str_wrap(y, width = 40))
p2
```

Figure 6F, ED_Fig7F

```
KEGG_all <- enrichKEGG(gene         = all_gene_id_to_ENTREZID$ENTREZID,
                  organism     = "mmu",
                  keyType      = "kegg",
                  pvalueCutoff = 0.05,
                  pAdjustMethod = "BH",
                  qvalueCutoff = 0.2)
KEGG_allx <- setReadable(KEGG_all, 'org.Mm.eg.db', 'ENTREZID')
p1 <- dotplot(KEGG_allx, showCategory=10,title="KEGG pathways")
```

```
p2 <- p1 + scale_y_discrete(labels = function(y) str_wrap(y, width = 40))
p2
cnetplot(KEGG_allx, showCategory = 6, circular = TRUE, colorEdge = TRUE, node_label = "category")
```

Figure 7A, ED_Fig10A

```
go<-read.csv("go.csv",header=T)

genelist<-read.csv("genelist.csv",header=T)

genename <- NULL

for (i in c(1:6)){

  list <- c(go[i,4])

  temp <- strsplit(list,",",)[[1]]

  genename <- append(genename,temp,after = length(genename))}

genename <- genename[-which(duplicated(genename))]
```

```
diffgene <- genelist[which(genelist$ID %in% genename),]
diffgene$logFC <- as.numeric(diffgene$logFC)
circ <- circle_dat(go,diffgene)
diffgene$ID <- toupper(diffgene$ID)
process<-read.csv("process.csv",header=T)
process<-process[1:6,]
chord <- chord_dat(circ,diffgene,process)

GOChord(chord, title="GOChord plot",
        space = 0.02,
        limit = c(3, 5),
        gene.order = 'logFC', gene.space = 0.25, gene.size = 4,
        lfc.col=c('red', 'white','green'),
        ribbon.col=colorRampPalette(c("royalblue3", "gray98"))(6),border.size=NA,process.label=8)
```

Figure ED_Fig5 and ED_Fig8

```
gene<-read.csv("MS preranked gene list.csv",header=T)
genesymbol<-gene$SYMBOL
length(genesymbol)
ENTREZID = bitr(genesymbol, fromType="SYMBOL", toType="ENTREZID", OrgDb="org.Mm.eg.db")
gene_df <- data.frame(logFC=gene$logFC, SYMBOL = gene$SYMBOL)
```

```
gene_df <- merge(gene_df,ENTREZID,by="SYMBOL")
sortdf<-gene_df[order(gene_df $logFC, decreasing = T),]
gene.expr = sortdf $logFC
names(gene.expr) <- sortdf $ENTREZID

GOBP<-gseGO(
  gene.expr,
  ont = "BP",
  OrgDb= org.Mm.eg.db,
  keyType = "ENTREZID",
  exponent = 1,
  minGSSize = 10,
  maxGSSize = 500,
  eps = 1e-10,
  pvalueCutoff = 0.05,
  pAdjustMethod = "BH",
  verbose = TRUE,
  seed = FALSE,
  by = "fgsea")

p1 <- dotplot(GOBP,split=".sign",title="GO Biological Processes")+facet_grid(~.sign)
p2 <- p1 + scale_y_discrete(labels = function(y) str_wrap(y, width = 40))
```

```
p2


GOCC<-gseGO(
  gene.expr,
  ont = "CC",
  OrgDb= org.Mm.eg.db,
  keyType = "ENTREZID",
  exponent = 1,
  minGSSize = 10,
  maxGSSize = 500,
  eps = 1e-10,
  pvalueCutoff = 0.05,
  pAdjustMethod = "BH",
  verbose = TRUE,
  seed = FALSE,
  by = "fgsea")

p1 <- dotplot(GOCC,split=".sign",title="GO Cellular Components")+facet_grid(~.sign)
p2 <- p1 + scale_y_discrete(labels = function(y) str_wrap(y, width = 40))
p2
```

```
GOMF<-gseGO(
  gene.expr,
  ont = "MF",
  OrgDb= org.Mm.eg.db,
  keyType = "ENTREZID",
  exponent = 1,
  minGSSize = 10,
  maxGSSize = 500,
  eps = 1e-10,
  pvalueCutoff = 0.05,
  pAdjustMethod = "BH",
  verbose = TRUE,
  seed = FALSE,
  by = "fgsea")
p1 <- dotplot(GOMF,split=".sign",title="GO Molecular Functions")+facet_grid(~.sign)
p2 <- p1 + scale_y_discrete(labels = function(y) str_wrap(y, width = 40))
p2
```

References

1 Meier, F. *et al.* diaPASEF: parallel accumulation-serial fragmentation combined with data-independent acquisition. *Nat Methods* **17**, 1229-1236, doi:10.1038/s41592-020-00998-0 (2020).

2 Kulak, N. A., Pichler, G., Paron, I., Nagaraj, N. & Mann, M. Minimal, encapsulated proteomic-sample processing applied to copy-number estimation in eukaryotic cells. *Nat Methods* **11**, 319-324, doi:10.1038/nmeth.2834 (2014).

3 Adachi, J. *et al.* Improved Proteome and Phosphoproteome Analysis on a Cation Exchanger by a Combined Acid and Salt Gradient. *Anal Chem* **88**, 7899-7903, doi:10.1021/acs.analchem.6b01232 (2016).

4 Bruderer, R. *et al.* Extending the limits of quantitative proteome profiling with data-independent acquisition and application to acetaminophen-treated three-dimensional liver microtissues. *Mol Cell Proteomics* **14**, 1400-1410, doi:10.1074/mcp.M114.044305 (2015).

5 Zhou, L. *et al.* Bioinformatics analyses of significant genes, related pathways and candidate prognostic biomarkers in glioblastoma. *Mol Med Rep* **18**, 4185-4196, doi:10.3892/mmr.2018.9411 (2018).

6 Chen, C. *et al.* TBtools: An Integrative Toolkit Developed for Interactive Analyses of Big Biological Data. *Mol Plant* **13**, 1194-1202, doi:10.1016/j.molp.2020.06.009 (2020).

7 Yu, G., Wang, L. G., Han, Y. & He, Q. Y. clusterProfiler: an R package for comparing biological themes among gene clusters. *OMICS* **16**, 284-287, doi:10.1089/omi.2011.0118 (2012).

8 Subramanian, A. *et al.* Gene set enrichment analysis: a knowledge-based approach for interpreting genome-wide expression profiles. *Proc Natl Acad Sci U S A* **102**, 15545-15550, doi:10.1073/pnas.0506580102 (2005).

9 Griffith, M., Walker, J. R., Spies, N. C., Ainscough, B. J. & Griffith, O. L. Informatics for RNA Sequencing: A Web Resource for Analysis on the Cloud. *PLoS Comput Biol* **11**, e1004393, doi:10.1371/journal.pcbi.1004393 (2015).

10 Hickman, R. A. *et al.* Gonadotroph tumours with a low SF-1 labelling index are more likely to recur and are associated with enrichment of the PI3K-AKT pathway. *Neuropathol Appl Neurobiol* **47**, 415-427, doi:10.1111/nan.12675 (2021).
