# Supplementary material for: Heart failure-induced cognitive dysfunction is mediated by intracellular Ca2+ leak through ryanodine receptor type 2
Source: Nat Neurosci. 2023 Jul 10;26(8):1365–78. doi: 10.1038/s41593-023-01377-6 (PMC10400432; doi:10.1038/s41593-023-01377-6)
Supplement: Supplementary file 2 — Reporting Summary [file 41593_2023_1377_MOESM2_ESM.pdf]

nature portfolio

Corresponding author(s): Haikel Dridi
Last updated by author(s): Apr 16, 2023

Reporting Summary

Nature Portfolio wishes to improve the reproducibility of the work that we publish. This form provides structure for consistency and transparency in reporting. For further information on Nature Portfolio policies, see our Editorial Policies and the Editorial Policy Checklist.

Statistics

For all statistical analyses, confirm that the following items are present in the figure legend, table legend, main text, or Methods section.

n/a	Confirmed	
☐	☒	The exact sample size (*n*) for each experimental group/condition, given as a discrete number and unit of measurement
☐	☒	A statement on whether measurements were taken from distinct samples or whether the same sample was measured repeatedly
☐	☒	The statistical test(s) used AND whether they are one- or two-sided<br>*Only common tests should be described solely by name; describe more complex techniques in the Methods section.*
☐	☒	A description of all covariates tested
☐	☒	A description of any assumptions or corrections, such as tests of normality and adjustment for multiple comparisons
☐	☒	A full description of the statistical parameters including central tendency (e.g. means) or other basic estimates (e.g. regression coefficient) AND variation (e.g. standard deviation) or associated estimates of uncertainty (e.g. confidence intervals)
☐	☒	For null hypothesis testing, the test statistic (e.g. *F*, *t*, *r*) with confidence intervals, effect sizes, degrees of freedom and *P* value noted<br>*Give P values as exact values whenever suitable.*
☒	☐	For Bayesian analysis, information on the choice of priors and Markov chain Monte Carlo settings
☒	☐	For hierarchical and complex designs, identification of the appropriate level for tests and full reporting of outcomes
☐	☒	Estimates of effect sizes (e.g. Cohen's *d*, Pearson's *r*), indicating how they were calculated

Our web collection on statistics for biologists contains articles on many of the points above.

Software and code

Policy information about availability of computer code

Data collection	Heart function was collected by electrocardiography using the Vevo 2100; VisualSonics, Toronto, Canada, imaging system.<br>Western blot was scanned by Licor scanner and analyzed by imageStudio software version 2022, 07.01.<br>Behavioral assays were recorded and analyzed by Etho Vision XT video tracking software (Noldue Information Technology<br>The brain imaging was acquired using an Inveon MicroPET scanner (Siemens, Germany) and MicroCT (MILabs, Netherlands).<br>Field excitatory postsynaptic potentials evaluation and Single channels experiments were recorded by Digidata 1440A and Axoscope 10.2 software and analyzed using Clampfit 10.2 (Molecular Devices).<br>The microsomal leak assay, mitochondrial ROS and Calcium were recorded on a plate reader from Tecan i-Control 1.12.<br>The RNA sequencing was done using the llumina NovaSeq 6000 at Columbia Genome Center.<br>The proteomics was done using timsTOFPro and Spectronaut software version 14.<br>The calcium imaging was recorded argon laser of a Zeiss LSM 800 inverted confocal microscope (40× oil immersion lens).
Data analysis	We used graphpad software version 8.0 for data and statistical analysis except for the sequencing data.<br>The RNA and proteomic sequencing analysis was done using Rstudio software version 4.1.2. Gene Set Enrichment Analysis (GSEA) was done using the online GSEA website.<br>Figures were made using Adobe illustrator V26.21.<br>Calcium imaging analysis was done using ImageJ software 1.53t.<br>Brain imaging analysis was done using VivoQuant version 4 (Invicro, MA).

For manuscripts utilizing custom algorithms or software that are central to the research but not yet described in published literature, software must be made available to editors and reviewers. We strongly encourage code deposition in a community repository (e.g. GitHub). See the Nature Portfolio guidelines for submitting code & software for further information.

Data

Policy information about availability of data

All manuscripts must include a data availability statement. This statement should provide the following information, where applicable:

- Accession codes, unique identifiers, or web links for publicly available datasets
- A description of any restrictions on data availability
- For clinical datasets or third party data, please ensure that the statement adheres to our policy

The data supporting the findings of this study are documented within the paper/supplementary information. Proteomic data are accessible on the Center for Computational Mass Spectrometry. Accession number: MassIVE MSV000091695.

Human research participants

Policy information about studies involving human research participants and Sex and Gender in Research.

Reporting on sex and gender	We used de-identified human hippocampal autopsies.<br>3 females and 1 male in the control group<br>3 females and 6 males in the heart failure group
Population characteristics	The control specimens were from 92, 81, 84 and 85 years old healthy donors (more details are listed in supplementary table 1).<br>The heart failure specimens were from 57, 67, 57, 67, 49, 64, 50, 28, and 62 years old patients diagnosed with heart failure.
Recruitment	The de-identified specimens were obtained from Columbia University biobank and the NIH neuro-Biobank by searching in these database using the following key words: Specimens: Hippocampus, clinical diagnosis: Heart failure. Only frozen specimens showing absence of known neurodegenerative diseases (Alzheimer's, parkinson, huntington's diseases...) were selected for this study.
Ethics oversight	IRB Committee at Columbia University

Note that full information on the approval of the study protocol must also be provided in the manuscript.

Field-specific reporting

Please select the one below that is the best fit for your research. If you are not sure, read the appropriate sections before making your selection.

☒ Life sciences ☐ Behavioural & social sciences ☐ Ecological, evolutionary & environmental sciences

For a reference copy of the document with all sections, see nature.com/documents/nr-reporting-summary-flat.pdf

Life sciences study design

All studies must disclose on these points even when the disclosure is negative.

Sample size	No sample size calculation was made for the human biopsies due to the unavailability of these tissues. All obtained samples were processed and not data were excluded.<br>For the non human experiments the sample size was determined based on our previous experience using a power analysis of 0.8, an effect size of 0.9, a confidence (type I error) of 0.05 and the standard deviation in behavioral assays associated with these experiments. The power analysis was performed by G*power 3.1 software.
Data exclusions	No data were
Replication	Animal were randomized before attributed to each groups. All experiments were replicated at least 3 times.
Randomization	animal were randomized and assigned to one of the described groups. Cell based experiments were randomly assigned to one of the experimental group. Randomization for in-vitro and biochemical analysis of the samples was not relevant as the tissues were collected from the previously assigned groups. The order in which the experiments were performed was random.
Blinding	All experiments were double blinded by an independent investigator.

Reporting for specific materials, systems and methods

We require information from authors about some types of materials, experimental systems and methods used in many studies. Here, indicate whether each material, system or method listed is relevant to your study. If you are not sure if a list item applies to your research, read the appropriate section before selecting a response.

Materials & experimental systems

n/a	Involved in the study
☐	☒ Antibodies
☒	☐ Eukaryotic cell lines
☒	☐ Palaeontology and archaeology
☐	☒ Animals and other organisms
☒	☐ Clinical data
☒	☐ Dual use research of concern

Methods

n/a	Involved in the study
☒	☐ ChIP-seq
☒	☐ Flow cytometry
☒	☐ MRI-based neuroimaging

Antibodies

Antibodies used

Primary anibodies:
RyR2 Custom made: Acta Neuropathologica volume 134, pages749–767 (2017) 1/2500

pSer2808 Custom made. Circ Res. 2004;94(6): e61–e70. 1/1000

DNP Millipore Oxyblot (S7150).
Lot. 3249659
Validated by Western blot of derivatized samples 1/1000

Cys-NO ABM Y061263
Lot. AP10387
Validated by Western blot of at 1:0,000 using nitrosylated Cysteine–BSA as control. 1/1000

Calstabin2 Custom. JBC. 267 (14):9474-9477 (1992). 1/2500

Snap25 Thermofisher, MA5 17609
Lot. WD 3256763
Validated by western blot of PC-12 cell lines 1/1000

Vamp8 Abnova, H00008673-B01P
WD3257113
Validate by Western blot of VAMP transfected Cell Lines 1/1000

Syt2 Abcam. Ab181123
Lot. GR164541
Validated by Western blot of rat and mouse brain tissue lysate 1/1000

Cplx3 Thermofisher, PA5-24148
Lot. WD3256486
Validated by Western blot analysis in mouse liver tissue lysate 1/1000

GAPDH Thermofisher, PA1987
Lot. XJ358966
Validated by Western Blot in tissue extract of Ms Brain 1/5000

p-AMPK Thermofisher, PA5-104982
Lot. VJ3103601
Validated by Western Blot of H202 treated EC304 Cells. 1/1000

AMPK Abcam, ab207442
Lot. GR300197
Validated by Western Blot of Human skeletal muscle lysate 1/1000

p-GSK3β (T216) Abcam, ab75745
Lot. 1010539
Validated by Western blot of 293 cell extracts treated with insulin or with a PKC activator. (phorbol 12-myristate 13-acetate. PMA). 1/1000

GSK3β Abcam, ab32391
Lot. 1024397
Validated by Western blot of A431 cell lysate as well as wild type HAP1 whole cell lysate and GSK3β knockout HAP1 whole cell lysate. 1/1000

p-Tau (S199) Thermofisher, 44-734G
Lot. 2285802
Validated by Western blot of untreated human recombinant Tau or treated with GSK-3β. The antibody has been used in several

manuscripts for Western blots. 1/1000

p-Tau (S202/T205) Abcam, ab210703
Lot. GR3256698
Validated by Western blot of human brain tissue lysate. 1/1000

p-Tau (S262) Thermofisher, 44-750G
Lot. 2548898
Validated by Western blot of mouse brain, rat brain, and mouse kidney lysate 1/1000

Tau Thermofisher, PA5-27287
Lot. WA3171630
Validated by Western blot of mouse and rat brain lysates 1/1000

CDK5 Thermofisher, AHZ0492
Lot. VJ3096132
Validated by Western blot of cell lines including CF7, Jurkat, PC-3, MDA-MB-231, A549, HeLa and HT-29. And with HEK (+/- CD5 ko). 1/1000

P25 Thermofisher, PA5-57726
Lot. XF3609058A
Validated by immunofluorescent staining of human cell line A549 1/1000

APP Thermofisher, 14-9749-82
Lot. 2458748
Validated by Western Blot of mice and rat brain lysate 1/1000

BACE1 Abcam, ab183612
Lot. GR3240345
Validated by Western Blotting of Mouse hippocampus lysate 1/1000

B-CTF Millipore, MABN381
Validated by Western Blotting in DAPT treated HEK293 cell lysate. 1/1000

TGF-b1 Abcam, ab215715
Lot. GR3412442
Validated by Western Blot of Wild-type A549, K562 and SH-SY5Y whole cell lysates 1/1000

p-Smad3 Abcam, ab52903
Lot. GR328135
Validated by Western Blot of HL-60 treated with TGF-ß cell lysates 1/1000

Smad3 Abcam, ab40854
Lot. GR3255567
Validated by Western Blot of Jurkat whole cell lysates 1/1000

Nox2 Thermofisher, PA5-79118
Lot. YA3804004
Validated by Western Blot of mice and rat thymus tissue and brain lysate 1/1000

Secondary antibodies
IRDye® 800CW Goat anti-Rabbit IgG

IRDye® 800CW Goat anti-mouse IgG

Validation

All commercial antibodies were validated by their manufacturers and were titrated in the lab to determine optimal concentration for experimentation with our tissues. All the home made antibodies were validated in previous publications from our lab and others and the references are cited in the antibodies section in the extended method section, in the supplementary information document.

Animals and other research organisms

Policy information about studies involving animals; ARRIVE guidelines recommended for reporting animal research, and Sex and Gender in Research

Laboratory animals

The study used C57BL/6 mice purchased from Jackson laboratory
RyR2-S2808A, available in the Marks laboratory (6months old)
RyR2-S2808D available in the Marks laboratory (6months old)
RyR1-S2844D available in the Marks laboratory (6months old)

Wild animals

he study did not involve wild animals

Reporting on sex	Experiments were performed in male animals. We chose to use males in this study to avoid interference of female hormonal cycles mainly with the behavior performance as previously reported (See PMID: 23737953& 30689543)
Field-collected samples	The study did not involve samples collected from the field
Ethics oversight	All the animals were maintained and studied according to protocols approved by the Institutional Animal Care and Use Committee of Columbia University (reference no. AC-AAAC5453).

Note that full information on the approval of the study protocol must also be provided in the manuscript.
