# Supplementary material for: Heart failure-induced cognitive dysfunction is mediated by intracellular Ca2+ leak through ryanodine receptor type 2
Source: Nat Neurosci. 2023 Jul 10;26(8):1365–78. doi: 10.1038/s41593-023-01377-6 (PMC10400432; doi:10.1038/s41593-023-01377-6)
Supplement: Source Data Fig. 1 — Unprocessed western blots and statistical source data. [file 41593_2023_1377_MOESM4_ESM.pdf]

Figure 1A

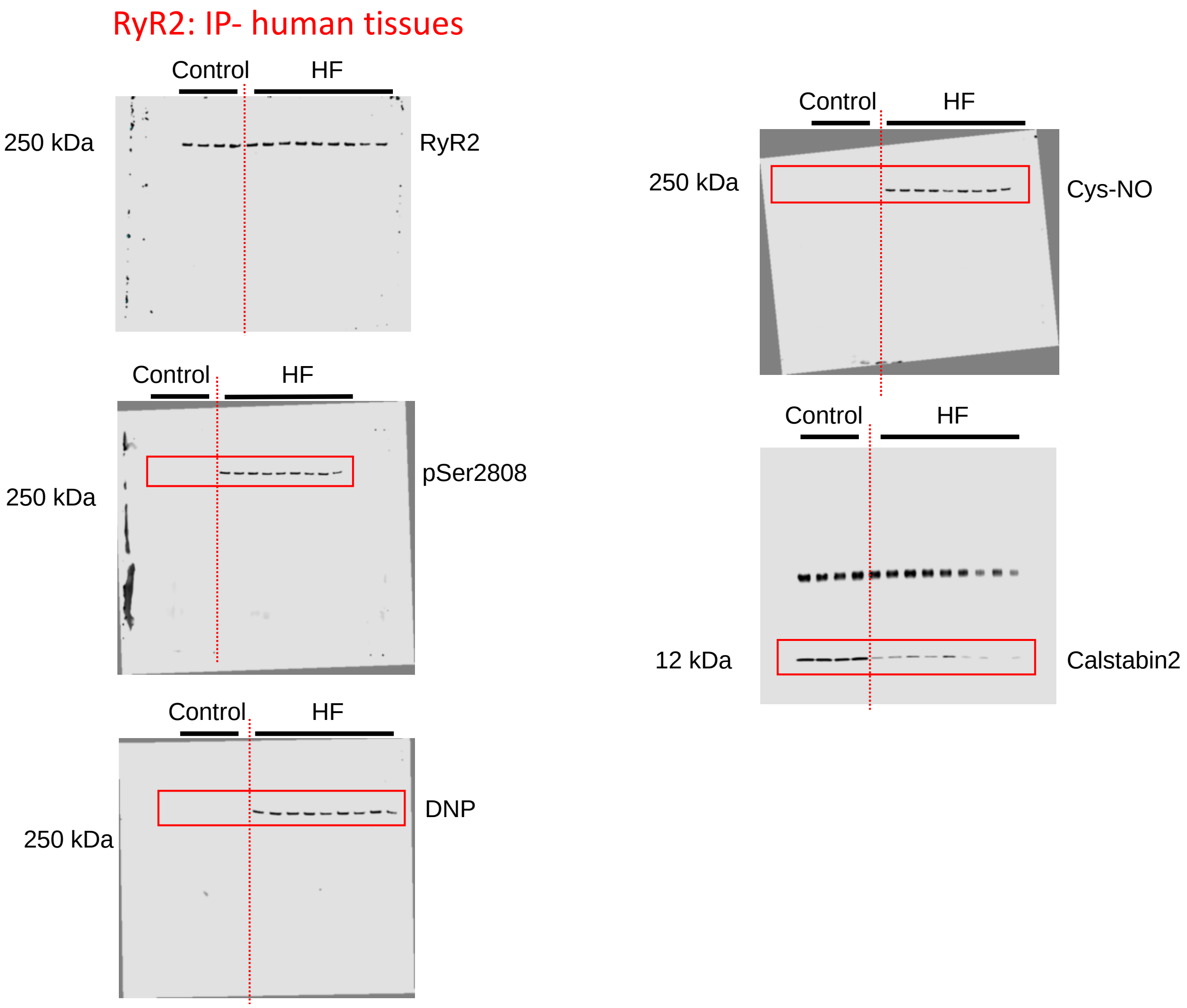
RyR2: IP- human tissues
Control
HF
250 kDa
RyR2
Control
HF
250 kDa
pSer2808
Control
HF
250 kDa
DNP
Control
HF
250 kDa
Cys-NO
Control
HF
12 kDa
Calstabin2

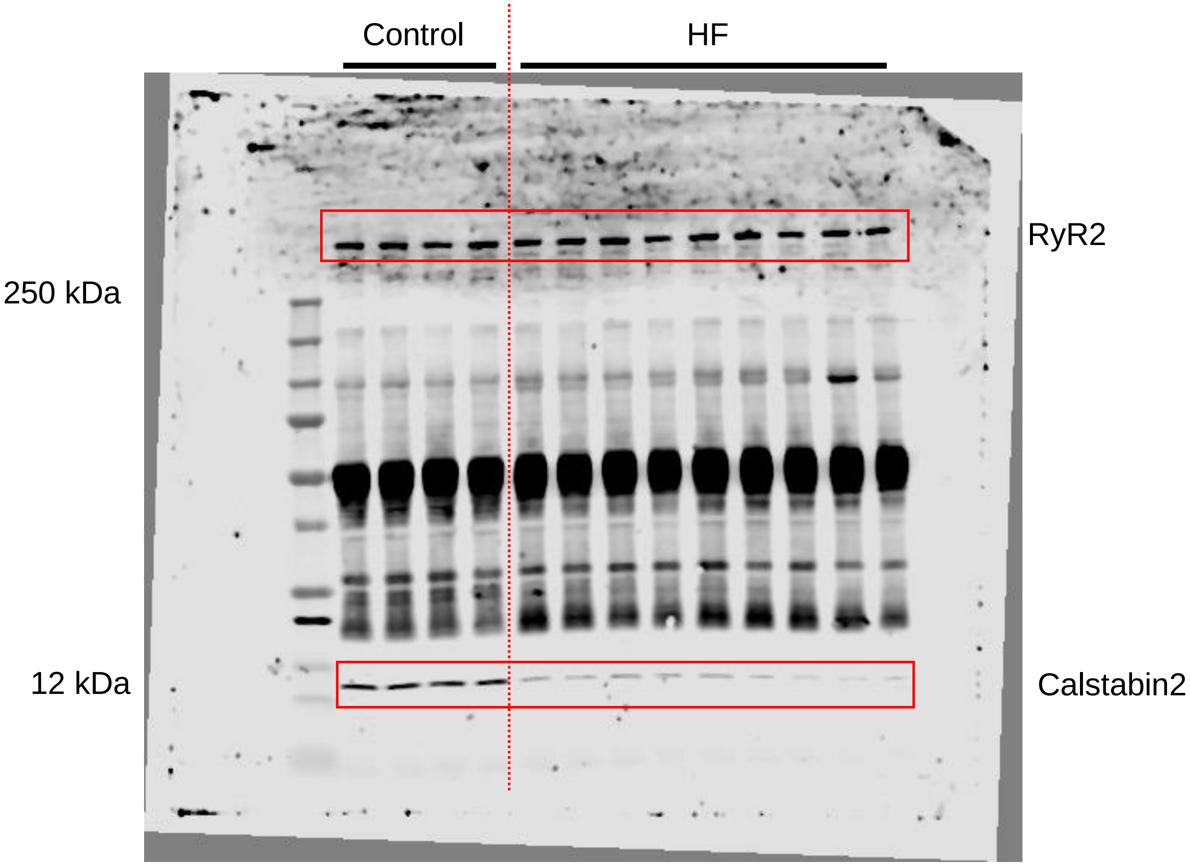

Loading Control gel showing even IgG and Total RyR2

Figure 1B

	Control				Patient								
**pSer2808**	0.1	0	0.1	0.2	3.5	3.6	3.8	3.7	3.1	3.6	2.9	3.7	3
**DNP**	0	0	0.1	0.1	3.5	3.8	3.7	3.9	3.7	3.5	2.9	3	2.8
**Cys-NO**	0.1	0	0	0.1	3.5	3.8	3.5	3.6	2.7	3.7	3.2	3.5	3.3
**Calstabin2**	4	3.8	3.6	3.9	0.5	0.6	0.7	0.5	0.8	0.3	0.5	0.2	0.3

Figure 1C_D

Open probability

Control	Patient
0.007	0.189
0.022	0.224
0.012	0.102
0.018	0.32
0.009	0.17
	0.154
	0.237
	0.186
	0.1371

Open Time (ms)

Control	Patient
2.1	18.6
3.1	21.3
1.7	12.9
2.1	15.7
1.6	27.6
	31.2
	17.4
	12.3
	9.36

Close Time (ms)

Control	Patient
543.6	72.1
387.6	38.3
698.3	84.2
487.2	44.7
458.1	55.6
	42.5
	67.3
	71.2
	53.6

Figure 1E

Time	Control 1				HF								
0	100	98	100	100	100	100	98	99	100	100	100	100	100
5	100	100	100	99	100	99	97	100	100	100	99	99	98
10	100	99	99	98	100	98	98	99	99	98	100	99	99
15	98	100	98	99	98	97	99	98	98	99	99	98	98
20	101	99	98	100	98	99	100	100	97	99	99	100	99
25	98	100	99	100	99	100	99	101	99	100	99	100	100
30	98	99	99	98	100	99	97	99	100	98	99	99	99
35	100	98	97	99	98	99	96	99	99	99	100	98	98
40	99	100	101	100	99	100	99	100	98	100	99	100	100
45	100	101	100	100	100	98	97	99	100	99	100	99	100
50	99	99	100	99	100	99	97	99	99	98	97	100	99
55	80	83	78	81	74	67	77	73	75	77	70	73	73
60	52	49	45	48	47	43	51	43	47	50	40	44	44
65	33	36	30	36	34	32	37	32	40	43	33	37	35
70	34	32	30	33	33	32	35	32	48	36	30	35	33
75	35	33	33	34	33	30	35	35	40	38	32	38	34
80	33	35	34	33	32	33	36	34	39	36	34	39	35
85	34	36	35	36	33	35	37	35	38	34	33	38	33
90	35	35	32	35	35	37	39	34	40	35	33	36	36
95	34	32	26	33	33	39	41	34	38	36	34	37	35
100	35	36	35	34	34	34	37	35	40	36	35	38	34
105	42	38	34	41	68	64	73	70	70	75	63	73	60
110	43	40	31	43	69	60	71	75	72	75	65	75	62
115	42	38	37	41	73	64	70	73	70	77	64	73	63
120	41	35	34	40	70	66	67	72	72	74	65	74	66
125	40	38	35	35	71	60	65	69	72	75	66	75	64
130	42	39	35	41	68	62	68	73	70	72	68	72	62
135	42	40	38	39	68	64	69	74	69	75	66	70	63
140	41	37	40	40	73	66	72	69	69	74	65	72	63
145	40	35	41	41	69	68	73	71	70	76	64	74	66
150	42	34	40	41	73	70	70	74	69	72	66	76	64
155	42	37	38	40	76	70	67	74	68	74	65	75	62
160	41	38	40	38	74	65	65	72	64	74	64	75	63
165	40	36	36	36	72	67	68	75	66	75	63	75	65

170	43	35	38	39	76	69	69	74	70	76	63	72	65
175	42	37	38	39	71	67	71	73	67	72	65	73	64
180	41	41	35	37	74	65	67	72	66	75	63	71	66
185	42	40	32	40	73	67	66	75	68	73	61	74	66
190	43	40	32	38	73	69	68	74	70	75	63	75	64
195	43	42	35	35	68	71	64	70	69	72	64	76	65
200	42	38	34	36	71	69	68	74	69	72	65	77	65
205	41	39	35	38	69	67	70	71	71	73	61	74	63
210	40	37	37	39	69	69	72	71	70	74	62	74	64
215	43	39	38	40	72	65	74	72	70	70	62	72	65
220	42	40	38	40	68	67	71	74	69	73	64	73	64
225	41	42	39	39	70	69	71	72	68	71	64	74	65
230	42	40	41	39	68	67	67	70	67	73	65	75	66
235	43	40	40	40	69	69	65	71	68	74	63	75	65
240	43	39	40	40	70	71	70	73	69	74	64	76	64
245	42	38	37	41	70	67	69	73	69	70	63	75	65
250	41	40	36	40	68	65	69	72	68	72	66	73	63
255	42	42	38	39	69	65	67	70	68	75	68	74	64
260	43	40	36	38	69	68	65	70	70	73	63	75	65
265	43	38	35	39	71	66	68	72	69	73	65	77	64
270	42	37	34	40	68	69	70	74	69	71	66	74	63
275	41	35	36	36	70	71	68	77	70	75	64	72	66
280	41	38	35	39	71	69	71	75	71	74	66	74	63
285	43	40	36	40	71	67	67	73	69	72	63	75	65
290	44	40	39	39	70	65	70	72	69	74	66	73	64
295	42	37	35	38	69	65	68	70	70	75	64	77	66
300	41	37	36	40	70	67	72	67	69	73	64	75	65
