# Supplementary material for: Heart failure-induced cognitive dysfunction is mediated by intracellular Ca2+ leak through ryanodine receptor type 2
Source: Nat Neurosci. 2023 Jul 10;26(8):1365–78. doi: 10.1038/s41593-023-01377-6 (PMC10400432; doi:10.1038/s41593-023-01377-6)
Supplement: Source Data Fig. 2 — Statistical source data. [file 41593_2023_1377_MOESM5_ESM.pdf]

Figure 2A

SHAM	SHAM	MI	MI	MI+ARM036	MI+ARM036	MI+S107	MI+S107	MI+Propranolol	MI+Propranolol	MI+SD208	MI+SD208
0.078152753	0.36902951	0.2730486	0.31919976	0.10743596	0.12154696	0.09329446	0.25	0.1050037	0.29774957	0.22101252	0.28927752
0.164774228	0.162427133	0.09388265	0.38927012	0.1124319	0.26348315	0.10331288	0.17986366	0.06232295	0.64413592	0.04325462	0.23155007
0.118839836	0.306206089	0.14053216	0.35226586	0.16824645	0.21498371	0.12358134	0.21034204	0.05595985	0.23835767	0.18949045	0.23553833
0.186933333	0.60206995	0.10910886	0.73590734	0.14665304	0.25768586	0.17756764	0.34618861	0.07300672	0.09261084	0.12390707	0.29477935
0.1367942	0.2612108	0.17087783	0.52422907	0.15611269	0.09625276	0.09702584	0.18639599	0.09470069	0.12810852	0.29976785	0.36948529
0.0536791	0.140829	0.17026316	0.23018043	0.25735913	0.18294615	0.08095124	0.06034075	0.06057035	0.1319797	0.05338664	0.14741138
0.1592709	0.2998267	0.19082958	0.47783251	0.102457	0.16671008	0.14636055	0.19142176	0.06888361	0.21149177	0.10592283	0.14617593
0.07219443	0.4241633	0.14344158	0.16237315	0.13948718	0.06586117	0.17240473	0.32900177	0.14155251	0.2145749	0.13073048	0.13301241
0.06736243	0.2062726	0.09038042	0.16974266	0.17645496	0.16369279	0.05810312	0.19099141	0.12895133	0.22783083	0.17810561	0.11281653
0.08985227	0.06427009	0.15325474	0.25411896	0.22855675	0.34012225	0.3063976	0.2391505	0.34892086	0.59340659	0.14773959	0.52331175
0.2055455	0.3504389	0.34208172	0.30487805	0.13018797	0.16957344	0.11409458	0.2551652			0.10706431	0.41267717
0.08218601	0.424276	0.18796199	0.21055466	0.12958061	0.14481778	0.15362302	0.1433732			0.1084296	0.15923091
0.01288616	0.1216351	0.30890052	0.25628141	0.25314368	0.30000433	0.14162618	0.16528731			0.09547023	0.27337095
		0.28132118	0.4720314	0.10897469	0.19659503	0.07055964	0.16397126			0.20010734	0.33949129
		0.082642	0.0649	0.06163033	0.05179344	0.11447137	0.26651969			0.15540699	0.27531139
		0.160841	0.160766	0.07064482	0.11510691	0.096959	0.11118			0.1013712	0.10539283
		0.702772	0.351554	0.2158876	0.27864657	0.094957	0.337693			0.18110236	0.29570976
		0.161215	0.153181	0.13915693	0.24411846	0.171722	0.276234			0.09547004	0.25770308
		0.292918	0.097293	0.222826	0.25139	0.131151	0.342783				
		0.241379	0.22758	0.106059	0.186944	0.232033	0.363946				
		0.181723	0.12881	0.212775	0.183977	0.006599	0.057641				
		0.417658	0.20773	0.181257	0.33363	0.172333	0.257159				
				0.114689	0.056028	0.198003	0.284063				
						0.213429	0.265378				

Figure 2B

SHAM	MI	MI+ARM036	MI+S107	MI+Propranolol	MI+SD208
0.25078111	0.06352887	0.08939343	0.03411951	0.14145382	0
0.08410064	0.20467164	0.26179841	0	0.05105421	0
0.10900206	0.06174951	0.15392979	0.11582657	0.08884476	0.14683215
1.23E-03	0	0.07414509	0.13991184	0.14873719	0.00932493
0.25000124	0.07884214	0.17219987	0.0430564	0.13106875	0.13436041
0.072355	0.05486215	0.05829592	0.07847283	0	0.17894303
0.192764	0.02619784	0.14783798	0.08859764	0	0.02606011
0.362629	0.65024185	0.36640511	0.13802069	0.00939505	0
0.199843	0.15901248	0.18596066	0.12173253	0	0.15671923
0.035461	0.09083368	0.15402528	0.02140316	0	2.96E-03
0.12267	0	0.2339688	0.18405183		0.02914188
0.189021	0.03314362	0.383122	0.06356775		0.16811909
0.163814	0.14232337	0.313533	0.14847693		0.072528
0.059151	0.18280298	0.180582	0.22566245		0.08535485
	0.5	0.355315	0.08821223		0.02457061
	0.333333	0.288053	0.050476		0.07968129
	0.4	0.516214	0.213781		0.07655709
	0.285714	0.231824	0.108173		0.0106843
	0.25		0.049799		
	0.375		0.143213		
	0.25		0.143424		
	0.909091		0.046266		
			0.185823		

Figure 2C

SHAM	MI	MI+ARM036	MI+S107	MI+Propranolol	MI+SD208
79.04759	-17.543937	29.5129785	28.0899423	86.2068668	20.8791526
30.031022	23.8807223	39.495876	55.1601265	40.3227854	25.2668432
31.9034368	0	21.014435	68.0555146	24.2235177	9.03956039
42.97687	3.84607067	7.26246065	47.5285576	55.1866256	55.1724769
63.72824	10.0775299	25.6317781	51.9685589	62.7705778	76.2068988
65.80173	42.0494369	21.9047551	62.9167274	28.5714286	-1.3953942
69.28571	27.6785734	20.0000339	22.1975883	58.3814311	42.2680572
33.77136	44.8087618	-2.8984064	25.9447524	32.6530612	71.4285298
46.13982	40.9691087	3.48835267	65.4676505	26.8817204	-12.635414
52.87958	-4.0650407	7.77204085	54.8192671	50.9202454	59.1131916
35.37415	-27.823691	28.1898956	83.8863076		30.0000625
25.92755	29.4797688	34.8571461	68.0672092		56.9619573
	37.28223	3.4483617	28.4999366		15.2071265
	15.51724	31.4775161	29		35.0516061
	16.99077	6.007067	35.2		26.7658621
	14.27481	17.31917	60		10.3448276
	13.5689	9.107067	43.41085		77.8761062
	0	-5.307415	56.00371		
	19.8566	13.86053	20.25641		
	28.16901	23.84717	29.78157		
	11.30621	0	29.06158		
		15.60694	59.84203		
		9.915014			

Figure 2D

	SHAM			MI			MI+ARM036			MI+S107			MI+Propranolol			MI+SD208		
	Mean	SD	N	Mean	SD	N	Mean	SD	N	Mean	SD	N	Mean	SD	N	Mean	SD	N
Day1	47.16	9.42	22	44.95	7.51	20	48.47	9.72	19	49.52	6.57	19	44.23	10.98	14	43.44	14.32	19
Day2	33.45	7.23	22	40.68	8.68	20	37.5	7.28	19	31.57	4.38	19	27.14	11.48	14	26.39	12.16	19
Day3	27	8.04	22	31.75	8.31	20	31.95	10.64	19	21.95	4.17	19	24.92	12.22	14	24.38	11.71	19
Day4	21.09	8.08	22	30.31	6.7	20	29.44	14.43	19	20.55	7.76	19	17.91	11.69	14	15.57	13.97	19

Figure 2E

SHAM	MI	MI+ARM036	MI+S107	MI+Propranolol	MI+SD208
34.2771	23.2268	12.415	18.975	20.5068	18.2624
37.5602	26.9121	14.6582	31.5596	38.6084	18.983
35.1638	27.7948	23.3892	38.7671	29.6332	24.4295
28.9961	13.9374	1.92229	24.59	21.8683	35.6431
35.4834	27.7913	19.7034	25.3823	28.9163	43.0129
38.769	20.3457	27.153	18.5028	19.4632	31.5596
29.7176	19.4628	8.08935	28.1162	24.9087	30.5989
14.6582	20.7457	13.7739	24.81	19.3844	14.417
21.7062	20.2641	28.516	25.81	16.0197	20.4248
6.88805	20.0249	9.76	18.74	34.4442	24.9097
16.5791	13.04	25.76	35.04	23.8659	40.3689
28.1101	21.12	11.37	20.6	22.08	26.9129
24.3447	16.47	12.31	21.66	35.12	23.1495
22.32	15.73	13.78	20.98	29.76	32.7553
24.17	10.56	13.34	34.51		22.1879
23.23	5	10	17.65		27.12
23.71	9.6	18.31			29.52
17.72	12.97	16.61			
23.01	10.02				
23.9	11.46				
26.35					
21.99					

Figure 2F

SHAM	MI	MI+ARM036	MI+S107	MI+Propranolol	MI+SD208
3	4	0	5	2	2
3	4	1	3	2	2
4	1	1	5	9	5
4	3	0	2	3	7
2	3	2	4	2	5
5	2	5	2	2	7
7	2	1	4	1	11
7	0	5	5	2	2
3	4	2	5	3	2
3	2	1	4	5	3
2	1	5	4	3	5
5	4	1	7	4	3
5	1	2	4	7	4
4	0	1	3	4	5
4	2	2	4		3
7	3	0	6		6
5	2	1			5
4	1	4			
8	4				
6	5				
