# Supplementary material for: Heart failure-induced cognitive dysfunction is mediated by intracellular Ca2+ leak through ryanodine receptor type 2
Source: Nat Neurosci. 2023 Jul 10;26(8):1365–78. doi: 10.1038/s41593-023-01377-6 (PMC10400432; doi:10.1038/s41593-023-01377-6)
Supplement: Source Data Fig. 3 — Unprocessed western blots and statistical source data. [file 41593_2023_1377_MOESM6_ESM.pdf]

Figure 3B

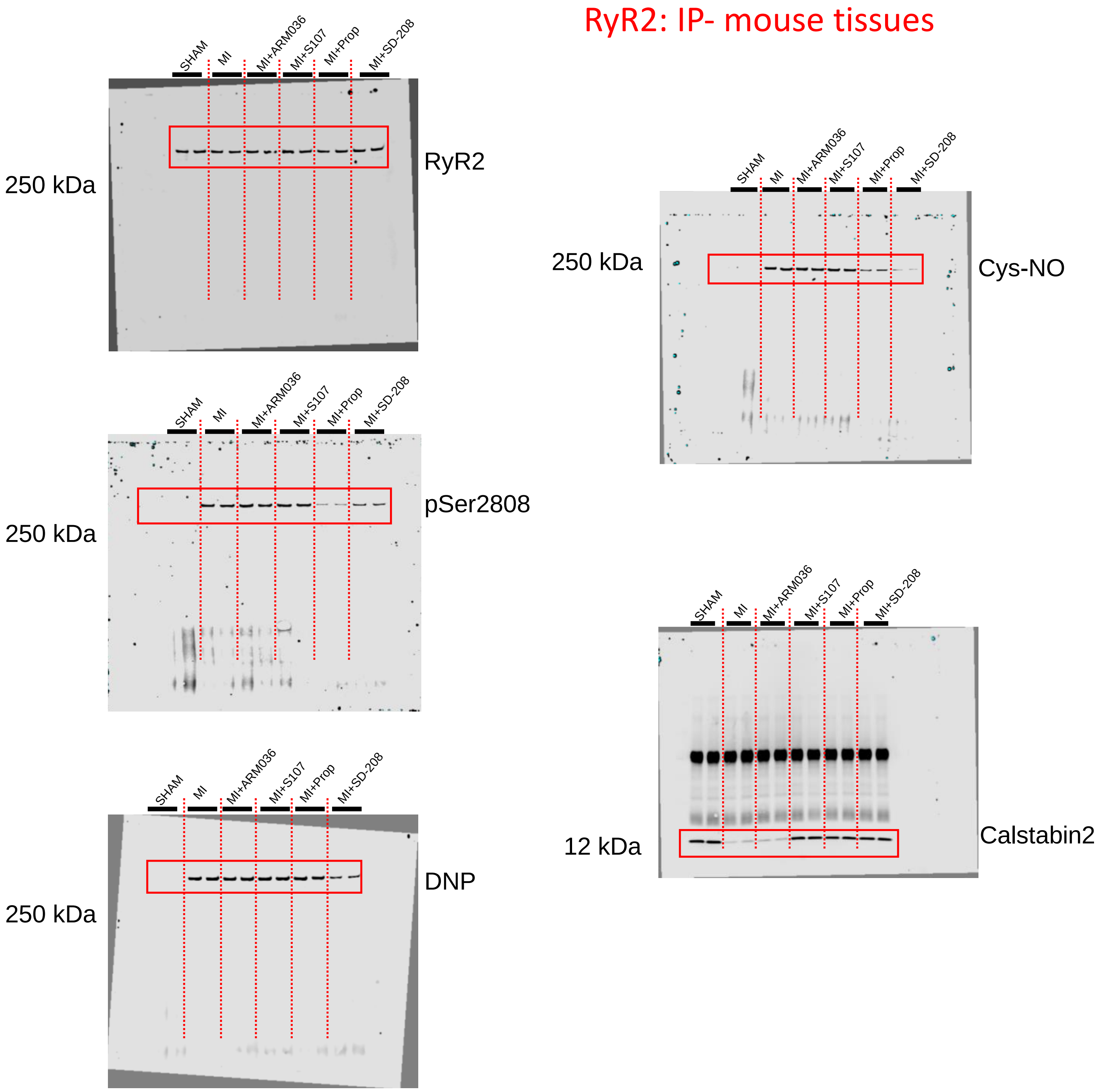
RyR2: IP- mouse tissues
SHAM
MI
MI+ARM036
MI+S107
MI+Prop
MI+SD-208
RyR2
250 kDa
pSer2808
250 kDa
DNP
250 kDa
Cys-NO
250 kDa
Calstabin2
12 kDa

RyR2: IP- mouse tissues

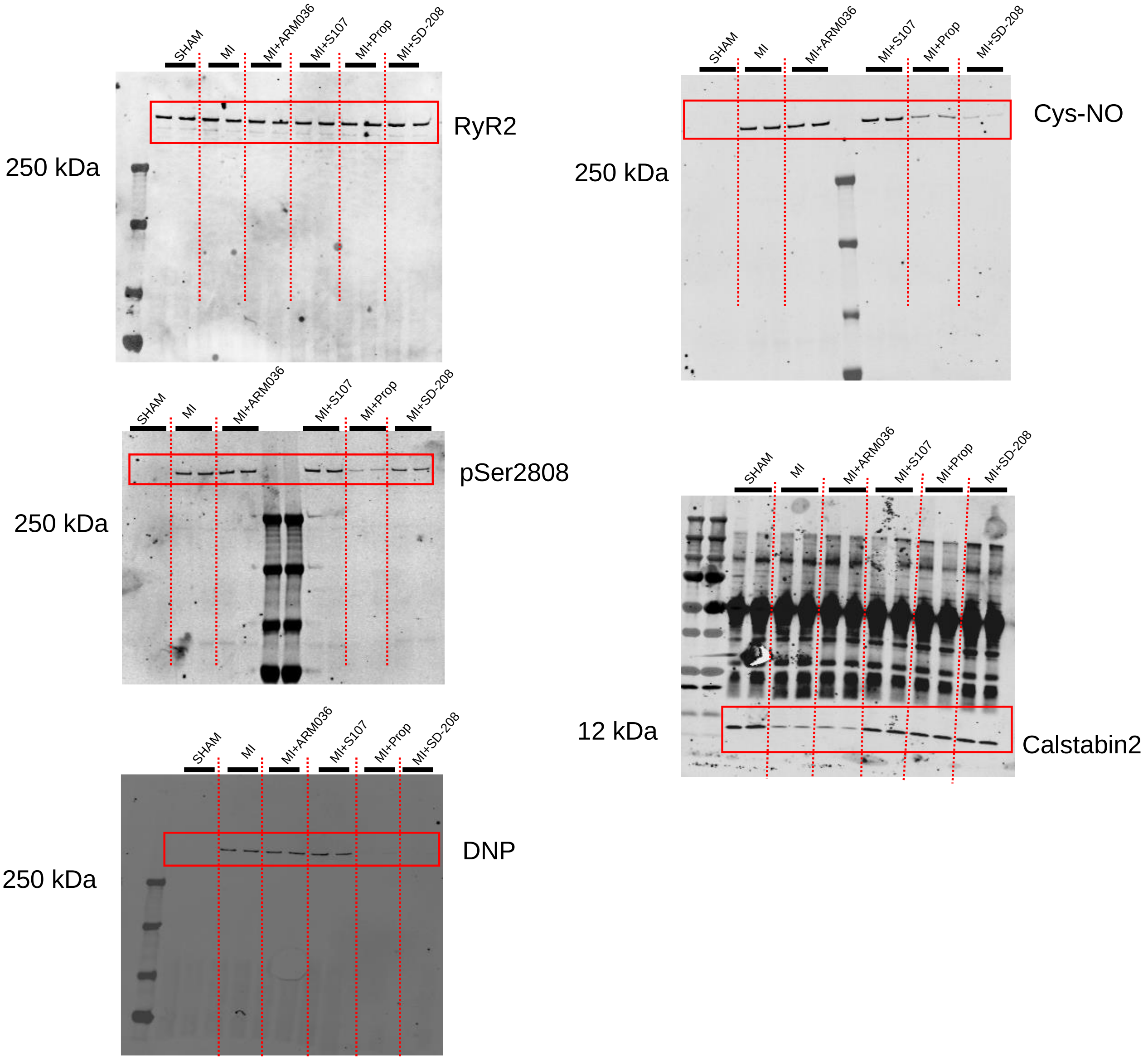

RyR2: IP- mouse tissues

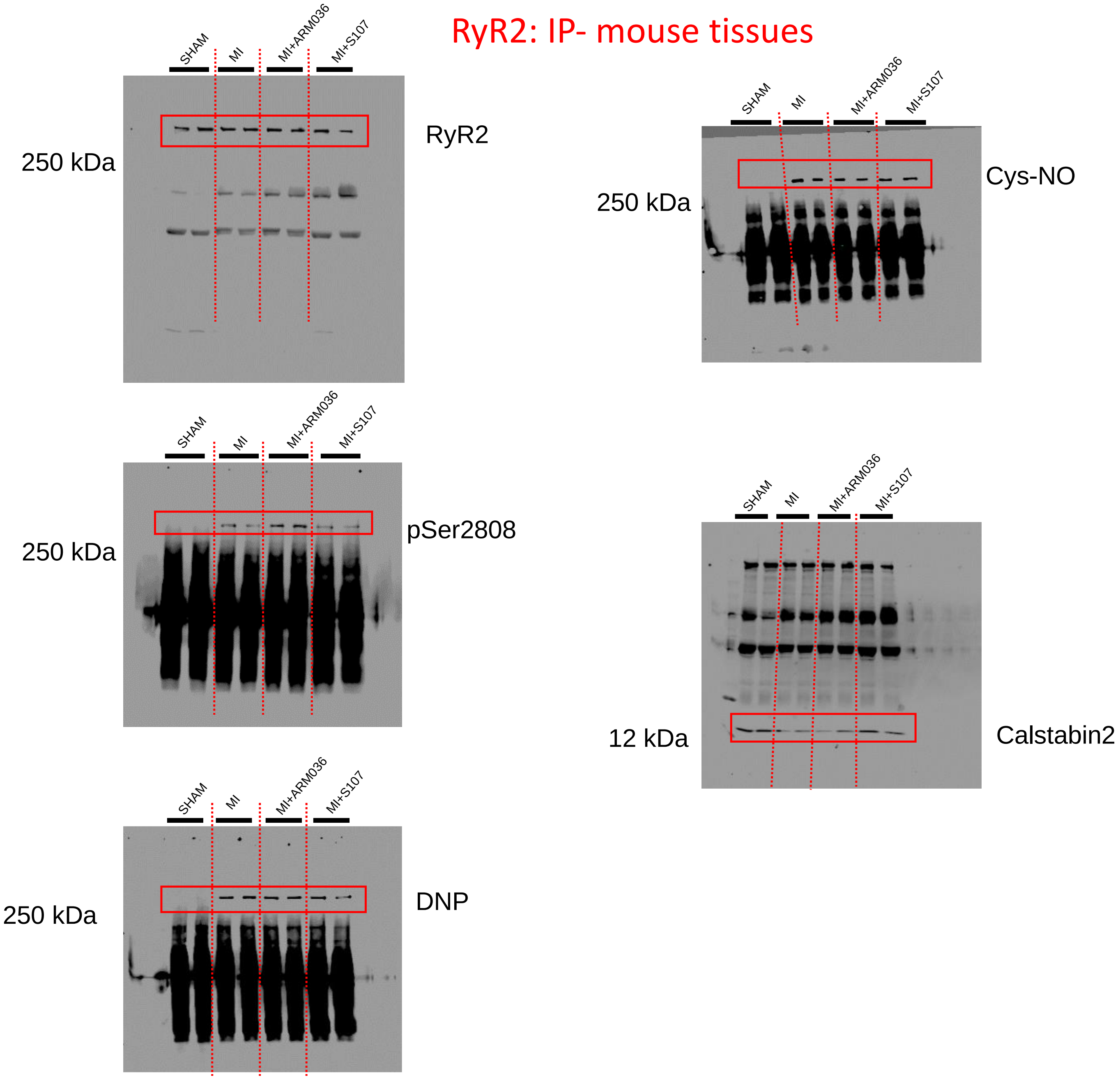

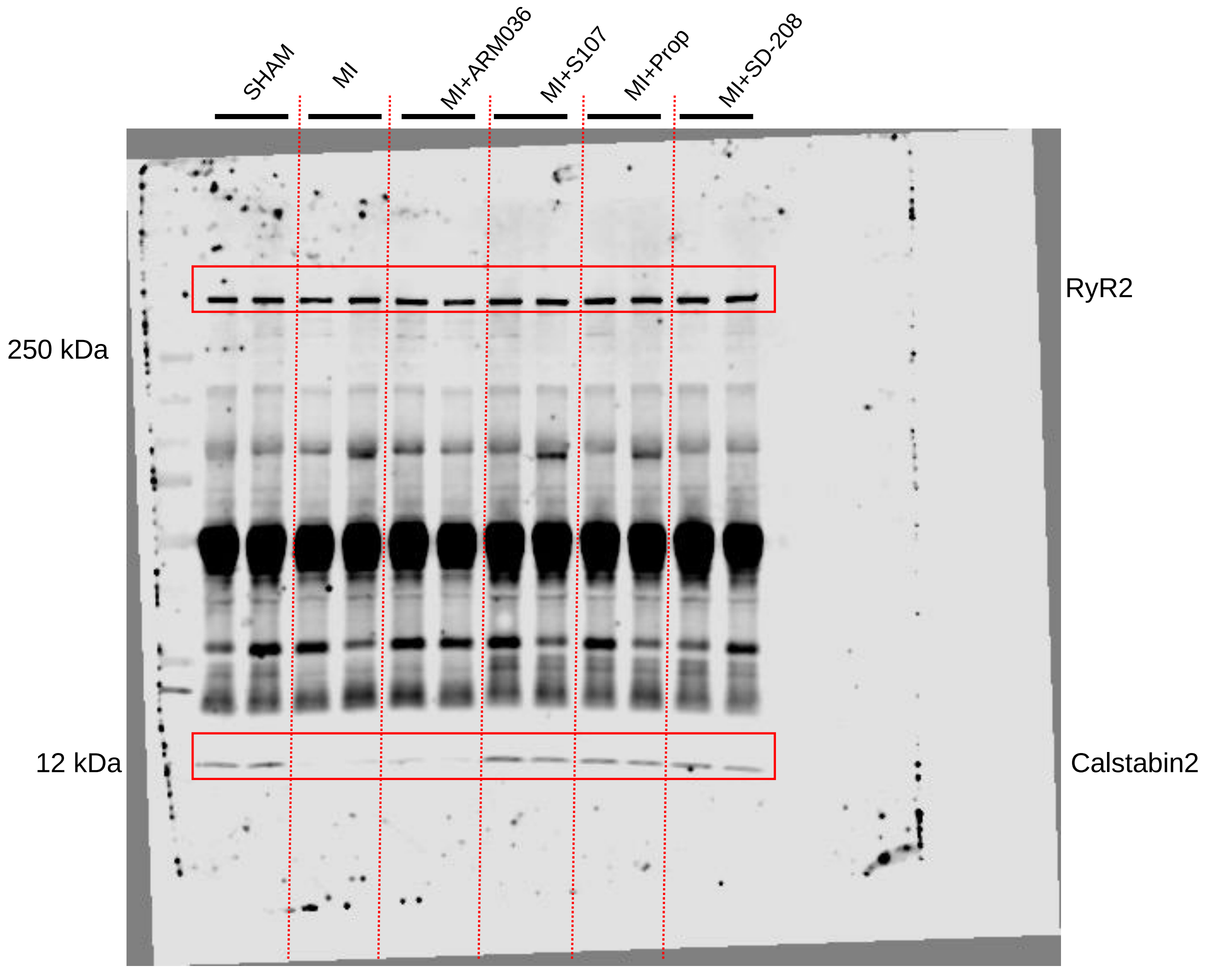

Loading Control gel showing even IgG and Total RyR2

Figure 3C

	pSer2808	DNP	Cys-NO	Calstabin2
SHAM	0.1	0.5	0.1	4.2
SHAM	0.2	0.4	0.2	3.8
SHAM	0.15	0.2	0.45	4.3
SHAM	0.9	0.1	0.09	3.75
SHAM	0.1	0	0.1	3.7
SHAM	0.1	0.1	0.1	4
MI	4.5	4.1	3.2	0.9
MI	4.2	3.8	3.6	1.1
MI	3.8	3.2	4.2	0.8
MI	3.5	4	4	1.5
MI	3.2	3.7	3.5	0.3
MI	3.3	3.9	3.7	0.5
MI+ARM036	4	4.5	3.85	0.85
MI+ARM036	3.9	3.6	3.9	1
MI+ARM036	3.7	3.7	3.7	1.3
MI+ARM036	4.1	4	4.15	0.5
MI+ARM036	3.8	3.8	3.8	0.5
MI+ARM036	3.1	4	3.6	0.4
MI+S107	4	3.9	4	4
MI+S107	3.6	4.12	4.2	4.3
MI+S107	3.55	4.3	3.8	3.6
MI+S107	3.9	3.6	3.75	3.75
MI+S107	3.22	3.9	3.7	3.6
MI+S107	3	4	3.5	3.7
MI+prop	0.8	3.8	1.1	3.6
MI+prop	0.7	3.6	1.3	3.8
MI+prop	0.6	4	0.9	3.9
MI+prop	0.9	3.5	1.4	3.4
MI+SD-208	1.6	1.8	0.5	4
MI+SD-209	1.8	2	0.3	3.6
MI+SD-210	1.8	2.2	0.4	3.7
MI+SD-211	1.5	1.7	0.4	3.9

Figure 3D-E

Open probability

SHAM	MI	MI+ARM36	MI+S107	MI+Prop	MI+SD-208

0.00882	0.01889	0.03875	0.00239	0.01433	0.05943
0.00068	0.22295	0.06897	0.0176	0.02337	0.02863
0.01	0.174	0.20064	0.00112	0.02199	0.0312
0.007	0.256	0.28667	0.02	0.0124	0.0401
0.013	0.211	0.26912	0.015	0.0193	0.0331
		0.175			
		0.181			

Open time

SHAM	MI	MI+ARM36	MI+S107	MI+Prop	MI+SD-208
2.62276	7.52974	24.1606	3.38772	3.76655	7.0553
4.04	19.5887	18.3	2.75187	2.85528	8.21224
3.52	15.4	32.2072	4.3	5.0806	4.301
2.11	21.1	15.6	5.1	3.214	3.321
1.81	6.5	7.43365		2.874	3.847
4.12		8.9			

Close time

SHAM	MI	MI+ARM36	MI+S107	MI+Prop	MI+SD-208
167.665	105.746	31	1130.63	244.107	385.271
345	102.218	45	1657.6	214.33	226.639
673	67.3	121.45	521.2	210.559	123.562
542	31.6	18.5	415	98.9657	156.247
532	63.7	19.6775	321	186.3	87.34
1500		23.4	231		

Figure 3F-G

SHAM	MI	MI+ARM36	MI+S107	MI+Prop	MI+SD-208
11.825	46.141	42.437	18.951	31.421	28.009
18.126	51.729	49.393	17.162	28.073	29.55
9.761	44.873	44.329	25.599	27.282	29.748
12.825	45.141	43.437	19.951		
17.126	50.729	42.394	16.162		
10.761	43.873	41.329	22.069		
