# Supplementary material for: Heart failure-induced cognitive dysfunction is mediated by intracellular Ca2+ leak through ryanodine receptor type 2
Source: Nat Neurosci. 2023 Jul 10;26(8):1365–78. doi: 10.1038/s41593-023-01377-6 (PMC10400432; doi:10.1038/s41593-023-01377-6)
Supplement: Source Data Fig. 4 — Statistical source data. [file 41593_2023_1377_MOESM7_ESM.pdf]

Figure 4B

SHAM

101.074	100.662	105.179	95.5166	97.512	97.7974	95.9173	93.0537	100.134	108.379	91.3242	97.9943	100.694
108.398	99.2439	100.398	97.4659	98.7159	103.084	86.9251	107.471	92.1228	100.853	98.1735	92.8367	87.0865
96.6797	96.4083	119.522	109.162	95.1043	79.2952	83.9277	95.675	98.1308	88.8108	89.0411	99.7135	76.2007
102.539	94.9905	83.6653	81.8713	89.0851	92.511	98.9147	99.6068	86.1148	99.3477	95.8904	91.1175	78.9222
106.934	96.4083	95.6175	103.314	89.0851	97.7974	94.4186	98.2962	96.1282	99.3477	79.9087	108.309	114.301
120.117	96.4083	88.4462	109.162	90.2889	111.013	89.9225	115.334	100.134	102.358	95.8904	91.1175	100.694
105.469	106.333	76.494	103.314	102.327	87.2247	85.4264	99.6068	108.144	106.874	89.0411	92.8367	68.0364
102.539	97.8261	71.7131	101.365	97.512	103.084	100.413	106.16	114.152	93.3266	91.3242	92.8367	92.5295
95.2148	96.4083	98.008	99.4152	97.512	84.5815	85.4264	99.6068	100.134	103.864	118.721	115.186	106.869
120.117	99.2439	95.6175	97.4659	98.7159	108.37	91.4212	95.675	114.152	94.8319	107.306	104.871	92.5295
106.934	96.4083	114.741	109.162	108.347	97.7974	97.416	93.0537	108.144	103.864	86.758	89.3983	100.694
106.934	97.8261	112.351	93.5672	102.327	124.229	97.416	100.917	102.136	99.3477	91.3242	91.1175	84.3651
102.539	102.079	78.8845	89.6686	107.143	103.084	101.912	95.675	128.171	94.8319	86.758	80.8023	76.2007
106.934	100.662	88.4462	120.858	96.3082	92.511	103.411	98.2962	102.136	102.358	100.457	96.2751	89.808
95.2148	94.9905	112.351	107.212	97.512	68.7225	107.907	102.228	102.136	93.3266	111.872	104.871	122.465
92.2852	97.8261	100.398	89.6686	107.143	113.656	95.9173	98.2962	98.1308	99.3477	91.3242	99.7135	97.9724
83.4961	103.497	98.008	93.5672	111.958	113.656	110.904	93.0537	106.142	97.8424	98.1735	101.433	92.5295
89.3555	96.4083	119.522	115.01	98.7159	100.441	107.907	98.2962	110.147	102.358	102.74	104.871	78.9222
106.934	94.9905	93.2271	105.263	102.327	108.37	115.401	94.3643	108.144	100.853	107.306	97.9943	109.609
108.398	99.2439	112.351	101.365	103.531	95.1542	104.91	99.6068	108.144	96.3372	100.457	113.467	114.301
86.4258	106.333	114.741	115.01	104.735	87.2247	101.912	102.228	96.1282	108.379	116.438	101.433	109.609
95.2148	99.2439	112.351	93.5672	93.9005	108.37	101.912	94.3643	88.1175	93.3266	109.589	99.7135	95.9078
89.3555	99.2439	83.6653	89.6686	101.124	124.229	110.904	93.0537	100.134	94.8319	102.74	108.309	112.349
84.9609	96.4083	98.008	77.9727	110.754	116.3	106.408	108.781	68.0908	93.3266	116.438	104.871	101.388
95.2148	102.079	88.4462	77.9727	110.754	103.084	118.398	98.2962	84.1121	100.853	91.3242	97.9943	112.349
96.6797	110.586	114.741	105.263	107.143	89.8678	94.4186	94.3643	90.1202	99.3477	105.023	104.871	106.869
96.6797	99.2439	107.57	97.4659	96.3082	97.7974	97.416	100.917	90.1202	103.864	105.023	99.7135	109.609
109.863	100.662	90.8367	107.212	96.3082	92.511	109.406	102.228	114.152	118.916	105.023	101.433	106.869
98.1445	109.168	121.912	97.4659	85.4735	105.727	95.9173	100.917	84.1121	94.8319	111.872	99.7135	109.609
89.3555	109.168	102.789	115.01	102.327	92.511	103.411	120.577	102.136	103.864	102.74	115.186	150.712
213.867	165.879	302.494	290.058	190.209	177.093	231.507	184.797	214.286	197.19	181.849	225.215	315.126

208.008	174.386	258.935	276.522	195.024	177.093	226.968	196.592	200.267	176.116	171.233	220.057	337.46
203.613	175.803	241.995	266.854	180.578	161.233	210.323	183.486	194.259	185.148	178.082	214.9	307.524
206.543	174.386	246.835	241.715	174.559	177.093	210.323	193.971	186.248	182.137	196.347	223.496	345.625
206.543	171.55	225.055	247.517	185.393	169.163	196.705	187.418	188.251	185.148	191.781	228.653	326.575
199.219	185.728	232.315	239.782	174.559	153.304	205.784	180.865	192.256	191.169	189.498	228.653	307.524
194.824	177.221	232.315	262.986	178.17	185.022	205.784	195.282	198.264	192.674	173.516	209.742	351.068
210.937	171.55	229.895	280.39	181.782	166.52	214.863	184.797	170.227	185.148	191.781	220.057	345.625
200.684	167.297	212.956	259.119	167.335	171.806	222.428	201.835	192.256	195.685	182.648	208.023	318.41
199.219	168.715	239.575	261.053	175.762	148.018	214.863	196.592	186.248	180.632	196.347	233.811	323.853
206.893	170.132	241.995	247.517	175.762	177.093	214.863	201.835	194.259	192.674	203.196	242.407	332.017
206.893	170.132	249.255	255.251	161.316	153.304	216.376	203.145	192.256	192.674	203.196	232.092	323.853
221.671	167.297	232.315	245.583	181.782	155.947	220.915	197.903	190.254	194.18	191.781	233.811	318.41
203.937	167.297	239.575	247.517	172.151	177.093	222.428	196.592	174.232	195.685	196.347	233.811	310.246
202.46	158.79	263.775	239.782	170.947	174.449	210.323	195.282	188.251	197.19	196.347	233.811	280.31
193.593	170.132	229.895	253.318	175.762	163.877	207.297	195.282	170.227	179.127	200.913	228.653	293.917
212.804	157.372	176.656	230.113	179.374	171.806	204.271	207.077	188.251	195.685	191.781	237.249	304.803
184.726	154.537	237.155	237.848	173.355	171.806	208.81	204.456	190.254	197.19	198.63	244.126	321.132
202.46	146.03	237.155	224.312	167.335	171.806	213.35	211.009	202.27	200.201	210.046	237.249	299.36
190.637	158.79	222.635	224.312	172.151	179.736	205.784	195.282	204.272	197.19	200.913	244.126	342.903
195.071	160.208	234.735	208.842	174.559	163.877	222.428	200.524	198.264	194.18	207.763	247.564	348.346
187.681	148.866	263.775	222.222	174.559	158.59	210.323	214.941	186.248	203.211	184.932	238.968	332.017
172.903	155.955	241.995	204.678	162.52	163.877	207.297	200.524	198.264	207.727	194.064	251.003	348.346
180.292	147.448	241.995	231.969	155.297	155.947	213.35	200.524	198.264	201.706	205.479	237.249	307.524
164.037	151.701	261.355	233.918	154.093	195.595	199.732	207.077	198.264	200.201	191.781	256.16	337.46
159.603	150.284	268.614	218.324	157.705	166.52	210.323	199.214	194.259	216.759	198.63	252.722	323.853
161.081	147.448	249.255	245.614	160.112	177.093	211.837	213.63	218.291	201.706	203.196	240.688	337.46
162.598	154.537	271.034	218.324	162.52	161.233	214.863	211.009	188.251	192.674	191.781	247.564	356.511
184.57	144.612	295.234	226.121	158.909	153.304	220.915	211.009	190.254	212.243	191.781	249.284	340.182

181.641	144.612	273.454	204.678	168.539	177.093	213.35	213.63	192.256	206.222	194.064	254.441	337.46
172.852	144.612	241.995	206.908	160.112	169.163	216.376	211.009	188.251	212.243	198.63	254.441	323.853
181.641	140.359	227.475	199.173	155.297	177.093	220.915	208.388	192.256	207.727	194.064	238.968	334.739
183.105	146.03	227.475	183.704	151.685	187.665	226.968	207.077	188.251	192.674	198.63	263.037	312.967
169.922	144.612	234.735	183.704	143.258	171.806	217.889	218.873	174.232	200.201	210.046	273.352	299.36
159.668	143.195	198.436	218.324	146.87	163.877	222.428	213.63	192.256	204.717	203.196	263.037	323.853
153.809	153.119	254.095	233.918	154.093	174.449	210.323	220.183	176.235	204.717	212.329	261.318	323.853
172.852	148.866	251.675	228.07	154.093	158.59	225.455	207.077	176.235	203.211	196.347	273.352	342.903
158.203	148.866	254.095	212.476	143.258	169.163	213.35	207.077	176.235	204.717	210.046	268.195	351.068
158.203	154.537	188.756	218.324	148.074	153.304	222.428	226.737	178.238	198.695	207.763	264.756	342.903
177.246	143.195	229.895	218.324	151.685	163.877	210.323	225.426	176.235	204.717	189.498	273.352	329.296
155.273	140.359	210.359	224.172	145.666	179.736	210.323	222.805	192.256	203.211	200.913	292.264	345.625
153.809	141.777	234.263	212.476	137.239	158.59	217.889	217.562	174.232	203.211	210.046	264.756	353.789
153.809	138.941	215.139	228.07	145.666	195.595	220.915	211.009	178.238	201.706	191.781	285.387	337.46
147.949	153.534	234.263	224.172	154.093	161.233	222.428	209.699	176.235	198.695	194.064	271.633	361.953
156.738	145.235	222.311	228.07	139.647	163.877	216.376	196.592	188.251	210.738	205.479	290.544	326.575
158.203	152.151	203.187	224.172	140.851	150.661	204.271	204.456	170.227	200.201	203.196	276.791	332.017
162.598	141.085	239.044	212.476	158.909	163.877	207.297	199.214	166.222	201.706	203.196	261.318	337.46
168.457	152.151	262.948	230.019	142.055	177.093	205.784	203.145	182.243	201.706	191.781	283.668	342.903
171.387	150.768	258.167	210.526	140.851	187.665	204.271	203.145	182.243	197.19	203.196	273.352	340.182
149.414	153.534	262.948	210.526	145.666	161.233	195.192	208.388	180.24	198.695	198.63	268.195	334.739
152.344	146.618	262.948	226.121	142.055	185.022	205.784	207.077	178.238	180.632	194.064	256.16	334.739
156.738	150.768	272.51	239.766	137.239	171.806	198.219	213.63	172.23	198.695	189.498	266.476	299.36
143.555	143.852	272.51	228.07	146.87	166.52	210.323	217.562	174.232	183.643	196.347	283.668	299.36
140.625	152.151	217.53	235.867	143.258	161.233	208.81	195.282	172.23	195.685	198.63	271.633	291.196
133.301	136.936	217.53	226.121	131.22	140.088	211.837	197.903	176.235	188.159	200.913	273.352	315.689
146.484	146.618	217.53	228.07	143.258	155.947	216.376	217.562	182.243	194.18	207.763	280.229	323.853
152.344	132.786	229.482	247.563	139.647	158.59	210.323	199.214	174.232	213.748	207.763	275.072	321.132

156.738	131.403	239.044	247.563	150.482	142.731	199.732	200.524	170.227	201.706	207.763	280.229	326.575
156.738	142.469	243.825	235.867	144.462	155.947	202.758	218.873	192.256	201.706	203.196	276.791	299.36
172.852	130.02	227.092	237.817	143.258	140.088	205.784	200.524	180.24	185.148	205.479	273.352	302.081
168.457	141.085	239.044	237.817	132.424	158.59	198.219	214.941	186.248	197.19	205.479	292.264	258.538
177.246	145.235	227.092	224.172	139.647	171.806	198.219	214.941	180.24	210.738	214.612	288.825	356.511
174.316	145.235	227.092	239.766	148.074	155.947	216.376	207.077	196.262	192.674	221.461	288.825	318.41
174.316	148.001	231.873	226.121	128.812	150.661	198.219	216.252	200.267	195.685	212.329	285.387	302.081
175.781	139.702	243.825	226.121	139.647	150.661	213.35	196.592	188.251	201.706	200.913	283.668	247.652
166.992	128.637	227.092	231.969	139.647	153.304	199.732	213.63	184.246	203.211	207.763	281.948	254.841
177.246	136.936	265.339	210.526	140.851	142.731	196.705	221.494	178.238	189.664	203.196	261.318	238.399
177.246	139.702	253.386	230.019	143.258	155.947	204.271	209.699	194.259	204.717	212.329	278.51	235.659
174.316	138.319	253.386	231.969	134.831	153.304	190.653	226.737	174.232	185.148	196.347	287.106	232.919
169.922	138.319	241.434	196.881	134.831	150.661	199.732	201.835	190.254	197.19	194.064	285.387	241.14
168.457	131.403	241.434	206.628	122.793	171.806	211.837	216.252	184.246	201.706	200.913	293.983	197.296
168.457	130.02	250.996	239.766	140.851	187.665	201.245	216.252	176.235	192.674	187.215	287.106	216.478
164.062	120.337	222.311	204.678	133.628	177.093	213.35	211.009	190.254	177.622	191.781	287.106	235.659
177.246	128.637	236.653	247.563	143.258	153.304	199.732	212.32	176.235	180.632	205.479	283.668	257.581
183.105	121.721	236.653	224.172	126.404	163.877	205.784	213.63	164.219	177.622	187.215	293.983	213.737
174.316	114.805	234.263	228.07	131.22	140.088	211.837	212.32	168.224	186.653	196.347	287.106	183.595
165.527	114.805	227.092	196.881	130.016	124.229	207.297	220.183	180.24	188.159	200.913	297.421	208.257
187.5	118.954	215.139	231.969	137.239	132.159	196.705	224.115	170.227	197.19	203.196	288.825	197.296
178.711	130.02	231.873	233.918	136.035	134.802	201.245	213.63	180.24	189.664	200.913	281.948	205.517
183.105	136.936	231.873	239.766	133.628	155.947	201.245	216.252	200.267	168.59	198.63	290.544	224.698
190.43	121.721	210.359	247.563	132.424	148.018	207.297	216.252	172.23	174.611	198.63	306.017	205.517
188.965	127.253	222.311	237.817	131.22	153.304	205.784	220.183	164.219	183.643	205.479	314.613	227.439
181.641	135.553	217.53	249.513	139.647	140.088	201.245	216.252	194.259	183.643	214.612	302.579	246.62
203.613	134.169	188.845	239.766	136.035	150.661	207.297	225.426	180.24	201.706	203.196	309.456	205.517
193.359	131.403	198.406	243.665	139.647	155.947	201.245	208.388	178.238	201.706	207.763	302.579	227.439

180.176	120.337	162.55	239.766	140.851	163.877	204.271	216.252	176.235	194.18	200.913	309.456	274.022
180.176	128.637	157.769	239.766	144.462	150.661	199.732	216.252	168.224	198.695	205.479	297.421	252.101
202.148	130.02	154.877	226.121	143.258	137.445	198.219	224.115	176.235	207.727	203.196	300.86	257.581
159.668	128.637	162.137	239.766	134.831	140.088	208.81	224.115	176.235	222.78	207.763	328.367	263.061
158.203	134.169	174.236	216.374	145.666	129.515	207.297	233.29	174.232	206.222	223.744	314.613	268.542
184.57	130.02	164.94	239.766	136.035	142.731	216.376	226.737	164.219	204.717	205.479	300.86	309.645
164.062	121.721	191.235	208.577	139.647	166.52	192.166	234.6	156.208	194.18	200.913	316.332	326.575
153.809	123.104	129.084	243.665	136.035	132.159	201.245	220.183	166.222	207.727	210.046	300.86	332.017
153.809	131.403	124.303	239.766	143.258	113.656	225.455	214.941	168.224	218.264	214.612	321.49	304.803
180.176	124.487	145.817	239.766	140.851	137.445	223.942	225.426	178.238	198.695	210.046	311.175	318.41
180.176	132.786	112.351	230.019	136.035	145.374	222.428	226.737	166.222	212.243	216.895	314.613	310.246
171.387	130.02	129.084	245.614	140.851	148.018	228.481	218.873	172.23	197.19	212.329	300.86	332.017
191.895	124.487	150.598	241.715	133.628	145.374	214.863	213.63	176.235	206.222	205.479	338.682	356.511
174.316	124.487	145.817	228.07	136.035	126.872	208.81	233.29	164.219	191.169	237.443	314.613	351.068
188.965	124.487	169.721	263.158	126.404	124.229	213.35	220.183	160.214	198.695	210.046	318.052	367.396
171.387	134.169	160.159	243.665	131.22	137.445	234.533	228.047	180.24	192.674	216.895	328.367	342.903
175.781	130.02	129.084	214.425	133.628	134.802	217.889	166.448	180.24	206.222	216.895	335.244	353.789
172.852	130.02	160.159	230.019	133.628	150.661	217.889	169.069	168.224	197.19	207.763	321.49	334.739
174.316	131.403	143.426	237.817	130.016	153.304	214.863	176.933	168.224	201.706	216.895	319.771	353.789
166.992	141.085	143.426	243.665	133.628	140.088	229.994	170.38	194.259	206.222	219.178	304.298	323.853
181.641	130.02	138.645	255.361	137.239	134.802	219.402	183.486	174.232	195.685	210.046	312.894	348.346
175.781	135.553	162.55	249.513	131.22	137.445	208.81	186.107	184.246	194.18	212.329	309.456	383.725
175.781	124.487	145.817	265.107	131.22	124.229	189.14	184.797	182.243	200.201	210.046	297.421	356.511
155.273	130.02	152.988	274.854	142.055	140.088	216.376	176.933	182.243	188.159	194.064	319.771	372.839
153.809	131.403	141.036	288.499	116.774	132.159	216.376	192.661	174.232	201.706	214.612	318.052	391.889
164.062	136.936	155.378	253.411	125.201	140.088	216.376	187.418	166.222	203.211	207.763	306.017	353.789
171.387	131.403	141.036	280.702	126.404	142.731	214.863	178.244	166.222	194.18	207.763	278.51	348.346
159.668	130.02	148.207	269.006	127.608	145.374	207.297	178.244	162.216	192.674	216.895	319.771	307.524

165.527	131.403	152.988	255.361	117.978	126.872	228.481	183.486	174.232	200.201	212.329	314.613	353.789
165.527	138.319	131.474	267.057	130.016	145.374	228.481	190.039	166.222	197.19	203.196	330.086	353.789
171.387	136.936	155.378	241.715	121.589	161.233	220.915	184.797	182.243	200.201	205.479	323.209	367.396
161.133	139.702	133.865	224.172	123.997	148.018	222.428	184.797	166.222	186.653	207.763	340.401	340.182
164.062	141.085	176.892	245.614	122.793	126.872	217.889	197.903	168.224	198.695	207.763	309.456	351.068
174.316	131.403	164.94	245.614	115.57	137.445	239.073	190.039	172.23	180.632	210.046	326.648	342.903
180.176	130.02	179.283	255.361	117.978	142.731	225.455	197.903	168.224	177.622	210.046	314.613	342.903

MI

101.513	89.4071	89.4495	91.0931	86.5633	105.672	96.0803	89.1089	96.3232	93.2835827	92.5778	98.7078926
99.1242	93.9921	90.3096	90.2256	103.359	98.8106	93.2122	107.673	99.4303	89.5522376	93.3759	95.8467912
94.3471	89.4071	98.0505	100.636	91.7313	93.3211	100.382	107.673	90.1088	104.477607	90.1836	107.291185
99.1242	96.2846	100.631	95.4309	117.571	103.614	96.0803	100.248	108.752	93.2835827	104.549	95.8467912
90.7643	103.162	96.3303	104.974	98.1912	100.183	98.9484	89.1089	91.6623	117.537312	101.357	94.4162435
100.318	98.5771	92.8899	99.7687	87.8553	87.8317	110.421	103.96	100.984	106.343282	93.3759	101.568994
102.707	84.8221	96.3303	97.166	111.111	102.928	93.2122	85.396	87.0016	97.0149278	97.3663	101.568994
91.9586	96.2846	103.211	98.0335	105.943	97.4382	103.25	85.396	85.448	97.0149278	97.3663	115.874483
99.1242	112.332	102.351	95.4309	105.943	98.1244	98.9484	111.386	114.966	100.746267	95.7702	101.568994
94.3471	93.9921	106.651	98.0335	94.3152	85.0869	96.0803	96.5347	107.198	97.0149278	102.953	87.2634991
101.513	91.6996	108.372	104.974	83.9793	98.8106	97.5143	96.5347	88.5552	108.208958	90.1836	105.860637
99.1242	98.5771	101.491	99.7687	100.775	100.869	98.9484	111.386	96.3232	93.2835827	98.1644	100.138434
100.318	112.332	92.0298	90.2256	100.775	96.7521	103.25	103.96	105.645	83.9552228	102.155	94.4162435
99.1242	121.502	108.372	104.974	114.987	103.614	103.25	89.1089	105.645	108.208958	95.7702	100.138434
91.9586	100.87	110.952	104.974	98.1912	99.4968	96.0803	85.396	90.1088	100.746267	100.559	104.430089
103.901	107.747	104.071	95.4309	99.4832	103.614	96.0803	96.5347	108.752	93.2835827	102.155	100.138434
100.318	114.625	100.631	104.974	107.235	85.0869	103.25	103.96	90.1088	93.2835827	101.357	101.568994
102.707	100.87	103.211	101.504	99.4832	106.359	110.421	118.812	97.8767	97.0149278	99.7606	104.430089
100.318	98.5771	106.651	104.974	107.235	96.0659	98.9484	100.248	97.8767	100.746267	97.3663	95.8467912

99.1242	100.87	96.3303	101.504	104.651	109.103	98.9484	100.248	99.4303	93.2835827	109.338	74.3885578
94.3471	119.209	96.3303	109.312	112.403	107.731	98.9484	103.96	107.198	93.2835827	111.732	87.2634991
103.901	98.5771	98.0505	98.0335	94.3152	96.0659	93.2122	111.386	102.538	97.0149278	110.934	95.8467912
107.484	98.5771	98.9106	101.504	96.8992	106.359	96.0803	126.238	110.306	113.805972	102.155	97.277339
101.513	100.87	103.211	96.2984	90.4393	100.183	96.0803	111.386	107.198	108.208958	101.357	97.277339
105.096	98.5771	98.9106	97.166	105.943	102.242	103.25	100.248	105.645	100.746267	103.751	108.721732
101.513	89.4071	96.3303	96.2984	86.5633	103.614	98.9484	103.96	113.413	100.746267	101.357	104.430089
100.318	93.9921	98.0505	102.371	109.819	103.614	103.25	74.2574	96.3232	104.477607	101.357	105.860637
107.484	91.6996	102.351	107.577	104.651	102.928	108.987	100.248	99.4303	108.208958	106.943	94.4162435
113.455	107.747	100.631	107.577	82.6873	107.045	103.25	96.5347	100.984	100.746267	101.357	104.430089
197.054	199.447	157.397	163.1	316.537	221.638	174.952	163.366	201.72	233.20896	161.798	115.874483
206.608	169.644	150.516	150.954	276.486	219.212	162.046	141.089	210.96	223.880594	154.553	140.193818
205.414	185.692	151.376	159.63	270.026	231.466	169.216	141.089	169.384	229.477609	152.138	181.679737
212.58	181.107	153.956	165.703	233.85	214.446	166.348	152.228	181.702	229.477609	156.968	180.249189
208.997	183.399	151.376	153.557	258.398	213.765	164.914	178.218	175.543	208.955214	158.819	174.526998
225.717	169.644	141.055	144.014	254.522	195.384	173.518	155.941	177.083	233.20896	158.021	167.374248
218.551	158.182	146.216	134.471	251.938	213.765	169.216	155.941	181.702	231.343273	163.607	183.110296
220.939	165.059	135.894	162.232	244.186	189.938	157.744	167.079	167.844	236.940288	164.405	197.415785
211.385	167.352	142.775	159.63	224.806	194.023	167.782	155.941	177.083	225.746258	163.607	171.665903
211.385	165.059	137.615	149.219	236.434	182.45	169.216	174.505	183.242	220.149243	158.819	193.12413
210.191	167.352	141.055	151.822	204.134	185.173	172.084	144.802	172.463	216.417915	162.011	194.554678
205.414	169.644	144.495	155.292	228.682	189.387	169.216	148.515	169.384	231.343273	158.819	193.12413
207.803	162.767	135.894	150.954	220.93	199.68	169.216	152.228	158.605	227.611945	165.204	194.554678
198.248	187.984	142.775	145.749	211.886	184.584	163.48	148.515	177.083	197.761184	154.828	191.693582
199.443	165.059	136.755	152.689	211.886	194.19	179.254	155.941	155.525	220.149243	159.617	183.110296
204.22	171.937	141.055	143.146	218.346	175.663	177.82	155.941	164.764	201.492535	151.636	200.276869
193.471	153.597	134.174	150.087	228.682	181.839	170.65	167.079	166.304	197.761184	145.251	203.137988
183.917	167.352	136.755	146.617	195.09	183.211	163.48	159.653	163.224	208.955214	158.021	205.999083

192.277	155.889	137.615	168.305	193.798	188.015	173.518	144.802	172.463	208.955214	158.021	188.832487
186.306	139.842	140.195	134.471	215.762	178.408	172.084	148.515	163.224	225.746258	163.607	195.985237
189.889	181.107	143.635	146.617	211.886	183.13	166.348	141.089	172.463	225.746258	162.011	184.540844
179.14	160.474	138.475	138.809	208.01	190.619	166.348	159.653	170.923	210.8209	162.809	195.985237
167.197	158.182	127.294	151.822	202.842	183.13	167.782	163.366	181.702	223.880594	159.617	170.235343
188.694	169.644	142.775	150.087	191.214	185.853	174.952	141.089	170.923	210.8209	156.425	171.665903
167.197	162.767	136.755	150.087	210.594	181.088	176.386	152.228	163.224	205.223885	152.434	194.554678
124.204	142.134	135.894	136.206	200.258	181.088	160.612	133.663	181.702	201.492535	150.04	190.263035
144.506	160.474	142.775	157.027	201.731	180.407	150.574	141.089	172.463	184.70149	161.213	175.957546
137.341	144.427	140.195	139.676	183.51	185.173	157.744	148.515	183.242	208.955214	155.626	191.693582
134.952	169.644	142.775	147.484	192.62	177.684	167.782	129.95	177.083	199.626871	154.03	183.110296
144.506	167.352	142.775	146.617	193.922	178.365	166.348	159.653	177.083	212.686564	158.021	173.09645
143.312	162.767	143.635	144.881	199.128	172.919	159.178	118.812	161.684	207.08955	162.809	194.554678
145.701	176.522	147.936	137.941	193.922	172.919	160.612	122.525	157.065	207.08955	153.232	175.957546
150.478	169.644	144.495	149.219	206.937	174.28	160.612	141.089	164.764	197.761184	158.021	174.526998
154.061	160.474	142.775	152.689	199.128	179.046	146.272	126.238	180.163	192.164169	151.636	183.110296
142.118	153.597	135.034	152.689	210.841	172.919	159.178	159.653	174.003	194.029856	169.194	185.971392
142.118	162.767	133.314	148.352	195.223	173.599	152.008	129.95	157.065	225.746258	159.617	185.971392
150.478	167.352	138.475	144.014	195.223	177.684	156.31	144.802	169.384	210.8209	161.213	170.235343
145.701	158.182	144.495	148.352	208.238	177.003	146.272	155.941	183.242	197.761184	164.405	174.526998
152.866	146.719	136.755	144.881	218.65	166.792	173.518	126.238	157.065	218.283579	155.626	185.971392
140.924	165.059	147.936	150.954	174.4	178.365	169.216	126.238	153.985	201.492535	163.607	180.249189
161.226	171.937	141.055	145.749	197.826	166.792	162.046	144.802	161.684	188.432841	160.415	177.388094
169.586	165.059	141.915	144.881	216.047	174.961	172.084	144.802	161.684	192.164169	162.011	188.832487
158.838	158.182	137.615	144.014	208.238	169.515	170.65	148.515	158.605	197.761184	152.434	175.957546
143.312	167.352	146.216	143.146	201.731	164.749	157.744	137.376	160.144	188.432841	161.213	184.540844
137.341	158.182	147.076	141.411	190.017	174.28	162.046	185.644	181.702	203.358199	166.8	184.540844
145.701	167.352	146.216	141.411	184.812	159.303	162.046	144.802	141.666	173.50746	165.823	175.957546

152.866	162.767	136.755	127.53	200.429	168.153	167.782	148.515	155.525	190.298505	166.628	188.832487
150.478	151.304	133.314	135.338	173.098	172.238	166.348	137.376	164.764	171.641785	148.113	173.09645
155.255	160.474	134.174	143.146	218.65	161.345	172.084	126.238	152.445	180.970151	152.138	183.110296
162.42	153.597	126.433	148.352	190.017	159.984	149.14	181.931	195.561	177.2388	153.748	175.957546
175.557	149.012	117.833	136.95	190.017	169.515	170.65	159.653	189.402	190.298505	165.018	181.679737
168.392	158.182	122.993	134.316	199.128	157.941	163.48	148.515	163.224	192.164169	161.798	174.526998
171.975	165.059	129.014	124.659	212.143	162.026	156.31	181.931	187.862	169.776121	158.578	187.401939
167.197	160.474	116.972	128.171	193.922	149.772	169.216	144.802	186.322	184.70149	160.993	193.12413
175.557	153.597	135.034	132.56	190.017	162.707	156.31	148.515	189.402	208.955214	169.043	175.957546
180.334	155.889	141.915	137.827	214.746	162.026	152.008	141.089	180.163	208.955214	160.188	178.818641
180.334	178.814	146.216	137.827	197.826	164.749	163.48	126.238	155.525	173.50746	147.309	195.985237
183.917	165.059	143.635	131.682	195.223	164.749	141.969	141.089	181.702	171.641785	157.773	181.679737
170.78	167.352	144.495	155.385	180.907	162.707	169.216	129.95	167.844	199.626871	161.798	184.540844
183.917	158.182	131.594	139.583	197.826	168.153	152.008	137.376	164.764	190.298505	169.043	188.832487
185.111	146.719	132.454	128.171	187.415	162.026	160.612	148.515	166.304	186.567165	146.504	183.110296
179.14	158.182	136.755	134.316	174.4	160.664	164.914	144.802	177.083	158.582091	160.188	177.388094
188.694	169.644	141.915	137.827	186.113	170.876	150.574	137.376	178.623	175.373136	162.603	177.388094
175.557	165.059	147.076	123.781	192.62	156.58	166.348	122.525	177.083	169.776121	159.383	168.804795
187.5	171.937	136.755	140.461	182.209	166.111	159.178	126.238	167.844	194.029856	165.823	173.09645
195.86	158.182	135.034	150.118	161.385	161.345	173.518	111.386	163.224	173.50746	151.333	177.388094
187.5	162.767	150.516	125.537	184.812	157.941	164.914	155.941	180.163	186.567165	153.748	181.679737
188.694	194.862	163.417	121.148	174.4	149.772	162.046	122.525	174.003	194.029856	160.993	171.665903
180.334	171.937	136.755	136.95	170.495	154.537	152.008	144.802	177.083	171.641785	160.993	164.513152
180.334	171.937	146.216	113.247	171.797	154.537	160.612	141.089	177.083	175.373136	160.188	173.09645
182.723	165.059	140.195	131.682	178.304	153.857	157.744	152.228	181.702	160.447755	160.993	165.9437
177.946	181.107	143.635	115.002	192.62	150.453	149.14	167.079	187.862	169.776121	161.798	174.526998
173.169	183.399	150.516	128.171	182.209	149.091	144.837	155.941	186.322	169.776121	152.943	167.374248
183.917	176.522	143.635	111.491	184.812	149.772	163.48	137.376	187.862	173.50746	160.188	178.818641

185.111	155.889	133.314	122.903	186.113	140.241	179.254	163.366	190.941	171.641785	167.433	167.374248
176.752	165.059	142.775	143.973	158.782	137.518	160.612	103.96	172.463	175.373136	156.968	185.971392
179.14	165.059	151.376	130.804	169.194	151.134	163.48	126.238	158.605	167.910446	147.309	177.388094
188.694	171.937	147.936	126.415	158.782	140.922	159.178	129.95	164.764	169.776121	147.309	188.832487
182.723	171.937	147.936	130.804	173.098	146.368	162.046	174.505	200.181	136.194032	166.628	160.221509
176.752	174.229	146.216	115.002	153.576	138.199	179.254	141.089	181.702	113.805972	157.773	174.526998
186.306	169.644	149.656	132.56	165.289	150.453	176.386	141.089	186.322	162.313431	145.699	173.980493
176.752	162.767	136.755	121.148	167.892	142.283	180.688	141.089	177.083	149.253726	144.089	166.908115
183.917	165.059	145.356	129.049	147.068	142.964	167.782	141.089	183.242	130.597017	151.333	176.80944
179.14	171.937	150.516	116.758	175.701	144.326	166.348	148.515	175.543	123.134327	160.415	161.25021
177.946	167.352	148.796	136.95	175.701	131.391	162.046	133.663	183.242	138.059696	158.021	179.638399
185.111	167.352	142.775	128.171	182.209	152.495	144.837	137.376	169.384	128.731342	158.819	172.56602
185.111	167.352	146.216	136.072	166.591	137.518	166.348	148.515	167.844	145.522386	171.588	179.638399
177.946	165.059	141.915	135.194	200.429	138.199	159.178	141.089	172.463	119.402987	169.194	178.223925
179.14	171.937	143.635	130.804	154.877	143.645	153.442	152.228	177.083	139.925371	146.848	172.56602
177.946	174.229	117.833	129.926	171.797	137.518	166.348	122.525	170.923	132.462681	164.405	158.421263
185.111	174.229	136.755	124.659	182.209	149.772	174.952	144.802	190.941	126.865666	174.781	157.00679
179.14	178.814	129.874	131.682	158.782	140.241	164.914	144.802	180.163	151.119401	167.598	171.151547
179.14	178.814	135.894	123.781	170.495	141.603	170.65	152.228	169.384	132.462681	172.386	172.56602
185.111	176.522	140.195	129.049	166.591	143.645	156.31	133.663	161.684	95.1492525	169.992	189.539724
185.111	183.399	147.936	129.926	190.017	138.879	162.046	159.653	172.463	126.865666	164.405	178.223925
177.946	215.494	141.915	144.85	167.892	129.349	166.348	152.228	166.304	143.656711	166.002	175.394967
185.111	199.447	151.376	131.682	163.988	134.114	169.216	118.812	155.525	110.074622	162.809	172.56602
177.946	194.862	129.874	123.781	169.194	139.56	164.914	129.95	161.684	115.671637	157.223	166.908115
173.169	190.277	146.216	122.903	178.304	130.029	174.952	167.079	181.702	112.923749	164.405	171.151547
170.78	187.984	149.656	129.049	180.907	132.752	147.706	148.515	183.242	140.691882	162.603	176.80944
163.615	220.079	142.775	121.148	149.671	136.156	167.782	141.089	163.224	124.031002	159.383	176.80944
138.535	183.399	156.537	121.148	165.289	128.668	169.216	118.812	177.083	109.221334	144.894	164.079168

130.175	199.447	147.936	117.636	121.039	139.56	164.914	170.792	184.782	111.072547	152.138	158.421263
136.146	181.107	151.376	124.659	109.325	108.925	177.82	118.812	167.844	112.923749	140.064	166.908115
143.312	187.984	146.216	111.491	126.244	135.476	159.178	137.376	160.144	131.435842	145.699	171.151547
146.895	190.277	153.096	111.491	137.958	156.58	162.046	152.228	169.384	120.328588	150.528	178.223925
137.341	153.597	116.972	118.514	149.671	149.772	154.876	129.95	169.384	105.51892	137.649	155.592316
157.643	149.012	135.894	126.415	147.068	151.814	152.008	148.515	184.782	75.8995736	141.674	175.394967
149.283	116.917	121.273	124.659	114.531	145.687	160.612	103.96	164.764	112.923749	135.234	171.151547
143.312	112.332	120.413	120.27	152.274	149.772	167.782	148.515	175.543	85.1556146	135.234	188.12525
148.089	110.04	116.972	122.903	148.37	140.922	173.518	163.366	172.463	103.667708	135.234	164.079168
149.283	135.257	119.553	121.148	180.907	142.283	167.782	181.931	197.101	109.221334	134.429	176.80944
144.506	142.134	118.693	115.002	165.289	141.603	157.744	155.941	170.923	72.1971539	136.844	182.467345
138.535	123.794	122.993	122.026	175.701	134.795	150.574	163.366	175.543	109.221334	136.844	149.934411
144.506	128.379	121.273	113.247	147.068	124.583	147.706	152.228	166.304	81.4532004	130.404	181.052872
152.866	135.257	122.133	128.171	182.209	142.964	163.48	129.95	163.224	87.0068272	139.259	181.052872
146.895	132.964	112.672	103.59	173.098	129.349	166.348	144.802	167.844	70.3459412	123.16	104.671193
148.089	149.012	122.993	105.346	165.289	131.391	166.348	133.663	166.304	72.7611931	125.574	145.916008
151.672	142.134	102.351	111.491	170.495	130.029	169.216	155.941	181.702	97.0149278	127.989	203.137988
152.866	135.257	114.392	106.224	154.877	125.945	160.612	148.515	174.003	93.2835827	129.599	210.290726

MI+ARM36

90.21544194	111.446	96.5073	99.3584	103.929	97.8532	104.717	93.361	90.3614	101.597	108.264	92.8793
90.21544194	105.422	115.809	91.1493	109.923	99.0593	104.717	105.809	90.3614	91.4369	84.728	102.167
91.5619397	108.434	85.4779	97.943	95.551	94.0741	110.377	99.5851	90.3614	104.499	100.418	111.455
87.52244067	99.3976	99.2647	87.1863	100.246	119.402	90.566	97.5104	93.9759	97.2424	106.695	102.167
94.25493522	93.3735	99.2647	91.1493	114.473	90.0539	107.547	107.884	133.735	105.951	111.402	130.031
95.60143873	102.41	97.886	93.98	102.123	120.447	101.887	95.4357	101.205	97.2424	111.402	111.455
92.90843746	102.41	104.779	99.6414	110.212	95.8431	93.3962	95.4357	86.747	98.6938	111.402	83.5913
98.29444	105.422	104.779	92.8477	111.151	102.838	87.7359	93.361	104.819	108.853	91.0042	65.0155
87.52244067	102.41	100.643	90.5831	93.8177	114.979	96.2264	99.5851	97.5904	82.7286	100.418	148.607
100.9874355	105.422	95.1287	91.7154	93.0955	113.854	87.7359	85.0622	97.5904	98.6938	117.678	74.3034
95.60143873	93.3735	96.5073	108.417	106.746	96.4863	107.547	101.66	108.434	78.3745	97.2803	55.7276
98.29444	105.422	103.401	104.737	88.4732	105.813	110.377	95.4357	86.747	91.4369	94.1423	120.743
94.25493522	102.41	102.022	106.718	99.3067	96.5667	93.3962	103.734	86.747	104.499	117.678	92.8793
103.680431	108.434	97.886	110.115	98.7289	94.3957	93.3962	105.809	133.735	98.6938	87.8661	111.455
95.60143873	102.41	95.1287	100.491	94.8288	118.517	90.566	101.66	115.663	94.3396	95.7113	92.8793
106.3734266	102.41	95.1287	108.983	99.2344	90.0539	118.868	120.332	104.819	95.791	105.126	148.607
107.7199243	96.3855	102.022	111.247	108.84	102.838	113.208	103.734	112.048	103.048	100.418	130.031
109.0664221	99.3976	102.022	99.6415	91.0732	107.019	127.358	130.705	126.506	107.402	111.402	74.3034
117.1454202	93.3735	102.022	103.038	118.807	93.5917	90.566	95.4357	115.663	103.048	87.8661	83.5913
109.0664221	117.47	95.1287	93.6969	89.051	100.105	99.0566	85.0622	93.9759	105.951	95.7113	55.7276

102.3339333	93.3735	107.537	96.2446	109.418	107.904	104.717	97.5104	97.5904	103.048	119.247	102.167
107.7199243	69.2771	99.2647	96.8107	99.3789	88.687	76.4151	101.66	108.434	91.4369	94.1423	55.7276
92.90843746	120.482	97.886	103.888	101.184	95.039	101.887	99.5851	93.9759	120.464	92.5732	111.455
105.0269288	90.3614	104.779	107.284	99.6678	110.557	104.717	103.734	83.1325	105.951	92.5732	102.167
88.86893843	96.3855	103.401	104.737	81.2509	94.9586	93.3962	101.66	79.5181	117.562	89.4351	130.031
99.64093776	93.3735	102.022	108.983	95.2622	91.5011	99.0566	93.361	90.3614	105.951	98.8494	102.167
105.0269288	90.3614	93.75	101.057	91.4344	87.4809	116.038	118.257	86.747	94.3396	97.2803	111.455
115.7989224	96.3855	97.886	105.303	94.3233	100.667	87.7359	95.4357	93.9759	105.951	95.7113	120.743
110.4129313	93.3735	100.643	107.284	110.429	83.6215	96.2264	87.1369	112.048	87.0827	83.159	74.3034
106.3734266	99.3976	102.022	85.7709	88.0399	85.7924	90.566	85.0622	83.1325	98.6938	100.418	102.167
187.1633727	139.797	191.636	183.853	228.143	200.45	200.943	161.91	285.542	188.679	152.197	176.471
185.8168749	154.669	190.257	180.678	256.175	228.11	161.321	168.218	285.542	177.068	144.351	185.759
179.0843861	148.72	190.257	170.576	254.514	218.22	175.472	187.143	300	165.457	144.351	204.334
184.4703772	148.72	187.5	165.381	264.192	248.05	181.132	197.656	296.386	164.006	142.782	176.471
179.0843861	145.745	183.364	163.072	158.674	226.1	155.66	178.732	292.771	162.554	141.213	167.183
180.4308839	157.643	180.607	175.194	180.919	256.01	147.17	187.143	267.47	184.325	139.644	241.486
175.0448813	160.617	187.5	166.824	215.802	227.949	141.509	174.526	274.699	161.103	136.506	176.471
171.0053881	151.694	183.364	163.36	207.858	222.079	138.679	178.732	249.398	165.457	138.075	191.901
162.92639	133.848	187.5	169.422	211.902	203.425	130.189	170.321	292.771	146.589	150.628	210.177
160.2333945	154.669	186.121	165.958	219.63	239.849	121.698	172.423	300	158.2	131.799	219.315
153.5008942	142.771	179.228	156.145	212.986	239.849	138.679	172.423	296.386	166.909	158.473	219.315
161.5798922	142.771	180.607	155.279	221.436	239.045	138.679	185.04	314.458	162.554	144.351	191.901
168.3123925	116.002	181.985	155.279	229.597	215.888	130.189	159.807	263.855	158.2	144.351	164.486
164.2728878	133.848	177.849	160.763	229.669	210.099	141.509	176.629	278.313	153.846	127.092	164.486
161.5798922	121.95	183.364	150.661	235.447	223.929	130.189	187.143	285.542	156.749	155.335	164.486
165.6193855	130.873	184.743	154.125	215.947	223.848	141.509	176.629	281.928	175.617	138.075	228.453
168.3123925	136.822	180.607	162.206	217.897	223.848	141.509	176.629	256.627	162.554	144.351	191.901
152.1543964	139.797	187.5	146.909	230.103	219.748	118.868	182.937	271.084	165.457	134.937	182.762

156.1939012	136.822	186.121	142.868	214.575	214.682	133.019	157.704	260.241	153.846	139.644	155.348
158.8868967	163.592	180.607	154.413	220.858	207.848	113.208	164.013	296.386	155.298	138.075	210.177
149.4614009	142.771	188.879	169.133	205.402	185.736	121.698	159.807	267.47	152.395	144.351	191.901
150.8078987	130.873	183.364	150.95	237.298	189.274	121.698	159.807	238.554	179.971	142.782	185.759
141.3824028	124.925	183.364	164.515	221.039	214.441	107.547	166.115	256.627	169.811	160.042	167.183
154.8473919	139.797	180.607	157.299	235.541	178.339	110.377	170.321	245.783	165.457	133.368	167.183
158.8868967	127.899	177.849	160.474	233.124	198.119	127.358	161.91	253.012	158.2	139.644	139.319
160.2333945	127.899	181.985	154.702	230.853	185.093	124.528	161.91	300	158.2	142.782	185.759
162.92639	130.873	181.985	147.198	240.081	185.093	130.189	155.602	267.47	165.457	142.782	176.471
149.4614009	130.873	180.607	146.62	252.02	171.987	127.358	153.499	249.398	166.909	144.351	167.183
157.540399	139.797	186.121	153.547	226.166	185.093	101.887	153.499	231.325	166.909	131.799	139.319
152.1543964	148.72	181.985	155.568	221.186	206.883	113.208	153.499	253.012	146.589	138.075	120.743
148.1149031	139.797	181.985	152.104	250.042	168.047	104.717	147.191	267.47	155.298	141.213	102.167
156.1939012	148.72	187.5	163.36	234.003	177.696	104.717	149.293	267.47	162.554	142.782	102.167
146.7684054	139.797	186.121	147.775	232.391	193.777	110.377	157.704	260.241	166.909	149.059	130.031
141.3824028	130.873	183.364	161.629	243.084	181.233	93.3962	145.088	274.699	155.298	141.213	130.031
157.540399	127.899	175.092	159.031	248.724	172.55	96.2264	147.191	234.94	149.492	150.628	111.455
149.4614009	133.848	184.743	151.816	237.005	194.581	76.4151	147.191	242.169	172.714	156.904	130.031
146.7684054	121.95	187.5	155.279	230.927	181.635	82.0755	132.472	296.386	165.457	125.523	92.8793
146.7684054	148.72	177.849	149.218	246.453	179.304	104.717	136.677	278.313	156.749	128.661	139.319
154.8473919	151.694	181.985	165.381	222.577	157.916	90.566	134.574	220.482	171.263	145.921	102.167
146.7684054	148.72	180.607	157.011	239.569	158.077	84.9057	132.472	220.482	161.103	139.644	127.934
150.8078987	142.771	179.228	151.816	238.543	168.208	90.566	132.472	216.867	174.165	141.213	127.934
148.1149031	124.925	179.228	153.259	227.557	177.937	96.2264	142.985	220.482	156.749	138.075	91.3812
141.3824028	133.848	168.199	153.259	227.265	180.671	101.887	128.266	209.639	159.652	134.937	63.9668
140.0359051	130.873	175.092	147.198	245.282	174.158	96.2264	130.369	209.639	155.298	131.799	82.2431
135.9964118	139.797	177.849	157.877	232.318	148.911	87.7359	126.164	227.711	171.263	141.213	82.2431
135.9964118	124.925	180.607	158.454	233.929	153.735	84.9057	138.78	249.398	178.52	131.799	54.8287

138.6894073	145.745	177.849	150.372	219.648	144.167	101.887	138.78	220.482	162.554	128.661	100.519
146.7684054	142.771	177.849	165.958	239.935	163.062	104.717	128.266	253.012	168.36	133.368	100.519
140.0359051	136.822	183.364	161.34	243.524	161.373	118.868	145.088	253.012	162.554	139.644	91.3812
144.0754098	130.873	175.092	159.32	226.312	140.709	104.717	130.369	249.398	175.617	158.473	73.105
142.7289006	136.822	168.199	151.527	216.425	169.333	107.547	138.78	249.398	168.36	150.628	91.3812
140.0359051	121.95	169.577	160.474	241.986	140.79	104.717	142.985	242.169	162.554	138.075	82.2431
145.4219076	113.027	165.441	167.978	224.774	161.293	107.547	117.753	231.325	161.103	130.23	63.9668
138.6894073	124.925	176.471	161.34	233.417	166.117	113.208	147.191	224.096	162.554	134.937	92.8793
133.3034163	157.643	173.713	167.401	221.991	153.011	90.566	142.985	245.783	166.909	138.075	65.0155
134.649914	154.669	173.713	157.011	227.631	174.56	121.698	136.677	220.482	145.138	139.644	92.8793
133.3034163	133.848	173.713	161.34	240.228	158.961	118.868	147.191	216.867	169.811	144.351	65.0155
131.956907	133.848	175.092	156.433	222.064	152.448	118.868	134.574	224.096	172.714	125.523	111.455
130.6104093	139.797	175.092	152.97	233.27	149.715	99.0566	136.677	245.783	166.909	133.368	74.3034
129.2639115	151.694	177.849	147.775	247.918	144.89	113.208	134.574	224.096	156.749	127.092	120.743
127.9174137	130.873	186.121	162.495	229.828	171.746	135.849	136.677	231.325	177.068	139.644	55.7276
121.1849249	139.797	176.471	155.279	209.98	160.087	121.698	124.061	231.325	156.749	120.816	111.455
123.8779204	151.694	170.956	153.259	233.783	139.744	93.3962	142.985	227.711	165.457	145.921	65.0155
127.9174137	116.002	176.471	163.938	231.146	171.906	118.868	136.677	227.711	158.2	142.782	102.167
126.570916	148.72	181.985	150.661	233.856	134.357	113.208	124.061	249.398	161.103	141.213	65.0155
117.1454202	136.822	175.092	156.433	250.115	160.408	99.0566	132.472	253.012	169.811	136.506	46.4396
125.2244182	139.797	169.577	150.95	231.805	160.408	116.038	136.677	249.398	171.263	138.075	46.4396
131.956907	133.848	179.228	150.084	239.203	166.198	96.2264	138.78	242.169	155.298	145.921	74.3034
131.956907	116.002	170.956	144.023	253.792	132.025	110.377	134.574	224.096	159.652	144.351	18.5759
137.3429096	136.822	176.471	146.043	243.464	168.288	101.887	136.677	216.867	165.457	139.644	74.3034
134.649914	145.745	161.305	139.116	242.958	136.769	118.868	130.369	216.867	179.971	122.385	46.4396
127.9174137	142.771	169.577	133.921	231.041	147.785	104.717	138.78	220.482	164.006	139.644	83.5913
142.7289006	139.797	166.82	131.035	240.792	163.383	107.547	140.883	231.325	169.811	147.49	83.5913
138.6894073	136.822	164.062	126.705	236.747	150.117	101.887	145.088	216.867	150.943	152.197	65.0155

142.7289006	148.72	181.985	130.169	233.569	139.181	107.547	128.266	216.867	171.263	141.213	83.5913
137.3429096	133.848	147.518	134.498	239.78	153.654	79.2453	117.753	253.012	169.811	150.628	46.4396
138.6894073	154.669	155.79	139.693	237.975	158.559	70.7547	134.574	256.627	169.811	145.921	55.7276
138.6894073	130.873	169.577	127.283	229.669	170.137	96.2264	130.369	202.41	164.006	127.092	55.7276
134.649914	139.797	175.092	135.653	245.414	157.031	96.2264	126.164	238.554	174.165	120.816	65.0155
129.2639115	116.002	172.335	126.128	239.492	121.814	93.3962	124.061	206.024	172.714	133.368	18.5759
142.7289006	130.873	183.364	121.799	218.619	150.921	84.9057	115.65	231.325	188.679	134.937	65.0155
131.956907	145.745	179.228	123.531	252.492	132.106	84.9057	136.677	234.94	177.068	133.368	46.4396
142.7289006	145.745	168.199	116.604	238.769	143.202	96.2264	126.164	213.253	177.068	134.937	37.1517
133.3034163	127.899	170.956	124.108	228.875	153.654	110.377	128.266	184.337	178.52	131.799	9.28793
134.649914	142.771	181.985	130.746	225.047	157.916	93.3962	145.088	209.639	165.457	125.523	37.1517
145.4219076	148.72	166.82	125.84	240.358	169.092	107.547	126.164	198.795	179.971	145.921	74.3034
150.8078987	127.899	172.335	130.169	243.608	154.941	107.547	130.369	227.711	175.617	128.661	74.3034
146.7684054	142.771	170.956	116.604	224.469	156.388	90.566	109.342	234.94	181.422	117.678	18.5759
123.8779204	127.899	161.305	120.933	226.636	158.961	67.9245	121.958	202.41	184.325	134.937	83.5913
137.3429096	130.873	172.335	121.799	210.169	109.592	84.9057	111.444	209.639	166.909	134.937	55.7276
141.3824028	139.797	165.441	125.262	217.247	104.125	56.6038	142.985	253.012	179.971	147.49	37.1517
148.1149031	127.899	172.335	118.335	222.88	111.683	101.887	119.855	206.024	174.165	130.23	55.7276
142.7289006	130.873	166.82	120.356	219.63	110.718	90.566	132.472	216.867	158.2	139.644	65.0155
141.3824028	113.027	165.441	111.12	218.908	114.577	104.717	107.239	245.783	162.554	127.092	9.28793
137.3429096	116.002	162.684	112.851	225.553	116.99	90.566	157.704	209.639	181.422	149.059	65.0155
145.4219076	121.95	177.849	121.799	212.047	111.442	113.208	105.136	206.024	182.874	138.075	27.8638
142.7289006	139.797	166.82	118.047	227.575	108.869	87.7359	77.8008	234.94	165.457	128.661	46.4396
146.7684054	130.873	165.441	114.583	213.347	117.954	93.3962	103.034	202.41	164.006	130.23	46.4396
140.0359051	121.95	173.713	110.254	206.991	121.01	93.3962	111.444	213.253	179.971	136.506	55.7276
148.1149031	157.643	170.956	99.8634	233.858	132.186	104.717	109.959	216.867	178.52	127.092	37.1517
134.649914	130.873	168.199	96.9773	215.08	115.703	93.3962	109.959	213.253	168.36	134.937	46.4396
146.7684054	116.002	168.199	92.9365	223.603	107.18	87.7359	105.809	213.253	190.131	134.937	9.28793

131.956907	148.72	164.062	74.7533	219.052	105.009	90.566	126.556	198.795	169.811	131.799	46.4396
134.649914	139.797	177.849	80.2371	225.625	110.236	116.038	130.705	224.096	155.298	134.937	111.455
134.649914	127.899	180.607	72.7329	215.514	108.628	116.038	124.481	209.639	171.263	125.523	46.4396
130.6104093	136.822	193.015	81.6802	226.203	120.849	110.377	112.033	202.41	172.714	128.661	46.4396
129.2639115	124.925	186.121	84.855	225.625	125.432	84.9057	118.257	224.096	161.103	130.23	74.3034
127.9174137	118.976	198.529	73.5988	225.625	112.969	118.868	126.556	220.482	169.811	149.059	18.5759
131.956907	121.95	195.772	71.5785	213.997	117.874	113.208	107.884	209.639	172.714	120.816	55.7276
131.956907	136.822	194.393	79.0826	206.558	118.759	90.566	109.959	198.795	169.811	153.766	18.5759
127.9174137	116.002	190.257	76.7736	228.153	117.15	101.887	103.734	195.181	161.103	133.368	37.1517
131.956907	145.745	193.015	62.3425	210.025	125.834	96.2264	95.4357	166.265	153.846	127.092	37.1517
130.6104093	127.899	172.335	64.6515	211.252	131.221	93.3962	105.809	231.325	162.554	145.921	27.8638
134.649914	151.694	191.636	74.4646	210.963	114.095	101.887	107.884	191.566	174.165	134.937	65.0155
126.570916	139.797	179.228	54.8384	213.419	100.185	99.0566	118.257	184.337	164.006	120.816	9.28793
135.9964118	116.002	179.228	65.5174	218.186	124.387	101.887	118.257	209.639	169.811	141.213	18.5759
133.3034163	133.848	180.607	69.558	202.441	126.477	104.717	95.4357	191.566	178.52	127.092	27.8638
138.6894073	127.899	181.985	66.6718	201.286	127.603	99.0566	112.033	216.867	159.652	142.782	55.7276
125.2244182	127.899	173.713	67.2492	205.402	98.1748	101.887	114.108	234.94	161.103	149.059	46.4396
126.570916	121.95	175.092	66.6718	201.791	129.292	110.377	101.66	162.651	152.395	138.075	27.8638

MI+S107

88.4982	93.0974	111.994	93.3099	101.042	115.799	91.7266	101.798	101.695	107.503	97.8139
83.1347	90.4872	66.7196	102.113	103.243	99.6409	89.9281	98.5607	130.751	114.222	94.441
87.6043	80.0464	102.462	88.0282	86.9538	88.8689	95.3237	81.6549	108.959	94.065	111.305
96.5435	80.0464	97.6966	112.676	75.0661	78.0969	88.1295	105.035	123.487	100.784	114.678
96.5435	67.8654	126.291	66.9014	80.5692	99.6409	95.3237	88.1297	112.591	77.2676	111.305
98.3313	68.7355	133.439	84.507	89.375	110.413	93.5252	82.3747	116.223	70.5487	101.187
101.013	94.8376	100.079	114.437	103.904	99.6409	88.1295	69.7843	123.487	97.4244	106.246
104.589	96.5777	107.228	77.4648	93.1171	91.5619	84.5324	70.8631	79.9031	83.9866	94.441
96.5435	96.5777	92.9309	100.352	95.9788	110.413	102.518	116.546	127.119	100.784	99.5003
98.3313	88.7471	111.994	88.0282	100.822	88.8689	104.317	84.5324	127.119	80.6271	102.873
94.7557	93.0974	92.9309	114.437	108.526	91.5619	95.3237	109.353	119.855	131.019	106.246
97.4374	105.278	102.462	89.7887	107.206	91.5619	106.115	93.1657	65.3753	124.3	91.0681
112.634	82.6566	116.759	66.9014	110.288	75.404	89.9281	91.367	90.799	107.503	112.992
102.801	96.5777	76.251	114.437	113.149	105.027	95.3237	127.697	83.5351	110.862	91.0681
99.2253	80.0464	92.9309	91.5493	78.3683	105.027	98.9209	110.432	112.591	117.581	84.3223
103.695	88.7471	85.7824	98.5915	108.967	123.878	116.906	120.863	105.327	90.7055	101.187
108.164	98.3179	90.5481	114.437	101.262	91.5619	109.712	78.4167	98.063	100.784	106.246
98.3313	96.5777	90.5481	88.0282	100.161	91.5619	104.317	109.353	98.063	114.222	109.619
92.0739	86.1369	95.3137	110.915	108.746	86.1759	95.3237	66.9068	79.9031	67.1892	101.187
102.801	84.3967	95.3137	91.5493	89.815	96.9479	106.115	116.907	79.9031	110.862	101.187

104.589	93.0974	128.674	117.958	95.5383	107.72	113.309	126.979	61.7433	117.581	111.305
102.801	106.148	92.9309	147.887	99.9412	102.334	100.719	128.058	105.327	87.346	97.8139
95.6496	136.601	97.6966	88.0282	103.464	99.6409	97.1223	93.1657	98.063	100.784	91.0681
101.907	141.821	97.6966	116.197	116.451	99.6409	107.914	140.287	72.6392	90.7055	94.441
103.695	128.77	111.994	110.915	115.791	131.957	98.9209	87.7698	87.1671	87.346	74.2036
104.589	127.9	85.7824	98.5915	90.9157	99.6409	102.518	83.8134	83.5351	114.222	97.8139
102.801	119.2	109.611	86.2676	100.822	102.334	120.504	107.554	105.327	67.1892	94.441
101.907	129.64	83.3995	91.5493	93.9976	91.5619	89.9281	84.8923	65.3753	87.346	91.0681
112.634	131.381	104.845	119.718	107.206	134.65	97.1223	117.266	123.487	117.581	94.441
106.377	116.589	97.6966	114.437	119.313	88.8689	120.504	106.475	112.591	127.66	89.3816
197.239	232.309	176.33	235.915	281.553	199.282	257.194	184.716	261.291	255.319	148.407
205.602	268.91	200.159	241.197	273.848	215.44	239.209	188.747	282.767	272.116	145.034
208.284	249.71	197.776	242.958	271.646	212.747	260.791	225.397	200.442	275.476	158.526
213.647	254.06	212.073	242.958	252.715	188.51	246.403	242.256	236.235	262.038	153.467
217.223	268.852	200.159	237.676	252.495	210.054	262.59	204.507	229.077	295.633	153.467
226.162	253.19	185.862	220.07	258.439	164.273	246.403	196.443	236.235	312.43	153.467
226.162	316.676	178.713	209.507	246.992	199.282	262.59	237.858	239.815	268.757	134.916
222.586	288.37	204.925	216.549	277.37	188.51	255.396	237.858	214.759	278.835	141.661
218.117	301.914	204.925	225.352	267.244	177.738	262.59	182.883	211.18	272.116	153.467
223.48	292.343	200.159	220.07	258.879	164.273	244.604	200.108	207.601	245.241	150.094
227.95	284.513	200.159	221.831	263.942	177.738	242.806	211.47	178.966	262.038	151.78
221.692	280.162	209.69	246.479	239.727	185.817	255.396	226.496	182.546	251.96	150.094
221.692	266.241	200.159	248.239	250.734	177.738	251.799	204.14	243.341	204.927	150.094
223.48	283.063	197.776	230.634	247.212	177.738	242.806	197.909	181.598	238.522	133.229
219.011	269.794	188.245	223.592	251.835	172.352	260.791	184.716	199.758	241.881	153.467
219.011	262.718	190.627	214.789	268.564	164.273	244.604	199.742	185.23	251.96	134.916
214.541	261.833	197.776	209.507	260.86	180.431	242.806	210.737	170.702	228.443	150.094
227.95	256.526	188.245	223.592	259.099	153.501	244.604	175.553	148.91	225.084	139.975

226.162	241.488	190.627	197.183	214.852	201.975	235.612	199.009	228.814	248.6	143.348
234.207	230.873	190.627	200.704	241.048	153.501	232.014	177.019	199.758	218.365	136.602
225.268	247.1	188.245	209.507	250.954	180.431	269.784	230.528	174.334	258.679	148.407
235.101	247.1	197.776	198.944	257.778	188.51	250	161.993	185.23	231.803	172.017
193.981	226.218	185.862	207.746	246.111	150.808	235.612	153.196	203.39	241.881	131.543
202.026	227.958	195.393	207.746	258.658	153.501	285.971	145.867	199.758	231.803	139.975
231.526	221.868	202.542	205.986	263.942	180.431	275.18	160.072	185.23	231.803	150.094
244.041	227.088	202.542	221.831	259.099	183.124	284.173	173.381	196.126	282.195	143.348
245.828	237.529	200.159	221.831	269.445	175.045	293.165	206.474	268.765	272.116	151.78
265.495	236.659	197.776	198.944	248.973	193.896	271.583	221.223	254.237	278.835	134.916
245.828	227.958	197.776	202.465	271.427	177.738	275.18	176.259	261.501	258.679	146.721
244.041	225.348	209.69	193.662	256.017	188.51	284.173	235.971	254.237	275.476	145.034
237.783	225.348	204.925	207.254	250.073	156.194	287.77	256.183	236.077	255.319	153.467
232.42	221.868	183.479	214.221	209.569	204.668	302.158	236.758	196.863	258.679	141.661
230.632	208.817	204.925	195.063	238.406	172.352	269.784	219.899	204.021	258.679	146.721
230.632	197.506	200.159	195.063	253.595	169.659	271.583	194.978	218.339	282.195	150.094
227.95	214.066	178.713	195.063	249.193	175.045	302.158	254.351	182.546	265.398	145.034
221.692	200.798	171.565	193.321	238.186	172.352	294.964	244.089	225.497	275.476	136.602
219.905	205.22	178.713	175.905	237.526	166.966	280.576	216.968	250.553	241.881	143.348
210.965	197.259	176.33	188.096	254.036	137.343	278.777	250.719	272.029	215.006	141.661
202.92	202.567	178.713	182.871	242.368	191.203	284.173	244.604	254.132	231.803	136.602
208.284	181.337	185.862	179.388	248.312	175.045	291.367	235.612	246.973	208.287	138.289
224.374	191.952	173.948	175.905	247.431	180.431	305.755	210.792	279.661	215.006	138.289
211.859	206.99	154.885	175.905	245.231	153.501	300.36	217.266	283.293	231.803	138.289
206.496	215.835	190.627	165.455	269.445	172.352	294.964	229.496	214.286	238.522	128.17
215.435	220.258	204.925	214.221	260.42	166.966	307.554	223.021	250.605	235.162	139.975
223.48	179.568	188.245	229.896	264.823	175.045	307.554	248.561	257.869	248.6	128.17
215.435	191.952	183.479	202.029	277.59	185.817	300.36	248.561	217.918	255.319	121.424

211.859	180.452	207.307	200.288	263.722	166.966	314.748	215.467	217.918	248.6	128.17
215.435	187.529	185.862	215.962	257.118	177.738	289.568	252.877	217.918	262.038	136.602
210.072	196.636	200.159	195.063	278.691	164.273	303.957	249.64	272.397	248.6	150.094
211.859	204.466	200.159	198.546	271.207	169.659	327.338	239.928	228.814	221.725	146.721
211.859	195.766	185.862	207.254	262.621	161.58	341.727	271.942	214.286	295.633	134.916
219.011	163.573	166.799	182.871	274.729	161.58	330.935	271.942	250.605	278.407	129.856
219.011	149.652	171.565	168.938	260.64	166.966	330.935	224.46	228.814	254.64	129.856
210.072	188.414	171.565	165.455	258.218	177.738	354.317	199.28	207.022	271.616	138.289
219.011	194.606	200.159	165.455	256.897	185.817	312.95	251.799	225.182	258.035	133.229
218.117	185.76	181.096	189.838	283.754	175.045	329.137	223.741	221.55	230.874	139.975
205.602	177.799	181.096	172.422	283.094	185.817	316.547	264.029	196.126	241.059	116.365
202.026	185.76	193.01	179.388	263.061	158.887	309.353	251.439	210.654	241.059	139.975
183.254	199.246	202.542	189.838	277.59	188.51	320.144	205.755	207.022	241.059	124.797
215.406	200.986	200.159	200.288	261.08	164.273	294.964	237.769	225.182	237.664	131.543
214.541	197.506	226.37	196.804	266.584	183.124	311.151	210.791	217.918	264.826	123.111
214.541	190.545	195.393	236.862	287.056	172.352	303.957	275.18	196.126	224.083	150.094
212.811	197.506	193.01	205.513	264.603	191.203	291.367	251.798	141.646	200.317	139.975
211.946	196.636	185.862	191.58	276.93	169.659	307.554	276.259	193.283	200.317	138.289
222.327	199.246	188.245	186.355	278.471	169.659	314.748	232.734	246.973	203.712	134.916
225.787	197.506	200.159	195.063	275.169	140.036	323.741	229.496	232.656	196.922	133.229
218.001	192.285	188.245	184.613	274.728	164.273	316.547	234.892	189.704	207.107	134.916
219.732	194.026	159.651	191.58	286.396	188.51	318.345	229.137	189.704	179.946	139.975
220.597	195.766	171.565	153.264	286.616	177.738	323.741	251.439	243.394	207.107	141.661
219.732	194.896	159.651	177.647	293.66	166.966	321.942	226.978	214.759	224.083	118.051
222.327	218.387	159.651	141.072	280.232	140.036	309.353	259.352	225.497	190.131	133.229
216.271	191.415	166.799	163.713	299.164	169.659	305.755	218.345	225.497	213.898	141.661
221.462	172.274	154.885	175.905	280.892	166.966	316.547	231.295	204.021	210.502	139.975
209.351	174.884	159.651	161.972	287.936	145.422	307.554	258.632	177.966	203.712	126.483

211.081	196.375	195.393	156.747	299.824	156.194	303.957	198.561	221.55	210.502	143.348
211.081	195.49	200.159	163.713	299.604	150.808	321.942	229.496	217.918	207.107	138.289
198.97	222.027	231.136	160.23	282.213	164.273	296.763	245.324	232.446	200.317	124.797
202.43	213.182	243.05	170.68	278.251	150.808	323.741	211.511	236.077	159.574	114.678
204.16	220.258	221.604	177.647	281.333	166.966	327.338	217.626	163.438	193.526	141.661
202.43	215.835	212.073	186.355	284.194	183.124	329.137	243.885	232.446	203.712	134.916
207.62	200.798	159.651	195.063	270.546	188.51	320.144	252.158	203.39	203.712	123.111
155.715	231.439	223.987	189.838	269.005	158.887	336.331	265.108	228.814	186.736	123.111
186.83	221.868	245.433	233.379	261.961	175.045	350.719	251.079	210.654	193.526	133.229
194.875	221.868	221.604	181.13	243.249	204.668	348.921	231.295	199.758	179.946	116.365
222.586	221.868	200.159	177.647	242.809	196.589	347.122	205.036	239.709	190.131	139.975
237.783	214.037	223.987	177.647	254.916	180.431	321.942	204.317	174.334	179.946	128.17
253.874	200.986	219.222	181.338	243.91	201.975	250.842	233.812	177.966	173.155	114.678
246.722	208.817	212.073	114.437	256.677	183.124	230.847	209.353	170.702	162.97	124.797
245.828	220.998	209.69	109.155	252.715	169.659	269.019	235.971	207.022	169.76	138.289
246.722	223.608	195.393	117.958	248.312	175.045	261.748	207.553	181.598	169.76	146.721
246.722	215.777	200.159	128.521	242.369	161.58	281.743	233.093	167.07	166.365	121.424
234.207	214.037	188.245	149.648	245.01	188.51	307.19	215.828	188.862	166.365	129.856
235.101	217.517	188.245	130.282	251.394	185.817	294.467	226.259	239.709	176.55	129.856
231.526	218.387	173.948	137.324	251.174	148.115	307.19	229.137	177.966	176.55	138.289
215.435	209.687	209.69	137.324	245.231	175.045	314.461	216.906	232.446	169.76	133.229
216.329	210.557	204.925	132.042	250.734	164.273	301.737	259.352	177.966	162.97	123.111
219.905	210.557	176.33	188.38	251.174	156.194	294.467	220.863	254.237	159.574	128.17
209.178	206.207	188.245	142.606	249.193	180.431	292.649	245.683	228.814	179.946	131.543
214.541	147.042	193.01	132.042	252.275	164.273	276.29	253.956	188.862	173.155	124.797
199.344	182.222	171.565	140.845	249.413	166.966	298.102	240.647	228.814	135.808	131.543
191.299	214.066	190.627	153.169	263.282	153.501	299.92	223.741	236.077	166.365	136.602
187.723	203.451	178.713	209.507	249.193	169.659	305.373	187.05	257.869	169.76	128.17

216.329	220.258	162.033	133.803	245.891	183.124	343.544	235.971	203.39	159.574	138.289
210.072	208.759	171.565	140.845	258.879	169.659	310.826	252.517	228.814	166.365	128.17
214.541	211.412	178.713	169.014	251.835	172.352	298.102	200.36	214.286	159.574	134.916
209.178	219.374	164.416	133.803	250.073	169.659	316.279	231.655	210.654	179.946	129.856
212.753	207.874	152.502	135.563	250.073	158.887	314.461	230.216	207.022	176.55	146.721
215.435	201.682	162.033	125	248.973	156.194	301.737	192.779	188.862	176.55	118.051
204.708	187.065	162.033	133.803	251.614	188.51	303.555	191.68	276.029	125.622	129.856
206.496	207.947	159.651	142.606	245.891	156.194	299.92	204.14	246.973	176.55	133.229
209.178	226.45	154.885	154.93	256.677	148.115	307.19	211.103	228.814	156.179	119.738
201.132	222.027	169.182	161.972	258.439	158.887	301.737	242.622	250.605	183.341	151.78
198.451	210.528	162.033	126.761	261.08	164.273	307.19	245.554	217.918	139.203	131.543
188.617	214.907	157.268	140.845	260.86	158.887	290.831	203.773	207.022	166.365	119.738
193.981	205.336	164.416	139.085	244.79	137.343	289.013	255.45	301.453	149.389	139.975
202.43	203.596	164.416	154.93	234.884	156.194	299.92	198.642	228.814	162.97	118.051
199.835	209.643	147.736	140.845	225.638	201.975	274.472	196.443	239.709	166.365	143.348
194.644	211.412	142.971	144.366	239.947	193.896	274.472	205.24	239.709	169.76	148.407
191.184	192.836	162.033	100.352	213.531	228.905	279.925	234.193	228.814	162.97	134.916
192.914	194.606	142.971	140.845	233.563	204.668	278.107	220.266	225.182	200.317	136.602

MI+Prop

90.68279127	91.6767	99.6169	101.626	89.2926	94.5473	78.8136	94.3396	76.14213358	109.056	99.8703	101.146	96.5909	87.2657	89.1473	97.4244	112.966
86.415365	85.6454	107.28	85.3659	102.049	99.0495	94.0678	101.887	82.23350262	86.4925	94.6822	91.0317	88.0682	87.2657	89.1473	94.0649	85.5254
92.8165018	91.6767	88.1226	95.5285	90.4523	97.5488	96.6102	105.66	109.6446702	94.0136	93.3852	95.0775	85.2273	95.0226	73.6434	94.0649	78.1947
83.214794	101.327	61.3027	95.5285	88.133	96.048	99.1525	109.434	85.27918486	101.535	94.6822	89.0088	79.5455	93.0834	89.1473	97.4244	112.405
87.48221507	86.8516	107.28	73.1707	89.2926	103.552	101.695	94.3396	118.781726	112.816	95.9792	101.146	102.273	114.415	85.2713	94.0649	79.3163
104.5519201	77.2014	122.605	97.561	81.1751	109.555	94.0678	113.208	100.5076143	99.6544	90.7912	99.1234	119.318	85.3264	93.0233	100.784	129.79
108.8193412	104.946	107.28	103.659	95.0908	105.053	88.9831	90.566	82.23350262	95.8939	89.4942	91.0317	90.9091	104.719	124.031	94.0649	98.5445
103.4850649	100.121	95.7854	105.691	96.2505	105.053	83.8983	109.434	82.23350262	90.2531	95.9792	99.1234	102.273	96.9619	85.2713	94.0649	78.1947
99.21764381	96.5018	95.7854	99.5935	93.9312	99.0495	99.1525	101.887	100.5076143	103.415	97.2763	86.9858	93.75	106.658	85.2713	107.503	80.6383
101.3513543	98.9144	137.931	99.5935	109.007	93.0465	101.695	98.1132	103.5532966	112.816	94.6822	93.0546	107.955	102.78	89.1473	94.0649	73.3075
108.8193412	96.5018	91.954	111.789	76.5365	102.051	104.237	101.887	121.8274082	94.0136	93.3852	99.1234	102.273	87.2657	89.1473	114.222	117.292
104.5519201	102.533	95.7854	105.691	102.049	109.555	101.695	109.434	94.4162407	112.816	93.3852	101.146	88.0682	83.3872	85.2713	90.7055	46.4281
103.4850649	115.802	84.2912	85.3659	111.326	102.051	73.7288	116.981	82.23350262	101.535	101.167	89.0088	105.114	106.658	65.8915	94.0649	62.4917
97.08392287	107.358	107.28	99.5935	111.326	108.054	114.407	94.3396	97.46193208	77.0912	93.3852	93.0546	99.4318	98.9011	108.527	100.784	122.58
116.2873385	98.9144	107.28	87.3984	93.9312	102.051	114.407	64.1509	112.6903524	90.2531	99.8703	101.146	125	93.0834	124.031	97.4244	129.51
99.21764381	123.04	103.448	109.756	99.7294	106.553	106.78	98.1132	91.37055847	105.295	97.2763	103.169	93.75	100.84	65.8915	90.7055	95.2998
106.6856307	106.152	95.7854	101.626	103.208	99.0495	94.0678	105.66	94.4162407	80.8517	90.7912	109.238	99.4318	104.719	96.8992	97.4244	92.8562
94.95021234	107.358	95.7854	99.5935	102.049	97.5488	114.407	113.208	76.14213358	88.3728	102.464	91.0317	122.159	114.415	116.279	90.7055	85.5254
102.4182096	101.327	111.111	107.724	95.0908	99.0495	106.78	98.1132	100.5076143	112.816	105.058	95.0775	119.318	139.625	112.403	107.503	141.728
98.15077814	102.533	107.28	99.5935	106.687	109.555	101.695	120.755	88.32487623	103.415	102.464	103.169	122.159	81.448	120.155	87.346	134.397
99.21764381	96.5018	103.448	97.561	103.208	93.0465	96.6102	94.3396	115.7360438	90.8883	103.761	121.376	99.4318	96.9619	108.527	107.503	134.397
97.08392287	102.533	107.28	111.789	93.9312	85.5428	96.6102	105.66	106.5989879	90.8883	114.137	93.0546	102.273	93.0834	77.5194	94.0649	127.387
96.0170676	95.2955	99.6169	103.659	109.007	109.555	106.78	109.434	115.7360438	98.3078	103.761	103.169	76.7045	102.78	116.279	100.784	93.7375
102.4182096	97.7081	107.28	99.5935	103.208	96.048	96.6102	98.1132	106.5989879	107.582	105.058	113.284	99.4318	108.597	112.403	114.222	92.8562
99.21764381	103.739	99.6169	117.886	106.687	93.0465	106.78	101.887	100.5076143	105.727	114.137	99.1234	133.523	137.686	108.527	114.222	58.646
106.6856307	110.977	68.9655	103.659	103.208	102.051	104.237	94.3396	112.6903524	120.566	108.949	101.146	79.5455	87.2657	116.279	110.862	110.562
100.2844991	104.946	114.943	105.691	109.007	103.552	94.0678	94.3396	103.5532966	98.3078	97.2763	105.192	82.3864	106.658	112.403	107.503	134.597
96.0170676	100.121	103.448	101.626	103.208	97.5488	122.034	113.208	130.9644641	107.582	112.84	117.33	93.75	108.597	120.155	100.784	91.334
97.08392287	89.2642	99.6169	109.756	114.805	93.0465	99.1525	67.9245	100.5076143	98.3078	105.058	103.169	90.9091	96.9619	124.031	104.143	112.966
116.2873385	102.533	72.7969	83.3333	117.124	88.5443	106.78	79.2453	106.5989879	109.437	108.949	111.261	99.4318	77.5695	116.279	107.503	86.5269
211.2375508	199.035	264.206	221.545	208.986	154.577	251.695	240.533	127.9187819	196.616	169.909	232.637	178.977	281.189	305.761	265.398	232.14

192.0341352	193.004	237.008	203.252	189.465	181.427	259.322	225.261	158.3756408	209.6	159.533	236.682	193.182	263.736	266.561	228.443	259.02
204.8364192	196.622	229.237	184.959	181.428	175.479	254.237	225.261	191.8781728	193.668	159.533	236.682	198.864	244.344	262.641	211.646	217.479
194.1678457	174.91	229.237	191.057	177.983	172.504	233.898	179.445	216.243649	212.471	160.83	228.591	187.5	242.405	266.561	184.77	249.246
183.4992931	200.241	248.664	209.35	188.317	168.043	216.102	167.991	201.0152286	224.438	147.86	206.339	144.886	230.769	274.401	218.365	173.494
192.0341352	180.941	233.123	203.252	163.055	176.966	233.898	198.535	228.4263962	220.729	151.751	224.545	187.5	242.405	258.721	194.849	285.899
177.0981615	180.941	229.237	199.187	174.538	191.837	246.61	194.717	213.1979759	218.874	151.751	194.201	210.227	223.012	250.881	184.77	171.051
181.3655825	189.385	233.123	184.959	186.021	182.914	244.068	206.171	222.3350317	233.713	158.236	182.063	196.023	244.344	250.881	208.287	261.463
178.1650167	188.179	198.154	178.862	179.131	179.94	246.61	206.171	225.3807048	226.293	162.127	194.201	204.545	213.316	262.641	164.614	210.148
170.6970091	190.591	237.008	193.089	172.241	185.888	238.983	209.989	201.0152286	226.293	154.345	200.27	187.5	232.708	250.881	178.052	180.825
173.8975853	185.766	209.811	174.797	180.279	173.992	238.983	194.717	188.8324814	217.019	154.345	192.178	176.136	248.222	270.481	188.13	193.043
185.6330036	190.591	248.664	162.602	179.131	176.966	238.983	206.171	213.1979759	218.874	156.939	220.499	159.091	226.891	266.561	191.489	266.351
172.83073	184.56	252.55	203.252	182.576	176.966	241.525	209.989	216.243649	224.438	150.454	222.522	181.818	217.195	250.881	201.568	215.035
179.231872	184.56	237.008	195.122	175.686	181.427	241.525	198.535	173.6040611	217.019	151.751	188.132	176.136	234.648	270.481	188.13	217.479
184.5661483	194.21	225.352	172.764	175.686	181.427	226.271	183.263	207.1065931	231.858	151.751	198.247	178.977	232.708	274.401	211.646	217.479
192.0341352	194.21	217.581	166.667	179.131	173.992	228.814	187.081	201.0152286	189.196	149.157	188.132	164.773	224.952	227.361	171.333	217.479
172.83073	196.622	217.581	184.959	175.686	181.427	211.017	175.627	207.1065931	209.6	153.048	192.178	170.455	224.952	235.201	198.208	215.035
192.0341352	195.416	217.581	186.992	177.983	176.966	228.814	202.353	210.1522845	215.164	142.672	184.086	190.341	217.195	235.201	191.489	215.035
167.4964433	189.385	237.008	199.187	184.872	179.94	236.441	187.081	197.9695373	213.309	151.751	194.201	181.818	215.255	274.401	198.208	190.6
172.83073	190.591	213.696	197.154	184.872	169.53	221.186	190.899	182.7411169	202.18	150.454	165.88	190.341	234.648	274.401	215.006	210.148
168.5632985	186.972	237.008	199.187	197.503	165.069	231.356	164.173	201.0152286	217.019	145.266	186.109	156.25	240.465	282.241	198.208	227.253
165.3627327	177.322	205.925	189.024	188.317	175.479	244.068	209.989	191.8781728	213.309	145.266	182.063	176.136	236.587	278.321	194.849	222.366
171.7638747	183.353	229.237	195.122	186.021	172.504	231.356	167.991	197.9695373	211.454	147.86	192.178	167.614	240.465	278.321	167.973	166.164
164.2958775	188.179	221.467	180.894	199.8	172.504	228.814	167.991	197.9695373	211.454	150.454	180.04	170.455	224.952	282.241	201.568	139.284
179.231872	176.116	213.696	180.894	202.096	171.017	241.525	187.081	185.7868083	209.6	147.86	188.132	187.5	217.195	266.561	191.489	210.148
169.6301538	171.291	217.581	182.927	189.465	168.043	244.068	187.081	197.9695373	207.745	154.345	186.109	190.341	244.344	286.161	204.927	273.681
172.83073	173.703	221.467	178.862	189.465	169.53	228.814	171.809	197.9695373	207.745	162.127	184.086	181.818	228.83	274.401	208.287	234.584
167.4964433	172.497	229.237	191.057	192.91	159.12	223.729	156.537	185.7868083	192.906	151.751	169.926	176.136	221.073	278.321	204.927	175.938
180.2987273	176.116	194.269	182.927	192.91	150.198	233.898	175.627	185.7868083	204.035	151.751	188.132	181.818	217.195	282.241	167.973	252.37

168.5632985	170.084	209.811	182.927	195.207	163.582	216.102	152.719	197.9695373	220.729	159.533	184.086	164.773	221.073	305.761	208.287	278.809
182.4324378	164.053	221.467	191.057	189.465	147.224	216.102	145.083	204.06092	209.6	151.751	188.132	181.818	193.924	282.241	194.849	259.02
185.6330036	176.116	213.696	186.992	189.465	153.172	221.186	167.991	182.7411169	213.309	140.078	184.086	176.136	205.559	262.641	181.411	161.277
161.0953013	155.609	221.467	172.764	188.317	145.736	241.525	190.899	179.6954256	213.309	145.266	184.086	176.136	236.587	278.321	201.568	219.923
174.9644301	170.084	225.352	172.764	189.465	150.198	238.983	167.991	170.5583697	204.035	147.86	188.132	173.295	221.073	282.241	194.849	229.697
162.1621669	165.259	237.008	182.927	203.245	153.172	231.356	167.991	188.8324814	194.761	136.187	190.155	184.659	275.372	282.241	215.006	183.269
177.0981615	176.116	213.696	201.22	194.059	160.608	218.644	148.901	146.1928935	207.745	145.266	192.178	181.818	223.012	286.161	184.77	229.697
180.2987273	159.228	209.811	178.862	187.169	154.659	231.356	152.719	155.3299494	204.035	151.751	190.155	210.227	190.045	266.561	198.208	268.794
172.83073	171.291	209.811	172.764	203.245	156.146	241.525	187.081	173.6040611	196.616	146.563	190.155	173.295	193.924	258.721	235.162	190.6
181.3655825	164.053	209.811	186.992	180.279	153.172	221.186	160.355	179.6954256	205.89	143.969	182.063	153.409	215.255	294.001	174.692	188.156
173.8975853	164.053	205.925	164.634	198.652	156.146	264.407	206.171	152.2842672	196.616	151.751	180.04	150.568	211.377	309.681	191.489	207.705
171.7638747	170.084	209.811	186.992	196.355	144.249	246.61	179.445	170.5583697	200.325	147.86	184.086	136.364	190.045	239.121	188.13	229.697
160.028446	151.99	170.957	178.862	199.8	142.762	223.729	141.265	185.7868083	194.761	145.266	186.109	144.886	254.04	274.401	208.287	197.93
173.8975853	168.878	186.498	189.024	203.245	147.224	233.898	167.991	158.3756408	187.341	145.266	196.224	153.409	219.134	270.481	194.849	241.915
169.6301538	165.259	209.811	182.927	205.541	142.762	218.644	156.537	167.5126966	161.373	150.454	214.43	176.136	201.681	258.721	188.13	266.351
155.7610249	178.528	213.696	178.862	207.838	144.249	223.729	156.537	179.6954256	187.341	154.345	200.27	187.5	223.012	258.721	181.411	207.705
170.6970091	176.116	194.269	186.992	181.428	142.762	241.525	175.627	188.8324814	185.486	141.375	200.27	167.614	221.073	278.321	194.849	232.14
172.83073	162.847	186.498	182.927	200.948	142.762	228.814	164.173	170.5583697	198.47	147.86	210.384	170.455	215.255	258.721	191.489	197.93
169.6301538	176.116	190.384	170.732	212.431	150.198	244.068	179.445	137.0558377	200.325	141.375	194.201	170.455	159.017	297.921	191.489	156.389
169.6301538	159.228	182.613	182.927	203.245	151.685	221.186	148.901	149.2385758	196.616	143.969	206.339	164.773	207.498	235.201	194.849	217.479
171.7638747	167.672	213.696	158.537	197.503	153.172	246.61	179.445	179.6954256	191.051	143.969	202.293	122.159	205.559	243.041	188.13	285.899
162.1621669	165.259	213.696	152.439	197.503	154.659	221.186	171.809	140.1015199	198.47	151.751	188.132	161.932	205.559	258.721	188.13	153.946
165.3627327	155.609	221.467	172.764	195.207	153.172	244.068	187.081	179.6954256	196.616	141.375	196.224	147.727	209.438	219.521	184.77	215.035
167.4964433	155.609	202.04	164.634	191.762	144.249	231.356	167.991	173.6040611	202.18	146.563	186.109	164.773	213.316	223.441	188.13	266.351
166.429588	150.784	225.352	154.472	198.652	130.865	254.237	187.081	140.1015199	194.761	146.563	198.247	161.932	232.708	231.281	181.411	261.463
153.6273144	142.34	217.581	176.829	188.317	138.301	231.356	171.809	146.1928935	176.212	146.563	192.178	159.091	226.891	211.681	161.254	278.569
156.8278802	144.753	202.04	158.537	199.8	138.301	246.61	190.899	152.2842672	185.486	155.642	196.224	193.182	201.681	262.641	204.927	197.93
155.7610249	139.928	202.04	160.569	207.838	139.788	233.898	183.263	167.5126966	183.631	145.266	196.224	156.25	213.316	223.441	188.13	207.705

155.7610249	143.546	190.384	170.732	203.245	136.814	226.271	160.355	167.5126966	189.196	138.781	204.316	153.409	219.134	219.521	191.489	276.125
164.2958775	148.372	205.925	170.732	208.986	138.301	231.356	190.899	185.7868083	172.502	146.563	178.018	156.25	209.438	219.521	201.568	207.705
157.8947355	141.134	217.581	166.667	195.207	136.814	233.898	179.445	161.4213139	179.922	145.266	198.247	153.409	223.012	203.841	174.692	202.817
158.9615907	132.69	194.269	152.439	204.393	139.788	233.898	148.901	155.3299494	187.341	146.563	196.224	181.818	203.62	215.601	157.895	315.222
157.8947355	130.277	217.581	146.341	217.024	138.301	246.61	171.809	164.4670052	187.341	142.672	194.201	187.5	215.255	227.361	198.208	210.148
164.2958775	136.309	209.811	162.602	200.948	142.762	264.407	209.989	146.1928935	187.341	150.454	200.27	164.773	217.195	215.601	184.77	215.035
158.9615907	125.452	198.154	156.504	194.059	136.814	256.78	202.353	158.3756408	192.906	136.187	194.201	173.295	252.101	207.761	188.13	183.269
152.5604591	137.515	194.269	164.634	207.838	145.736	218.644	145.083	149.2385758	204.035	146.563	198.247	153.409	263.736	207.761	194.849	237.028
152.5604591	136.309	190.384	170.732	171.093	151.685	251.695	183.263	164.4670052	192.906	140.078	192.178	190.341	223.012	192.081	191.489	278.569
162.1621669	121.834	205.925	142.276	166.5	145.736	226.271	145.083	146.1928935	189.196	138.781	204.316	193.182	223.012	199.921	194.849	273.681
148.2930277	136.309	205.925	142.276	163.055	144.249	211.017	133.629	161.4213139	207.745	146.563	198.247	181.818	207.498	192.081	225.084	193.043
163.2290222	126.659	198.154	158.537	164.203	141.275	238.983	148.901	158.3756408	196.616	142.672	206.339	153.409	244.344	164.641	221.725	227.253
144.0256066	138.721	202.04	148.374	161.907	144.249	238.983	171.809	158.3756408	196.616	145.266	210.384	144.886	236.587	152.881	188.13	232.14
162.1621669	142.34	198.154	160.569	187.169	144.249	236.441	152.719	185.7868083	198.47	142.672	214.43	142.045	228.83	199.921	174.692	256.576
146.1593171	125.452	205.925	168.699	177.983	150.198	236.441	167.991	170.5583697	183.631	146.563	204.316	178.977	164.835	235.201	201.568	283.456
154.6941697	133.896	194.269	152.439	181.428	142.762	244.068	160.355	143.1472113	179.922	133.593	206.339	173.295	221.073	199.921	151.176	202.817
161.0953013	150.784	182.613	144.309	198.652	139.788	261.864	183.263	149.2385758	189.196	137.484	198.247	176.136	219.134	207.761	194.849	197.93
156.8278802	138.721	198.154	144.309	191.762	145.736	221.186	141.265	164.4670052	211.454	137.484	202.293	196.023	182.288	199.921	181.411	217.479
145.0924619	137.515	190.384	142.276	194.059	142.762	236.441	175.627	146.1928935	228.148	136.187	194.201	156.25	221.073	184.241	174.692	251.689
162.1621669	156.815	182.613	156.504	194.059	142.762	236.441	164.173	161.4213139	189.196	136.187	208.361	125	221.073	215.601	174.692	210.148
165.3627327	151.99	178.728	148.374	206.69	133.84	244.068	171.809	140.1015199	198.47	151.751	206.339	156.25	203.62	192.081	164.614	273.681
173.8975853	158.022	194.269	174.797	203.245	139.788	238.983	137.447	124.8730996	204.035	140.078	216.453	150.568	174.531	223.441	174.692	188.156
170.6970091	162.847	205.925	154.472	190.614	136.814	241.525	167.991	140.1015199	191.051	151.751	210.384	144.886	199.741	192.081	174.692	256.576
162.1621669	165.259	186.498	174.797	196.355	141.275	244.068	167.991	176.6497525	170.647	142.672	198.247	122.159	246.283	188.161	171.333	175.938
162.1621669	164.053	202.04	138.211	191.762	136.814	238.983	160.355	170.5583697	196.616	140.078	210.384	139.205	238.526	203.841	181.411	256.576
169.6301538	144.753	186.498	154.472	191.762	144.249	238.983	171.809	167.5126966	183.631	140.078	210.384	144.886	215.255	192.081	178.052	185.712
164.2958775	144.753	221.467	160.569	205.541	126.404	254.237	167.991	158.3756408	189.196	151.751	214.43	133.523	213.316	192.081	164.614	200.374
151.4936039	161.641	151.53	142.276	190.614	138.301	238.983	152.719	170.5583697	207.745	141.375	210.384	122.159	236.587	199.921	161.254	205.261

162.1621669	143.546	202.04	162.602	195.207	124.917	236.441	141.265	170.5583697	187.341	132.296	210.384	142.045	211.377	184.241	157.895	207.705
149.3598829	148.372	170.957	150.407	206.69	135.327	226.271	148.901	164.4670052	192.906	143.969	208.361	144.886	215.255	223.441	161.254	298.117
160.028446	159.228	170.957	148.374	200.948	144.249	221.186	145.083	152.2842672	191.051	138.781	200.27	153.409	217.195	203.841	164.614	283.456
158.9615907	149.578	186.498	168.699	206.69	132.353	249.153	175.627	164.4670052	200.325	137.484	188.132	164.773	213.316	203.841	167.973	215.035
151.4936039	162.847	190.384	168.699	203.245	130.865	241.525	160.355	164.4670052	198.47	128.405	208.361	142.045	215.255	211.681	161.254	244.358
164.2958775	156.815	198.154	168.699	208.986	133.84	251.695	187.081	170.5583697	196.616	138.781	206.339	156.25	213.316	196.001	164.614	234.584
156.8278802	156.815	186.498	176.829	221.617	135.327	221.186	194.717	155.3299494	207.745	133.593	210.384	119.318	213.316	199.921	164.614	180.825
153.6273144	160.434	163.186	174.797	210.134	133.84	249.153	148.901	191.8781728	198.47	133.593	206.339	142.045	217.195	192.081	157.895	237.028
155.7610249	159.228	174.842	176.829	211.283	139.788	233.898	183.263	197.9695373	196.616	130.999	210.384	142.045	215.255	219.521	161.254	139.284
166.429588	171.291	167.071	178.862	211.283	141.275	259.322	179.445	167.5126966	198.47	134.89	214.43	136.364	215.255	156.801	184.77	228.335
152.5604591	171.291	182.613	184.959	211.283	141.275	246.61	156.537	173.6040611	196.616	137.484	194.201	139.205	236.587	211.681	174.692	194.686
157.8947355	161.641	205.925	178.862	189.465	145.736	261.864	171.809	185.7868083	183.631	136.187	206.339	133.523	211.377	192.081	161.254	302.844
148.2930277	162.847	170.957	174.797	207.838	148.711	266.949	175.627	143.1472113	187.341	138.781	192.178	136.364	199.741	199.921	167.973	225.931
146.1593171	162.847	194.269	162.602	202.096	151.685	256.78	187.081	176.6497525	200.325	143.969	210.384	147.727	211.377	196.001	181.411	274.002
170.6970091	151.99	182.613	170.732	206.69	141.275	228.814	206.171	167.5126966	191.051	151.751	228.591	127.841	244.344	188.161	164.614	201.896
157.8947355	168.878	186.498	172.764	195.207	139.788	238.983	206.171	167.5126966	194.761	140.078	220.499	116.477	182.288	184.241	167.973	221.124
157.8947355	153.197	147.644	168.699	196.355	139.788	264.407	145.083	179.6954256	192.906	138.781	210.384	122.159	205.559	207.761	174.692	218.721
152.5604591	171.291	186.498	176.829	215.876	147.224	236.441	175.627	179.6954256	213.309	140.078	208.361	127.841	191.984	192.081	164.614	225.931
160.028446	155.609	182.613	178.862	174.538	147.224	256.78	209.989	164.4670052	200.325	136.187	230.614	122.159	199.741	192.081	167.973	225.931
145.0924619	162.847	182.613	180.894	198.652	141.275	266.949	171.809	158.3756408	215.164	133.593	214.43	116.477	209.438	199.921	154.535	173.054
161.0953013	165.259	190.384	154.472	195.207	138.301	251.695	175.627	146.1928935	213.309	137.484	182.063	96.5909	219.134	184.241	184.77	223.528
168.5632985	155.609	194.269	154.472	219.321	139.788	241.525	213.807	155.3299494	189.196	137.484	216.453	107.955	213.316	160.721	167.973	216.317
149.3598829	151.99	155.415	154.472	211.283	130.865	238.983	190.899	155.3299494	200.325	125.811	206.339	102.273	213.316	188.161	157.895	173.054
158.9615907	142.34	167.071	146.341	213.579	138.301	226.271	171.809	155.3299494	168.793	137.484	216.453	93.75	201.681	184.241	147.816	177.861
155.7610249	160.434	167.071	162.602	204.393	133.84	233.898	183.263	161.4213139	192.906	137.484	218.476	105.114	197.802	207.761	167.973	247.563
162.1621669	159.228	182.613	176.829	207.838	142.762	233.898	167.991	158.3756408	181.777	136.187	208.361	105.114	215.255	184.241	157.895	187.475
156.8278802	171.291	186.498	164.634	207.838	142.762	241.525	167.991	161.4213139	166.938	129.702	216.453	102.273	217.195	184.241	154.535	221.124
162.1621669	160.434	190.384	168.699	202.096	144.249	236.441	167.991	167.5126966	187.341	140.078	208.361	85.2273	193.924	188.161	171.333	269.195

165.3627327	159.228	178.728	174.797	197.503	145.736	236.441	183.263	146.1928935	176.212	141.375	222.522	76.7045	199.741	203.841	154.535	242.756
162.1621669	153.197	190.384	172.764	198.652	141.275	256.78	164.173	164.4670052	187.341	136.187	212.407	88.0682	197.802	172.481	141.097	194.686
156.8278802	156.815	132.103	180.894	198.652	144.249	236.441	175.627	152.2842672	181.777	127.108	208.361	99.4318	201.681	180.321	164.614	225.931
151.4936039	150.784	217.581	160.569	194.059	138.301	246.61	206.171	146.1928935	168.793	127.108	210.384	82.3864	205.559	184.241	164.614	300.441
150.4267382	165.259	170.957	152.439	198.652	141.275	238.983	164.173	161.4213139	176.212	136.187	178.018	79.5455	201.681	160.721	157.895	199.493
156.8278802	150.784	178.728	158.537	186.021	145.736	231.356	190.899	176.6497525	168.793	127.108	171.949	90.9091	217.195	172.481	161.254	240.353
155.7610249	161.641	170.957	144.309	194.059	151.685	236.441	183.263	152.2842672	191.051	133.593	182.063	105.114	191.984	160.721	157.895	266.791

MI+SD-208

89.33	80.9717	89.5522	96.3773	93.2358	114.202	91.2095	97.5395	106.047	98.4849	97.5779	102.484	101.329	90.6149	82.5226	95.4712
111.663	117.409	106.774	108.681	115.174	113.104	83.2783	105.448	86.5116	68.1818	97.5779	116.46	89.4718	93.2039	110.27	89.9633
91.8114	97.166	103.33	100.478	104.205	107.613	92.2009	118.629	108.837	75.7576	101.73	107.143	103.485	98.3819	91.5315	75.2754
116.625	80.9717	92.9966	102.529	109.689	109.81	92.2009	105.448	114.419	94.697	91.3495	83.8509	101.329	77.6699	94.054	78.9474
91.8114	105.263	99.8852	96.3773	95.9781	113.104	92.2009	105.448	117.209	106.061	101.73	88.5093	115.343	82.8479	86.8468	84.4553
96.7742	89.0688	92.9966	102.529	95.9781	87.8477	93.1923	108.084	86.5116	94.697	116.263	102.484	83.004	75.0809	100.18	86.2913
86.8486	80.9717	92.9966	100.478	76.7825	106.515	96.1666	108.084	72.5581	102.273	99.654	135.093	95.9396	121.683	106.667	77.1114
81.8859	113.36	96.4409	96.3773	101.463	105.417	98.1494	105.448	114.419	90.9091	93.4256	116.46	88.3938	82.8479	90.8108	128.519
91.8114	93.1174	120.551	108.681	106.947	105.417	102.115	105.448	83.7209	98.4849	91.3495	60.559	102.407	88.0259	97.2973	95.4712
96.7742	101.215	61.9977	90.2256	93.2358	110.908	107.072	97.5395	86.5116	71.9697	93.4256	88.5093	95.9396	113.916	107.387	91.7993
81.8859	105.263	82.6636	77.9221	104.205	117.496	102.115	121.265	78.1395	79.5455	122.491	79.1925	94.8617	95.7929	110.991	84.4553
89.33	105.263	99.8852	100.478	120.658	101.025	87.2439	115.993	89.3023	94.697	112.111	93.1677	92.7057	98.3819	110.27	53.2436
96.7742	101.215	96.4409	106.63	93.2358	103.221	99.1408	108.084	120	98.4849	107.958	102.484	89.4718	93.2039	88.2883	84.4553
94.2928	89.0688	99.8852	102.529	115.174	103.221	98.1494	102.812	100.465	121.212	114.187	93.1677	102.407	95.7929	114.955	97.3072
84.3672	105.263	134.328	112.782	131.627	80.1611	92.2009	108.084	111.628	128.788	101.73	125.776	94.8617	111.327	104.144	126.683
104.218	89.0688	137.773	104.58	71.298	84.5534	104.098	100.176	122.791	125	80.9689	79.1925	97.0176	113.916	107.387	95.4712
104.218	101.215	75.775	92.2761	90.4936	97.7306	105.089	86.9947	117.209	109.848	99.654	111.801	98.0956	98.3819	89.009	82.6193
99.2556	105.263	75.775	77.9221	98.7203	90.0439	106.081	102.812	78.1395	109.848	124.567	97.8261	91.6277	121.683	109.189	91.7993
101.737	76.9231	120.551	110.731	101.463	86.7496	99.1408	92.2671	89.3023	113.636	85.1211	79.1925	104.563	103.56	105.946	130.355
126.551	117.409	103.33	104.58	87.7514	93.3382	102.115	81.7223	94.8837	106.061	95.5017	130.435	109.953	121.683	109.55	88.1273
89.33	97.166	106.774	100.478	106.947	88.9458	93.1923	76.4499	86.5116	106.061	99.654	125.776	99.1736	95.7929	120	119.339
119.107	109.312	103.33	104.58	82.2669	102.123	93.1923	81.7223	92.093	102.273	74.7405	102.484	105.641	113.916	89.7296	139.535
101.737	125.506	99.8852	92.2761	109.689	93.3382	112.029	79.0861	97.6744	87.1212	101.73	97.8261	108.875	108.738	98.0179	123.011
116.625	101.215	96.4409	106.63	106.947	103.221	112.029	102.812	94.8837	102.273	97.5779	102.484	114.265	103.56	90.09	128.519
111.663	72.8745	103.33	98.4279	98.7203	102.123	109.055	100.176	111.628	102.273	93.4256	107.143	94.8617	113.916	105.225	119.339
116.625	133.603	89.5522	96.3773	109.689	104.319	109.055	102.812	106.047	109.848	105.882	51.2422	94.8617	111.327	87.2073	82.6193
99.2556	76.9231	99.8852	102.529	87.7514	88.9458	106.081	84.3585	111.628	109.848	101.73	125.776	113.187	113.916	109.549	110.159
99.2556	113.36	110.218	108.681	95.9781	98.8287	107.072	110.721	86.5116	117.424	97.5779	102.484	109.953	103.56	98.0181	115.667
106.7	113.36	113.662	104.58	98.7203	95.5344	113.02	94.9033	108.837	68.1818	91.3495	102.484	99.1736	85.4369	80	99.1432
101.737	101.215	92.9966	92.2761	95.9781	91.142	102.115	89.6309	125.581	106.061	107.958	88.5093	107.797	108.738	104.865	124.847
205.955	254.19	197.176	156.998	191.956	230.6	258.757	239.895	309.767	276.515	267.82	149.068	238.232	223.209	148.523	414.933

220.844	184.492	156.381	133.971	230.347	262.021	243.886	239.895	301.395	246.212	298.962	177.019	231.764	199.575	158.4	372.705
210.918	213.191	173.379	167.464	202.925	258.759	241.223	237.258	315.349	242.424	282.353	204.969	244.7	202.201	140.475	394.737
218.362	217.291	186.977	169.557	186.472	260.934	234.387	237.258	295.814	261.364	276.125	177.019	224.218	194.323	163.888	374.541
198.511	213.191	183.578	173.744	186.472	260.934	245.129	247.803	290.233	280.303	269.896	218.944	237.154	186.445	145.597	335.985
193.548	196.792	183.578	163.278	202.925	257.672	233.41	234.622	326.512	272.727	274.048	223.602	246.856	181.193	151.815	335.985
203.474	225.491	173.379	177.931	202.925	260.934	224.62	224.077	315.349	219.697	284.429	237.578	250.09	215.331	142.67	335.985
208.437	229.591	166.58	171.651	219.378	258.759	218.761	234.622	315.349	257.576	286.505	237.578	230.686	186.445	158.4	385.557
203.474	221.391	197.176	173.744	211.152	259.847	229.015	231.986	301.395	231.061	284.429	232.919	256.558	204.827	146.328	358.017
223.325	204.992	193.776	175.837	202.925	248.974	235.955	218.805	298.605	246.212	269.896	251.553	254.402	191.697	150.718	365.361
208.437	204.992	197.176	163.278	183.729	218.521	229.015	239.895	284.651	250	240.83	242.236	245.778	178.567	134.622	365.361
225.806	209.091	200.576	177.931	186.472	250.062	230.998	218.805	287.442	246.212	263.668	228.261	244.7	207.453	141.573	350.673
205.955	192.692	203.975	171.651	191.956	262.021	209.187	231.986	301.395	250	274.048	223.602	249.012	191.697	144.499	370.869
210.918	196.792	197.176	175.837	222.121	273.981	204.23	226.714	301.395	265.152	253.287	228.261	254.402	194.323	114.502	348.837
181.141	221.391	193.776	167.464	200.183	262.021	201.256	216.169	293.023	227.273	271.972	214.286	246.856	162.811	135.719	317.625
200.993	217.291	200.576	171.651	202.925	273.981	197.29	213.533	284.651	257.576	278.201	237.578	240.388	196.949	153.279	308.446
186.104	241.89	180.178	165.371	216.636	280.504	201.256	187.17	270.698	265.152	263.668	195.652	241.466	189.071	144.865	299.266
183.623	237.79	186.977	163.278	186.472	268.544	187.376	218.805	298.605	238.636	242.907	237.578	242.544	199.575	127.671	304.774
208.437	188.592	180.178	163.278	235.832	258.759	192.333	200.351	290.233	227.273	249.135	232.919	243.622	173.315	124.745	291.922
183.623	225.491	180.178	163.278	194.698	257.672	207.204	213.533	293.023	242.424	259.516	218.944	228.53	175.941	150.352	297.43
208.437	221.391	180.178	159.091	194.698	259.847	215.135	221.441	284.651	242.424	232.526	214.286	229.608	189.071	137.914	334.735
171.216	188.592	163.18	159.091	227.605	272.893	214.144	216.169	315.349	223.485	244.983	186.335	219.907	189.071	137.914	311.213
181.141	184.492	186.977	154.904	208.41	271.23	207.204	224.077	295.814	227.273	274.048	204.969	224.218	194.323	113.039	331.116
178.66	184.492	190.377	163.278	202.925	255.857	207.204	195.079	279.07	234.848	263.668	209.627	225.296	175.941	155.839	354.638
176.179	213.191	183.578	166.097	211.152	250.366	207.204	208.26	298.605	200.758	244.983	204.969	224.218	181.193	141.938	325.688
193.548	192.692	166.58	166.097	208.41	265.739	209.187	205.624	293.023	227.273	247.059	214.286	225.296	160.185	129.5	322.069
181.141	180.393	159.781	176.35	194.698	262.445	220.093	197.715	281.86	250	251.211	237.578	242.544	186.445	143.036	358.257
171.216	229.591	197.176	166.097	222.121	261.347	203.239	197.715	315.349	238.636	263.668	214.286	258.714	194.323	154.01	358.257
171.216	225.491	180.178	166.097	216.636	250.366	202.247	176.626	301.395	234.848	259.516	214.286	252.246	191.697	140.109	396.254

173.697	184.492	180.178	157.895	222.121	251.464	198.282	187.17	290.233	253.788	263.668	200.311	260.87	168.063	155.108	396.254
191.067	200.892	186.977	168.148	208.41	237.189	191.342	195.079	284.651	227.273	226.298	232.919	263.026	183.819	138.28	343.782
193.548	184.492	163.18	178.401	216.636	233.895	203.239	179.262	295.814	227.273	271.972	214.286	263.026	181.193	125.476	343.782
198.511	221.391	197.176	166.097	213.894	221.816	210.948	187.17	293.023	219.697	244.983	237.578	241.466	175.941	150.718	376.35
166.253	176.293	166.58	172.249	208.41	234.993	196.299	195.079	265.116	234.848	205.536	246.894	255.439	170.689	136.817	343.782
198.511	221.391	176.778	170.198	197.441	234.993	205.088	197.715	304.186	227.273	207.612	237.578	251.072	175.941	135.353	347.4
178.66	196.792	176.778	157.895	191.956	240.483	198.252	184.534	284.651	223.485	236.678	209.627	248.889	173.315	141.207	338.353
188.586	196.792	183.578	149.692	216.636	232.796	199.229	187.17	304.186	231.061	240.83	218.944	247.797	165.437	122.916	365.494
188.586	209.091	193.776	157.895	208.41	239.385	195.322	163.445	276.279	223.485	230.45	237.578	237.973	181.193	150.718	331.116
186.104	217.291	186.977	143.541	208.41	231.698	195.322	150.264	284.651	219.697	242.907	232.919	231.423	168.063	148.157	320.26
181.141	184.492	173.379	168.148	197.441	240.483	204.112	160.808	287.442	193.182	209.689	223.602	228.148	170.689	138.646	320.26
168.734	168.093	203.975	169.557	200.183	189.177	179.696	168.717	287.442	204.545	232.526	237.578	229.24	157.559	141.939	313.022
176.179	188.592	193.776	167.464	208.41	217.445	180.673	189.807	284.651	219.697	199.308	228.261	218.324	152.307	121.453	320.26
171.216	163.993	197.176	177.931	208.41	247.072	191.416	171.353	287.442	196.97	213.841	242.236	222.69	194.323	143.036	331.116
176.179	168.093	207.375	173.744	222.121	250.366	194.346	155.536	284.651	246.212	207.612	200.311	231.423	173.315	142.67	331.116
173.697	192.692	180.178	154.904	208.41	224.012	196.299	155.536	265.116	242.424	184.775	214.286	222.69	157.559	147.425	336.544
151.365	168.093	183.578	163.278	200.183	242.451	185.556	150.264	295.814	212.121	188.927	260.87	221.599	181.193	145.962	378.16
168.734	172.193	203.975	156.998	183.729	235.928	184.579	160.808	262.326	223.485	193.08	214.286	227.057	175.941	132.427	329.307
168.734	168.093	183.578	146.531	200.183	245.713	196.299	142.355	279.07	223.485	203.46	218.944	229.24	168.063	136.085	352.828
166.253	163.993	166.58	156.998	194.698	233.753	188.486	179.262	262.326	223.485	184.775	200.311	216.14	154.933	139.378	345.591
176.179	176.293	166.58	150.718	194.698	217.445	188.486	192.443	278.83	204.545	180.623	237.578	236.881	160.185	126.208	389.016
166.253	127.095	180.178	155.844	213.894	223.968	181.65	181.898	270.548	200.758	159.862	204.969	218.324	154.933	139.743	361.875
166.253	192.692	183.578	151.743	194.698	214.183	180.673	208.26	281.59	223.485	188.927	251.553	213.957	157.559	129.135	338.353
166.253	184.492	183.578	151.743	180.987	204.398	177.743	187.17	281.59	204.545	174.394	218.944	219.415	168.063	146.694	329.307
173.697	213.191	186.977	147.642	197.441	200.049	173.837	205.624	267.787	185.606	191.003	228.261	208.499	168.063	134.256	271.407
163.772	159.893	193.776	141.49	200.183	204.398	177.743	187.17	298.155	219.697	180.623	256.211	220.507	173.315	144.865	347.4
168.734	192.692	197.176	145.591	180.987	198.962	185.556	187.17	292.633	219.697	184.775	214.286	213.957	160.185	154.01	305.785
176.179	209.091	149.582	141.49	191.956	202.224	174.813	184.534	292.633	231.061	166.09	237.578	209.591	152.307	138.646	314.832

173.697	180.393	166.58	139.44	197.441	202.224	171.883	202.988	278.83	223.485	180.623	204.969	204.133	170.689	131.695	314.832
171.216	168.093	166.58	129.187	172.761	193.526	172.86	202.988	265.026	185.606	180.623	228.261	207.408	149.681	135.719	287.691
176.179	159.893	146.182	143.541	200.183	207.66	185.556	208.26	281.59	204.545	161.938	200.311	210.682	144.429	116.697	255.122
173.697	159.893	169.979	129.187	200.183	203.311	170.907	202.988	270.548	231.061	188.927	209.627	223.782	183.819	159.498	296.738
176.179	176.293	183.578	129.187	170.018	204.398	170.907	195.079	270.698	215.909	164.014	214.286	203.041	154.933	148.889	296.738
176.179	188.592	169.979	127.136	200.183	203.311	169.93	202.988	253.953	223.485	199.308	232.919	209.591	144.429	133.524	302.166
193.548	192.692	149.582	135.338	208.41	203.311	173.837	210.896	270.698	212.121	188.927	223.602	209.591	154.933	152.547	330.477
168.734	225.491	169.979	133.288	183.729	187.003	169.93	210.896	293.023	223.485	170.242	223.602	208.499	139.177	139.743	361.689
196.03	184.492	166.58	127.136	194.698	205.485	174.813	200.351	270.698	208.333	182.699	242.236	207.408	141.803	140.109	361.689
208.437	155.794	183.578	133.288	213.894	191.351	167.977	195.079	267.907	212.121	164.014	204.969	197.583	168.063	141.938	337.821
173.697	184.492	149.582	135.338	189.214	170.694	157.234	210.896	273.488	185.606	182.699	190.994	203.041	152.307	150.718	337.821
188.586	180.393	166.58	139.44	167.276	167.433	165.047	208.26	293.023	208.333	170.242	218.944	195.4	141.803	175.594	337.821
178.66	184.492	169.979	127.136	197.441	177.218	157.234	202.988	267.907	196.97	157.785	163.043	204.133	144.429	162.79	308.446
200.993	172.193	166.58	139.44	178.245	181.566	163.094	197.715	262.326	227.273	184.775	237.578	203.041	144.429	155.108	347.001
193.548	184.492	159.781	145.591	164.534	187.003	151.375	192.443	287.442	208.333	149.481	223.602	197.583	173.315	140.841	337.821
198.511	188.592	163.18	137.389	175.503	202.224	173.837	192.443	306.977	238.636	168.166	181.677	203.041	165.437	142.67	341.493
208.437	184.492	149.582	139.44	170.018	178.305	154.304	192.443	276.279	196.97	199.308	195.652	193.216	173.315	138.28	334.149
208.437	168.093	159.781	147.642	183.729	177.218	163.094	210.896	279.07	189.394	195.156	190.994	198.675	147.055	157.668	328.641
203.474	196.792	156.381	129.187	219.378	185.915	176.767	197.715	270.698	223.485	191.003	190.994	209.591	144.429	141.207	334.149
193.548	200.892	163.18	137.389	180.987	187.003	165.047	197.715	281.86	200.758	186.851	190.994	206.316	162.811	154.376	374.541
196.03	168.093	142.783	142.344	189.214	187.003	177.743	200.351	284.651	219.697	176.471	214.286	214.517	131.299	120.721	358.017
205.955	163.993	166.58	138.158	191.956	171.781	146.492	213.533	284.651	193.182	188.927	246.894	231.764	152.307	136.817	324.969
186.104	204.992	166.58	148.624	194.698	191.351	176.767	202.988	262.326	223.485	186.851	204.969	238.232	139.177	124.013	334.149
181.141	184.492	180.178	150.718	200.183	238.287	168.954	226.714	265.116	219.697	186.851	256.211	224.218	141.803	132.793	359.853
186.104	180.393	203.975	159.091	200.183	239.385	164.071	221.441	270.698	227.273	164.014	218.944	227.452	149.681	130.964	313.953
191.067	204.992	180.178	154.904	186.472	249.268	190.439	221.441	259.535	253.788	188.927	204.969	238.232	147.055	147.426	350.673
176.179	184.492	186.977	174.299	186.472	251.464	174.813	216.169	298.605	231.061	159.862	177.019	233.92	160.185	133.524	345.165
183.623	172.193	183.578	147.642	186.472	268.544	166.024	218.805	306.977	196.97	170.242	265.528	244.7	162.811	143.767	330.477

181.141	217.291	173.379	147.642	186.472	239.189	180.673	208.26	293.023	204.545	164.014	288.82	240.388	160.185	155.474	323.133
171.216	176.293	190.377	143.541	161.792	259.847	160.164	208.26	267.907	215.909	151.557	279.503	214.517	152.307	144.499	356.181
181.141	176.293	166.58	147.642	189.214	246.8	178.72	205.624	259.535	223.485	180.623	270.186	223.14	149.681	146.328	341.493
188.586	176.293	163.18	141.49	186.472	240.277	172.86	202.988	309.767	227.273	153.633	228.261	211.283	152.307	152.913	339.657
203.474	196.792	183.578	141.49	159.049	274.524	160.164	208.26	306.977	208.333	182.699	270.186	219.907	149.681	155.108	396.573
203.474	155.794	173.379	129.187	180.987	264.641	177.743	202.988	304.186	181.818	157.785	265.528	214.517	133.925	133.525	348.837
196.03	180.393	186.977	133.288	197.441	252.562	166.024	192.443	315.349	204.545	139.1	246.894	204.815	141.803	120.721	348.837
210.918	200.892	169.979	133.288	202.925	245.974	173.837	189.807	306.977	212.121	178.547	204.969	205.893	154.933	142.304	354.345
196.03	155.794	152.981	140.251	202.925	247.072	153.328	187.17	326.512	185.606	145.329	232.919	199.425	160.185	143.036	352.509
210.918	196.792	156.381	142.344	205.667	228.404	167.977	189.807	301.395	234.848	178.547	214.286	198.347	141.803	135.719	334.149
205.955	196.792	180.178	123.505	194.698	222.914	165.047	166.081	334.884	196.97	151.557	246.894	196.191	162.811	140.475	341.493
183.623	176.293	156.381	129.785	194.698	220.717	156.258	189.807	309.767	227.273	149.481	237.578	198.347	165.437	157.303	321.297
191.067	168.093	166.58	131.878	197.441	218.521	171.883	173.989	301.395	223.485	149.481	232.919	191.879	165.437	142.67	330.477
181.141	172.193	166.58	136.065	205.667	196.559	159.188	176.626	298.605	246.212	151.557	223.602	196.191	139.177	126.94	295.594
163.772	192.692	186.977	117.225	208.41	204.246	153.328	189.527	293.023	223.485	168.166	223.602	188.645	162.811	120.721	321.297
173.697	204.992	169.979	129.785	202.925	202.05	151.375	173.95	290.233	231.061	164.014	223.602	198.347	147.055	139.011	284.578
173.697	180.393	149.582	136.065	186.472	197.657	135.749	189.527	287.442	231.061	153.633	223.602	184.333	141.803	144.865	249.694
191.067	180.393	176.778	136.065	197.441	189.971	138.679	189.527	295.814	223.485	155.709	200.311	189.723	136.551	127.306	286.414
161.29	188.592	149.582	136.065	180.987	185.578	145.515	197.316	273.488	204.545	143.253	237.578	182.178	152.307	141.939	273.562
183.623	163.993	149.582	129.785	208.41	184.48	168.539	184.335	287.442	212.121	166.09	195.652	204.815	162.811	147.425	266.218
186.104	168.093	166.58	125.598	175.503	186.676	176.471	173.95	290.233	212.121	161.938	260.87	180.022	160.185	164.253	282.742
203.474	188.592	149.582	131.878	175.503	184.48	190.35	192.123	298.605	227.273	141.176	190.994	191.879	157.559	154.376	244.186
183.623	213.191	203.975	117.225	186.472	189.971	192.333	194.72	273.488	227.273	168.166	190.994	189.723	149.681	132.061	235.006
188.586	139.394	156.381	136.065	208.41	209.736	200.264	205.105	290.233	231.061	159.862	204.969	199.425	141.803	160.595	251.53
181.141	159.893	166.58	129.785	189.214	200.952	201.256	197.316	290.233	234.848	143.253	200.311	199.425	139.177	136.085	321.297
163.772	172.193	176.778	119.318	191.956	194.363	191.342	186.931	281.86	234.848	157.785	214.286	197.269	165.437	130.232	317.625
168.734	188.592	210.774	140.251	189.214	195.461	178.453	202.508	306.977	223.485	151.557	200.311	211.283	152.307	129.866	308.446
163.772	163.993	173.379	108.852	194.698	176.794	172.505	205.105	279.07	212.121	145.329	256.211	189.723	152.307	156.571	308.446

163.772	180.393	207.375	127.691	167.276	173.499	194.316	202.508	279.07	253.788	130.796	237.578	196.191	170.689	125.476	251.53
163.772	204.992	210.774	146.531	202.925	184.48	182.419	205.105	276.279	212.121	159.862	242.236	191.879	160.185	127.306	251.53
171.216	209.091	217.573	131.878	172.761	196.559	181.428	197.316	256.744	231.061	134.948	218.944	182.178	144.429	137.914	286.414
161.29	217.291	210.774	150.718	186.472	176.794	192.333	181.738	259.535	208.333	155.709	186.335	186.489	154.933	142.304	238.678
161.29	209.091	183.578	154.904	194.698	183.382	185.393	189.527	284.651	227.273	143.253	223.602	195.113	160.185	159.498	291.922
166.253	217.291	220.973	152.811	159.049	189.971	185.393	184.335	256.744	181.818	164.014	228.261	194.035	110.291	156.205	233.17
166.253	192.692	197.176	129.785	167.276	197.657	184.402	194.72	284.651	242.424	130.796	218.944	197.269	131.299	124.379	258.874

Figure 4C

SHAM	MI	MI+ARM36	MI+S107	MI+Propr	MI+SD-208
180.1758	135.2569	126.570916	192.914	166.2531	161.6405
130.0198	114.3922	121.9503	194.6056	192.6921	170.9568
179.2829	106.2237	175.0919	142.9706	197.176	144.3089
255.3606	154.8773	66.67183	140.8451	129.7847	194.0585
117.9775	125.9446	201.7911	233.5634	167.276	151.6849
142.7313		129.2916	204.6679	197.6574	236.4407
225.4547	160.6119	110.3774	278.1073	184.4018	183.263
197.903	148.5149	101.6598	220.2656	194.7196	152.2842672
168.2243	174.003113	162.6506	225.1816	284.6512	191.0509
177.6217	93.28358	152.3948	200.3169	242.4242	133.5927
210.0457	129.599308	138.0753	136.6021	130.7958	182.0634
314.6132	210.290726	27.86378		218.9441	105.1136
342.9032				197.2691	191.9845
				131.2991	160.7206
				124.3789	157.8947
				258.8739	266.7913

Figure 4D

	SHAM												
5	4.161488	0.416149	0.241437	0.685453	0.573639	0.352126	0.518799	0.992442	0.235153	0.162328	0.165856	0.152588	0.158286
7	5.931239	0.454964	0.313868	1.788139	1.089913	0.349438	0.915527	1.519676	0.653202	0.243491	0.230197	2.390544	0.300744
9	6.325014	0.769939	1.231326	2.205372	1.77828	0.757115	1.800537	2.326035	0.783842	0.730474	0.328853	4.679362	0.395715
10	6.16792	1.189906	1.593481	2.920628	2.093781	1.019194	2.149552	2.419076	1.071251	1.163347	0.295968	4.83195	0.506516
11	5.994524	1.609872	2.076354	2.831221	1.893008	1.71807	2.512856	2.760228	1.724452	1.089913	0.526165	5.137125	0.664802
12	5.20186	1.88985	2.052211	2.801418	2.610056	1.601591	2.633958	3.008338	1.81527	1.226153	0.98656	4.984538	0.80726
13	5.562075	2.694786	2.172929	3.09381	2.495328	1.776309	2.755059	2.946311	2.209894	1.198905	1.160995	5.289713	0.838917
14	5.586686	2.5198	2.414365	3.153883	2.982921	1.980148	2.966987	3.039352	2.367743	1.144409	1.061481	4.730225	1.139661
15	5.315965	2.834775	2.51094	3.183921	2.982921	2.154867	2.81561	2.512118	2.456038	1.00817	1.525879	5.0354	1.218804
25	5.291354	2.169828	2.599826	3.033736	2.66742	2.154867	2.573407	2.729214	2.142501	1.117161	1.227337	4.425049	0.680631
30	5.119078	3.359733	2.551231	2.883551	2.839511	2.154867	2.960205	2.946311	2.456038	1.226153	1.492708	4.918952	1.092175
35	4.799135	3.534719	3.017957	3.153883	3.011603	2.446065	3.204346	2.760228	2.893908	1.198905	1.791249	4.918952	1.060518
40	4.725302	4.234664	3.017957	2.463033	3.269741	2.649904	3.570557	3.256449	2.893908	1.307896	1.990277	5.069532	1.361261

MI

0.602321	1.298057	1.519127	1.093724	0.930968	1.839712	0.222019	0.133381	0.415704	1.284541	0.538545	0.999969
1.520143	1.867334	1.957986	1.253782	2.202533	3.435933	0.284073	0.052982	1.027034	2.101976	1.256606	1.370328
1.835644	1.894011	2.936979	2.187449	2.792903	5.735573	0.486983	0.266762	1.173753	2.374454	2.833138	1.777723
2.093781	2.240801	3.34208	2.614268	2.906436	7.304739	0.669601	0.506848	1.736176	2.997262	2.543132	2.036974
2.3806	2.187449	4.152281	2.854354	3.201621	7.331794	0.706425	0.400143	1.736176	2.724784	2.520823	2.444369
2.466646	2.054068	3.814697	2.854354	3.33786	7.981104	0.50459	0.453495	1.662817	2.763709	2.81083	1.999938
2.66742	2.000715	4.456107	3.147792	3.088088	7.764668	0.766976	0.506848	1.785083	2.530156	2.877754	2.407333
2.638738	2.10742	3.848456	3.361202	3.83727	8.873906	0.867894	0.693581	1.931802	2.880486	2.699289	2.740656
2.753466	2.054068	4.253556	3.65464	3.566403	8.90096	1.271566	0.613553	1.980708	2.608007	2.917121	3.111015
2.724784	2.054068	4.152281	3.387878	3.543831	8.60336	0.968812	0.506848	1.516098	2.724784	2.98444	2.444369
2.868193	2.187449	4.321073	3.86805	3.769553	8.549251	1.231199	0.826962	2.200787	3.152964	2.715167	2.8888
3.126331	1.787306	4.186039	3.921402	3.724409	8.846851	1.594503	1.147077	2.608761	2.919411	2.98444	3.073979
3.183695	1.787306	4.186039	4.695012	3.566403	9.754725	1.251382	1.253782	2.608761	2.724784	2.647849	2.740656

MI+ARM36

0.211928	0.397741	0.337584	0.4014	1.050423	2.831129	0.067717	0.6186	0.031014	0.17856	0.226296	0.122561
0.701904	0.63277	0.337584	0.643913	1.802921	5.565832	0.20315	1.134099	0.093041	0.373353	0.293438	0.199161
1.647949	0.83164	2.194295	0.747051	3.750197	7.899393	0.970603	2.061999	0.217097	0.308422	0.912919	0.228882
1.861572	0.958194	2.768187	0.830676	3.691839	8.713248	1.625196	2.577498	0.496221	0.565739	0.905182	0.291081
2.755059	0.958194	3.13953	1.106638	4.217052	9.912357	1.896062	2.680598	1.333593	0.533411	1.131478	0.320435
3.509521	1.04859	3.510872	1.067613	3.989769	9.80309	2.189501	2.938348	1.860828	0.727379	1.196134	0.411987
4.02832	1.138985	3.814697	1.218139	4.548765	9.874242	2.166929	2.680598	1.829814	0.872854	1.108544	0.411987
3.936768	1.355935	3.915972	1.226501	4.665479	10.16739	2.166929	2.989898	1.612718	0.614231	1.499796	0.473022
3.997803	1.518647	4.017248	1.193051	4.665479	9.650528	2.347506	3.092998	1.829814	0.759707	1.010732	0.50354
3.967285	1.482489	3.915972	1.140088	4.855907	9.777814	2.347506	3.041448	1.7988	0.743543	1.271566	0.534058
3.814697	1.717518	4.051006	1.123363	5.540832	9.226244	2.347506	3.453847	2.139952	0.925267	1.499796	0.62561
4.425049	1.717518	4.287314	1.243226	5.718975	9.746957	2.482939	3.556947	2.481104	0.892801	1.422429	0.488281
4.211426	1.699439	4.051006	1.223714	5.411833	9.974529	2.460367	3.814697	2.574145	1.12006	1.487085	0.656128

MI+S107

0.099514	0	0.277892	0.641409	0.223651	0.646559	0.3093	0.222316	0.289724	0.396729	0.177428
0.361739	0.26537	0.530521	0.877718	0.566153	1.422429	0.25775	0.391704	0.362155	1.068115	0.440157
1.085216	0.364884	1.212619	0.978993	2.057613	1.810365	0.46395	0.818699	0.72431	1.312256	0.857568
1.348298	1.249642	1.187356	1.485369	2.371876	2.133644	0.56705	0.790462	1.038177	1.556396	1.330708
1.54561	2.720045	1.338933	1.519127	2.868193	2.068988	1.005224	0.666955	1.231326	1.617432	1.862992
2.071775	3.615669	1.465248	1.789194	2.768426	2.165972	1.340299	1.231571	1.424476	1.831055	2.217847
2.696596	3.847583	1.263145	1.417852	3.26226	2.35994	1.391849	1.090419	1.472763	1.983643	2.631845
3.156991	3.979124	1.439985	1.789194	3.479245	2.165972	1.366074	1.280975	1.496907	2.210102	2.963726
3.814697	4.14355	1.490511	1.890469	3.384468	2.715547	1.623824	1.602108	1.617625	2.210102	2.934382
3.09122	4.345438	1.313671	1.654161	3.838388	2.295284	1.675374	1.344494	1.472763	2.300929	2.37685
4.57106	4.932798	1.389459	1.620402	3.895758	2.230628	1.984674	1.70797	1.569337	2.331204	2.787663
5.228766	4.867028	1.74314	2.025503	3.623899	2.1983	2.371298	1.644451	1.762487	2.391755	3.110445
6.070344	4.998569	1.490511	1.789194	3.706205	2.165972	1.933124	1.880881	1.847369	2.563477	3.169133

MI+Prop

0.370083	0.769632	0.917459	0.873244	1.144409	0.73115	0.551522	0.55689	0.562824	0.717294	0.035651	0.132455	0.193968	0.627074	0.131541	0.487593	0.303442
1.537266	1.204641	1.352045	1.149005	2.034505	1.398722	0.735363	0.30629	0.687896	1.630213	0.106954	0.052982	0.452591	1.4893	0.526165	0.860458	0.471479
2.476707	2.208509	2.028067	1.746488	2.161662	1.716614	1.011125	0.501201	1.438329	1.728025	0.211928	0.450346	0.905182	2.011861	1.414069	1.204641	0.72865
2.448239	2.777367	2.241135	1.884369	2.511342	1.811981	1.286886	0.473357	1.375793	1.923651	0.570422	0.55631	1.09915	2.325398	1.808693	1.405415	1.585885
2.448239	3.011603	2.670288	1.930329	2.861023	1.970927	1.641476	0.723957	1.563401	2.086672	0.819982	0.688765	1.422429	2.377654	2.119276	1.405415	1.671609
3.330743	3.145452	2.897238	2.11417	2.988179	2.415975	1.548998	0.779646	1.594669	1.891047	0.998239	0.768238	1.616397	2.482166	2.433514	1.548824	1.603907
3.159936	3.212377	2.559227	2.25205	3.147125	2.585167	1.595237	0.891024	2.157493	1.923651	1.069541	0.768238	1.745709	2.273142	2.565055	1.77828	1.777302
3.330743	3.74718	2.897238	2.16013	3.306071	2.742799	1.733953	1.280847	2.126225	2.327612	1.212147	0.874201	1.875021	2.769575	2.59794	1.92169	2.557581
3.188404	3.679664	2.655802	2.29801	3.178914	2.868905	1.803311	1.364381	1.751009	1.972005	1.426055	0.900692	2.068988	2.42991	2.630826	1.950372	2.861023
3.50155	3.645905	2.945526	2.29801	3.083547	2.616693	1.641476	1.085936	1.969885	2.004333	1.28345	0.953674	1.713381	2.42991	2.706153	1.835644	2.05736
3.586954	4.152281	2.607515	2.481851	3.210704	2.900431	1.780192	1.503603	1.907349	2.068988	1.319101	0.900692	1.810365	2.377654	2.771361	1.92169	4.421581
3.786229	4.287314	2.800664	2.25205	3.019969	3.026537	1.965147	1.962634	2.501441	1.616397	1.426055	1.006656	2.03666	2.482166	2.696596	2.122463	4.421581
3.757761	4.316631	2.51094	2.25205	3.751119	3.528595	1.942028	2.045562	2.595245	1.681053	1.568661	1.298057	2.392268	2.795703	2.795252	2.437964	4.843379

MI+SD-208

0	0.367682	0.296952	0.226616	0.747209	1.535183	0.131541	0.455486	0.298542	0.431227	0.23657	0.370359	1.063112	0.234031	0.215611	0.115597
0.447534	1.149005	0.18274	0.793155	1.061823	3.582094	0.164427	1.224119	0.630254	0.431227	0.410814	0.62961	1.860059	0.748898	0.240493	0.023119
0.895069	1.884369	0.499544	1.926233	1.140476	4.791632	0.624821	1.508798	0.630254	0.928796	1.056378	1.296256	2.080744	1.651485	0.489278	0.231194
1.619648	1.838408	0.59037	2.190618	1.390775	4.931194	0.690592	1.651138	0.630254	1.227337	1.212423	1.555508	2.11227	1.860828	0.572205	0.393029
1.83276	1.976289	0.499544	2.190618	1.708666	5.629004	1.249642	1.651138	1.061481	1.459536	1.626421	2.148082	1.986165	2.246693	0.626106	0.459602
2.067182	2.068209	0.59037	2.670288	1.788139	5.768567	1.085216	1.765009	1.360023	1.426365	2.010848	1.925867	1.986165	2.504126	0.675864	0.485507
2.045871	2.481851	0.567663	2.593994	1.708666	5.768567	1.381184	1.979053	1.459536	1.525879	2.217847	2.148082	2.427535	2.410514	0.704889	0.554865
2.152427	2.25205	0.658489	2.708435	1.827876	6.326815	1.578495	2.237191	1.724907	1.393194	1.922134	2.333261	2.238376	2.667948	0.729768	1.063491
2.067182	2.527812	0.772022	2.479553	1.629194	6.373336	1.677151	2.179827	1.857592	1.592221	2.523569	2.111046	2.679746	2.597739	0.953674	0.716701
2.109805	2.11417	0.635783	2.757157	1.748403	6.69898	1.644266	2.151145	1.426365	1.55905	2.112755	2.148082	2.332955	2.246693	0.95782	0.73982
2.131116	2.435891	0.794729	3.097081	2.106031	6.792022	1.512725	2.351919	1.82442	1.55905	2.347506	2.185118	2.963484	2.76156	1.003432	0.670462
2.749139	2.573772	0.953674	3.13485	1.947085	6.65246	1.907349	2.3806	2.32199	1.990277	2.464881	2.25919	3.310275	3.323233	1.057334	0.901656
2.685206	2.481851	0.999087	3.66362	2.145767	6.00117	1.940234	2.179827	2.189305	1.890763	2.582257	2.666584	2.900431	3.440248	1.252215	1.040372

Figure 4F

SHAM	MI	MI+ARM036	MI+S107	MI+propranolol	MI+SD-208
103.614458	69.2237443	113.333333	82.5396825	202.409639	160.655738
96.3855422	67.5799087	60	98.4126984	185.542169	236.065574
100	62.6484018	80	85.7142857	200	95.0819672
82.5396825	86.0273973	106.666667	117.460317	204.819277	108.196721
117.460317	67.9452055	93.3333333	165.079365	66.6666667	111.47541
86.6666667	69.4063927	106.666667	136.507937	105.555556	121.311475
113.333333		51.1111111		53.3333333	
103.614458		56.6666667			
96.3855422		60			
100					
82.5396825					
117.460317					
86.6666667					
113.333333					
100					
95.0819672					
104.918033					

Figure 4G

Time			Sham				MI	
10	6.61E-14	0.455867	1.220877	9.92E-12	1.35391	1.306354	5.38E-09	4.19E-18
20	3.68E-08	1.450862	1.59301	1.105578	1.701334	1.946503	1.140967	0.184089
30	0.00042	2.087529	2.064374	2.187397	2.071767	2.127518	1.857147	1.202719
40	0.837937	2.879032	3.281264	2.876618	4.143933	2.385182	2.708812	2.321416
50	1.143428	3.946627	3.596165	3.053969	4.594324	2.119806	2.675503	2.692763
60	1.819863	4.28369	4.36611	3.500599	4.706116	2.896073	2.806483	2.911117
70	2.308245	4.607466	4.517828	3.731033	5.097819	2.823043	3.255247	3.582495
80	3.385018	4.968643	4.847978	4.104429	5.510344	3.186872	3.560457	4.008473
90	3.691809	5.06644	5.458374	4.389735	5.879888	3.725384	3.610866	4.466627
100	4.156576	5.653012	5.53029	3.918527	5.869845	3.692843	3.884115	4.260383
110	4.293852	5.553723	5.986867	4.666897	6.149668	3.536073	3.944461	4.634053
120	4.658205	5.799339	5.592099	4.782429	6.439783	4.069323	3.935042	5.183448

Figure 4H

SHAM	MI
7.265	7.338
7.149	7.222
7.193	7.088
7.368	7.174
7.362	7.251
7.346	7.237
	7.104

Figure 4I

SHAM	MI
103	62
66	31
83	52
74	76
86	62
80	113
	121

Figure 4J

SHAM	MI
48.4	41.4
56.5	55.7
53.2	66.2
28.8	48.8
31.8	54.5
45.1	58.8
	83.9
