# Supplementary material for: Heart failure-induced cognitive dysfunction is mediated by intracellular Ca2+ leak through ryanodine receptor type 2
Source: Nat Neurosci. 2023 Jul 10;26(8):1365–78. doi: 10.1038/s41593-023-01377-6 (PMC10400432; doi:10.1038/s41593-023-01377-6)
Supplement: Source Data Fig. 5 — Statistical source data. [file 41593_2023_1377_MOESM8_ESM.pdf]

Figure 5A

Time	WT+Iso+prop	WT+ISO	WT+Iso+s107	WT+S107	S2808D	WT	S2808D+S107
0	1		1	1	1		1
0.933	1.02081467	1	0.99722135	0.9843107	1.0226199	1	1.02341045
1.866	1.03106827	0.99309452	0.97606568	1.02001886	1.01092619	0.98531333	0.97556657
2.799	1.0612676	1.0058707	1.00858857	1.01230281	1.01551679	0.95855349	1.03352219
3.732	1.05628117	1.00075919	0.99854752	1.00214335	1.03139561	0.94150901	1.0184136
4.665	1.0360547	0.99668246	0.99595832	1.01251715	1.01442344	0.92375433	0.95298237
5.598	1.05188295	0.99012537	0.97196085	1.01508916	1.02134554	0.91003352	0.99252439
6.531	1.07210941	1.01008263	0.97347648	1.01371742	1.0000362	0.89747742	0.98630784
7.464	1.0360547	1.00048359	0.97455005	1.01984739	1.0101623	0.88892677	1.00204596
8.397	1.02168362	0.97644961	0.98181244	1.02503429	0.99406264	0.87977956	0.98197986
9.33	1.03165648	1.00117518	0.9845911	1.01384602	0.98747362	0.88006363	0.98343563
10.263	1.03429007	0.99247053	1.0102305	1.03532236	0.97148257	0.8799216	0.97100252
11.196	1.0360547	1.00337994	0.97587622	1.01924726	0.97898391	0.86770638	0.95565785
12.129	1.02755237	0.99627166	0.98383328	1.03789438	0.96095823	0.86818931	0.99378344
13.062	1.00145716	1.01008263	1.00170508	1.03703704	0.99304532	0.85242316	0.99378344
13.995	1.01260645	0.99599607	0.99943164	1.02812071	0.95290659	0.87645588	0.96313346
14.928	1.02081467	0.99274612	1.00985159	1.03450789	0.95877155	0.86159877	0.96769751
15.861	0.99676484	0.98549745	0.99349542	1.06074246	0.95184945	0.85875803	0.98260938
16.794	1.0067377	0.98743182	0.96785602	1.06665809	0.94398245	0.84668485	0.97119924
17.727	0.99236662	0.97396405	0.9772024	1.09087791	0.9239873	0.8708312	0.9548316
18.66	1.02433057	0.99551248	0.98364383	1.05384088	0.92067469	0.86350207	0.95050362
19.593	1.00029411	0.98473826	0.97827597	1.07878944	0.89827563	0.8643543	0.98713409
20.526	1.01143002	0.9770008	0.97417114	1.09246399	0.91022276	0.87722289	0.98528486
21.459	1.01260645	0.97569042	0.98793811	1.10223765	0.89230207	0.88892677	0.98095688
22.392	1.00644359	0.98611624	1.00650458	1.09134945	0.88115503	0.90534629	1.03064998
23.325	1.04015882	0.98017794	1.01711399	1.11166838	0.87754917	0.88355775	1.01180359
24.258	1.01524003	0.98770741	1.00909378	1.08620542	0.88833055	0.89426737	1.0016525

25.191	1.01612235	0.97251327	0.99204294	1.07926097	0.89762035	0.87983637	1.00539031
26.124	1.02696416	0.97617401	0.9875592	1.0932356	0.89601654	0.88270553	1.0217186
27.057	1.0035159	0.97299686	0.9926113	1.09863683	0.88599905	0.8894381	0.98799969
27.99	0.99383715	0.97907556	0.97549732	1.08693416	0.88909444	0.87117209	1.01554139
28.923	0.98798177	0.97707359	0.95579413	1.09053498	0.90297484	0.88219419	1.01408562
29.856	0.98973303	0.97251327	1.02172403	1.09344993	0.90730838	0.88673939	0.98363236
30.789	1.00086895	0.97769238	0.96659299	1.10682442	0.89233827	0.86679734	0.96104816
31.722	0.99354304	0.98708342	0.96785602	1.09653635	0.87787862	0.87034828	0.95400535
32.655	0.97976017	0.96975212	0.99349542	1.09242112	0.88406941	0.86949605	0.95215612
33.588	0.96921246	0.98522185	0.97568677	1.11329733	0.90443021	0.86983694	0.95546113
34.521	0.95748834	0.96284664	0.90716767	1.1038666	0.90683412	0.87452417	0.98237331
35.454	0.94458778	0.96105267	0.96899274	1.1196845	0.90715995	0.8685586	0.95817595
36.387	0.9522078	0.9707869	0.94190085	1.10575274	0.88611128	0.88048974	0.96439251
37.32	0.95250191	0.96726656	0.97530786	1.1253858	0.87988067	0.88682461	0.95298237
38.253	0.9486919	0.96181186	0.99071677	1.09940844	0.87602139	0.90301687	0.98154706
39.186	0.96540246	0.97161889	0.96772971	1.11364026	0.86476575	0.92244759	0.98197986
40.119	0.96218066	0.97051131	0.96324597	1.09692215	0.87423656	0.91577183	0.99087189
41.052	0.93667366	0.96892533	0.97530786	1.12495713	0.86549343	0.92267485	0.97056972
41.985	0.94107188	0.97354805	1.00612567	1.16863855	0.86006654	0.9122209	0.98713409
42.918	0.94107188	0.98956898	0.98364383	1.14437586	0.85045091	0.91665246	0.96832704
43.851	0.93842493	0.96989252	0.96210925	1.14386145	0.84364105	0.92148173	0.98674064
44.784	0.93989546	0.99185174	0.97701295	1.20374657	0.85999414	0.91514687	0.96915329
45.717	0.92846544	0.98777501	0.97922324	1.18865741	0.85529493	0.90384069	0.99106862
46.65	0.93726187	0.98756701	0.91089359	1.18102709	0.85722819	0.90412477	0.98072081
47.583	0.95132548	0.9770008	0.96103568	1.14656207	0.85933885	0.91315834	0.95132987
48.516	0.92259669	0.99371331	0.97789706	1.15629287	0.8503061	0.90378388	0.98154706
49.449	0.94048367	0.98943378	0.95579413	1.13743141	0.83832277	0.91918073	1.00767233
50.382	0.96100424	0.98508146	0.98402273	1.16743827	0.82524971	0.90946537	0.94822159
51.315	0.91908079	0.97486363	0.9926113	1.15046296	0.83770369	0.92111244	0.97702235

52.248	0.94341136	0.97962675	1.02191348	1.17052469	0.83049197	0.91699335	1.01200031
53.181	0.93198134	0.97969435	1.035491	1.15393519	0.82728797	0.9175615	1.00661001
54.114	0.95132548	1.0056627	1.02380802	1.17283951	0.84786599	0.92366911	1.01447907
55.047	0.93286366	0.98936618	1.0988317	1.15174897	0.86148934	0.93474803	1.01778407
55.98	0.92846544	1.00179397	1.06574045	1.09692215	0.87412795	0.93625362	0.97596003
56.913	0.95778244	0.99723885	1.02898642	1.03210734	0.89707368	0.94895176	0.97989455
57.846	0.93520313	0.98798301	1.06927692	1.10901063	0.88122744	0.93284472	0.9774158
58.779	0.94692726	1.00752427	1.01951374	1.0007716	0.88155689	0.94369638	0.91717816
59.712	0.92962849	1.01560493	1.04868961	0.98812586	0.87791483	0.94062837	1.43653604
60.645	0.93549724	0.99592327	1.06296179	1.01663237	0.87678528	0.93261746	3.49055713
61.578	0.91527078	1.01650452	1.00909378	1.07145919	0.90599782	0.94292938	3.22383538
62.511	0.91234309	1.01221979	0.99873698	1.17476852	0.89587172	0.9380717	3.04658483
63.444	0.9419542	1.00117518	1.00353647	1.22389403	0.91856041	0.94304301	4.36358986
64.377	0.93345187	0.99212734	1.01597727	1.18432785	0.90839811	0.96761548	5.18838527
65.31	0.92905365	1.00337994	0.99033786	1.17189643	0.91743086	0.98133629	5.84175323
66.243	0.94077778	0.98943378	0.99330597	1.17091049	0.90501309	0.97096756	6.04324048
67.176	0.92113953	0.97859197	0.94764762	1.17112483	0.88359514	0.96281461	6.29898489
68.109	0.93051081	0.98791541	0.94133249	1.16336591	0.89798238	0.96670644	6.83840888
69.042	0.89533842	0.988607	0.9417114	1.1773834	0.91572206	0.96292824	7.02852534
69.975	0.93637956	0.99019297	0.93425955	1.18055556	0.89568709	0.97153571	7.14656122
70.908	0.92728901	0.9944049	0.94133249	1.17884088	0.90625124	0.96741662	7.14636449
71.841	0.94399957	0.9942645	0.94562678	1.18338477	0.91138489	0.97730243	7.13101983
72.774	0.94136599	0.99378611	0.93943795	1.18454218	0.93888501	0.97201864	7.14510545
73.707	0.93579135	0.97603881	0.96659299	1.20096022	0.91076581	0.97792739	7.31137866
74.64	0.93256955	0.9943373	0.96564572	1.18008402	0.90843793	0.98752912	7.31637551
75.573	0.91087256	0.98874219	0.93167035	1.18642833	0.9005311	0.98636441	7.27057759
76.506	0.9337326	0.98680783	0.94910009	1.19933128	0.91429926	0.99818192	7.2834435
77.439	0.94166009	1.00614629	0.95156299	1.20820473	0.90814468	0.97744446	7.42610954
78.372	0.9299226	0.99889242	0.92017682	1.19097222	0.90107778	0.98218851	7.47517312

79.305	0.91731615	1.00863185	0.97998105	1.20914781	0.88053596	0.97801261	7.4619531
80.238	0.90647434	0.99675526	1.01395643	1.21274863	0.89317457	0.99065394	7.64294145
81.171	0.9011938	1.00414433	0.99797916	1.21553498	0.89011538	0.97892165	7.75059018
82.104	0.91878668	1.00055119	0.98629618	1.20057442	0.89117252	0.98835293	7.75908876
83.037	0.92377311	1.00497111	1.00524155	1.20250343	0.92205766	1.00397705	7.9005351
83.97	0.9161531	1.01284378	1.02860751	1.21656379	0.91058479	1.00292597	7.92001102
84.903	0.8976779	0.99613127	0.99892643	1.18968621	0.9137888	0.99170502	7.9953966
85.836	0.89708969	0.91105553	1.04982633	1.20361797	0.92132997	0.99389239	7.98752754
86.769	0.89856022	0.10972851	1.0453426	1.22007888	0.90486827	1.00249986	7.97820271
87.702	0.88389503	0.12077832	1.04628986	1.25248628	0.92682927	1.00215897	8.02644004
88.635	0.89738379	0.36351015	1.05759394	1.26350309	0.95859053	1.0132947	8.1291706
89.568	0.90384076	0.81824233	1.04610041	1.25625857	1.1433221	1.02641895	8.15336796
90.501	0.90384076	1.0874209	1.02955478	1.06460048	1.48554216	1.03119141	8.10556342
91.434	0.90559202	1.23292479	1.04085886	1.12414266	2.21139539	1.05192887	8.10182562
92.367	0.90676845	1.31088705	1.04868961	1.57848937	2.74191668	1.04681552	7.96868115
93.3	0.91468257	1.39410122	1.0757815	3.88644547	2.97957041	1.0588887	7.98937677
94.233	0.92377311	1.51032442	0.9922324	7.2188786	3.00805164	1.07493892	7.91151243
95.166	0.91380025	1.68302905	0.92611304	8.61831276	2.93935927	1.06323504	8.05441454
96.099	0.919669	1.90573602	0.94562678	9.07561728	2.85985294	0.9893472	7.87736072
97.032	0.87921607	2.09998908	0.88929586	9.13125857	2.73368764	0.53312312	7.83179887
97.965	0.894162	2.33754179	0.89340069	9.13194444	2.63545691	0.54664508	7.78249921
98.898	0.89708969	2.57405973	0.89933691	9.18389918	2.48200871	0.93963411	7.74024237
99.831	0.9047097	2.76969596	0.91196716	9.23971193	2.38196056	1.03766831	7.74067517
100.764	0.89563253	2.94260859	0.89359015	9.22800926	2.22275964	1.05599114	7.74523922
101.697	0.88771841	3.09487757	0.85607831	9.16915295	2.00524588	1.07002443	7.63940038
102.63	0.87334733	3.17277223	5.15352068	9.23452503	1.84284095	1.08161468	7.56008026
103.563	0.88858736	3.24362621	8.66251973	9.16152263	1.69770144	1.15760468	7.60607491
104.496	0.87275911	3.3253168	8.76444585	9.13648834	1.56213774	1.21186296	7.48390777
105.429	0.90354665	3.37503835	8.7873066	9.10703875	1.42278716	1.25035509	7.47332389

106.362	0.86396268	3.47606741	8.43700663	9.06678669	1.33894366	1.34409977	7.35383223
107.295	0.88243787	3.47917696	7.95389959	9.00252915	1.25561062	1.46278621	7.30827038
108.228	0.89239736	3.44830509	7.55086833	9.00801612	1.20319893	1.6364127	7.25236072
109.161	0.89738379	3.46715996	7.1568677	8.92815501	1.15635895	1.83654338	7.20349386
110.094	0.89269147	3.49215593	6.89706347	8.89437586	1.14463266	2.07033691	7.1049339
111.027	0.83874978	3.50210336	6.64704768	8.87397119	1.11148843	2.27762627	7.07865124
111.96	0.81265457	3.49492229	6.55099463	8.85583848	1.07641456	2.51883416	6.96285804
112.893	0.81324278	3.5465054	6.26422482	8.83654835	1.08351767	2.73203227	6.9527463
113.826	0.82439207	3.52689134	6.02911272	8.7394976	1.07659558	3.00650531	6.85064526
114.759	1.90910792	3.50499971	5.79191664	8.76105967	1.09105522	3.28626214	6.87012118
115.692	4.47695948	3.52081784	5.56103568	8.69148663	1.09356774	3.48423385	6.72556657
116.625	5.14832293	3.52634015	5.30154721	8.67005316	1.09604405	3.72848134	6.75247875
117.558	5.51301418	3.5114216	5.03706978	8.58281893	1.09426284	3.95906483	6.72474032
118.491	5.70533267	3.5116296	4.87546574	8.65474966	1.08861511	4.18021703	6.60398961
119.424	5.82465944	3.54795618	4.62450268	8.56665809	1.11236455	4.3343276	6.50956091
120.357	5.86189057	3.52171223	4.42355542	8.60313786	1.09826694	4.54613374	6.50814448
121.29	5.99235325	3.48352408	4.26403536	8.56361454	1.09615628	4.61374354	6.54851275
122.223	6.15417831	3.5058993	4.1115251	8.56648663	1.10853785	4.63817397	6.55429651
123.156	6.29049637	3.46715996	3.9724029	8.48182442	1.09750305	4.64078746	6.38987252
124.089	6.38694972	3.47537582	3.84389012	8.4371142	1.10307476	4.61104483	6.42217501
125.022	6.498349	3.4827649	3.77196085	8.36852709	1.08631981	4.61536276	6.27887945
125.955	6.59450824	3.43228416	3.63694348	8.35198045	1.07579548	4.57894438	6.16768964
126.888	6.70240498	3.42731305	3.56021471	8.30559842	1.06239297	4.51940231	6.15317123
127.821	6.77716134	3.42883142	3.45456268	8.30182613	1.02025219	4.31722629	6.13267233
128.754	6.82699892	3.37476275	3.42374487	8.21167695	1.02385805	4.27702971	6.06456563
129.687	6.7786185	3.34265331	3.37524471	8.22496571	1.00630302	4.24833816	5.99665565
130.62	6.87858776	3.34341249	3.30167351	8.16298011	0.97731132	4.25325266	5.90324992
131.553	6.7783244	3.30764231	3.28481213	8.18227023	0.94052502	4.25294017	5.96848442
132.486	6.84019358	3.27836161	3.21161983	8.10549554	0.94551385	4.18783024	5.96683192

133.419	6.82318891	3.29700329	3.20568361	8.11235425	0.92387869	4.12547583	5.88231822
134.352	6.82113017	3.28229794	3.13659615	8.08912037	0.91437167	4.08263735	5.88338055
135.285	6.77686724	3.25778037	3.11468266	8.0304784	0.88698378	4.00352253	5.73654391
136.218	6.7135409	3.25177447	2.99412693	8.03519376	0.87492805	3.96886541	5.63157066
137.151	6.77070438	3.22346096	2.98503315	7.93385631	0.83828656	3.8780467	5.62865911
138.084	6.74696202	3.18996833	2.91758762	7.95258916	0.82353729	3.81759559	5.65828612
139.017	6.73259094	3.18665599	2.88487528	7.85549554	0.81355601	3.7450429	5.60713724
139.95	6.72644145	3.14273235	2.93021787	7.90963649	0.80434224	3.5873814	5.55638181
140.883	6.65431868	3.10358222	2.86447742	7.75587277	0.79214169	3.52210102	5.62700661
141.816	6.72263145	3.07526871	2.90331544	7.71103395	0.78103086	3.41102778	5.48430123
142.749	6.64406508	3.01263578	2.86611936	7.69324417	0.78252606	3.32339072	5.43008341
143.682	6.64141812	2.97396405	2.77998105	7.64669067	0.76292915	3.3115448	5.35819956
144.615	6.60272984	2.90165929	2.73072308	7.62311385	0.75714022	3.27495597	5.49032106
145.548	6.59949467	2.89275184	2.70767288	7.55671296	0.77265701	3.2674848	5.39239062
146.481	6.59803751	2.8547717	2.65033154	7.46677812	0.75972514	3.21393671	5.25361977
147.414	6.56636766	2.79103119	2.59400063	7.40290638	0.7491284	3.12013522	5.29505036
148.347	6.6112188	2.77528587	2.59589517	7.35528121	0.7522962	3.02298165	5.39467265
149.28	6.57751694	2.76044012	2.59179034	7.36912723	0.72636369	2.95261633	5.26565943
150.213	6.58630001	2.7212172	2.62393432	7.25068587	0.74788663	2.96849611	5.27435474
151.146	6.59480235	2.6861386	2.5466372	7.25385802	0.72792768	2.91341401	5.18799182
152.079	6.55376121	2.6625206	2.62058731	7.1816701	0.74828848	2.85830351	5.21946805
153.012	6.57369357	2.59484377	2.59216925	7.12242798	0.76041663	2.78180217	5.06000157
153.945	6.47725359	2.58510954	2.57808652	7.12821502	0.79210548	2.77788194	4.99043909
154.878	6.47109073	2.55437806	2.63896432	7.02336248	0.77050652	2.72498722	4.92583412
155.811	6.51095544	2.52517017	2.61780865	7.07047325	0.78729767	2.67868303	4.96289739
156.744	6.48897771	2.48981078	2.59627408	7.00634431	0.76067367	2.59445486	4.81295247
157.677	6.41685494	2.45459178	2.5568677	6.99837106	0.77738879	2.53516846	4.80197513
158.61	6.3280216	2.43290815	2.48702242	6.98152435	0.77400377	2.49914778	4.73386843
159.543	6.37756507	2.40259268	2.56804547	6.85686728	0.76216525	2.48315437	4.61418004

160.476	6.3312434	2.38146024	2.59330597	6.8386917	0.79574755	2.46824044	4.5530768
161.409	6.28228814	2.34368809	2.52156615	6.74104081	0.79428855	2.50980058	4.61894082
162.342	6.27965456	2.32987713	2.50059994	6.73825446	0.7655901	2.52193057	4.61418004
163.275	6.25795757	2.3374066	2.46030944	6.71746399	0.75728503	2.49014261	4.57420523
164.208	6.20929642	2.3431369	2.45917272	6.65466392	0.75120286	2.45446281	4.47895027
165.141	6.20519231	2.3198621	2.44319545	6.6570216	0.75167712	2.39034714	4.40128266
166.074	6.14830956	2.32905034	2.48222292	6.57728909	0.74027667	2.39185274	4.38967579
167.007	6.18818764	2.31088705	2.47388696	6.43629973	0.73925573	2.36657008	4.42677841
167.94	6.1761694	2.28284913	2.45045785	6.40625	0.74202529	2.29390376	4.34206799
168.873	6.15651779	2.23112562	2.45045785	6.3441358	0.73419449	2.25649111	4.35056657
169.806	6.08176143	2.22960725	2.45228923	6.26736111	0.7343031	2.20143742	4.16458137
170.739	6.02899616	2.18879835	2.43094411	6.20387517	0.73186299	2.17643884	4.27663676
171.672	6.01609561	2.15986085	2.45418377	6.20070302	0.73914712	2.1261576	4.12858042
172.605	6.00378327	2.15937726	2.50786233	6.12084191	0.7375071	2.11578888	4.20620869
173.538	5.99879684	2.13265492	2.53981686	6.10086591	0.72749324	2.11314698	4.1086717
174.471	5.96185982	2.13438129	2.47123461	6.08037551	0.72796388	2.12450997	4.05402109
175.404	5.9507239	2.11394044	2.47035049	6.0527692	0.72989715	2.13212317	4.05815234
176.337	5.86746521	2.07464472	2.44098516	5.91516632	0.7264723	2.1318391	4.02958766
177.27	5.88358756	2.08493534	2.42576571	5.95627572	0.71284896	2.10735186	3.99602613
178.203	5.84253305	2.08106661	2.45266814	5.88653121	0.73098687	2.08013749	4.01715455
179.136	5.82055533	2.0350786	2.4630881	5.88880316	0.71802242	2.1037157	4.03021719
180.069	5.82230659	2.05075113	2.43075466	5.86629801	0.72854676	2.10854497	3.86059962
181.002	5.77217491	2.02596315	2.41401958	5.83256173	0.70119507	2.07962616	3.83325464
181.935	5.74344612	1.94868728	2.36577202	5.78862311	0.72771046	2.059258	3.76388889
182.868	5.76720185	1.95462558	2.28999053	5.76020233	0.71284896	2.03227089	3.76243311
183.801	5.72702966	1.93163158	2.32876539	5.7129201	0.71048125	2.07133117	3.68932956
184.734	5.69859498	1.91236591	2.30483107	5.66332305	0.70119507	2.01744219	3.64152502
185.667	5.66048153	1.91105553	2.29055889	5.64171811	0.71274035	1.97326856	3.56924772
186.6	5.63028221	1.91623464	2.28512788	5.57758916	0.71245072	1.95863871	3.58270381

187.533	5.62706041	1.89613699	2.26473003	5.48778292	0.71926058	1.96375206	3.5066887
188.466	5.57663463	1.89150907	2.2529839	5.48002401	0.71372508	1.91324357	3.47045168
189.399	5.5171183	1.89275184	2.22904957	5.40543553	0.72151967	1.86787683	3.43421467
190.332	5.53148938	1.88419279	2.19823177	5.46832133	0.72632749	1.86097381	3.44314605
191.265	5.49571541	1.86837466	2.25191033	5.35853909	0.7232321	1.82233964	3.36713094
192.198	5.42800422	1.85111096	2.21086201	5.27889232	0.71882252	1.79697176	3.40482373
193.131	5.46054303	1.81810193	2.22128197	5.30907064	0.75699541	1.76282598	3.32117564
194.064	5.49044824	1.778879	2.21180928	5.28986626	0.78259846	1.70899381	3.26420365
194.997	5.4013208	1.80926728	2.16368803	5.18642833	0.79756858	1.68518834	3.24370475
195.93	5.45204069	1.78758365	2.17739185	5.14681927	0.82160765	1.66425203	3.23190116
196.863	5.42565138	1.76493284	2.2152826	5.135631	0.83941611	1.65331515	3.16210261
197.796	5.37846076	1.80187821	2.19709504	5.11552641	0.8613047	1.64913925	3.16107963
198.729	5.35060091	1.79338155	2.25449953	5.04471022	0.88927908	1.58351798	3.1517548
199.662	5.35969146	1.78744326	2.23814335	5.0258059	0.92235091	1.63959434	3.14431854
200.595	5.38343382	1.7963507	2.21401958	4.98139575	0.9725035	1.61516391	3.16997167
201.528	5.2852292	1.77045515	2.24704768	4.87379973	1.01365955	1.57192773	3.14349229
202.461	5.33740625	1.7500819	2.19134828	4.85819616	1.05379466	1.55502528	3.17618823
203.394	5.32128391	1.72964105	2.13154405	4.86685528	1.10824823	1.55792285	3.16603714
204.327	5.32128391	1.74290082	2.11651405	4.84113512	1.14638129	1.56235441	3.11779981
205.26	5.28727457	1.76369006	2.00467319	4.87264232	1.1926746	1.56127493	3.16729619
206.193	5.31307568	1.73882929	1.98408589	4.75977366	1.25732305	1.58883018	3.06189015
207.126	5.29021563	1.75188107	2.0178718	4.74056927	1.2608203	1.59641498	3.08341202
208.059	5.29226101	1.72964105	2.04477423	4.78034979	1.27320187	1.58576217	3.09977967
208.992	5.19346818	1.74255763	2.04553205	4.70794753	1.29024644	1.53968525	3.08238905
209.925	5.2491745	1.73178341	2.05127881	4.58011831	1.27338288	1.54650304	3.107452
210.858	5.24154111	1.70733863	2.05797284	4.60305213	1.25550201	1.5497699	3.02958766
211.791	5.20578052	1.69567003	2.0735712	4.59100652	1.24857992	1.56846202	3.09006138
212.724	5.15711937	1.70885701	2.03403852	4.56597222	1.21722052	1.53406056	3.0167611
213.657	5.17675762	1.66583815	2.07748658	4.45528978	1.20545803	1.54502585	3.03910922

214.59	5.19580765	1.63759223	2.13337543	4.44791667	1.20804295	1.54877564	3.06251967
215.523	5.11313717	1.65181919	2.14006947	4.39176097	1.22534457	1.50857906	2.9343327
216.456	5.12016898	1.64159616	2.19115883	4.43865741	1.18407629	1.49394921	2.90824677
217.389	5.14392471	1.61349065	2.10369435	4.39437586	1.18549908	1.51474348	2.93700818
218.322	5.05509137	1.61224787	2.14827913	4.33676269	1.21827766	1.54934379	3.02691218
219.255	5.09848535	1.64083698	2.15213135	4.35163752	1.18451435	1.51752741	3.02730563
220.188	5.0632996	1.64712367	2.14063783	4.29368141	1.1793445	1.51511278	3.00889204
221.121	5.1213454	1.64470571	2.1617935	4.23735425	1.17096703	1.57087665	3.0163283
222.054	5.04424956	1.62951157	2.19671614	4.19624486	1.19096218	1.59635816	2.97552723
222.987	5.00584201	1.59139103	2.15863593	4.14566187	1.16732497	1.58962559	2.94220176
223.92	4.99001377	1.58897827	2.12188191	4.11076818	1.18298656	1.59968184	2.95959238
224.853	5.01697793	1.58027362	2.20789391	4.15222051	1.14583462	1.58655758	2.97926503
225.786	4.92990923	1.52944969	2.17088728	4.08620542	1.13884011	1.57723993	2.98587504
226.719	4.92258332	1.56308271	2.21844016	4.08590535	1.12263546	1.61772058	2.94157224
227.652	4.94339799	1.55934918	2.25468898	4.07420267	1.12255944	1.55530936	2.98363236
228.585	4.96743446	1.56653546	2.21180928	4.0774177	1.11214371	1.56261008	2.90533522
229.518	4.96480088	1.55666083	2.24894222	4.04050926	1.06931145	1.57959775	2.9790683
230.451	4.92462869	1.59498417	2.25784654	3.99858539	1.08216728	1.59797739	2.92603085
231.384	4.89678221	1.57737727	2.27622356	3.96090535	1.06279121	1.607153	2.92418162
232.317	4.86130235	1.56487668	2.26435112	3.93055556	1.08359008	1.59252315	2.85812087
233.25	4.76074489	1.56584387	2.22608147	3.95940501	1.07386584	1.59400034	2.86661945
234.183	4.73876716	1.5524437	2.3167035	3.95143176	1.07065821	1.61249361	2.80594901
235.116	4.74022432	1.55873039	2.27736028	3.96965021	1.06188251	1.6474348	2.78419106
236.049	4.74785771	1.56556827	2.28127566	3.86201132	1.0603149	1.64939492	2.80406043
236.982	4.68687085	1.5463026	2.2203347	3.95276063	1.06792848	1.64726436	2.80138495
237.915	4.67544082	1.51032442	2.2301863	3.85691015	1.03740537	1.63257769	2.80118823
238.848	4.64406508	1.5465054	2.24130092	3.85395233	1.03995771	1.65297426	2.75912811
239.781	4.58807802	1.55845479	2.34474266	3.84126372	1.02837262	1.68564286	2.77156122
240.714	4.65550847	1.53663077	2.26359331	3.82017318	0.98514212	1.67598432	2.67485049

241.647	4.58690159	1.56791343	2.2782444	3.81893004	0.95855433	1.65075848	2.68995908
242.58	4.57869337	1.57647768	2.24654247	3.81014232	0.93925066	1.68797227	2.76388889
243.513	4.58162106	1.56038916	2.27306599	3.75904492	0.91830698	1.67433669	2.77341045
244.446	4.55640817	1.53994831	2.26454057	3.71789266	0.89827563	1.65899665	2.67898174
245.379	4.58162106	1.52799892	2.40846227	3.72436557	0.8581731	1.64115675	2.69743469
246.312	4.53354812	1.51418796	2.33305968	3.69157236	0.86214462	1.64678143	2.61126849
247.245	4.55289226	1.50528051	2.34865804	3.68171296	0.85129083	1.66482018	2.62952471
248.178	4.45498175	1.53110846	2.3163246	3.60052298	0.82251635	1.65609909	2.6634797
249.111	4.48429876	1.51522274	2.33343859	3.62765775	0.83493775	1.62178285	2.65643689
250.044	4.48048875	1.52357899	2.31840859	3.62795782	0.8180742	1.62107267	2.6015502
250.977	4.44324425	1.49153715	2.29908431	3.57308813	0.7931228	1.64413954	2.59761568
251.91	4.45468765	1.48995117	2.37467635	3.58333333	0.78653378	1.6420942	2.60941926
252.843	4.46816304	1.50935724	2.3444269	3.5988083	0.78176217	1.64939492	2.67134876
253.776	4.43562424	1.48428847	2.38825387	3.60335219	0.7891187	1.64635532	2.66989298
254.709	4.42419422	1.4594953	2.35850963	3.59675069	0.75681077	1.60908471	2.71793359
255.642	4.38050613	1.4827701	2.37284496	3.57656036	0.76868549	1.63817397	2.70302172
256.575	4.39956954	1.46578199	2.42349226	3.54406722	0.74428076	1.62340208	2.67052251
257.508	4.42360601	1.45314621	2.38193874	3.53450789	0.75619169	1.6227203	2.6241344
258.441	4.40337954	1.45880371	2.47533944	3.51393176	0.74869034	1.61368672	2.58353006
259.374	4.34240605	1.44506554	2.43391222	3.54471022	0.74974748	1.57312085	2.63778722
260.307	4.38022539	1.44243959	2.45361541	3.48825446	0.76587972	1.56255326	2.58789739
261.24	4.38432951	1.41481767	2.42222924	3.51718964	0.75622789	1.51383444	2.56861819
262.173	4.36438378	1.41709523	2.42557626	3.51251715	0.73849184	1.50829498	2.54831602
263.106	4.34533374	1.42254993	2.4227976	3.46274863	0.74009565	1.48869382	2.55972616
264.039	4.27907972	1.37994186	2.4203347	3.49159808	0.75925088	1.48434748	2.54855209
264.972	4.27291686	1.37083162	2.42500789	3.48105281	0.76005459	1.48309755	2.60257318
265.905	4.24301164	1.34368809	2.41850332	3.48100995	0.75506214	1.46284302	2.56074913
266.838	4.28758205	1.34714083	2.4177455	3.47509431	0.76274813	1.44602579	2.51955461
267.771	4.27262276	1.34141053	2.4532365	3.46257716	0.74686931	1.45182092	2.54855209

268.704	4.22835983	1.32311204	2.44212188	3.44371571	0.75677456	1.45594	2.45908089
269.637	4.1978664	1.32794276	2.43669087	3.36616941	0.76125655	1.46156468	2.54564054
270.57	4.18056762	1.32359563	2.46738238	3.32377401	0.73823479	1.4680984	2.45077904
271.503	4.09438124	1.29783528	2.4584149	3.34357853	0.74628643	1.47639339	2.44664778
272.436	4.17060813	1.29839167	2.47811809	3.34139232	0.76628158	1.51184592	2.44810356
273.369	4.13630469	1.31164624	2.46757183	3.29385288	0.76671964	1.50085222	2.4423198
274.302	4.13748112	1.30377358	2.45494159	3.29805384	0.75160472	1.50232941	2.39797765
275.235	4.14187934	1.29576571	2.45209978	3.29492455	0.7624223	1.50068178	2.39073812
276.168	4.12399235	1.28374872	2.48089675	3.29119513	0.75943552	1.51619226	2.36941297
277.101	4.13893828	1.25157167	2.43372277	3.26131687	0.73546885	1.5190046	2.3808231
278.034	4.12544951	1.27663524	2.44913167	3.22749486	0.74406354	1.50374979	2.35926188
278.967	4.11724129	1.25281445	2.42443953	3.16045096	0.74923701	1.53891824	2.39345294
279.9	4.11490181	1.27021335	2.47142406	3.22856653	0.75790411	1.53812283	2.31991659
280.833	4.06828603	1.26330787	2.48443322	3.12362826	0.74715894	1.5490029	2.35139282
281.766	4.07239014	1.26006313	2.5316072	3.16803841	0.74712273	1.52539628	2.40006295
282.699	4.06037191	1.2382391	2.46214083	3.16460905	0.76191183	1.54138969	2.42469311
283.632	4.06447602	1.24880532	2.43599621	3.18458505	0.75786791	1.51667519	2.37747875
284.565	4.05803243	1.25115568	2.50413641	3.19024348	0.76318619	1.50346571	2.41186654
285.498	4.03252543	1.25184727	2.50337859	3.22492284	0.7624223	1.51775467	2.42803746
286.431	4.05715011	1.24452579	2.47256078	3.15655007	0.73849184	1.52397591	2.3511961
287.364	4.04014545	1.2499129	2.50078939	3.13901749	0.75033036	1.53400375	2.41351904
288.297	4.06418192	1.23727712	2.47016104	3.12109911	0.78409005	1.52085109	2.36878344
289.23	4.07737658	1.26510184	2.50243132	3.12028464	0.80401641	1.5178683	2.45845137
290.163	4.07649426	1.26593383	2.49150616	3.14304698	0.79826006	1.52249872	2.38597734
291.096	4.027539	1.26075472	2.55112093	3.08329047	0.78041178	1.50508494	2.32054611
292.029	4.03926313	1.25688598	2.4836754	3.07621742	0.77793546	1.45048577	2.31000157
292.962	4.07121372	1.2443178	2.52453426	3.12688615	0.79366947	1.45537185	2.26278722
293.895	4.0888066	1.27256371	2.48279129	3.05208333	0.80073638	1.46772911	2.36115046
294.828	4.06506424	1.25675078	2.54910009	3.06447188	0.79352466	1.45071303	2.38806264

295.761	4.04132187	1.23934668	2.54240606	3.03365055	0.80121064	1.4377024	2.32011331
296.694	4.01053434	1.21324313	2.49112725	3.07557442	0.80547178	1.44835521	2.35595688
297.627	3.81617047	1.18099329	2.51304073	3.07193073	0.82576018	1.45531504	2.47501574
298.56	4.00232611	1.17029187	2.51304073	3.07518861	0.82350109	1.43812852	2.54646679
299.493	3.98766092	1.1688411	2.513988	3.09747942	0.81712567	1.44460542	2.53962071
300.426	3.93137976	1.12726781	2.50729397	3.05761317	0.80823773	1.45062781	2.53859773
301.359	3.83903052	1.13790163	2.54404799	3.03120713	0.79428855	1.46534288	2.59076959
302.292	3.84900338	1.13873361	2.57177139	3.06558642	0.80419742	1.45054258	2.88050834
303.225	3.83961873	1.10172065	2.579539	3.06267147	0.79997249	1.43741833	3.17847025
304.158	3.84635643	1.11083609	2.57044522	3.09319273	0.79967924	1.42412363	3.25220334
305.091	3.82495354	1.0990271	2.63656457	3.05478395	0.78846342	1.45451963	3.40832546
306.024	3.78098472	1.09730073	2.54777392	3.00510117	0.7844919	1.44366797	3.43382121
306.957	3.77629239	1.11373244	2.6252605	2.98915466	0.79155881	1.42597012	3.51683979
307.89	3.72498429	1.11428883	2.62058731	2.96960734	0.79286937	1.14808818	3.67630626
308.823	3.74726949	1.10358222	2.61465109	3.00981653	0.7836194	1.24609397	3.68087032
309.756	3.76280363	1.08783689	2.56059362	3.03125	0.80816532	1.59380149	3.79453887
310.689	3.78039651	1.05766181	2.61559836	3.00205761	0.81417509	2.40128402	3.83844822
311.622	3.8240846	1.06332451	2.55314177	2.95987654	0.85085277	3.25535481	3.91796506
312.555	3.84342874	1.06276812	2.59532681	2.96587791	0.83726925	3.51031191	3.84092698
313.488	3.7950617	1.0350786	2.58402273	2.94414438	0.84021983	3.6052497	3.95813661
314.421	3.78098472	1.05075633	2.60221029	3.00428669	0.85081657	3.5862451	3.96828769
315.354	3.76280363	1.0523423	2.55876224	3.02207647	0.82903659	3.59439805	4.02274158
316.287	3.73232357	1.05303389	2.59141143	2.99528464	0.83271486	3.57238225	4.02108908
317.22	3.76486237	1.08859608	2.59368488	2.95404664	0.82317164	3.54675871	4.05299811
318.153	3.77541008	1.06774443	2.58383328	2.97612311	0.84466199	3.49758536	4.05091281
319.086	3.7440477	1.05648663	2.51101989	2.95486111	0.83289588	3.46224646	4.16564369
320.019	3.78450062	1.06532648	2.61149353	2.90805041	0.82080393	3.40795978	4.1701684
320.952	3.7730706	1.0639433	2.60593622	2.93668553	0.84510005	3.32395887	4.24929178
321.885	3.8020935	1.07147277	2.59867382	2.95014575	0.83359098	3.30677234	4.27104973

322.818	3.77482187	1.04895716	2.62450268	2.92939815	0.82583259	3.24149196	4.2002282
323.751	3.8091253	1.07181596	2.59513735	2.91872428	0.84557069	3.2233964	4.24016368
324.684	3.77951419	1.07464992	0	2.92061043	0.84338763	3.0976365	4.19751338
325.617	3.80853709	1.06270052	0	2.88211591	0.8439705	3.06013863	4.22485836
326.55	3.79153243	1.06069856	0	2.91199417	0.86374481	3.01014147	4.07369374
327.483	3.75019718	1.06670445	0	2.84872257	0.85158046	2.91636839	4.21431382
328.416	3.75488951	1.05303389	0	2.88220165	0.84156659	2.84685529	4.20105445
329.349	3.71824659	1.07257515	0	2.85043724	0.89441273	2.79111982	4.22568461
330.282	3.67779367	1.05648663	0	2.85708162	0.8690993	2.74833816	4.1797293
331.215	3.73085304	1.05662703	0	2.84083505	0.85475188	2.68621101	4.22466163
332.148	3.73085304	1.05531145	0	2.8388203	0.84214947	2.63962275	4.14679729
333.081	3.68863548	1.0640837	0	2.79916838	0.83034715	2.57721152	4.13829871
334.014	3.68219189	1.05682982	0	2.81738683	0.82386312	2.55795125	4.10556342
334.947	3.63879791	1.06035536	0	2.78287894	0.82998512	2.47008693	4.07408719
335.88	3.686296	1.07299114	0	2.76984739	0.82728797	2.44736095	4.16005666
336.813	3.65169846	1.04612841	0	2.76067387	0.83566544	2.42321459	4.03808624
337.746	3.56608692	1.04709559	0	2.74361283	0.83078522	2.37435373	4.16623387
338.679	3.67104261	1.06297612	0	2.76363169	0.88603525	2.31884552	4.07015266
339.612	3.61886555	1.04972154	0	2.75857339	0.89969481	2.28893245	4.17744728
340.545	3.64084328	1.06221693	0	2.80765604	0.92271294	2.26697347	4.05547686
341.478	3.61710092	1.0756847	0	2.81142833	0.93061615	2.21299926	4.07015266
342.411	3.60420037	1.09619315	0	2.78587963	0.95086834	2.18348389	3.99437362
343.344	3.68863548	1.08549173	0	2.85695302	0.94095946	2.15570138	4.07491344
344.277	3.67104261	1.08818528	0	2.75578704	0.93557239	2.13141299	4.01613157
345.21	3.66137722	1.08424895	0	2.48070988	0.91353537	2.09411397	3.97096317
346.143	3.71942302	1.06891441	0	2.31815844	0.91273528	2.04525311	3.86185867
347.076	3.69244549	1.07257515	0	2.51937586	0.91087804	2.05150276	3.94570349
348.009	3.69743192	1.06353251	0	2.78613683	0.91167814	2.02176013	3.97076645
348.942	3.69772603	1.04688759	0	2.78566529	0.89783757	1.99377876	3.88070507

349.875	3.63673917	1.05758901	0	3.05902778	0.89678043	1.95917846	3.86992446
350.808	3.65052204	1.04509362	0	3.23096708	0.89532505	1.90563036	3.91859459
351.741	3.64700614	1.04260807	0	3.35193759	0.89190021	1.89051758	3.92170286
352.674	3.65843616	1.0409493	0	3.44131516	0.89911193	1.85813306	3.75188857
353.607	3.65258078	1.00427953	0	3.46257716	0.89477838	1.85679791	3.78604029
354.54	3.67339545	0.98867459	0	3.48092421	0.87900817	1.79350605	3.69432641
355.473	3.63088379	0.97720879	0	3.49069787	0.91739466	1.77518323	3.71750079
356.406	3.6349879	0.95090244	0	3.48010974	0.91859661	1.75103687	3.68995908
357.339	3.62970736	0.95635715	0	3.50184328	0.89040863	1.71018692	3.67174221
358.272	3.67016029	0.94910328	0	3.48152435	0.91022276	1.69961934	3.68043752
359.205	3.65140436	0.93639989	0	3.47856653	0.91641354	1.68300097	3.67713251
360.138	3.60889269	0.92520969	0	3.44727366	0.92285775	1.65180956	3.56240164
361.071	3.58163443	0.91402468	0	3.45297497	0.93072838	1.61362991	3.57668398
362.004	3.61857145	0.91360869	0	3.45220336	0.92883132	1.62675416	3.50090494
362.937	3.59159392	0.91215791	0	3.48358196	0.93593443	1.5835748	3.53899119
363.87	3.5801639	0.91319789	0	3.46840706	0.92697408	1.55403102	3.39321687
364.803	3.59746267	0.91809621	0	3.44358711	0.89896712	1.54593489	3.43775574
365.736	3.57372031	0.9301808	0	3.41345165	0.92679306	1.50664735	3.3975842
366.669	3.56257102	0.9067708	0	3.37890089	0.95665727	1.47383671	3.41206327
367.602	3.57195567	0.89413502	0	3.41752401	0.94991981	1.45332652	3.43441139
368.535	3.57635389	0.91678583	0	3.38100137	0.94099929	1.43500369	3.39860718
369.468	3.58309159	0.91954698	0	3.34696502	0.93863158	1.39977842	3.31700504
370.401	3.53062043	0.90974515	0	3.39218964	0.92988846	1.39557412	3.34187126
371.334	3.57635389	0.91243351	0	3.353738	0.94190799	1.4011988	3.37004249
372.267	3.49368341	0.78861844	0	3.38022977	0.93633629	1.39756264	3.3306972
373.2	3.51976525	0.37987427	0	3.34014918	0.92828465	1.38392705	3.29815864
374.133	3.53706402	0.32497881	0	3.35343793	0.9411803	1.3940117	3.34104501
375.066	3.52152989	1.10814254	0	3.35120885	0.93979733	1.38912562	3.30956878
375.999	3.50305469	1.45666655	0	3.36316872	0.94282032	1.36412704	3.26609223

376.932	3.52064757	1.65292157	0	3.28472222	0.93152847	1.3529629	3.23190116
377.865	3.5224122	1.69414646	0	3.24112654	0.93677435	1.35233794	3.18177526
378.798	3.47286874	1.73620334	0	3.21630658	0.91670317	1.3510596	3.21037929
379.731	3.45556996	1.79207118	0	3.20507545	0.93812111	1.34907108	3.18551306
380.664	3.45264227	1.76403326	0	3.15436385	0.93633629	1.34054883	3.1538401
381.597	3.44500889	1.79455673	0	3.1663666	0.93874019	1.35972388	3.12444917
382.53	3.44325762	1.76369006	0	3.15342078	0.94631395	1.34267939	3.11429808
383.463	3.45409943	1.73185101	0	3.16589506	0.94059743	1.37557525	3.16686339
384.396	3.4743259	1.75201627	0	3.17866941	0	1.39494915	3.17618823
385.329	3.42947475	1.70768183	0	3.18046982	0	1.3913414	3.20270696
386.262	3.43416708	1.69062612	0	3.17511145	0	1.36574626	3.10206169
387.195	3.40045185	1.72280837	0	3.18981481	0	1.36276348	3.09977967
388.128	3.43709477	1.69546204	0	3.11964163	0	1.37656951	3.1037142
389.061	3.37816665	1.68910775	0	3.12281379	0	1.3708028	3.18118508
389.994	3.35560071	1.73931288	0	3.05709877	0	1.37818874	3.15816808
390.927	3.37319359	1.72971385	0	3.05366941	0	1.40125561	3.10993075
391.86	3.37523896	1.72321916	0	3.06434328	0	1.43832737	3.14077746
392.793	3.32657781	1.75063829	0	3.03785151	0	1.471138	3.06291313
393.726	3.34445143	1.7559526	0	3.07968964	0	1.47306971	3.11595058
394.659	3.34884965	1.74000447	0	3.05838477	0	1.51212999	3.09419263
395.592	3.34621606	1.77549906	0	3.0304784	0	1.52156127	2.98465534
396.525	3.34943786	1.75091388	0	3.04633916	0	1.50943128	3.03993547
397.458	3.38110771	1.75450182	0	3.01564643	0	1.55062212	3.06543122
398.391	3.34884965	1.79455673	0	2.99361283	0	1.57235384	3.05465061
399.324	3.33800783	1.85601448	0	3.00861626	0	1.57945571	2.94322474
400.257	3.36116199	1.87970007	0	3.02490569	0	1.61255042	2.98756689
401.19	3.34387658	1.8953778	0	3.01427469	0	1.58726777	2.94861505
402.123	3.35530661	1.9591183	0	3.05096879	0	1.62198171	2.96041863
403.056	3.29198027	1.99164374	0	2.99074074	0	1.64090109	2.87177369

403.989	3.33097603	1.98024554	0	2.99112654	0	1.64891199	2.92024709
404.922	3.31279494	2.06636126	0	2.94058642	0	1.66899608	2.91485678
405.855	3.35911661	2.14639828	0	2.96077675	0	1.70870973	2.87511804
406.788	3.31542853	2.18928194	0	3.00222908	0	1.73569684	2.89187913
407.721	3.37759181	2.2560592	0	2.98353909	0	1.74194648	2.83018571
		2.34452007	0	2.96416324	0	1.77041077	2.87740006
		2.49899382	0	2.95777606	0	1.78626214	2.90242367
		2.89095788	0	2.92026749	0	1.80367593	2.82601511
		3.16414038	0	2.91872428	0	1.85898528	2.86701291
		3.43960564	0	2.9113083	0	1.8605477	2.81362134
		3.77141713	0	2.92609739	0	1.86537697	2.87138023
		4.22380415	0	2.89433299	0	1.8898074	2.81960183
		4.5058993	0	2.89334705	0	1.90997671	2.89707271
		4.76313367	0	2.95186043	0	1.91253338	2.87594429
		5.00427433	0	2.906893	0	1.95281518	2.96663519
		5.07146757	0	2.91443759	0	1.97247315	2.9268571
		4.99481569	0	2.90955075	0	1.98369411	2.92335537
		5.11842276	0	2.90286351	0	1.99227317	2.93681146
		5.02810032	0	2.85429527	0	2.01528322	2.96415644
		4.872103	0	2.82527435	0	2.05187205	3.03186969
		4.84793382	0	2.83972051	0	2.08431339	3.00889204
		4.73578734	0	2.84559328	0	2.09036418	2.94924457
		4.66783491	0	2.90097737	0	2.15206522	2.97304847
		4.57412733	0	2.86977023	0	2.17686495	2.92563739
		4.45099864	0	2.85506687	0	2.20967559	2.97800598
		4.30874469	0	2.85999657	0	2.23325379	2.99563267
		4.25674558	0	2.92253944	0	2.28092154	2.88857413
		4.25384403	0	2.87071331	0	2.32302142	2.96250393
		4.10502779	0	2.82608882	0	2.31549344	2.89250866

3.98570025	0	2.84057785	0	2.34432703	2.94054926
3.97713599	0	2.80954218	0	2.36137151	2.90387945
3.90055691	0	2.85832476	0	2.41307312	2.93496223
3.83052971	0	0	0	2.43074257	2.94963802
3.84462147	0	0	0	2.4259133	2.87488196
3.70982419	0	0	0	2.44687802	0
3.53704156	0	0	0	2.44801432	3.39223324
3.55575604	0	0	0	2.44920743	3.40690903
3.45272501	0	0	0	2.45980342	3.37924929
3.32731877	0	0	0	2.43983296	3.40238432
3.29023821	0	0	0	2.45653656	3.42953258
3.14073038	0	0	0	2.47613772	3.52101039
3.00448232	0	0	0	2.5421851	3.50259679
3.03611338	0	0	0	2.5288904	3.53989613
2.98183671	0	0	0	2.55582069	3.60225842
2.92928122	0	0	0	2.60084654	3.69727731
2.89233585	0	0	0	2.6694506	3.73359301
2.78005418	0	0	0	2.6956707	3.7546034
2.71272574	0	0	0	2.71084029	3.86890148
2.65982705	0	0	0	2.79336401	3.88141328
2.64014539	0	0	0	2.80239759	3.93885741
2.57102298	0	0	0	2.84387251	3.96214983
2.58621192	0	0	0	2.86068973	4.0434372
2.55085773	0	0	0	2.91937958	4.04225685
2.50196297	0	0	0	2.91852736	4.14896128
2.4827649	0	0	0	2.99556843	4.09092698
2.46799195	0	0	0	2.95366741	4.23178313
2.47233908	0	0	0	3.05536617	4.24291785
2.46605758	0	0	0	3.06340549	4.2449638

2.39748116	0	0	0	3.06488268	4.22816336
2.2906542	0	0	0	3.05275268	4.28765345
2.2326492	0	0	0	3.09570479	4.33762197
2.1804421	0	0	0	3.13178229	4.38719704
2.13852562	0	0	0	3.16757571	4.38818067
2.16455637	0	0	0	3.16828589	4.49008499
2.13859322	0	0	0	3.24186126	4.49217029
2.05061593	0	0	0	3.24336685	4.4744649
2.0522019	0	0	0	3.23058349	4.54567989
2.02817312	0	0	0	3.28200102	4.55343091
2.07789466	0	0	0	3.2875973	4.59128108
2.05897219	0	0	0	3.31492529	4.59710419
2.04798997	0	0	0	3.31912959	4.58478911
2.08300097	0	0	0	3.31023805	4.6237803
2.06552927	0	0	0	3.33838986	4.63027227
2.01850648	0	0	0	3.3430771	4.63086245
2.03272824	0	0	0	3.34350321	4.62393768
1.99537208	0	0	0	3.33850349	4.64038401
2.01960886	0	0	0	3.35037782	4.64620711
2.01553733	0	0	0	3.35458213	4.59875669
1.97714119	0	0	0	3.32032271	4.68413598
1.96277384	0	0	0	3.38196693	4.62079005
1.94129821	0	0	0	3.3362309	4.68543437
1.88508718	0	0	0	3.38290438	4.68681146
1.85504729	0	0	0	3.38389864	4.63428549
1.82929214	0	0	0	3.34597466	4.67744728
1.82929214	0	0	0	3.37713766	4.69074599
1.85587928	0	0	0	3.38673939	4.7123859
1.78295573	0	0	0	3.4023635	4.69239849

1.75843815	0	0	0	3.3879325	4.73284545
1.74835553	0	0	0	3.41713539	4.73599308
1.67723115	0	0	0	3.41940799	4.73402581
1.70492068	0	0	0	3.41798761	4.7629446
1.77238951	0	0	0	3.43886711	4.77998111
1.72888186	0	0	0	3.45116755	4.7658955
1.71127497	0	0	0	3.44099767	4.74740321
1.71548689	0	0	0	3.42764616	4.7220255
1.69587283	0	0	0	3.40932333	4.69290998
1.65009282	0	0	0	3.3883018	4.72851747
1.63186193	0	0	0	3.41401057	4.80968681
1.59525976	0	0	0	3.47261519	4.75889204
1.60748475	0	0	0	3.43594114	4.78214511
1.56867262	0	0	0	3.45272996	4.7541706
1.57696128	0	0	0	3.43253224	4.68130312
1.53311043	0	0	0	3.3799216	4.74307523
1.49858302	0	0	0	3.42452133	4.77671545
1.49595707	0	0	0	3.44880973	4.80524079
1.54167468	0	0	0	3.46940515	4.77525968
1.48117892	0	0	0	3.44775865	4.77659742
1.50210336	0	0	0	3.4487245	4.73815707
1.4826973	0	0	0	3.4209704	4.76322002
1.44886148	0	0	0	3.43906596	4.7287142
1.39444442	0	0	0	3.4141526	4.7658955
1.38471018	0	0	0	3.44099767	4.78057129
1.32283645	0	0	0	3.45159366	4.78958137
1.30971707	0	0	0	3.45809897	4.80169972
1.30142842	0	0	0	3.46684847	4.81173277
1.26911097	0	0	0	3.47409238	4.83396286

1.27421728	0	0	0	3.49014261	4.78399433
1.25716158	0	0	0	3.45406511	4.7495672
1.23789591	0	0	0	3.42920857	4.75889204
1.23161442	0	0	0	3.43594114	4.77250551
1.19798139	0	0	0	3.44577013	4.73316021
1.18223607	0	0	0	3.41736265	4.77474819
1.18286005	0	0	0	3.44738935	4.72371734
1.13175533	0	0	0	3.41054486	4.71899591
1.12588983	0	0	0	3.40713596	4.70479226
1.11684199	0	0	0	3.39688086	4.71840573
1.10379021	0	0	0	3.40670985	4.72938307
1.11836036	0	0	0	3.41463553	4.7474819
1.14619029	0	0	0	3.42770297	4.74575071
1.12326388	0	0	0	3.42645304	4.68279824
1.10172065	0	0	0	3.38100108	4.6877164
1.12913458	0	0	0	3.38455201	4.688582
1.10199625	0	0	0	3.38517698	4.71218917
1.10233944	0	0	0	3.40222146	4.68307365
1.08203899	0	0	0	3.38119993	4.69153289
1.0641513	0	0	0	3.38730754	4.65690903
1.05082393	0	0	0	3.36230896	4.688582
1.04536922	0	0	0	3.38517698	4.67854895
1.04398604	0	0	0	3.37793307	4.69468052
1.05393347	0	0	0	3.38958014	4.68653604
1.03652937	0	0	0	3.38369979	4.64813503
1.03604578	0	0	0	3.35597409	4.66997167
1.01436215	0	0	0	3.37174024	4.6605288
1.01588053	0	0	0	3.36492245	4.63971514
1.00697308	0	0	0	3.34989489	4.66902738

0.99074416	0	0	0	3.37105846	4.6923198
0.9997244	0	0	0	3.38787569	4.65029902
1.00939104	0	0	0	3.3575365	4.63263299
1.06325171	0	0	0	3.34478155	4.58766132
1.07920504	0	0	0	3.3123118	4.61067831
1.07913744	0	0	0	3.32893017	4.62692792
1.09688473	0	0	0	3.34066246	4.65612213
1.13016936	0	0	0	3.36174081	4.63467894
1.13168773	0	0	0	3.34625874	4.61469153
1.11497522	0	0	0	3.33182774	4.62944602
1.1279594	0	0	0	3.34248054	0
1.14052758	0	0	0	0	0
1.09819511	0	0	0	0	0
1.0874209	0	0	0	0	0
1.05144792	0	0	0	0	0
1.06242493	0	0	0	0	0
1.07015719	0	0	0	0	0
1.06214933	0	0	0	0	0
1.06511848	0	0	0	0	0
1.06898721	0	0	0	0	0
1.06594527	0	0	0	0	0
1.07444192	0	0	0	0	0
1.08770169	0	0	0	0	0
1.07050559	0	0	0	0	0
1.10675936	0	0	0	0	0
1.10337422	0	0	0	0	0
1.10219904	0	0	0	0	0
1.08176339	0	0	0	0	0
1.07478511	0	0	0	0	0

1.0815554	0	0	0	0	0
1.06325171	0	0	0	0	0
1.09646874	0	0	0	0	0
1.0874209	0	0	0	0	0
1.07381793				0	

Figure 5B

WT	WT + ISO	WT + ISO + Propranonol	2808D	2808D+S107	WT+ S107	WT+ISO+S107
	3.76224189	7.2071251	7.51677852	7.72888889	4.37481481	3.762345
2.64212963	1.72532189	6.23204787	4.53073967	10.3076923	5.29457364	5.232345
2.88596491	1.85462754	6.43878273	3.88222698	11.981982	4.24489796	5.2345
3.22823529	3.11615245	6.73156682	4.59479554	6.94610778	3.423423	5.37481481
2.80264609	1.46401515	6.78065499	4.7164592	3.04347826	3.55788097	4.04371585
4.31838565	3.07142857	9.39454355	3.77234043	4.57414449	6.0532	3.33632287
3.24466223	1.44664511	3.97927807	3.07705363	2.73221757	4.336	3.32453425
6.22897196	3.214	6.61810756	1.54812734	6.77835052	4.69	5.2345235
4.4314759	2.345	9.3531746	6.23809524	4.66208791	5.79235423	3.23452345
4.23469994	4.213	10.25	4.89268293	3.42823894	6.74345213	4.62354
2.34155421	1.89	7.70226537	3.01152637	6.03108808	4.54134523	5.32469
2.68464961	1.567	8.42124542	2.83473313	6.71987382	3.8223423	4.2568
3.32028924	1.689	5.67		6.99438202	5.893425342	6.53247
2.71641791	2.213	9.39673913	1.62743473	6.38495575	6.212134123	8.230894
3.2096486	2.894	7.48028674	3.2027972		5.346534	5.8937
6.616	3.789	6.672	4.72019465	6.28313253	6.32786885	5.32871
4.53051948	2.867	8.36403509	1.75983437	4.1671159	3.6835879	7.7635627
4.20059347	3.357	8.61538462	2.15466667	4.14669927	6.41225166	6.321447
3.44949066	3.792	4.67	1.82897727	5.69871795	4.63131	4.256457
6.6888361	3.85505155	5.76	2.02329133	4.42345277	7.22761194	5.231247
2.8245804	4.20795756	6.74	2.10732538	4.68014706	4.39320388	8.2145663
3.37455986	3.18080495	4.541	1.79096045	5.57567568	4.66381766	4.786924
3.49238095	2.6005988	4.82	2.02713987	5.96835443	6.47122507	5.321147
3.83505155	3.25777778	3.89	1.79108635	5.13577023	3.34562345	4.02329133
4.50795756	4.26097561	4.21	2.35561497	7.71287129	4.456345	4.10732538
3.38080495	3.55384615	5.11	1.75	6.61081081	2.21342134	4.732452345
2.7005988		3.782	3.93293413	3.90873786	6.5648567	4.23452345

3.05777778	4.21	5.22074468	5.7275641	4.2345234	5.314654
4.56097561	3.44	2.68333333	7.23986486	3.789567	6.3124475
3.15384615	4.11	1.17401961	5.20506912	6.23452354	3.345324512
	5.678	3.29768271	6.16816817		4.4325234
2.76976629	4.567	1.95951417	5.38738739		6.32147
4.23352903	6.324	1.79742173	4.57007126		3.221457
4.67605634	4.445	1.40178571	7.03125		5.2564124
5.37481481	5.778	1.83673469	5.71270718		4.2254478
5.04371585		1.82931727	7.66772152		3.214878
3.33632287		1.93137255	5.89473684		4.21477
3.63131313		1.4361949	4.80962343		3.21478
5.22761194		1.51136364	5.81661891		5.23127
3.39320388		2.07276995	3.91923077		
2.66381766			3.85067712		
3.29457364		1.54761905	5.75		
5.32786885		2.71181319	5.08371041		
2.68358797		1.40109523	6.485623		
5.41225166		1.75143403	4.94004796		
5.47122507		3.60167131	5.48082596		
2.24489796		1.51047031	6.04034582		
2.55788097		2.72289157	5.88235294		
2.41609267		4.07692308	6.41237113		
4.8707552		4.14507772	6.04792332		
4.36279548		2.33479769	4.98704104		
		1.70094325	5.46282974		
		2.65374176	4.16159696		
		2.59802539	3.80573951		
		3.5501845			
		4.1580756			

2.31130614
5.22167488
3.14089232
3.78765432
2.55865646
2.15946206
3.06949289
3.57142857
2.4429498
1.84077543
3.62429379
1.75554871
3.27341772
1.76416778
3.01030928
