# Supplementary material for: Heart failure-induced cognitive dysfunction is mediated by intracellular Ca2+ leak through ryanodine receptor type 2
Source: Nat Neurosci. 2023 Jul 10;26(8):1365–78. doi: 10.1038/s41593-023-01377-6 (PMC10400432; doi:10.1038/s41593-023-01377-6)
Supplement: Source Data Fig. 6 — Statistical source data. [file 41593_2023_1377_MOESM9_ESM.pdf]

Figure 6

Comparison (group1/group2)	AVG Log2 Ratio	Pvalue	Qvalue	UniProtIds
MI / SHAM	-2.5993	8.51E-11	4.7E-08	Q3UQ44
MI / SHAM	-2.5378	0.003918	0.003459	Q99JP6;Q99JP6-2
MI / SHAM	-2.47437	1.88E-05	9.7E-05	Q8K3Z9
MI / SHAM	-2.12531	0.072035	0.032504	Q02788
MI / SHAM	-2.06366	0.036449	0.018757	Q8R4I7
MI / SHAM	-2.05492	2.01E-08	1.39E-06	Q3UH99-2
MI / SHAM	-2.04179	3.29E-08	1.65E-06	Q8BHJ7
MI / SHAM	-1.9994	2.59E-09	3.91E-07	Q9D7X1
MI / SHAM	-1.96153	7.97E-06	5.52E-05	P05532;P05532-2
MI / SHAM	-1.93124	0.001595	0.00182	Q8R100
MI / SHAM	-1.90022	1.72E-08	1.32E-06	Q9WUK6
MI / SHAM	-1.88796	0.067346	0.030814	Q5RJH6;Q5RJH6-2;Q5RJH6-3
MI / SHAM	-1.87535	2.3E-08	1.41E-06	P23818
MI / SHAM	-1.86746	0.005215	0.004235	O88895
MI / SHAM	-1.80849	0.03938	0.01986	Q5SUF2;Q5SUF2-2;Q5SUF2-3
MI / SHAM	-1.7386	2.53E-06	2.65E-05	O89029
MI / SHAM	-1.71143	6.21E-09	6.3E-07	Q8BGN8-2
MI / SHAM	-1.69902	2.51E-08	1.49E-06	Q9Z140
MI / SHAM	-1.69793	1.92E-09	3.48E-07	G3X9J0
MI / SHAM	-1.68834	7.99E-07	1.26E-05	O35449
MI / SHAM	-1.67828	2.14E-07	5.21E-06	Q91WG7
MI / SHAM	-1.66643	3.34E-10	1.11E-07	Q8VHW2
MI / SHAM	-1.64639	0.000402	0.0007	Q60571
MI / SHAM	-1.63933	9.88E-06	6.42E-05	Q9ERZ4
MI / SHAM	-1.63473	5.86E-08	2.37E-06	P0C7L0
MI / SHAM	-1.58456	1.95E-09	3.48E-07	Q8BRV5
MI / SHAM	-1.57965	0.000902	0.001247	Q0KK59
MI / SHAM	-1.5571	1.56E-07	4.57E-06	P97333
MI / SHAM	-1.52929	0.000179	0.000413	Q69Z38
MI / SHAM	-1.5198	0.006421	0.004945	O35671
MI / SHAM	-1.48324	6.09E-05	0.000206	Q96DY5;Q96DY5-2;Q96DY5-3
MI / SHAM	-1.47884	2.07E-05	0.000103	Q0VE82
MI / SHAM	-1.44083	0.059097	0.02769	Q8K0V4
MI / SHAM	-1.4344	2.7E-07	6.14E-06	Q8VCY8-2
MI / SHAM	-1.41827	0.031874	0.016919	Q8K1S1
MI / SHAM	-1.40965	5.34E-10	1.47E-07	Q03137
MI / SHAM	-1.3862	4.26E-09	5.43E-07	P35436

MI / SHAM	-1.36637	2.07E-07	5.21E-06	Q8R071
MI / SHAM	-1.36215	3.27E-07	6.98E-06	Q9QVP9
MI / SHAM	-1.3578	0.051262	0.024634	O88622;O88622-2
MI / SHAM	-1.34985	0.007116	0.005359	Q6IE26
MI / SHAM	-1.32039	4.03E-09	5.43E-07	P59281;P59281-2
MI / SHAM	-1.32019	0.001874	0.002034	Q3TBW2
MI / SHAM	-1.30831	0.062883	0.02912	Q6P5H2;Q6P5H2-2
MI / SHAM	-1.29673	1E-06	1.43E-05	Q0VGY8
MI / SHAM	-1.29167	0.06215	0.028821	Q3TGW2
MI / SHAM	-1.28519	8.03E-06	5.54E-05	O70507
MI / SHAM	-1.28439	0.001292	0.001571	Q8BLS7
MI / SHAM	-1.26254	0.00654	0.005017	A2ALI5
MI / SHAM	-1.25329	8.28E-07	1.29E-05	Q9ET80
MI / SHAM	-1.25326	7.67E-07	1.23E-05	O54983
MI / SHAM	-1.253	0.005093	0.004159	P54754
MI / SHAM	-1.22184	8.54E-07	1.29E-05	Q7TME0
MI / SHAM	-1.21619	0.00012	0.00032	Q8BKR5
MI / SHAM	-1.20976	0.040824	0.020467	Q99KG3;Q99KG3-2;Q99KG3-3
MI / SHAM	-1.20759	2.39E-10	9.88E-08	O08967
MI / SHAM	-1.20151	0.008748	0.006299	Q3UFT3
MI / SHAM	-1.19891	0.023214	0.013156	Q9QZH6
MI / SHAM	-1.19009	0.055203	0.026224	Q68EF8
MI / SHAM	-1.1759	0.019931	0.011717	Q64737
MI / SHAM	-1.17068	2.39E-06	2.53E-05	Q7TT15;Q7TT15-2
MI / SHAM	-1.15726	0.011723	0.007762	P26048
MI / SHAM	-1.15034	2.71E-07	6.14E-06	Q5U4C3
MI / SHAM	-1.14741	0.009628	0.00676	Q8BJ34;Q8BJ34-3;Q8BJ34-4;Q8BJ34-6
MI / SHAM	-1.13572	7.18E-05	0.000228	Q5QNQ6
MI / SHAM	-1.13421	0.020384	0.01189	Q9D0V7
MI / SHAM	-1.12157	0.105552	0.044454	Q8C3P7
MI / SHAM	-1.11517	0.10172	0.043103	Q9D9M2;Q9D9M2-2
MI / SHAM	-1.10881	4.37E-08	1.9E-06	Q9CPW4
MI / SHAM	-1.10085	2.95E-05	0.000129	Q2PFD7;Q2PFD7-2;Q2PFD7-5
MI / SHAM	-1.09475	2.29E-08	1.41E-06	Q3TBL6
MI / SHAM	-1.09209	3.25E-06	3.1E-05	P97492
MI / SHAM	-1.08605	0.000262	0.000519	Q8K097-2
MI / SHAM	-1.06396	1.9E-07	5.16E-06	Q61097;Q61097-2
MI / SHAM	-1.06304	7.9E-07	1.26E-05	Q3UHB1
MI / SHAM	-1.0628	4.07E-05	0.000156	Q920P5
MI / SHAM	-1.05267	0.000257	0.000512	Q570Y9
MI / SHAM	-1.05252	1.93E-06	2.24E-05	O08914

MI / SHAM	-1.05021	0.042073	0.020992	Q61183;Q61183-3;Q61183-4
MI / SHAM	-1.04799	6.37E-05	0.000213	Q60625
MI / SHAM	-1.04411	1.71E-05	9.15E-05	Q5DTT2;Q5DTT2-3
MI / SHAM	-1.04403	0.000495	0.000814	O54929
MI / SHAM	-1.04263	0.009183	0.00652	Q9JJ69;Q9JJ69-2
MI / SHAM	-1.03896	2.55E-05	0.000117	Q0VBF8
MI / SHAM	-1.03133	0.008774	0.006306	P31001
MI / SHAM	-1.02887	5.36E-06	4.32E-05	Q9Z2W9
MI / SHAM	-1.02628	0.001001	0.00133	Q8JZU0
MI / SHAM	-1.02309	4.69E-05	0.000172	F6SEU4
MI / SHAM	-1.01917	3.55E-06	3.3E-05	P23819-4
MI / SHAM	-1.01365	0.084449	0.036964	Q8BRK9;Q8BRK9-2
MI / SHAM	-1.01213	0.000407	0.000706	Q8K0L9;Q8K0L9-2
MI / SHAM	-1.00752	9.94E-06	6.43E-05	P58871
MI / SHAM	-1.00661	1.67E-06	2.01E-05	Q80YA9
MI / SHAM	-1.00224	1.56E-05	8.51E-05	Q8BIZ1
MI / SHAM	-0.9996	3.53E-07	7.22E-06	P83093
MI / SHAM	-0.99783	1.21E-05	7.21E-05	Q9D415
MI / SHAM	-0.99697	0.026356	0.014573	Q3TTY5
MI / SHAM	-0.99125	0.060511	0.028235	Q07113
MI / SHAM	-0.97684	0.08677	0.037798	Q8BT14;Q8BT14-2;Q8BT14-3
MI / SHAM	-0.95766	0.029226	0.015842	P11679
MI / SHAM	-0.95704	0.000254	0.00051	Q6NSW3;Q6NSW3-2;Q6NSW3-3
MI / SHAM	-0.95426	5.38E-06	4.32E-05	Q8BLR2
MI / SHAM	-0.94837	3.89E-08	1.79E-06	P53995
MI / SHAM	-0.94731	0.00309	0.002904	Q9QUN9
MI / SHAM	-0.94727	0.000209	0.000453	Q64444
MI / SHAM	-0.94614	0.000232	0.000482	Q810B9
MI / SHAM	-0.94159	2.58E-05	0.000118	Q6WQJ1
MI / SHAM	-0.94064	0.00039	0.000692	O08747;O08747-2
MI / SHAM	-0.93935	6.87E-09	6.3E-07	Q4KML4
MI / SHAM	-0.92753	0.026238	0.014517	Q66T02;Q66T02-2
MI / SHAM	-0.92326	2.18E-06	2.36E-05	Q68ED7
MI / SHAM	-0.92246	1.84E-08	1.32E-06	G5E829
MI / SHAM	-0.91959	2.11E-07	5.21E-06	Q62443
MI / SHAM	-0.91817	4.12E-06	3.69E-05	P35438
MI / SHAM	-0.91699	1.87E-05	9.7E-05	Q3TXX4
MI / SHAM	-0.91165	0.000271	0.00053	P27546-4
MI / SHAM	-0.90883	3.38E-07	7E-06	Q60629
MI / SHAM	-0.9083	0.048899	0.023737	Q9JL35
MI / SHAM	-0.90428	3.01E-05	0.000129	Q9WUR9

MI / SHAM	-0.90414	0.025759	0.014289	Q3UHQ6;Q3UHQ6-2
MI / SHAM	-0.89851	0.005724	0.004549	Q3TRR0
MI / SHAM	-0.89687	8.35E-06	5.74E-05	Q9Z1S3
MI / SHAM	-0.89465	0.000363	0.000658	Q9ES46
MI / SHAM	-0.89159	1.02E-05	6.5E-05	A2AV25
MI / SHAM	-0.89045	6.97E-08	2.68E-06	Q8CBF3
MI / SHAM	-0.88257	0.067007	0.030721	Q9CXJ1
MI / SHAM	-0.87993	0.008953	0.006389	Q61210-5
MI / SHAM	-0.87565	0.000399	0.000698	Q8VEL9
MI / SHAM	-0.87441	0.05649	0.026683	Q921R8;Q921R8-2
MI / SHAM	-0.87304	3.12E-05	0.000131	P20444
MI / SHAM	-0.87054	2.88E-06	2.91E-05	P63250
MI / SHAM	-0.8671	0.000144	0.000358	P60761
MI / SHAM	-0.86692	0.049696	0.024	P0DN89;P0DN90;P0DN91
MI / SHAM	-0.85728	0.000189	0.000428	Q91W43
MI / SHAM	-0.85122	0.002228	0.002319	Q8R2R3
MI / SHAM	-0.8485	0.001287	0.001567	Q8R4F1;Q8R4F1-2;Q8R4F1-3
MI / SHAM	-0.84614	9.41E-08	3.32E-06	P70257-1;P70257-2
MI / SHAM	-0.84225	0.001273	0.001559	Q9Z2W8
MI / SHAM	-0.84216	0.028007	0.015286	Q9JJ80
MI / SHAM	-0.84053	2.06E-07	5.21E-06	O55033
MI / SHAM	-0.83282	0.055171	0.026221	P48455
MI / SHAM	-0.83235	6.32E-05	0.000212	Q3UHL1
MI / SHAM	-0.82988	0.000217	0.000462	Q8R409
MI / SHAM	-0.82303	0.008887	0.006357	Q8R104
MI / SHAM	-0.81848	0.000154	0.000377	Q9QWW1;Q9QWW1-2
MI / SHAM	-0.81349	6.28E-06	4.65E-05	P63318
MI / SHAM	-0.80444	2.38E-05	0.000112	O88737
MI / SHAM	-0.80159	2.85E-05	0.000125	Q80TL0
MI / SHAM	-0.80048	0.002508	0.002507	Q9EPQ7
MI / SHAM	-0.79805	4.33E-07	8.14E-06	Q9JL26
MI / SHAM	-0.79802	0.002713	0.002655	Q9DCH4
MI / SHAM	-0.79761	5.67E-06	4.45E-05	P97384
MI / SHAM	-0.79196	0.008841	0.006337	Q8K0F1
MI / SHAM	-0.79173	0.037217	0.019022	O09159
MI / SHAM	-0.79028	0.112003	0.046755	Q9QXW2;Q9QXW2-2
MI / SHAM	-0.78983	0.020246	0.011839	P48381
MI / SHAM	-0.78735	0.034384	0.017985	Q6IFZ6
MI / SHAM	-0.78649	0.001982	0.002131	Q8C7M3-2
MI / SHAM	-0.7864	0.035795	0.018513	Q7TSZ8
MI / SHAM	-0.78354	7.18E-07	1.17E-05	Q8BXR1

MI / SHAM	-0.78126	0.002573	0.002551	Q3UNH4
MI / SHAM	-0.77539	6.26E-06	4.65E-05	Q9QZF2
MI / SHAM	-0.77363	0.000135	0.000346	P11798
MI / SHAM	-0.77147	0.09209	0.0396	Q61112
MI / SHAM	-0.76591	0.001084	0.0014	Q3V0I2
MI / SHAM	-0.76244	4.85E-06	4.1E-05	Q8C0D4
MI / SHAM	-0.76061	0.004318	0.003705	O70404
MI / SHAM	-0.75775	0.021984	0.012587	Q3UQ84
MI / SHAM	-0.75742	0.00042	0.000725	Q6PDG5;Q6PDG5-2
MI / SHAM	-0.75724	2.84E-05	0.000125	Q9QXS6
MI / SHAM	-0.75514	0.000177	0.000412	Q6NXN1;Q6NXN1-2
MI / SHAM	-0.75372	0.011604	0.007708	Q8R555
MI / SHAM	-0.75337	0.020028	0.011756	Q62407
MI / SHAM	-0.7509	4.59E-08	1.95E-06	P84075
MI / SHAM	-0.75064	1.89E-05	9.7E-05	O88444
MI / SHAM	-0.74547	0.000138	0.000349	Q9D8W7
MI / SHAM	-0.7451	0.002841	0.002741	Q3TIV5
MI / SHAM	-0.74461	2.19E-07	5.25E-06	Q80VP9
MI / SHAM	-0.74051	0.000817	0.001159	Q80TE7
MI / SHAM	-0.73974	3.67E-07	7.33E-06	Q6PGE7
MI / SHAM	-0.73801	1.26E-06	1.66E-05	P50429
MI / SHAM	-0.7361	5.09E-09	5.62E-07	Q9DC07
MI / SHAM	-0.7334	0.016861	0.010298	Q8R1B5
MI / SHAM	-0.73062	0.049746	0.024017	P02535;P02535-2;P02535-3
MI / SHAM	-0.73003	5.87E-05	0.000202	Q8BG39
MI / SHAM	-0.72947	0.113162	0.047156	P09470
MI / SHAM	-0.72592	6.03E-06	4.65E-05	Q9Z0J4;Q9Z0J4-5
MI / SHAM	-0.72361	3.06E-05	0.00013	Q8R5H6
MI / SHAM	-0.72302	0.021992	0.012587	Q91XC9
MI / SHAM	-0.72231	0.000159	0.000385	Q80ZF8
MI / SHAM	-0.72089	5.9E-05	0.000202	P55288
MI / SHAM	-0.71692	0.0039	0.003445	P03995
MI / SHAM	-0.71391	5.28E-05	0.000186	B0V2N1;B0V2N1-2;B0V2N1-6
MI / SHAM	-0.71274	7.04E-09	6.3E-07	Q812A2
MI / SHAM	-0.71201	0.000256	0.000511	Q3V3V9-2
MI / SHAM	-0.70986	0.021583	0.012428	Q6P2K6
MI / SHAM	-0.70886	4.66E-05	0.000171	Q9Z2D8;Q9Z2D8-2
MI / SHAM	-0.70879	0.06992	0.031709	O08648
MI / SHAM	-0.707	7.85E-05	0.000241	P54763;P54763-2;P54763-4
MI / SHAM	-0.70695	0.001298	0.001576	Q8BGB5
MI / SHAM	-0.70443	3.26E-08	1.65E-06	Q9WTS5

MI / SHAM	-0.70222	2.99E-05	0.000129	Q9QZB0
MI / SHAM	-0.69874	0.058875	0.027619	P56528
MI / SHAM	-0.69873	0.017832	0.01072	Q91WV0
MI / SHAM	-0.69773	0.036639	0.018796	Q91VL8
MI / SHAM	-0.69633	8.63E-07	1.29E-05	Q8K4I3
MI / SHAM	-0.69599	5.11E-07	9.11E-06	Q60598
MI / SHAM	-0.69363	1.5E-05	8.39E-05	A2CG49
MI / SHAM	-0.6934	0.009472	0.006664	Q8C3Q5-2
MI / SHAM	-0.69241	3.26E-05	0.000134	Q68EF0
MI / SHAM	-0.69185	2.51E-05	0.000115	Q80Y24
MI / SHAM	-0.69149	1.15E-06	1.59E-05	Q9CVB6
MI / SHAM	-0.69127	0.000959	0.001295	Q3UHC7;Q3UHC7-2;Q3UHC7-3
MI / SHAM	-0.6829	0.015725	0.009786	P24788;P24788-2
MI / SHAM	-0.68187	0.001625	0.001838	Q80WT0
MI / SHAM	-0.68149	0.007108	0.005358	Q6DFY8
MI / SHAM	-0.6811	0.001306	0.001584	Q8QZT2
MI / SHAM	-0.6793	0.036807	0.018859	Q9JI18
MI / SHAM	-0.67884	4.08E-05	0.000156	Q9Z1T6
MI / SHAM	-0.67816	0.001789	0.001965	Q9R0I7
MI / SHAM	-0.67697	0.078413	0.034785	Q8BP27;Q8BP27-2
MI / SHAM	-0.67685	0.036547	0.018777	Q7SIG6
MI / SHAM	-0.67648	7.6E-05	0.000235	Q3UHD9
MI / SHAM	-0.676	0.020626	0.012	Q8K1H1
MI / SHAM	-0.67311	0.004834	0.004019	Q7M6Z0
MI / SHAM	-0.67059	2.22E-05	0.000107	B9EJA2;B9EJA2-4;B9EJA2-5
MI / SHAM	-0.66882	0.000162	0.000391	Q3UVX5
MI / SHAM	-0.66873	5.71E-05	0.000198	B1AXH1
MI / SHAM	-0.66788	5.31E-06	4.31E-05	Q8K0S0
MI / SHAM	-0.66565	0.021877	0.012549	Q9JLB9
MI / SHAM	-0.66416	0.000153	0.000375	Q99MD9;Q99MD9-2
MI / SHAM	-0.66264	0.004178	0.003619	P12657
MI / SHAM	-0.66242	3.66E-07	7.33E-06	Q6WVG3
MI / SHAM	-0.66082	0.085471	0.037351	Q3URQ0
MI / SHAM	-0.65854	0.003068	0.002898	Q8BTH8
MI / SHAM	-0.65755	0.031909	0.016931	P63166
MI / SHAM	-0.65501	0.033433	0.01761	Q80U12;Q80U12-2
MI / SHAM	-0.65463	0.001623	0.001837	Q9DB25
MI / SHAM	-0.65321	0.000385	0.000684	D3YZU1
MI / SHAM	-0.65278	0.000141	0.000354	P26040
MI / SHAM	-0.64985	3.69E-05	0.000145	Q01815;Q01815-2
MI / SHAM	-0.64681	0.000596	0.000935	Q8BGC0

MI / SHAM	-0.64551	0.002337	0.002394	Q641P0
MI / SHAM	-0.64539	2.29E-07	5.41E-06	Q61831;Q61831-2
MI / SHAM	-0.64459	0.006095	0.004762	Q32P12;Q32P12-2
MI / SHAM	-0.64306	5E-05	0.000179	Q9D5V6
MI / SHAM	-0.64278	0.002358	0.002411	P59644
MI / SHAM	-0.64222	0.021846	0.012538	Q9EPB4
MI / SHAM	-0.64221	5.71E-07	9.85E-06	P28652
MI / SHAM	-0.64106	0.007364	0.005498	O88428
MI / SHAM	-0.64043	0.001932	0.002086	Q61772;Q61772-2;Q61772-3
MI / SHAM	-0.6398	0.016362	0.01008	P39038
MI / SHAM	-0.63877	0.009212	0.00653	P10637-3;P10637-4
MI / SHAM	-0.63682	0.00167	0.001875	Q9QUS6
MI / SHAM	-0.63599	0.102074	0.043218	Q8R3N6
MI / SHAM	-0.63482	6.05E-06	4.65E-05	Q61220
MI / SHAM	-0.63456	0.002887	0.002776	Q91ZP9
MI / SHAM	-0.63419	0.000213	0.000458	Q9WV32
MI / SHAM	-0.63327	0.021603	0.012435	Q3UHH1;Q3UHH1-2;Q3UHH1-3
MI / SHAM	-0.63169	0.00067	0.001019	Q9CQX0
MI / SHAM	-0.63159	0.000372	0.000669	Q62504;Q62504-2;Q62504-3
MI / SHAM	-0.63156	0.000293	0.000563	A2AP18-2
MI / SHAM	-0.63145	0.002455	0.002476	Q9JKF1
MI / SHAM	-0.62865	4.42E-07	8.22E-06	P70211;P70211-2
MI / SHAM	-0.62814	2.98E-05	0.000129	P08030
MI / SHAM	-0.62811	1.69E-06	2.01E-05	Q64332
MI / SHAM	-0.62788	0.000178	0.000413	P63080
MI / SHAM	-0.62768	0.00019	0.00043	P84086
MI / SHAM	-0.62698	3.37E-07	7E-06	Q8C8N2
MI / SHAM	-0.62669	0.00882	0.006328	Q923T9
MI / SHAM	-0.62619	2.78E-08	1.55E-06	Q8BYM5
MI / SHAM	-0.62586	0.006623	0.005061	Q6P5U7
MI / SHAM	-0.62263	0.080061	0.035402	E9Q1P8
MI / SHAM	-0.62138	3.82E-05	0.000147	Q9R1V4
MI / SHAM	-0.62045	5.17E-06	4.24E-05	P59999
MI / SHAM	-0.62028	0.000772	0.001122	Q61290
MI / SHAM	-0.61937	0.001797	0.001973	Q9WVK4
MI / SHAM	-0.61902	4.17E-05	0.000158	Q80U40;Q80U40-2
MI / SHAM	-0.61786	3.25E-05	0.000134	Q80Y55
MI / SHAM	-0.61654	0.000396	0.000697	Q8BHD7;Q8BHD7-2
MI / SHAM	-0.61516	0.000232	0.000482	Q99NF2-3
MI / SHAM	-0.61482	3.1E-06	3.04E-05	Q80WC7;Q80WC7-2
MI / SHAM	-0.61387	0.002076	0.002204	Q9JJR9

MI / SHAM	-0.613	1.89E-07	5.16E-06	Q4KMM3
MI / SHAM	-0.61216	9.83E-05	0.000279	P15379;P15379-10;P15379-11;P15379-2;P15379-3;P15379-4;P15 8;P15379-9
MI / SHAM	-0.61064	5.99E-05	0.000204	Q9WUB4
MI / SHAM	-0.60959	0.001088	0.0014	Q8BTY8
MI / SHAM	-0.60678	9.75E-05	0.000277	Q8BH64
MI / SHAM	-0.60611	0.068837	0.031385	Q9WV31
MI / SHAM	-0.6052	0.016974	0.010346	P70453;P70453-2
MI / SHAM	-0.60481	0.080317	0.035496	Q9R020;Q9R020-2
MI / SHAM	-0.60069	0.066166	0.030386	Q924H7;Q924H7-2
MI / SHAM	-0.59865	3.88E-07	7.57E-06	Q8BG67;Q8BG67-2
MI / SHAM	-0.59804	1.29E-06	1.67E-05	Q68ED2
MI / SHAM	-0.59772	0.051949	0.024906	Q8VE80
MI / SHAM	-0.5952	0.00024	0.000492	Q9R226
MI / SHAM	-0.59448	5.06E-06	4.19E-05	Q9Z1L5
MI / SHAM	-0.59447	0.112306	0.046858	P59041
MI / SHAM	-0.5938	0.007924	0.005824	Q8K3X4
MI / SHAM	-0.59289	0.04734	0.023105	Q8K377
MI / SHAM	-0.59258	9.6E-05	0.000276	Q9CYG7
MI / SHAM	-0.59234	0.107108	0.045041	Q45VK7;Q45VK7-2
MI / SHAM	-0.59219	0.062561	0.028979	Q9Z1S8
MI / SHAM	-0.59	1.19E-05	7.18E-05	Q8VHR5
MI / SHAM	-0.58813	1.18E-05	7.14E-05	Q8CHG7
MI / SHAM	-0.58701	1.51E-05	8.39E-05	Q68FM6
MI / SHAM	-0.58663	1.57E-07	4.57E-06	Q99JY9
MI / SHAM	-0.58507	1.12E-06	1.56E-05	Q9R257
MI / SHAM	-0.5847	5.97E-07	1.02E-05	Q9R0Q6
MI / SHAM	-0.58446	0.018808	0.011191	P59114
MI / SHAM	-0.5829	4.96E-05	0.000179	P28660
MI / SHAM	-0.58071	0.042646	0.021221	O88811
MI / SHAM	0.58071	0.003353	0.00309	Q8CHT1;Q8CHT1-2
MI / SHAM	0.581065	1.55E-06	1.92E-05	O09111
MI / SHAM	0.581467	0.011302	0.007562	Q6GQW0
MI / SHAM	0.583409	0.021922	0.01257	Q6QWF9
MI / SHAM	0.584186	2E-06	2.27E-05	Q99N87
MI / SHAM	0.584422	0.007935	0.00583	P04370-10
MI / SHAM	0.584745	3.79E-05	0.000147	Q62426
MI / SHAM	0.585457	0.000529	0.000859	Q8R1V4
MI / SHAM	0.588573	0.014514	0.009184	Q8BR63
MI / SHAM	0.589312	4.78E-05	0.000174	P60521
MI / SHAM	0.589901	0.044323	0.021845	Q8K3B1

MI / SHAM	0.590698	0.000324	0.000609	Q9D164;Q9D164-2
MI / SHAM	0.590994	0.000603	0.00094	Q9CR86
MI / SHAM	0.591255	0.007574	0.005624	P70689
MI / SHAM	0.591568	0.007559	0.005619	Q05186
MI / SHAM	0.591697	0.099337	0.042233	Q6PGH0
MI / SHAM	0.592127	1.1E-05	6.86E-05	Q8BYY4
MI / SHAM	0.592907	0.008242	0.006011	P24288
MI / SHAM	0.59487	0.004024	0.003527	Q02257
MI / SHAM	0.594892	0.004927	0.00407	O70496
MI / SHAM	0.596096	8.34E-07	1.29E-05	O35136
MI / SHAM	0.59995	0.00093	0.001275	Q6PIE5
MI / SHAM	0.600463	0.000146	0.000361	Q3UUQ7
MI / SHAM	0.600918	0.001041	0.001369	Q8BGR9
MI / SHAM	0.601292	7.44E-05	0.000232	P56565
MI / SHAM	0.60138	0.006708	0.005107	P21661
MI / SHAM	0.605595	0.005567	0.004449	P13634
MI / SHAM	0.606173	0.002313	0.002379	P21300
MI / SHAM	0.606288	1.22E-06	1.62E-05	A6X919
MI / SHAM	0.60676	0.003618	0.00326	P35762
MI / SHAM	0.610964	0.000759	0.001112	Q99LB7
MI / SHAM	0.611492	0.076278	0.033977	E9Q4N7
MI / SHAM	0.611589	0.010008	0.006941	Q9JMB0
MI / SHAM	0.613204	0.051566	0.024759	Q8CF89
MI / SHAM	0.613654	0.00162	0.001837	O08638;O08638-2
MI / SHAM	0.614134	0.047546	0.023171	P63042
MI / SHAM	0.614234	0.001984	0.002132	O35409
MI / SHAM	0.614981	0.001573	0.001804	Q8R366
MI / SHAM	0.615488	2.81E-08	1.55E-06	Q9CZC8
MI / SHAM	0.615553	0.026689	0.014708	O08600
MI / SHAM	0.616191	0.011949	0.007878	Q7TNG5
MI / SHAM	0.617629	0.03052	0.016383	P70444
MI / SHAM	0.617758	0.000981	0.001317	P10605
MI / SHAM	0.619163	0.000431	0.000735	P54227
MI / SHAM	0.619266	0.006413	0.004942	P10922
MI / SHAM	0.619501	0.07103	0.032103	Q9EQF6
MI / SHAM	0.62131	0.001525	0.001768	Q3UNZ8
MI / SHAM	0.621551	0.010823	0.007345	P85094
MI / SHAM	0.621986	0.027541	0.015082	Q921C1
MI / SHAM	0.622965	0.001172	0.001475	Q99JW2
MI / SHAM	0.624743	1.22E-06	1.62E-05	P31324
MI / SHAM	0.625381	0.001157	0.001465	Q8K448

MI / SHAM	0.625382	0.022284	0.012732	Q9D2P8
MI / SHAM	0.625913	0.007347	0.005492	O88703
MI / SHAM	0.626226	0.035662	0.018455	Q924S8
MI / SHAM	0.627934	0.016864	0.010298	Q6PDG8;Q6PDG8-2
MI / SHAM	0.628009	0.00098	0.001316	Q9WUM5
MI / SHAM	0.628264	0.002374	0.002423	Q9CZP7;Q9CZP7-2;Q9CZP7-3;Q9CZP7-4
MI / SHAM	0.63024	1.93E-05	9.82E-05	Q9WU28
MI / SHAM	0.630466	0.094859	0.040603	Q8R2U6
MI / SHAM	0.631851	0.003175	0.002962	Q9CQZ1
MI / SHAM	0.635474	0.000744	0.001101	Q9WV92-3
MI / SHAM	0.63644	0.00094	0.001284	Q3V0G7;Q3V0G7-2
MI / SHAM	0.637993	0.007081	0.005342	Q8BJS4;Q8BJS4-2;Q8BJS4-3
MI / SHAM	0.64055	4.55E-06	3.93E-05	O08842
MI / SHAM	0.642214	0.038489	0.019502	Q99M31;Q99M31-2
MI / SHAM	0.644028	0.012579	0.00822	P40240
MI / SHAM	0.644059	0.071325	0.03221	Q8JZX9
MI / SHAM	0.64432	0.002298	0.002372	Q8BH86
MI / SHAM	0.64456	0.009355	0.006607	P26049
MI / SHAM	0.647132	0.010452	0.00717	O88952
MI / SHAM	0.648401	0.00071	0.001062	P54797
MI / SHAM	0.650259	0.017433	0.010529	Q921Q3;Q921Q3-2
MI / SHAM	0.652175	0.000779	0.001124	Q80TN5;Q80TN5-2
MI / SHAM	0.655457	2.39E-05	0.000112	Q8K183
MI / SHAM	0.656372	0.108697	0.045593	Q9JIK5
MI / SHAM	0.657119	0.012148	0.007983	P55012
MI / SHAM	0.657174	0.00029	0.000561	Q8BGP6
MI / SHAM	0.658445	0.000524	0.000852	Q91WL8
MI / SHAM	0.662021	0.002802	0.002715	Q9QY42;Q9QY42-2;Q9QY42-3
MI / SHAM	0.663292	0.009675	0.006775	Q3TYD4;Q3TYD4-2
MI / SHAM	0.66348	0.008783	0.006309	Q9R087
MI / SHAM	0.663689	0.000163	0.000392	P11404
MI / SHAM	0.665455	0.005933	0.004677	Q80VQ0
MI / SHAM	0.665904	7.95E-06	5.52E-05	Q9ERD7
MI / SHAM	0.665915	3.9E-06	3.59E-05	P51880
MI / SHAM	0.665984	2.93E-05	0.000129	Q9D1L9
MI / SHAM	0.666583	0.000301	0.000572	Q8QZV4
MI / SHAM	0.66775	0.017299	0.010471	P40237
MI / SHAM	0.668819	9.57E-07	1.38E-05	Q91YS8
MI / SHAM	0.669298	1.01E-05	6.46E-05	P16125
MI / SHAM	0.669669	0.077816	0.034566	O35566
MI / SHAM	0.670627	2.77E-05	0.000123	P52503

MI / SHAM	0.671435	0.001339	0.001611	P48320
MI / SHAM	0.674281	0.003711	0.00332	Q64689
MI / SHAM	0.674654	0.000128	0.000333	Q8CCK0
MI / SHAM	0.675146	0.05844	0.027454	Q8BG89;Q8BG89-2
MI / SHAM	0.675208	2.88E-07	6.36E-06	Q6PDN3;Q6PDN3-3
MI / SHAM	0.676058	1.95E-05	9.87E-05	P70452
MI / SHAM	0.677108	0.000129	0.000333	Q9WUT3
MI / SHAM	0.677307	0.000298	0.000568	Q9JKN6
MI / SHAM	0.677806	0.00071	0.001062	Q8VEB6
MI / SHAM	0.67829	0.04419	0.021799	Q3UFY8
MI / SHAM	0.680478	0.000168	0.000399	Q9EQP2
MI / SHAM	0.680628	0.001476	0.001729	Q61595
MI / SHAM	0.680976	8.32E-05	0.000251	Q62219;Q62219-2;Q62219-3;Q62219-4;Q62219-5;Q62219-7;Q62
MI / SHAM	0.683706	0.013182	0.008497	Q9QYB1
MI / SHAM	0.684428	0.00309	0.002904	Q60673
MI / SHAM	0.685199	0.000187	0.000427	Q99JR5
MI / SHAM	0.68623	0.01261	0.008228	B1AXP6;B1AXP6-2;B1AXP6-3;B1AXP6-4
MI / SHAM	0.687482	0.002588	0.002563	O35218
MI / SHAM	0.68855	0.001556	0.001789	Q61207
MI / SHAM	0.689738	0.000158	0.000384	P21836
MI / SHAM	0.691971	0.002907	0.00279	Q8K2T1
MI / SHAM	0.692351	0.113251	0.047181	Q8CFI0;Q8CFI0-3
MI / SHAM	0.692608	0.000917	0.001259	Q8BGD8
MI / SHAM	0.693021	0.002515	0.00251	O55074;Q7TN79
MI / SHAM	0.693315	0.001	0.00133	Q9CPW0
MI / SHAM	0.696863	0.013088	0.00845	P16330
MI / SHAM	0.703549	1.38E-06	1.74E-05	P32211
MI / SHAM	0.703764	0.079782	0.035298	Q99PI8
MI / SHAM	0.703841	0.028761	0.015636	O35604
MI / SHAM	0.704855	0.073092	0.032891	Q91X56
MI / SHAM	0.706897	0.000596	0.000935	Q9Z2S7;Q9Z2S7-3;Q9Z2S7-4
MI / SHAM	0.707577	0.048948	0.023743	Q5FWI3
MI / SHAM	0.709098	0.000747	0.001104	Q6ZWM4
MI / SHAM	0.71272	0.001989	0.002135	Q8BXA0;Q8BXA0-2
MI / SHAM	0.714841	0.013005	0.008416	Q8C170
MI / SHAM	0.717419	0.000136	0.000347	Q7TNF0
MI / SHAM	0.718425	0.0212	0.012241	Q80YN3
MI / SHAM	0.721097	0.000779	0.001124	Q640M6
MI / SHAM	0.721454	0.054851	0.026101	Q9CQ79
MI / SHAM	0.721958	0.009464	0.00666	O09117-2
MI / SHAM	0.722625	0.020638	0.012	O70305

MI / SHAM	0.722912	0.004168	0.003614	O35393
MI / SHAM	0.723112	0.001622	0.001837	Q00612
MI / SHAM	0.724919	0.008283	0.006032	Q3V0K9
MI / SHAM	0.725791	0.000599	0.000938	Q6PE15;Q6PE15-2
MI / SHAM	0.726863	0.0004	0.000698	Q3UUF8
MI / SHAM	0.726978	2.46E-05	0.000114	E9PV24;E9PV24-2
MI / SHAM	0.732002	0.001193	0.001496	P97450
MI / SHAM	0.733249	0.001767	0.001953	Q8C6B2;Q8C6B2-2
MI / SHAM	0.733382	0.028046	0.015293	P12265
MI / SHAM	0.73425	0.010484	0.007174	Q61282
MI / SHAM	0.736137	0.000136	0.000347	Q9DCC8
MI / SHAM	0.736345	0.004082	0.003554	Q05BC3;Q05BC3-2
MI / SHAM	0.737046	0.090988	0.039177	O35680
MI / SHAM	0.737393	0.000171	0.000404	Q91V64
MI / SHAM	0.738502	0.000807	0.001147	Q8VCI5
MI / SHAM	0.740716	2.01E-07	5.21E-06	Q8K0E8
MI / SHAM	0.741127	0.000159	0.000384	P98200
MI / SHAM	0.742843	0.00255	0.00254	A2APX8;A2APX8-2;A2APX8-3
MI / SHAM	0.743143	4.9E-06	4.1E-05	Q9R069
MI / SHAM	0.744719	0.002013	0.002149	Q9R1V7;Q9R1V7-2
MI / SHAM	0.746546	0.009961	0.006919	Q9CX80
MI / SHAM	0.747202	0.015736	0.009789	Q62433
MI / SHAM	0.747244	0.080621	0.035602	Q6V4S5
MI / SHAM	0.749064	0.00026	0.000517	Q8K4Z0-2
MI / SHAM	0.750323	0.004805	0.004009	Q65CL1
MI / SHAM	0.752156	0.004782	0.003998	Q91X51
MI / SHAM	0.752968	0.001215	0.001512	Q8R127
MI / SHAM	0.755385	3.38E-05	0.000137	P70392
MI / SHAM	0.757983	0.000235	0.000485	Q8K3R3;Q8K3R3-2
MI / SHAM	0.762485	3.82E-05	0.000147	O35633;O35633-2
MI / SHAM	0.762903	0.004647	0.003922	Q8BFZ2;Q8BFZ2-2
MI / SHAM	0.764478	3.29E-07	6.98E-06	Q8R0A5
MI / SHAM	0.76853	0.004008	0.003519	P46656
MI / SHAM	0.769621	0.000299	0.00057	P50153
MI / SHAM	0.774401	0.00786	0.005788	Q6A152
MI / SHAM	0.77454	0.000137	0.000348	Q811I0
MI / SHAM	0.778209	0.016915	0.010317	Q61646
MI / SHAM	0.779967	0.000203	0.000446	P37804
MI / SHAM	0.780554	9.61E-05	0.000276	Q9DB32
MI / SHAM	0.780668	0.004485	0.003801	P0C7M9
MI / SHAM	0.783372	0.026874	0.014805	Q62442;Q62442-2

MI / SHAM	0.784006	0.002597	0.002568	O88343
MI / SHAM	0.784877	0.000112	0.000305	Q9CPZ8
MI / SHAM	0.785781	1.41E-05	8.07E-05	Q9CR21
MI / SHAM	0.786811	0.061662	0.028643	Q9D6I9
MI / SHAM	0.787923	0.021248	0.012264	O55242;O55242-2
MI / SHAM	0.789756	0.002233	0.002322	P97370
MI / SHAM	0.790024	0.000743	0.0011	P60202
MI / SHAM	0.790368	0.011301	0.007562	Q5SYD0
MI / SHAM	0.793759	0.001397	0.001663	Q80XP9;Q80XP9-2
MI / SHAM	0.795333	0.002802	0.002715	P61793;P61793-2
MI / SHAM	0.797026	0.013015	0.008419	Q61469-2
MI / SHAM	0.798951	2.93E-05	0.000129	P07758;Q00896
MI / SHAM	0.801476	0.011218	0.007542	Q8VDQ8
MI / SHAM	0.802926	0.001196	0.001497	Q8CCF0;Q8CCF0-2
MI / SHAM	0.80698	0.000539	0.000867	Q9CXT8
MI / SHAM	0.807523	0.002522	0.002514	Q8C522
MI / SHAM	0.80964	0.000359	0.000653	P97467
MI / SHAM	0.815238	0.000998	0.001329	Q922F4
MI / SHAM	0.815413	2.36E-05	0.000112	Q61923
MI / SHAM	0.815473	0.000107	0.000296	Q00623
MI / SHAM	0.81903	0.012851	0.008336	Q8BGN3
MI / SHAM	0.820014	0.000783	0.001124	Q7TPB0
MI / SHAM	0.822056	0.041657	0.020824	Q9CR30
MI / SHAM	0.823163	0.044086	0.021754	P28481-4
MI / SHAM	0.826625	0.002965	0.002835	Q3U7R1
MI / SHAM	0.829374	0.010315	0.007097	Q80XD1;Q80XD1-2;Q80XD1-3
MI / SHAM	0.833407	4.66E-07	8.57E-06	O54879
MI / SHAM	0.836881	0.004821	0.004016	Q60803
MI / SHAM	0.838285	4.76E-11	3.94E-08	Q04899
MI / SHAM	0.839526	0.00035	0.000643	Q9Z0L0
MI / SHAM	0.840594	8.88E-05	0.000264	Q6P069;Q6P069-2
MI / SHAM	0.840838	7.24E-06	5.15E-05	P29391
MI / SHAM	0.841913	0.001315	0.001592	P17095
MI / SHAM	0.84219	0.07996	0.035367	P63054
MI / SHAM	0.844441	4.52E-05	0.000167	P06837
MI / SHAM	0.846892	0.000297	0.000568	P02469
MI / SHAM	0.84957	0.003604	0.003252	P28650
MI / SHAM	0.850841	0.000468	0.000783	Q8C996
MI / SHAM	0.85154	0.006168	0.004795	Q61330
MI / SHAM	0.851581	0.00015	0.000369	Q5NCI0;Q5NCI0-2
MI / SHAM	0.851929	0.00076	0.001113	O55091

MI / SHAM	0.853161	0.036497	0.018767	Q9D486;Q9D486-2
MI / SHAM	0.853257	0.018232	0.010912	P49025;P49025-3
MI / SHAM	0.854968	0.000851	0.001195	P53668
MI / SHAM	0.855305	0.009666	0.006774	Q80Y50;Q80Y50-2;Q80Y50-4
MI / SHAM	0.856347	0.017101	0.010405	Q80WM4
MI / SHAM	0.85725	1.29E-05	7.6E-05	Q9JIM1;Q9JIM1-2
MI / SHAM	0.85832	0.011481	0.007648	Q922J6
MI / SHAM	0.858576	0.001062	0.001386	Q99L13
MI / SHAM	0.859815	0.000267	0.000527	Q8BUE4;Q8BUE4-2
MI / SHAM	0.860852	0.010948	0.007406	Q08091;Q08091-2
MI / SHAM	0.86248	0.058864	0.027619	Q8CIZ8
MI / SHAM	0.863931	0.002808	0.002716	Q8BUM6
MI / SHAM	0.866612	0.118994	0.049153	Q9DC04;Q9DC04-1;Q9DC04-4
MI / SHAM	0.866697	0.000198	0.000441	Q9CWD3;Q9CWD3-2
MI / SHAM	0.867503	0.033646	0.017694	Q7TPM1
MI / SHAM	0.869777	0.000533	0.000863	Q9R1B9
MI / SHAM	0.870027	0.000686	0.001037	Q9D7J4
MI / SHAM	0.876279	0.000769	0.001119	Q9QXC1
MI / SHAM	0.877114	0.031269	0.016688	Q9Z1M0
MI / SHAM	0.879342	0.001593	0.001818	O35668
MI / SHAM	0.885593	0.002942	0.002818	Q9JHR9;Q9JHR9-2
MI / SHAM	0.887264	0.012002	0.007897	Q8R3P0
MI / SHAM	0.889084	0.000791	0.001133	Q8CFA2
MI / SHAM	0.889296	0.000422	0.000726	P22599
MI / SHAM	0.889911	2.38E-05	0.000112	D3YVE8
MI / SHAM	0.890789	0.005851	0.004629	Q9QXX4
MI / SHAM	0.893409	0.002449	0.002473	Q78TU8
MI / SHAM	0.89587	0.002998	0.002856	Q8VHP7
MI / SHAM	0.896519	0.027311	0.014989	Q8R2Q4;Q8R2Q4-2;Q8R2Q4-3
MI / SHAM	0.898056	0.067668	0.030921	Q3V3N7
MI / SHAM	0.899178	1.52E-05	8.42E-05	Q91YQ3
MI / SHAM	0.900684	0.008785	0.006309	Q91VC7
MI / SHAM	0.901772	0.011022	0.007435	Q61885
MI / SHAM	0.905781	0.009931	0.00691	Q8BZF2
MI / SHAM	0.906791	0.009635	0.00676	P46660
MI / SHAM	0.909507	0.012462	0.008157	P61953
MI / SHAM	0.915141	0.000799	0.001138	Q8K0S5
MI / SHAM	0.919575	0.004913	0.004064	Q8BQU6
MI / SHAM	0.919684	0.000249	0.000504	Q8BPU7
MI / SHAM	0.919863	0.00015	0.000369	Q8CCX5
MI / SHAM	0.928306	0.01056	0.007205	Q91WF7

MI / SHAM	0.928499	0.001781	0.00196	P61148
MI / SHAM	0.930382	0.000993	0.001326	P14231
MI / SHAM	0.932138	0.00226	0.002345	P63141
MI / SHAM	0.937757	0.003938	0.003466	P35505
MI / SHAM	0.93822	0.017823	0.01072	A2A6T1
MI / SHAM	0.939343	0.000903	0.001248	P62715
MI / SHAM	0.940098	2.29E-05	0.000109	Q8CD19
MI / SHAM	0.941052	0.001166	0.001471	Q9R0N8;Q9R0N8-2
MI / SHAM	0.944441	0.000233	0.000483	Q80U57
MI / SHAM	0.946706	3.77E-05	0.000146	Q8BZ98
MI / SHAM	0.948112	0.07629	0.033977	Q8C033
MI / SHAM	0.950522	0.010743	0.007297	Q3UL36
MI / SHAM	0.951171	0.000232	0.000482	O35488
MI / SHAM	0.953881	0.007758	0.005723	P97382
MI / SHAM	0.958257	1.34E-05	7.78E-05	P28665
MI / SHAM	0.959636	0.000783	0.001124	Q8BRU6
MI / SHAM	0.961814	3.82E-07	7.53E-06	P42232
MI / SHAM	0.971861	0.008339	0.006059	Q8K298
MI / SHAM	0.973966	0.039294	0.019838	Q91YT8
MI / SHAM	0.975473	0.051703	0.02481	Q8BZQ7
MI / SHAM	0.978964	0.005128	0.004182	Q8C4Y3-3
MI / SHAM	0.979128	0.056297	0.026621	Q8C3J5
MI / SHAM	0.982851	0.006187	0.004805	Q9D154
MI / SHAM	0.983518	0.006059	0.004746	P15388-3
MI / SHAM	0.987558	0.003556	0.003225	Q9CPU2
MI / SHAM	0.989247	0.017965	0.010783	Q8BKY8
MI / SHAM	0.992541	0.025169	0.014019	Q4VBD2
MI / SHAM	0.993649	0.107847	0.0453	P41778;P41778-2
MI / SHAM	1.000249	0.046929	0.022938	Q9JKK0
MI / SHAM	1.002751	0.003185	0.002969	Q9D387
MI / SHAM	1.012868	0.001643	0.00185	Q9WU63
MI / SHAM	1.015054	0.001822	0.001996	P08553
MI / SHAM	1.016805	0.020368	0.011885	Q3TVA9;Q3TVA9-2
MI / SHAM	1.016986	0.005324	0.004297	Q8BL06
MI / SHAM	1.018453	0.000116	0.000313	Q6ZQB6;Q6ZQB6-2;Q6ZQB6-3
MI / SHAM	1.019786	0.094903	0.040609	B9EJI9
MI / SHAM	1.021274	0.001771	0.001955	Q9WV95
MI / SHAM	1.025369	0.00106	0.001385	Q9JHG2
MI / SHAM	1.028398	8.79E-06	6.02E-05	P56382
MI / SHAM	1.029369	0.001549	0.001784	Q9WTQ5
MI / SHAM	1.03906	0.050899	0.024488	Q9Z126

MI / SHAM	1.040033	0.004853	0.004032	Q8K406
MI / SHAM	1.042354	0.036503	0.018767	Q80TQ5;Q80TQ5-2;Q80TQ5-3
MI / SHAM	1.049512	0.000182	0.000418	P58069
MI / SHAM	1.049529	0.02101	0.01216	P61458
MI / SHAM	1.051644	0.009999	0.006937	O88492;O88492-2
MI / SHAM	1.056496	0.001254	0.001549	Q6P5H6;Q6P5H6-2;Q6P5H6-3;Q6P5H6-4
MI / SHAM	1.057686	0.000958	0.001295	P19536
MI / SHAM	1.058245	0.003188	0.002969	Q6A026
MI / SHAM	1.063636	0.000355	0.000647	P12787
MI / SHAM	1.066351	0.066131	0.030382	Q9R0X5;Q9R0X5-2
MI / SHAM	1.070704	0.007111	0.005358	P20917
MI / SHAM	1.077066	2.66E-05	0.00012	Q8JZW4
MI / SHAM	1.078623	0.003652	0.003281	Q9DB72
MI / SHAM	1.085822	0.043717	0.021617	B1AWL2
MI / SHAM	1.086724	0.029353	0.015901	Q6P8J2
MI / SHAM	1.091447	0.039253	0.019824	Q8BGV8
MI / SHAM	1.091513	0.000988	0.00132	Q9JM63
MI / SHAM	1.09482	0.004753	0.003982	Q9JL62
MI / SHAM	1.096294	1.21E-07	4.08E-06	Q8VCM7
MI / SHAM	1.102833	0.000399	0.000698	P61922
MI / SHAM	1.10414	0.001328	0.001601	Q64436
MI / SHAM	1.104246	0.000182	0.000418	Q9CRB6
MI / SHAM	1.108407	0.034865	0.018161	Q91V24
MI / SHAM	1.111257	0.007148	0.005376	Q9DC11
MI / SHAM	1.111486	0.117549	0.048646	Q9D8Z6
MI / SHAM	1.113284	0.016633	0.010201	P56671
MI / SHAM	1.120916	1.88E-05	9.7E-05	Q9JLC4;Q9JLC4-2;Q9JLC4-3;Q9JLC4-4
MI / SHAM	1.125841	0.000777	0.001124	Q9JJF0
MI / SHAM	1.137629	0.002618	0.002584	Q7TN98
MI / SHAM	1.152759	0.080935	0.035712	Q7TPV2;Q7TPV2-2
MI / SHAM	1.153585	0.000842	0.001187	Q8HW98
MI / SHAM	1.15423	0.003538	0.003214	P50114
MI / SHAM	1.159757	0.000684	0.001034	P59823
MI / SHAM	1.163498	0.002092	0.002212	Q9Z0Z4;Q9Z0Z4-2
MI / SHAM	1.170867	0.005134	0.004184	Q8R4V2
MI / SHAM	1.175868	0.004249	0.003661	P22933
MI / SHAM	1.177585	0.004738	0.003978	Q9WV92-7
MI / SHAM	1.179991	0.001046	0.001375	Q9JJ59
MI / SHAM	1.182965	0.003573	0.003237	Q9WVT6
MI / SHAM	1.188374	0.016344	0.010073	Q9R171
MI / SHAM	1.189074	0.002133	0.002244	Q02357-6

MI / SHAM	1.189861	0.000271	0.00053	P51125;P51125-2;P51125-3;P51125-6
MI / SHAM	1.198626	0.001776	0.001958	Q99L20
MI / SHAM	1.201795	0.003362	0.003094	P08551
MI / SHAM	1.204412	0.006581	0.005041	Q8BXQ2
MI / SHAM	1.211598	0.002939	0.002818	P51910
MI / SHAM	1.214758	0.001589	0.001814	P24549
MI / SHAM	1.218344	0.049552	0.023973	Q8K2P7
MI / SHAM	1.219911	0.000192	0.000433	Q9DCB4;Q9DCB4-3;Q9DCB4-4;Q9DCB4-5;Q9DCB4-9
MI / SHAM	1.220996	0.049345	0.023893	Q8C0Z1
MI / SHAM	1.231527	6.72E-06	4.86E-05	P0DP26;P0DP27;P0DP28
MI / SHAM	1.23356	2.97E-05	0.000129	P07759
MI / SHAM	1.254598	0.006004	0.004713	P84096
MI / SHAM	1.257812	0.000302	0.000572	Q61205
MI / SHAM	1.264936	4.93E-06	4.1E-05	P54830
MI / SHAM	1.286154	0.000358	0.000652	Q6PHZ2-5
MI / SHAM	1.294436	0.042206	0.021033	Q8BUB4
MI / SHAM	1.302263	3.48E-06	3.26E-05	Q91X72
MI / SHAM	1.304691	0.006674	0.005088	Q9EPQ8;Q9EPQ8-2
MI / SHAM	1.308804	3.24E-05	0.000134	Q62465
MI / SHAM	1.309352	1.6E-05	8.69E-05	A6X935
MI / SHAM	1.31411	0.012169	0.00799	Q6P9J5
MI / SHAM	1.319106	0.046177	0.022644	Q8BLK9;Q8BLK9-2;Q8BLK9-3
MI / SHAM	1.3235	0.076841	0.034179	P70365;P70365-2;P70365-3;P70365-4
MI / SHAM	1.324431	0.000112	0.000307	Q69ZS6
MI / SHAM	1.337488	3.96E-06	3.6E-05	Q8CJF9
MI / SHAM	1.337782	0.067078	0.030745	Q8K2I4
MI / SHAM	1.35197	0.03179	0.0169	Q8VCE1
MI / SHAM	1.356877	0.02314	0.013123	Q6P9Z1
MI / SHAM	1.383182	0.000433	0.000737	Q6PHS9;Q6PHS9-2;Q6PHS9-3;Q6PHS9-4;Q6PHS9-5;Q6PHS9-6
MI / SHAM	1.385926	0.004709	0.003963	P97799
MI / SHAM	1.388416	0.076369	0.033988	Q8BIL5
MI / SHAM	1.395513	2.11E-05	0.000104	Q01065
MI / SHAM	1.426229	0.000558	0.000891	Q8BWU8
MI / SHAM	1.466055	0.000208	0.000452	Q03517
MI / SHAM	1.474344	0.000123	0.000324	Q61503
MI / SHAM	1.484809	0.000446	0.000756	O88533
MI / SHAM	1.502039	0.00163	0.00184	O08599-2
MI / SHAM	1.505753	0.102153	0.043232	Q8C650;Q8C650-2
MI / SHAM	1.515357	0.000103	0.000289	O54834;O54834-2;O54834-3;O54834-4
MI / SHAM	1.532813	0.000237	0.000487	P84309
MI / SHAM	1.537253	0.002829	0.002732	Q9JLM9

MI / SHAM	1.545504	0.001025	0.001355	Q8K3E5
MI / SHAM	1.597652	0.001053	0.001378	P28571-1
MI / SHAM	1.59964	9.09E-05	0.000266	A2ARS0
MI / SHAM	1.614875	0.027678	0.015147	Q6VVW5;Q6VVW5-2
MI / SHAM	1.622276	0.002092	0.002212	Q9ESM3
MI / SHAM	1.642531	0.002018	0.002151	P54116
MI / SHAM	1.644791	1.84E-05	9.65E-05	Q61327
MI / SHAM	1.68139	0.020268	0.011848	O35143
MI / SHAM	1.684847	0.000421	0.000726	Q7TQA1;Q7TQA1-5
MI / SHAM	1.700488	0.001729	0.001923	P32848
MI / SHAM	1.728134	0.002005	0.002144	P28230
MI / SHAM	1.734645	0.003096	0.002908	P19246
MI / SHAM	1.735386	5.98E-15	9.91E-12	Q6W8Q3
MI / SHAM	1.781581	0.000194	0.000436	Q8CGK7
MI / SHAM	1.794242	0.000551	0.000883	P31650
MI / SHAM	1.811821	0.003315	0.003059	Q0PMG2
MI / SHAM	1.823681	9E-05	0.000265	Q9QXV0
MI / SHAM	1.823931	0.000592	0.000933	P56389
MI / SHAM	1.824921	0.035114	0.018257	Q8R3L5-2
MI / SHAM	1.838173	0.003242	0.003005	Q63959
MI / SHAM	1.853591	0.004794	0.004004	Q8R3Q0
MI / SHAM	1.854976	0.001069	0.001389	Q9Z0F7
MI / SHAM	1.866554	0.00015	0.000369	Q61704
MI / SHAM	1.868958	4.86E-08	2.01E-06	Q50H33
MI / SHAM	1.919936	0.047082	0.022999	Q6P5G6
MI / SHAM	1.943197	0.003543	0.003216	P46097
MI / SHAM	2.011333	3.7E-05	0.000145	Q80TB8
MI / SHAM	2.014951	1.48E-05	8.34E-05	Q08331
MI / SHAM	2.027975	0.000164	0.000393	Q9Z123
MI / SHAM	2.037068	6.03E-05	0.000205	Q8CA95;Q8CA95-2;Q8CA95-3
MI / SHAM	2.03839	1.55E-05	8.5E-05	Q8BFZ3
MI / SHAM	2.081458	4.9E-06	4.1E-05	Q9QUG9
MI / SHAM	2.1224	0.007918	0.005823	Q761V0;Q761V0-2
MI / SHAM	2.288958	2.69E-05	0.00012	Q60829
MI / SHAM	2.349128	0.062422	0.028931	O08644
MI / SHAM	2.54647	0.004983	0.004093	Q9JJA9
MI / SHAM	2.575774	0.046554	0.022795	P63084
MI / SHAM	2.668204	0.00011	0.000302	Q00898
MI / SHAM	2.79329	0.000258	0.000513	Q80TT2
MI / SHAM	2.853638	0.009241	0.006546	Q91X58
MI / SHAM	2.87159	3.11E-05	0.000131	Q7M729

MI / SHAM	3.474303	0.000313	0.000591	A3KGB4
MI / SHAM	3.542859	0.000682	0.001033	Q9D4V7;Q9D4V7-2
MI / SHAM	4.127165	2.66E-05	0.00012	P24529
MI / SHAM	5.326836	0.002185	0.002286	Q8BG51-3
