# Supplementary material for: Heart failure-induced cognitive dysfunction is mediated by intracellular Ca2+ leak through ryanodine receptor type 2
Source: Nat Neurosci. 2023 Jul 10;26(8):1365–78. doi: 10.1038/s41593-023-01377-6 (PMC10400432; doi:10.1038/s41593-023-01377-6)

Figure 7C

	SNAP25/GAPDH	VAMP8/GAPDH	SYT2/GAPDH	SYT13/GAPDH	CPLX3/GAPDH
C1	0.8	2.3	1	3	
C2	1.2	2	1.1	3.3	2.2
C3	1	2.7	0.9	3.4	2.3
C4	1.1	2.3	1.2	3.6	2.4
HF1	2.5	0.8	3.3	0.8	2.6
HF2	2.8	0.6	3.6	0.6	0.8
HF3	2.4	0.7	3	1	0.9
HF4	2.9	0.4	3.1	0.4	1
HF5	2.6	0.4	2.6	0.5	0.8
HF6	2.7	0.2	3.1	0.1	1
HF7	2.8	0.4	2	0.4	1.1
HF8	3	0.5	1.6	0.1	1.2
HF9	2.8	0.6	1.1	0.7	1

Figure 7E

	Snap25	VAMP8	SYT2	SYT13	CPLX3
SHAM	1	3.7	0.8	3.5	3.3
SHAM	1.1	3.9	0.9	3.8	3.5
SHAM	1	3.5	0.5	3.5	3.5
SHAM	1.2	4	0.8	2.8	3.7
SHAM	0.8	3.2	1.3	2.4	4.2
SHAM	0.7	4.4	0.9	4	4
MI	4	0.2	4	1	0.6
MI	3.9	0.3	4.2	0.8	0.8
MI	3.5	0.9	2.5	0.3	1
MI	4	1.2	3.9	0.5	0.5
MI	3.7	0.7	3	1	1
MI	4.6	0.5	2.8	0.75	1.2
MI+ARM036	3.8	0.5	4.4	0.7	0.9
MI+ARM036	3.7	0.4	4.4	0.6	0.7
MI+ARM036	3.2	1.5	2	1	0.95
MI+ARM036	4	2.2	2.6	1.2	1.2
MI+ARM036	3.9	1.7	3	0.85	0.85
MI+ARM036	4.2	2	3.5	0.65	1.15
MI+S107	1.2	3.8	1	3.7	3.6
MI+S107	1	3.6	0.8	3.8	3.5
MI+S107	1.5	3.2	0.5	3.9	3.9
MI+S107	2.3	3.9	0.2	3.2	3.2
MI+S107	1.9	3.6	1	2.9	4
MI+S107	2	4.5	1.15	2.75	3.7
MI+prop	1.5	2.6	0.9	3.5	3.5
MI+prop	1.7	2.2	1	3.6	3.6
MI+prop	1.9	2.3	0.8	3.2	3.3
MI+prop	1.4	2.8	1.1	2.5	3.8
MI+SD-208	1.7	3.3	0.8	3.7	3.6
MI+SD-208	1.5	3.5	0.7	3.5	3.8
MI+SD-208	1.4	3.1	0.6	3.3	3.8
MI+SD-208	1.7	3.6	0.9	3.7	3.6

Figure 7B-D

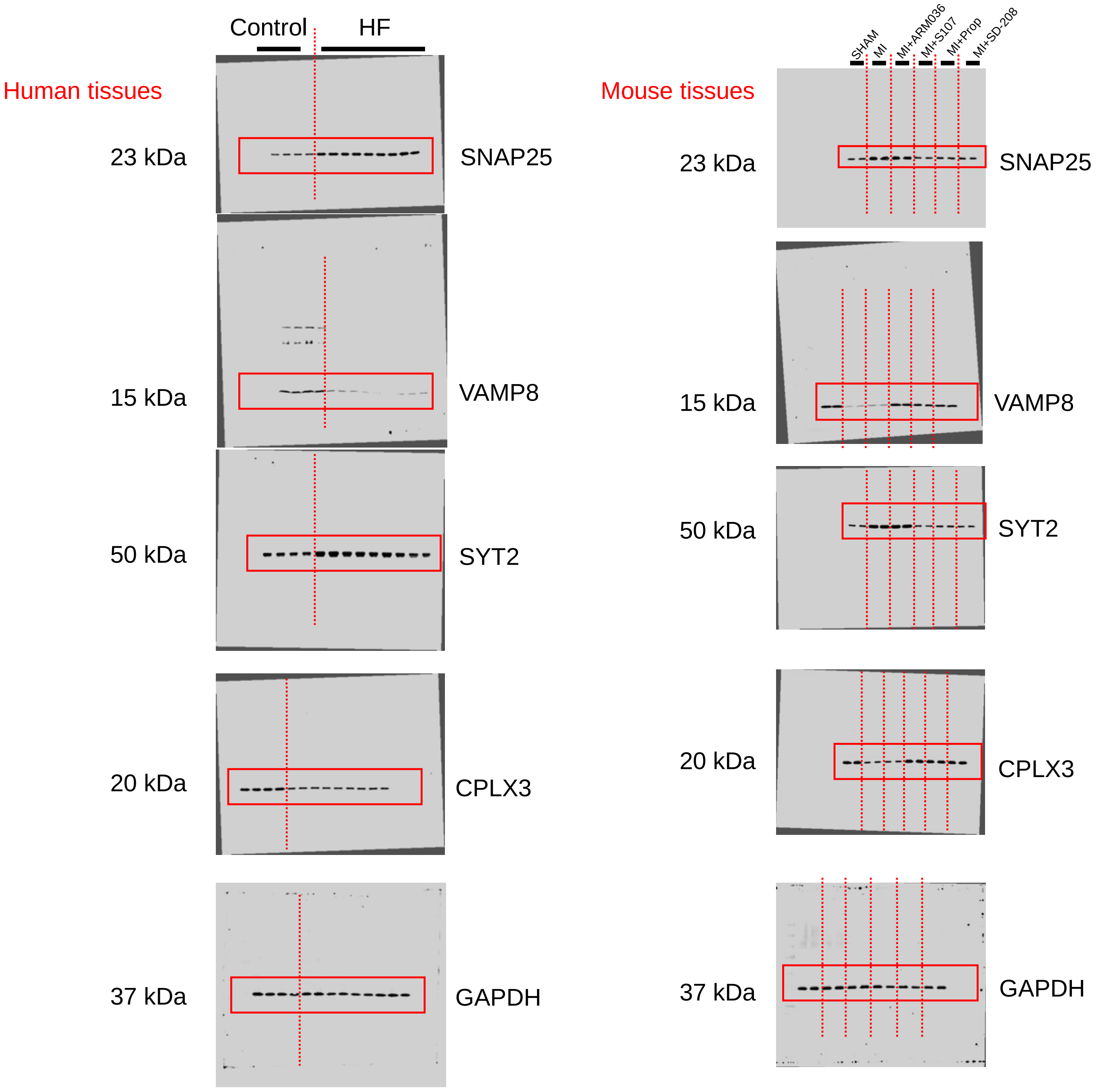

Control
HF
Human tissues
23 kDa
SNAP25
15 kDa
VAMP8
50 kDa
SYT2
20 kDa
CPLX3
37 kDa
GAPDH
SHAM
MI
MI+ARM036
MI+S107
MI+Prop
MI+SD-208
Mouse tissues
23 kDa
SNAP25
15 kDa
VAMP8
50 kDa
SYT2
20 kDa
CPLX3
37 kDa
GAPDH

Mouse tissues

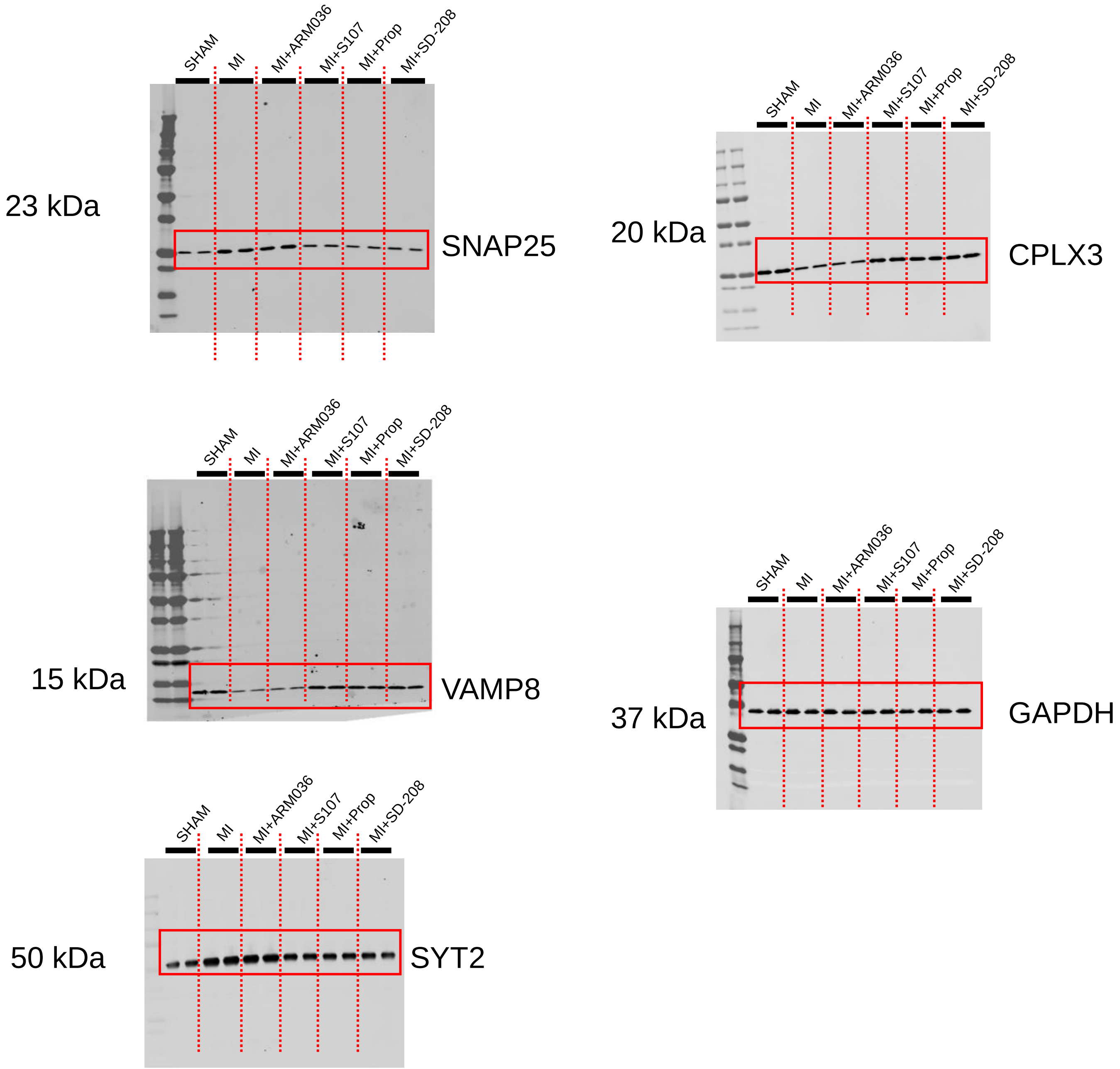

Mice tissues

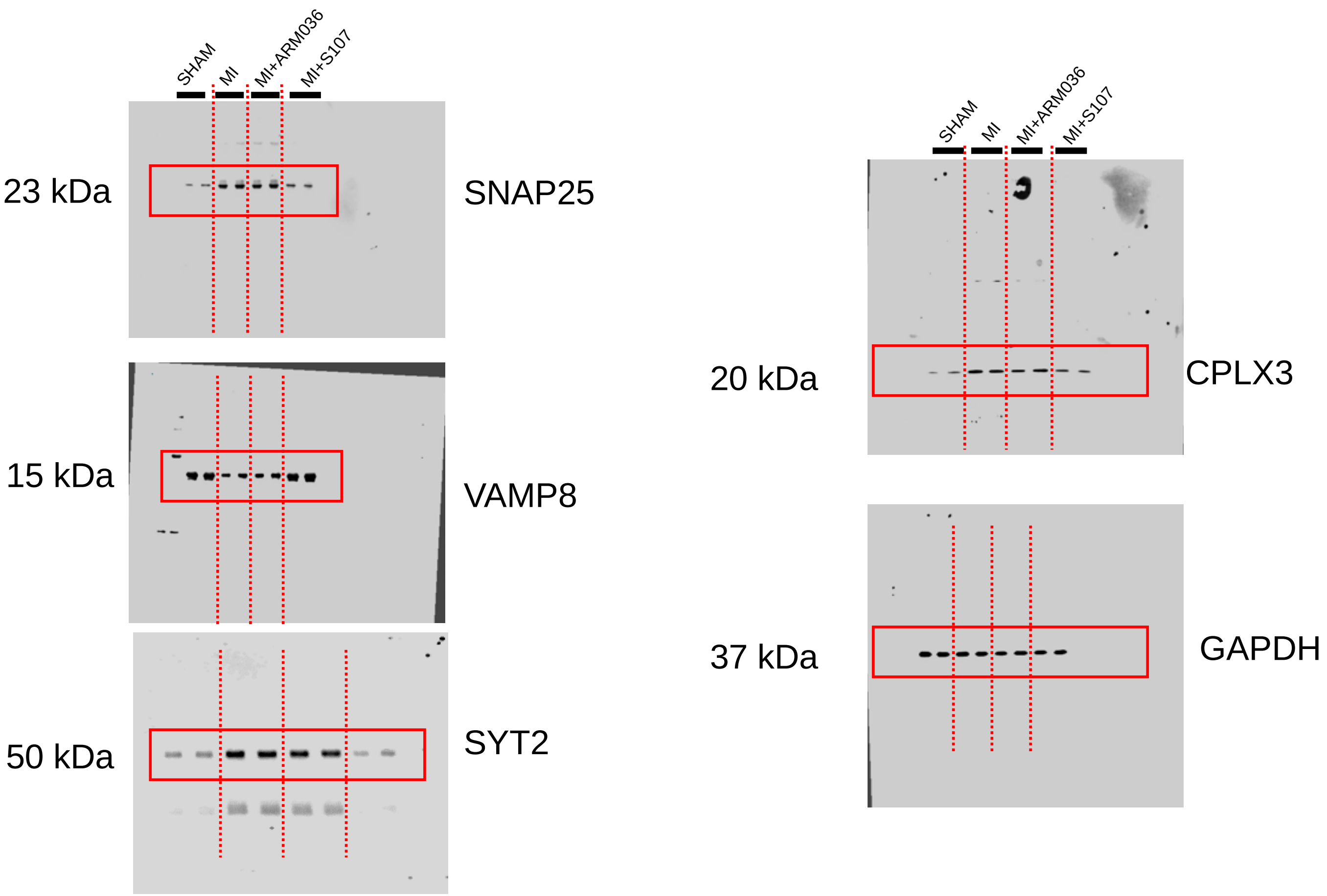

Supplement: Source Data Fig. 7 — Unprocessed western blots and statistical source data. [file 41593_2023_1377_MOESM10_ESM.pdf]
