# Supplementary material for: Heart failure-induced cognitive dysfunction is mediated by intracellular Ca2+ leak through ryanodine receptor type 2
Source: Nat Neurosci. 2023 Jul 10;26(8):1365–78. doi: 10.1038/s41593-023-01377-6 (PMC10400432; doi:10.1038/s41593-023-01377-6)
Supplement: Source Data Extended Data Fig. 1 — Statistical source data. [file 41593_2023_1377_MOESM11_ESM.pdf]

ED_Fig1A

C57BL	C57BL	S2808A Sham	S2808A Sham	S2808A+MI	S2808A+MI	S2808D	S2808D	S2808D+S107	S2808D+S107
0.2663571	0.583514	0.0509722	0.0939589	0.0425113	0.2017683	0.0550997	0.1153798	0.4520813	0.589928
0.1295127	0.3442868	0.1135857	0.2050613	0.2402674	0.2217471	0.193634	0.1701229	0.1679969	0.3178124
0.1452355	0.1570354	0.1092623	0.8198362	0.0295127	0.3442868	0.1985658	0.3815335	0.5933434	0.550254
0.218769	0.4554757	0.1455483	0.204255	0.0452355	0.2570354	0.219347	0.1349306	0.1218448	0.2784999
0.1842884	0.5438717	0.1984317	0.0887316	0.218769	0.2554757	0.094358	0.2351609	0.1895884	0.2972038
0.3053884	0.2825994	0.1880735	1.079482	0.2519997	0.3861081	0.2899527	0.5040107	0.2525224	0.3590034
0.1697094	0.2378791	0.107965	0.0997068	0.0697094	0.2378791	0.1870219	0.2958963	0.2116601	0.1232449
0.2899527	0.5040107	0.188668	0.3366998			0.1370097	0.3074744	0.1434379	0.1707317
0.1870219	0.2958963					0.1313639	0.1602424		
0.1370097	0.3074744					0.2338909	0.1972861		
0.1313639	0.4602424					0.1267606	0.0353753		
0.2338909	0.3972861					0.2916188	0.1790136		
0.1267606	0.4353753					0.283514	0.2663571		
0.2916188	0.3790136								

ED_Fig1B

C57sham	S2808A Sham	S2808A MI	S2808D	S2808D+S107
0.052632	0.058268	0.042857	1.090909	0.291863
0.401786	0.143717	0.333333	0.588235	0.626499
0.476033	0.140196	0.166667	1.111111	0.327791
0.054688	0.433023	0.05	0.555556	0.238527
0	0.212566	0.333333	1	0.295398
0.44	0.09173	0.090909	0.538462	0.269117
0.3125	0.156227	0.25	0.909091	0.228489
0.057632	0.153949		0.666667	
0.201786			0.590654	
0.231007			0.386625	
0.048688			0.633868	
0.177777			0.715031	
0.183662			0.622917	
0.3125				

ED_Fig1C

C57sham	S2808A Sham	S2808A MI	S2808D	S2808D+S107
46.59091	42.2895	46.59091	-7.604563	15.60694
44.74849	38.1106	34.74849	19.20398	17
52.0197	59.72696	52.0197	-6.91079	9.915014
49.43925	48.10997	49.43925	11.15974	19.48052
66	41.62043	50	19.88858	28.60636
57.77778	40.22333	27.777778	-14.74849	28.01653
65.51724	51.978261	44	6.08365	19.68668
56.99077	42.36227	46.81458	12.96703	7
44.27481			-6.85393	
33.5689			-7.802776	
38.5689			0	
39.8566			4.066161	
			-8.01917	

ED_Fig1D

	C57sham			S2808A MI			S2808D			S2808D+S107			S2808A Sham		
	Mean	SD	N	Mean	SD	N	Mean	SD	N	Mean	SD	N	Mean	SD	N
**Day1**	59.15972	0.6046838	14	57.84762	2	7	57.32308	1.407061	12	58	1.875	8	56	3	10
**Day2**	42.57777	5.308613	14	46.99047	4	7	58.01026	3.11801	12	57	2.293184	8	44	5	10
**Day3**	34.40973	5.153997	14	36.575	3	7	50.72821	2.370323	12	49	5.042468	8	34	4	10
**Day4**	25.72361	4.116466	14	27.42857	3	7	45.63334	3.900673	12	39	4.249664	8	26	4	10

ED_Fig1E

C57sham	S2808A Sham	S2808A MI	S2808D	S2808D+S107
29.6	36.08	19.34	10.98	10.47
33.8	18.86	22.6	13.16	0
21.8	31.64	24.72	11.88	13.47
31.8	16.66	32.09	15.31	12.11
25.2	18.17	18.48	16.28	13.25
27.4	22.1	19.78	19.26	13.12
22	19.35	21.46	8.19	11.68
20.8	21.1		10.1	20.46
29.4	16.12		11.21	
20.6	20.96		11.74	
17.5			6.11	
16.4			0	
16.2				
18.2				

ED_Fig1F

C57sham	S2808A Sham	S2808A MI	S2808D	S2808D+S107
5	5	5	2	2
5	6	5	3	0
5	3	4	3	4
4	3	2	2	2
4	4	6	3	3
4	6	5	2	1
3	6	5	1	1
7	5		2	2
3			1	
3			3	
7			0	
4			2	
4				
3				
