# Supplementary material for: Heart failure-induced cognitive dysfunction is mediated by intracellular Ca2+ leak through ryanodine receptor type 2
Source: Nat Neurosci. 2023 Jul 10;26(8):1365–78. doi: 10.1038/s41593-023-01377-6 (PMC10400432; doi:10.1038/s41593-023-01377-6)
Supplement: Source Data Extended Data Fig. 2 — Statistical source data. [file 41593_2023_1377_MOESM12_ESM.pdf]

ED_Fig2A

WT	Wt	44D	44D
0.01755	0.366941	0.01983	0.107284
0.016949	0.310817	0.06692	0.276958
0.015289	0.229992	0.047852	0.349224
0.121915	0.183113	0.026165	0.366017
0.080108	0.112622	0.121915	0.258311
0.065972	0.215449	0.080108	0.112622
0.188197	0.248439	0.065972	0.115449
0.106263	0.392758	0.188197	0.248439
0.052508	0.241046	0.106263	0.392758
0.07389	0.260802	0.052508	0.141046
		0.07388	0.24608
		0.129873	0.322849
		0.035931	0.537148
		0.109194	0.204255
		0.033176	0.099035
		0.06993	0.092366
		0.106263	0.392758
		0.052508	0.141046
		0.007388	0.460802
		0.129873	0.322849
		0.035931	0.253715

ED_Fig2B

WT	44D
0.166667	0.444444
0.3	0.461538
0.4	0.428571
0.428571	0.428571
0.290909	0.222222
0.242736	0.111111
0.192301	0.163636
0.336364	0.454545
0.444557	0.375
0.364478	0.266742
	0.25
	0.142857
	0.333333
	0.363636
	0.3
	0.3
	0.372512
	0.177778
	0.260339
	0.142853
	0.334473

ED_Fig2C

WT	44D
60	13.70244
11.11111	43.46154
33.33333	25.46342
33.33333	12.64642
33.33333	40.85074
20	43.91304
24.48774	58.6262
21.73459	12.02046
39.01674	66.0105
31.64706	40
	22.48804
	21.73709
	38.01874
	31.64706
	44.87297
	21.85431
	17.70244
	23.46154
	25.46342
	12.64642
	44.375074

ED_Fig2D

	WT			44D		
	Mean	SD	N	Mean	SD	N
Day1	56.24533	3.644307	11	58	1.236667	22
Day2	53.76667	4.397797	11	54	3.140493	22
Day3	46.84333	5.576741	11	46	4.798626	22
Day4	30.92667	3.964393	11	29	4.168919	22
Day5	26.59467	3.065319	11	27	4.896637	22

ED_Fig2E

WT	44D
19.13	21.79
22.12	17.23
21.23	30.5
13.57	20.85
13.61	16.91
18.54	19.48
20.15	11.53
18.91	13.15
16.48	19.45
21.53	16.95
	18.8
	14.17
	19.36
	22.54
	17.02
	21.28
	12.15
	19.55
	16.94
	19.87
	14.17

ED_Fig2F

WT	44D
2	6
2	2
5	5
4	3
3	2
4	2
2	2
3	2
3	3
4	4
	3
	4
	2
	4
	2
	5
	2
	3
	4
	3
	4
