# Supplementary material for: Heart failure-induced cognitive dysfunction is mediated by intracellular Ca2+ leak through ryanodine receptor type 2
Source: Nat Neurosci. 2023 Jul 10;26(8):1365–78. doi: 10.1038/s41593-023-01377-6 (PMC10400432; doi:10.1038/s41593-023-01377-6)
Supplement: Source Data Extended Data Fig. 3 — Statistical source data. [file 41593_2023_1377_MOESM13_ESM.pdf]

ED_Fig3A

RyR2: IP- KI mouse tissues

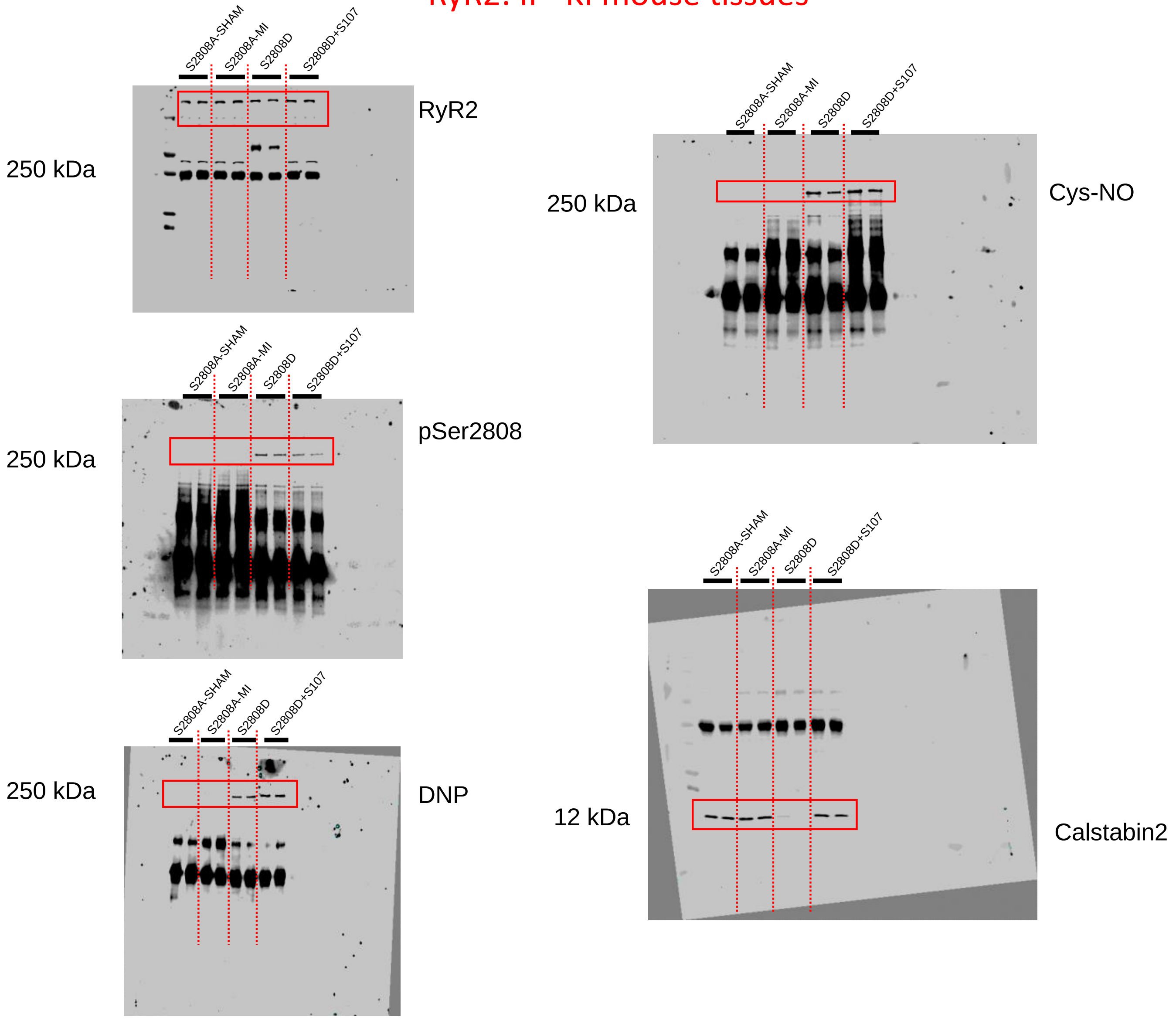

RyR2: IP- KI mouse tissues

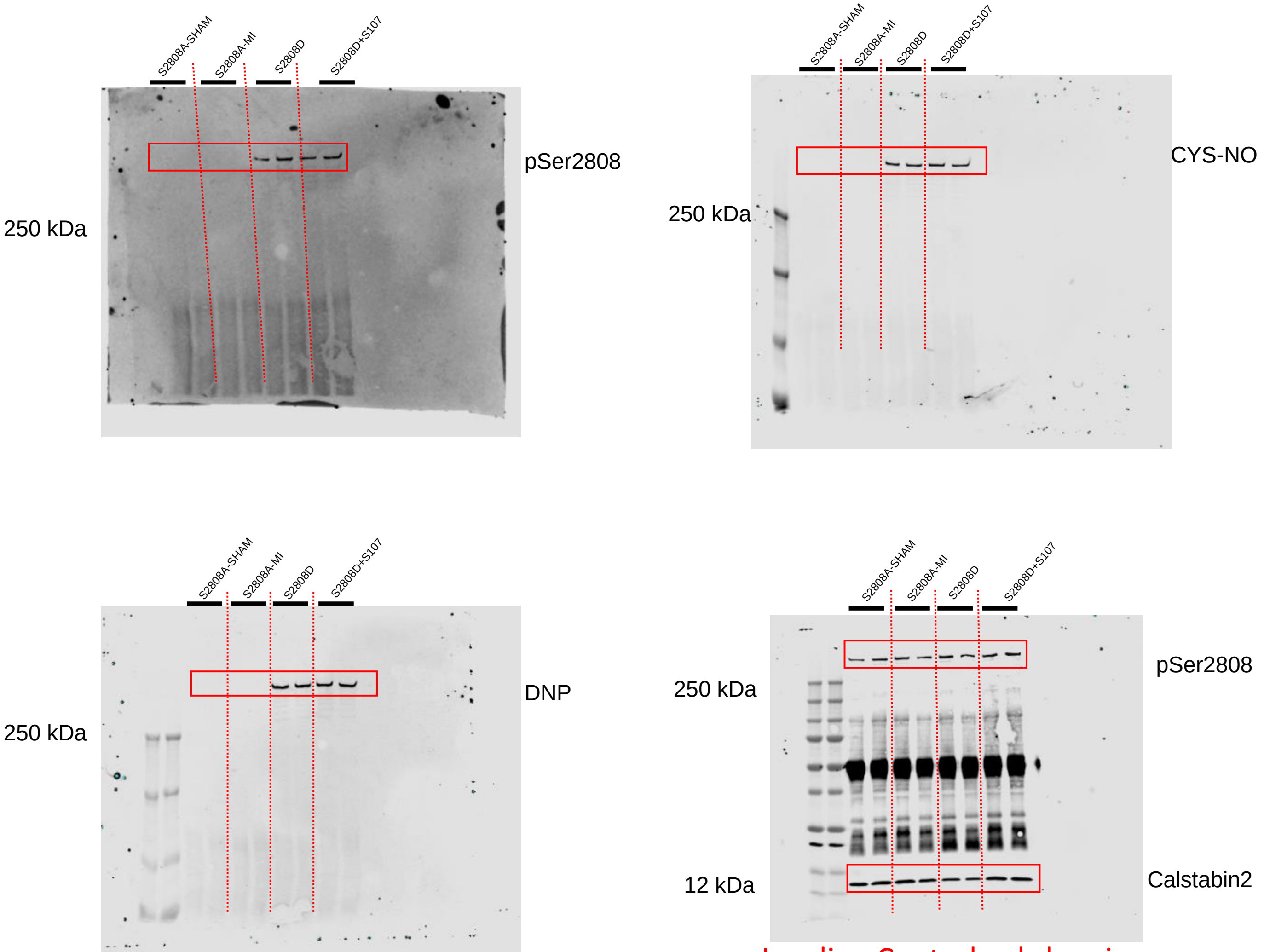

Loading Control gel showing even IgG and Total RyR2

ED_Fig3B-H

ED_Fig3B

	Sham2808				S2808MI				2808D				2808D+S107			
**pSer2808**	0.15	0.25	0.15	0.1	0.5	0.3	0.2	0.1	4	3.9	3.7	4.1	4	3.7	3.55	3.9
**DNP**	0.25	0.3	0.2	0.1	0.1	0.15	0.9	0.4	4.5	3.6	3.7	4	3.9	4.12	4.3	3.6
**Cys**	0.1	0.2	0.45	0.09	0.2	0.3	0.1	0.17	3.85	3.9	3.7	4.15	4	4.2	3.8	3.75
**Calstabin2**	4.2	3.8	4.3	3.75	3.9	4.5	3.7	4	0.85	1	1.3	0.5	4	3.9	3.6	3.75

ED_Fig3D

S2808Asham	S2808AMi	S2808D	S2808D+S107
14.70945782	19.1	36.7923728	15.0171254
10.00543855	11.8010941	40.2255786	11.80542326
15.2	18.6380744	35.7923728	14.0171254
9.78	10.9	41.2255786	10.80542326

ED_Fig3F

**S2808A-SHAM**	S2808A- MI	S2808D	S2808D+S107
0.00458	0.10548	0.02526	0.0107
0.00006	0.00476	0.16495	0.04587
0.00047	0.00446	0.03343	0.01843

0.00007	0.02495	0.19296	0.11215
0.00045	0.00684	0.35473	0.02085
	0.00286	0.11215	0.00036
**ED_Fig3G**			
S2808A-SHAM	S2808A- MI	S2808D	S2808D+S107
3.89757	10.0409	11.8206	7.35996
1.5	4.77588	10.6609	7.27178
2.95238	6.96552	16.5676	3.7
3.875	3.40203	6.33537	
3.63426		30.8811	

ED_Fig3H

**S2808A-SHAM**	S2808A- MI	S2808D	S2808D+S107
960.593	832.48	96.655	390.964
568.6	764.261	137.177	304.556
987.42	234.019	30.1748	231.932
767	208.79	131.932	704.5
641.62	753.213		
