# Supplementary material for: Heart failure-induced cognitive dysfunction is mediated by intracellular Ca2+ leak through ryanodine receptor type 2
Source: Nat Neurosci. 2023 Jul 10;26(8):1365–78. doi: 10.1038/s41593-023-01377-6 (PMC10400432; doi:10.1038/s41593-023-01377-6)
Supplement: Source Data Extended Data Fig. 4 — Statistical source data. [file 41593_2023_1377_MOESM14_ESM.pdf]

ED_Fig4A

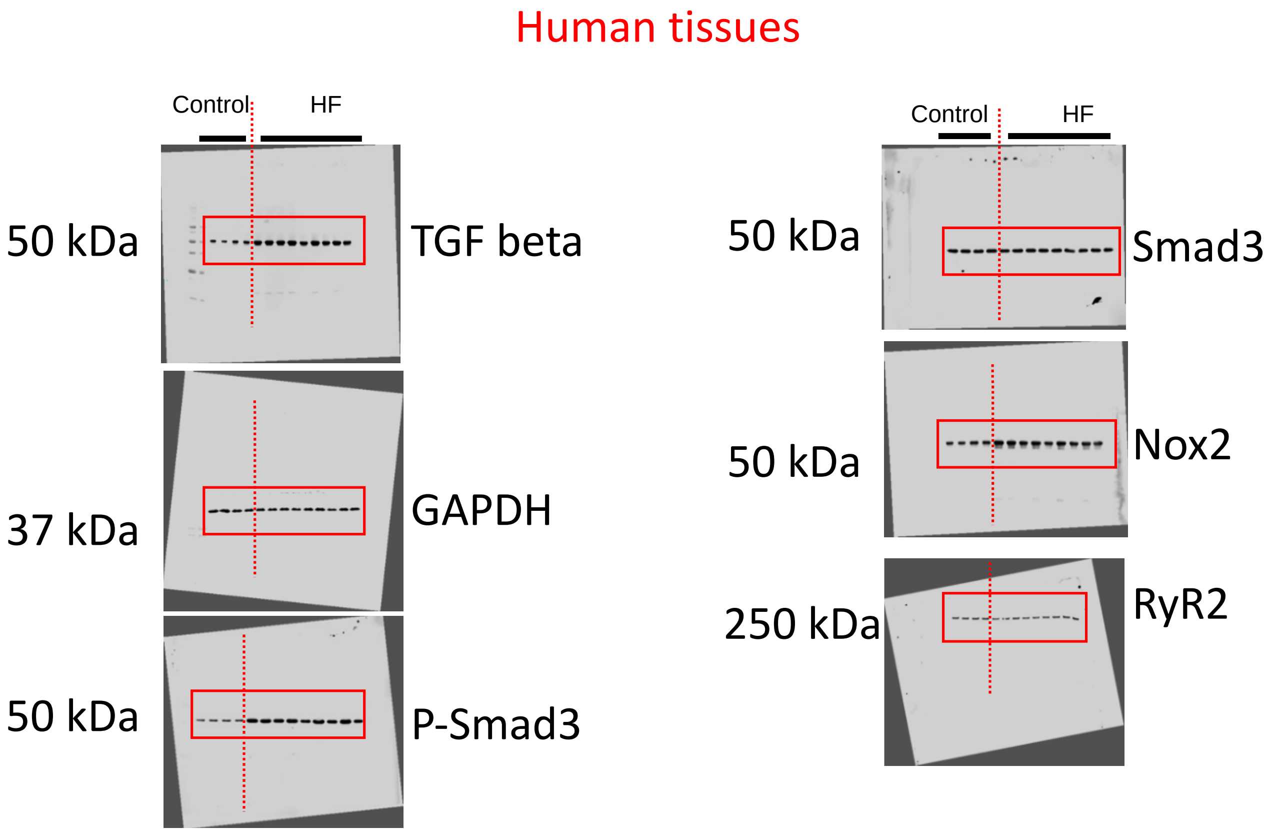

Human tissues
Control
HF
50 kDa
TGF beta
37 kDa
GAPDH
50 kDa
P-Smad3
Control
HF
50 kDa
Smad3
50 kDa
Nox2
250 kDa
RyR2

ED_Fig4B

Controls

TGF	0.912901	0.498183	1.184944	1.403971
pSmad/Smad	0.8	1	1	0.9
Nox2/RyR2	1	1.1	2	1.7

HF

3.387646	3.829572	3.549149	4.556748	2.273685	3.004507	3.297891	2.824021	2.131621
4	3.8	3.6	3.8	2.2	3.6	2.6	3.8	2.5
4	3.9	2.3	4	2.6	4	2.9	3.8	3.5

ED_Fig4C

Figure S2D-mouse tissues

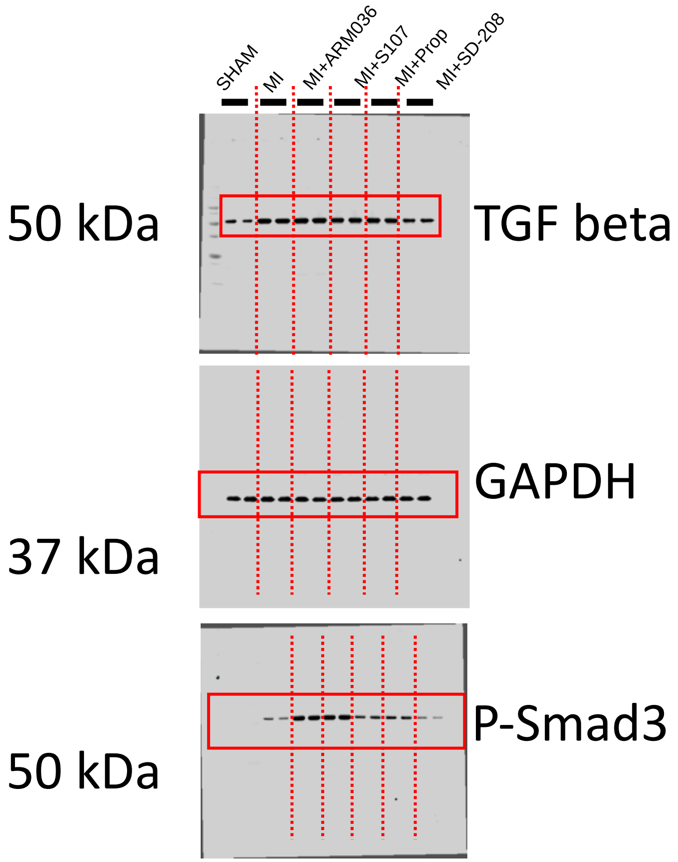

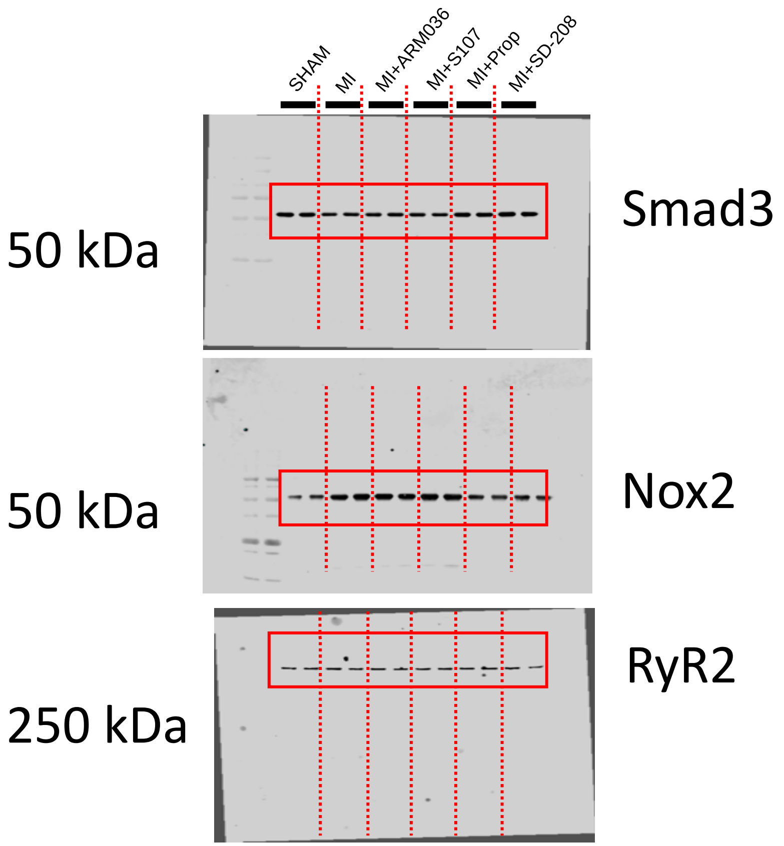

ED_Fig4D

	Sham					
TGF beta	1.227202	0.772798	1.05	0.95		
**p/Smad**	0.7	0.9	0.4	0.6	0.2	0.55
**Nox/RyR2**	0.5	0.7	0.4	0.5	0.1	0.7

	MI					
**TGF beta**	2.610166	2.744029	2.6	3		
**p/Smad**	4	3.8	3.6	3.9	4	3.1
**Nox/RyR2**	3.3	3.7	3.8	3.9	4.5	3.5

	MI+ARM					
**TGF beta**	2.531245	3.06409	2.8	2.3		
**p/Smad**	3.7	3.9	3.8	3.3	3.2	4.3
**Nox/RyR2**	3.8	3.6	4	3.7	3.5	4.3

	MI+S107					
**TGF beta**	2.056848	2.054783	2.5	2.2		
**p/Smad**	1.6	1.8	2.2	1.8	1.5	2.7
**Nox/RyR2**	3.5	3.8	2.5	2.9	3.5	2.6

	Prop			
**TGF beta**	2.650686	1.889506	2	2.3
**p/Smad**	1.9	2	2.2	1.8
**Nox/RyR2**	1.3	1.1	1.6	1.3

	SD			
**TGF beta**	1.329647	1.374367	1.1	1.45
**p/Smad**	0.7	0.6	0.6	0.8
**Nox/RyR2**	1.3	1.2	1.5	1
