# Supplementary material for: Heart failure-induced cognitive dysfunction is mediated by intracellular Ca2+ leak through ryanodine receptor type 2
Source: Nat Neurosci. 2023 Jul 10;26(8):1365–78. doi: 10.1038/s41593-023-01377-6 (PMC10400432; doi:10.1038/s41593-023-01377-6)
Supplement: Source Data Extended Data Fig. 10 — Statistical source data. [file 41593_2023_1377_MOESM15_ESM.pdf]

ED_Fig10B

SHAM	MI	MI+ARM036	MI+S107
0.353106	6.12479	3.267647	0.244688
0.26382	5.665606	3.98831	0.282953
0.155402	4.071218	3.67581	0.206422
0.353106	3.835249	4.007443	0.2128
0.372239	6.207698	2.559739	0.321218
0.161779	5.703871	3.171983	0.314841
0.595453	2.865861	3.765096	0.219177
0.346728	2.470453	4.058463	0.314841
0.174534	6.43729	0.952596	0.295708
	7.024024	3.018922	
	4.198769	2.884994	
	3.548259	3.567392	

ED_Fig10C

SHAM	C57MI	C57MI+ARM036	C57MI+S107
85.69684	280.5929	297.9121	228.2068
75.35482	314.153	272.9176	226.3461
74.17008	426.5078	250.4876	241.2957
85.89453	109.736	128.1086	131.2717
80.15465	110.4756	147.525	112.9433
77.67594	105.3302	149.289	122.5153
131.4804	117.4735	103.5123	70.33381
98.75949	130.3634	106.2592	63.67185
211.1793		98.36273	80.47537
69.98544			
116.8031			
92.84542			
